# Machine Learning and Bioinformatics Framework Integration to Potential Familial DCM-Related Markers Discovery

**DOI:** 10.3390/genes12121946

**Published:** 2021-12-02

**Authors:** Concetta Schiano, Monica Franzese, Filippo Geraci, Mario Zanfardino, Ciro Maiello, Vittorio Palmieri, Andrea Soricelli, Vincenzo Grimaldi, Enrico Coscioni, Marco Salvatore, Claudio Napoli

**Affiliations:** 1 Department of Advanced Medical and Surgical Sciences (DAMSS), University of Campania “Luigi Vanvitelli”, 80138 Naples, Italy; direzione.immunoematologia@unicampania.it; 2 IRCCS SDN, 80121 Naples, Italy; monica.franzese@synlab.it (M.F.); mario.zanfardino@synlab.it (M.Z.); soricelli@uniparthenope.it (A.S.); vincenzo.grimaldi@policliniconapoli.it (V.G.); marcosalvatore2.segreteria@gmail.com (M.S.); 3 Institute for Informatics and Telematics, CNR, 56124 Pisa, Italy; filippo.geraci@iit.cnr.it; 4 Department of Cardiovascular Surgery and Transplant, Azienda dei Colli, Monaldi Hospital, 80131 Naples, Italy; ciromaiello64@gmail.com (C.M.); vpalmieri68@gmail.com (V.P.); 5 Department of Exercise and Wellness Sciences, University of Naples Parthenope, 80133 Naples, Italy; 6 Division of Cardiac Surgery, AOU San Giovanni di Dio e Ruggid’Aragona, 84131 Salerno, Italy; coscionienrico@gmail.com; 7 Clinical Department of Internal Medicine and Specialistic Units, Division of Clinical Immunology, Immunohematology, Transfusion Medicine and Transplant Immunology (SIMT), Azienda Universitaria Policlinico (AOU), Regional Reference Laboratory of Transplant Immunology (LIT), 80131 Naples, Italy

**Keywords:** RNA-sequencing, heart failure, gene expression analyses, machine learning, dilated cardiomyopathy

## Abstract

Objectives: Dilated cardiomyopathy (DCM) is characterized by a specific transcriptome. Since the DCM molecular network is largely unknown, the aim was to identify specific disease-related molecular targets combining an original machine learning (ML) approach with protein-protein interaction network. Methods: The transcriptomic profiles of human myocardial tissues were investigated integrating an original computational approach, based on the Custom Decision Tree algorithm, in a differential expression bioinformatic framework. Validation was performed by quantitative real-time PCR. Results: Our preliminary study, using samples from transplanted tissues, allowed the discovery of specific DCM-related genes, including MYH6, NPPA, MT-RNR1 and NEAT1, already known to be involved in cardiomyopathies Interestingly, a combination of these expression profiles with clinical characteristics showed a significant association between NEAT1 and left ventricular end-diastolic diameter (LVEDD) (Rho = 0.73, *p* = 0.05), according to severity classification (NYHA-class III). Conclusions: The use of the ML approach was useful to discover preliminary specific genes that could lead to a rapid selection of molecular targets correlated with DCM clinical parameters. For the first time, NEAT1 under-expression was significantly associated with LVEDD in the human heart.

## 1. Introduction

Dilated cardiomyopathy (DCM) is a common heart muscle disease [1,2] leading to advanced heart failure (HF), and at the end stage, to organ transplantation. Heart transplantation occurs with an annual incidence of approximately 5–7 per 100,000 subjects in the entire population [3]. In addition to hereditary abnormalities, during HF, many factors have been associated with a number of molecular and conformational changes, such as transcriptional activation of fetal genes and changes in the cytoskeletal components of cardiomyocytes [2,4,5]. However, the specific targets for DCM onset have not yet been identified. In this context, measurement of left ventricular (LV) size and ejection fraction (EF) are the two central parameters for diagnosis, risk stratification, and treatment [6].

Several studies have highlighted the key role of long non-coding RNAs (lncRNAs) in the development and progression of heart disease, allowing them to be considered as a new class of circulating biomarkers [7,8,9]. Therefore, an accurate analysis of the complex signal networks underlying the disease requires global approaches to gene expression. The advent of new generation technologies has made it possible to obtain an analysis of the global gene expression profile of HF, representing a more sensitive method for quantifying gene transcripts [10].

**Figure 1:**
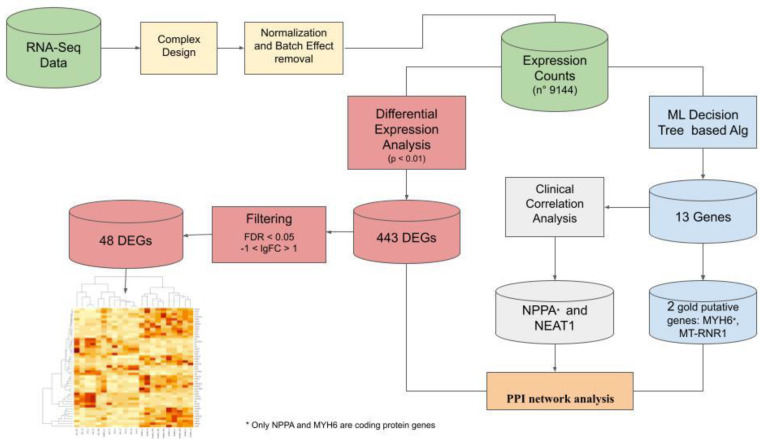
The framework of the integrated analysis. We started from RNA-seq data and performed a complex design drawing, then we normalized and corrected the batch effect. Finally we created a counts table for all 9144 genes. In the blue workflow we applied the ML approach, which led to the selection of two genes: *MYH6* and *MT-RNR1*. A sub-workflow, using a correlation analysis with clinical parameters (grey), led to the selection of *NPPA* and *NEAT1*. In the red workflow we used a classic differential expression analysis, which let, from a list of 443 DEGs, to a clusterization based on a list of 48 genes (after filter based on FDR and lgFC applications). A PPI network analysis brought together the results to discover connections.

In this study, the challenge was to identify targets that could significantly discriminate pathological from healthy groups. For this purpose, we used rare samples from transplanted myocardial tissues. The preclinical diagnosis of DCM could substantially reduce morbidity and mortality, allowing an early use of cardio-protective therapy. In order to identify the pivotal targets, we performed an RNA-seq on myocardial tissues collected from both HF patients and healthy donors, during organ transplant. The use of ML-based methods, improved the identification of functional genes through the construction of a gene regulatory network [11,12]. In this perspective, in order to realize an automated diagnostic method that can allow a translational approach for precision medicine, we show the advantages of a ML approach to discover the non-naive expression relationships that lead to DCM. Aware of the potential applications of ML-based biomarker panels in the precision medicine, we took up the challenge of centering our approach on explainable ML algorithms. The two approaches allowed the highlighting of a selected group of genes and gene regulators, such as some protein-coding genes, a mitochondrial gene, and a lncRNA, which could facilitate the identification of new therapeutic strategies and provide clinicians with an interesting calculation tool capable of distinguishing a functionally damaged heart from a healthy one. A comprehensive graphical description of all frameworks is shown in Figure 1.

## 2. Methods

### 2.1. Patient Cohorts and Ethics Approval 

All subjects gave their informed consent for inclusion before they participated in the study. The study was performed according to protocols approved by the Ethical Committee of Monaldi Hospital (protocol 438) and in conformity with the principles outlined in the Declaration of Helsinki. Left-ventricular myocardial tissues were directly dissected after heart transplantation, were snap-frozen in liquid nitrogen and stored at −80 °C. Tissue samples were collected from familial cardiomyopathy patients (n.22), of which n.2 were collected from a previous study (GSE71613). Control myocardial tissue samples were acquired from non-failing heart donors (n.7 new samples and n.4 from GSE71613) that were not transplanted due to non-cardiac reasons. For further details, see Supplementary Materials.

### 2.2. RNA-Seq Data Analysis 

**Figure 2:**
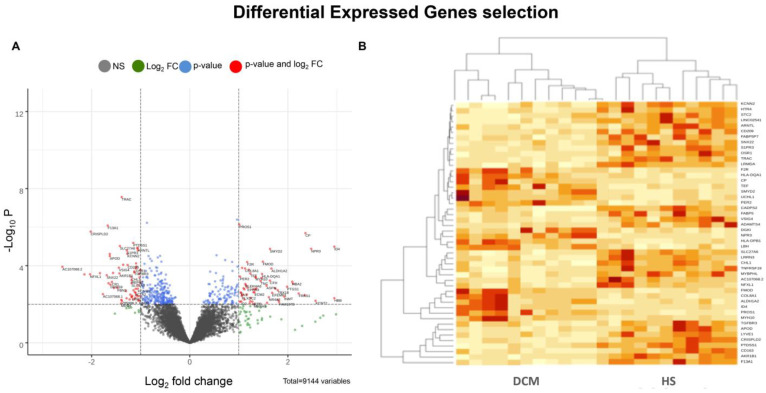

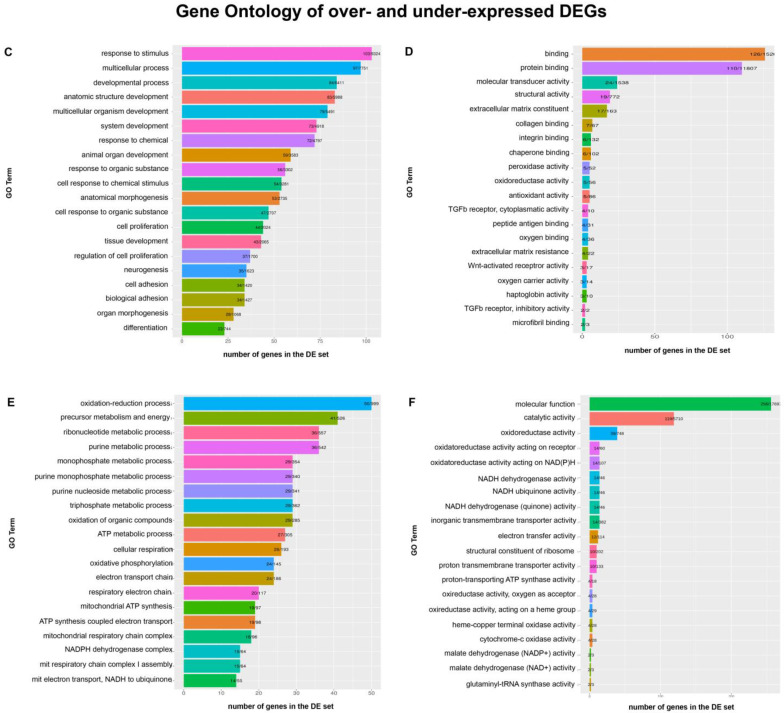
(**A**) Volcano plot displaying differential expressed genes (DEGs) between DCM patients and healthy subjects (HS). The y-axis is related to the mean expression value of log10 P and the horizontal x-axis displays the log2 fold change (log2FC) value. Positive and negative x-values represent up- and down-regulation, respectively. We highlighted in red the genes with a FC of ±1 and Log10 P > 2. (**B**) Heatmap displaying expression levels for a subset of n.48 DEGs (FDR < 0.05, logFC > 1/logFC < −1) related to DCM patients. (**C**–**F**) Gene Ontology plots of over-expressed (**C**,**D**) and under-expressed terms (**E**,**F**). The first n.20 Biological Process (BP) and n.20 Molecular Function (MF) terms organized by gene number for each term and with increasing *p*-value.

IlluminaTruSeq RNA Sample Preparation Kit was used to perform cDNA library preparation and the libraries were sequenced at high coverage on the Illumina HiSeq2000 [11,13,14]. Raw counts were quantified by Feature Counts (Rsubread v2.4.3) and combined with RNA-seq data from GSE71613 dataset (using a batch effect correction). Based on statistical filtering *p*-value < 0.01, among about 9000 genes (Figure 2A), 443 differential expressed genes (DEGs) were identified (Tables S1 and S2) and the high DEGs (FDR < 0.05, |log2 FC| > 1) were shown through a heatmap (Figure 2B). Raw sequencing data (fastq files) of biological samples were submitted to NCBI BioProject Database with as PRJNA667310 accession id (https://www.ncbi.nlm.nih.gov/bioproject?term=PRJNA231566, accessed on 9 November 2021).

### 2.3. Gene Ontology Analysis

Gene Ontology (GO) analysis was conducted on over- and under-expressed genes using “GOANA” from Limma R package (v3.42.2) [15]. Plots of the first 20 categories by increasing *p*-value were obtained by ggplot2 (v3.3.0) and were shown in Figure 2C–F. For further details, see Supplementary Materials.

### 2.4. Custom Decision Tree Analysis

Starting from normalized raw counts matrix for 22 patients, we considered a ML algorithm by using the leave-one-out cross-validation approach for training and testing due to small, but valuable sampling (Figure S1). We repeated the training procedure n.22 times using n − 1 samples as training examples and tested the classifier with the remaining one in order to use the widest possible set of samples for training without sacrificing testing and also to estimate the independence of the training phase from the random choice of the examples. For further details, see Supplementary Materials.

### 2.5. Clinical Correlation Analysis and Protein–Protein Interaction Network 

Spearman’s rank correlation was conducted on DCM-related panel genes (n.13) selected during features selection step. A Spearman’s ρ greater than 0.5 and significant *p*-value (*p* < 0.05) was set as threshold to identify a possible association between expression values and clinical parameters. A protein–protein interaction network was built using all *p*-value-filtered DEGs (n.443) inferred on a *NPPA* protein-coding gene, which was correlated with echocardiographic parameters, using String (May 2020 online version) tool [16]. For further details, see Supplementary Materials.

### 2.6. Experimental Validation by qRT-PCR 

Quantitative reverse transcriptase PCR (qRT-PCR) was carried out to validate data obtained. In the Table 1 was reported primers sequences used. For further details, see Supplementary Materials.

## 3. Results

### 3.1. Identification of Set of DEGs Able to Clusterize Healthy/Sick Sample in DCM

We investigated the changes occurring in the cardiac tissue transcriptome of familial DCM patients and HS that underwent surgical for heart transplantation by a NGS technology. Clinical characteristics of all subjects were reported in Table 2. 

Specifically, RNA-seq analysis revealed 9144 candidate genes (GRCh38) (Figure 2A and Table S1). Among these, 8461 were annotated as protein coding, while 230 were annotated as lncRNAs. Subsequently, we selected 443 DEGs with a *p*-value < 0.01 (Table S2), where 154 were up- and 289 were down-expressed genes, which were annotated as protein coding. The degree of this subset was variable between DCM and healthy tissues, with log2FC ranging from −2.59 to +3.14. About 65% of DEGs were significantly under-expressed in the DCM group. Among these (log2FC < −2), there was AC107068.2, which is annotated as lncRNA and works as antisense on atrial natriuretic peptide-converting enzyme (*CORIN*), encoding a member of the type II trans-membrane serine protease class of the trypsin superfamily. The encoded protein converts pro-atrial natriuretic peptide (*pro-ANP*) to biologically active atrial natriuretic peptide (*ANP*), a cardiac hormone that regulates blood volume and pressure. Among its related pathways are “Cardiac Conduction and Myometrial Relaxation” and “Contraction Pathways”. Experimental and clinical studies attributed the protective effect of cardiac *CORIN* in DCM patients and HFrEF development [17]. Moreover, *CORIN* over-expression significantly reduced the development of myocardial fibrosis and prolonged life in mice with DCM [17]. Similarly, among the most over-expressed genes (log2FC > 2), there was natriuretic peptide receptor 3 (*NPR3*). It has been shown that loss-of-function mutations in *NPR3* result in increased NPR-A/B signaling activity and cause a phenotype marked by CV abnormalities and enhanced bone growth [18]. We also selected the most important DEGs by FDR < 0.05 and |log2FC| > 1, obtaining a list of 48 genes (17 over-expressed and 31 under-expressed), from which we generated the heatmap (Figure 2B). These genes were able to cluster data in the HS and pathologic subject groups.

### 3.2. Improved Target Genes Selection by an Original Machine Learning Approach 

Feature selection leverages on the assumption that most genes have similar and low expression profiles that do not influence the phenotype, and thus they can be discarded from the analysis. Performing gene clustering we observed that most genes aggregated into few big homogeneous clusters (Figure S2A,B). However, only the centroids of a few (small) of them have at least one detectable expression level, causing the removal of clusters with no detectable signals, leaving only about 85 genes.

**Figure 3:**
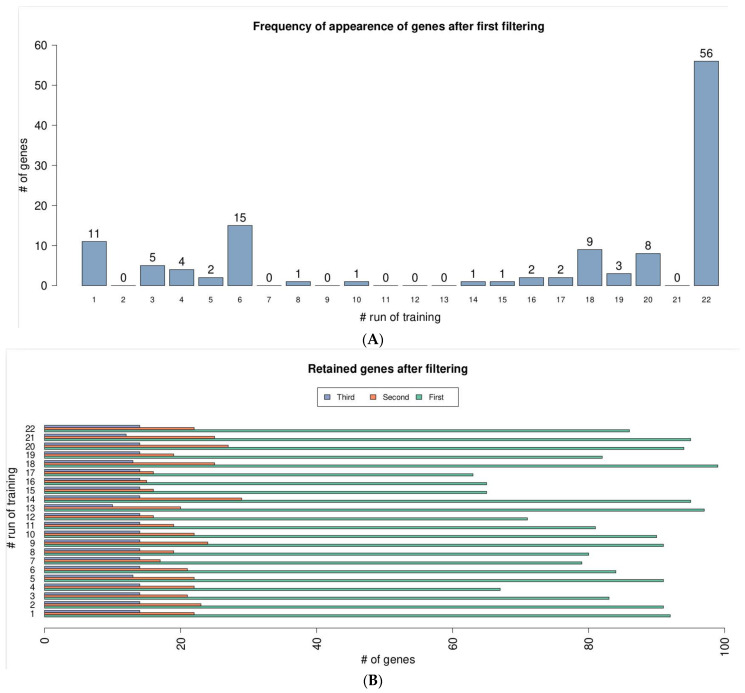

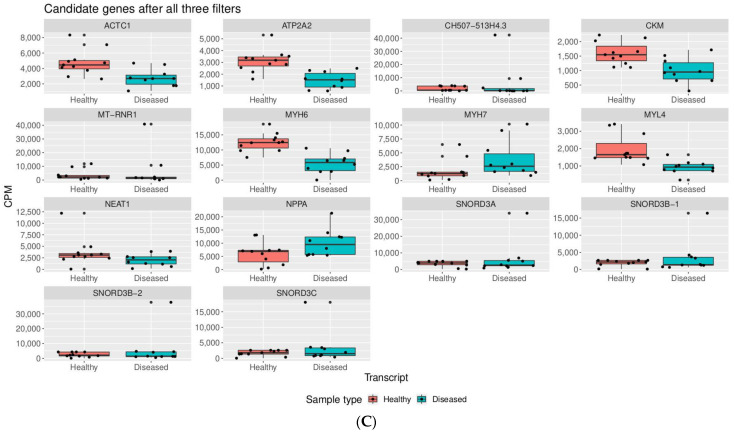
(**A**) Gene frequency of appearance after first selection. (**B**) Barplot reporting the number of retained genes after multiple feature selection step for every run of training/test. (**C**) DCM-related panel genes identified during feature selection step.

Starting from the assumption that relevant genes should emerge independently from the training examples, we experimentally investigated the stability of results returned by our feature selection and classification. To this end, we exploited the fact that the leave-one-out cross-validation approach runs a feature selection and training for each element of the dataset allowing us to compare them. For each gene retained downstream any run of the k-means based selection, we computed its frequency of appearance. Out of 115 distinct genes, more than half have been reported in all 22 runs (Figure 3A). This result proved that our feature selection method was not biased by randomization. Although detectable, most of the genes that passed clustering filtering have a rather constant expression level (Figure S3). The second filtering removed most of these genes, thus requiring a fold change FC of at least 0.5 between healthy and DCM samples. Even using this permissive threshold, the average number of retained genes dropped from on average n.85 to n.21 (Figure S4). The rationale of the last filtering step was that of removing low frequency genes that, if used into the classification model, would generate a potentially non-general decision tree. This filter was the least aggressive (Figure 3B). The outcome of the entire feature selection process was a list of 13 genes: *ACTC1*, *ATP2A2*, *CH507-513H4.3*, *MT-RNR1*, *MYH7*, *MYH6*, *MYL4*, *NEAT1*, *NPPA*, *SNORD3A*, *SNORD3B-1*, *SNORD3B-2* and *SNORD3C* (where *ATP2A2* and *MYL4* were also identified by differential expression analysis), which we called DCM-related panel genes. As for all the supervised methods, the decision tree training depended on the set of examples (i.e., samples) and on the selected features, such as genes. In general, it is not unlikely that even slightly different training sets lead to different trees. We examined the 22 decision trees produced with both the standard implementation and our modification of the splitting criterion finding that, in the former case, found the resulting trees were all different among each other, whereas in the latter case they were only of two types with one to be a specialization of the other. In detail, an examination of these two alternatives showed that this phenomenon derived from the composition of the training set allowing the runs with an extra healthy sample to learn a more accurate classification model (Figure S5). Finally, starting from DCM-related panel genes, our specialized tree identified two gold putative genes associated with DCM disease, which guided the ML process: *MYH6*, which was classified as DCM-related genes and *MT-RNR1*, a mitochondrial-derived peptide MOTS-c, which regulates metabolic homeostasis. Moreover, we investigated possible associations between the outcome of the entire feature selection process (13 genes) and some available clinical features (Figure 3C). The results showed a significant association between the expression levels of *NEAT1* and LVEDD (Rho = 0.73, *p* = 0.05) and that *NPPA* expression level was positively correlated with the Left Ventricular End-Systolic Dimension (LVESD) (Rho = 0.96, *p* = 0.0004). The whole framework, then, made it possible to focus our attention on four genes (*MYH6*, *MT-RNR1*, *NPPA* and *NEAT1*), neither of which would have been selected considering differential expression analysis results filtered by *p*-value.

### 3.3. Validation of Potential DCM-Related Expression Targets

**Figure 4:**
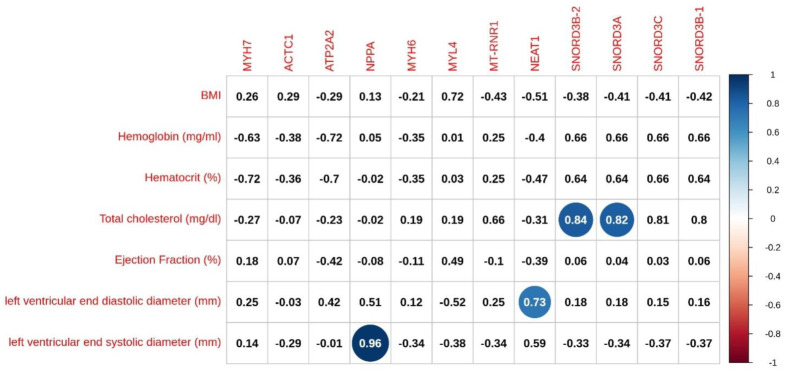
Quantitative real-time-polymerase chain reaction (qRT-PCR) analysis data for the top four most significantly target genes, such as protein coding genes (*MYH6* and *NPPA*), mtRNA (*MT-RNR1*), and lncRNA (*NEAT1*), in DCM patients compared to HS. Three technical replicates were performed for each tissue sample. The relative expression levels are reported as the fold change derived from mean average of sample-specific Ct values.

Many of 48 genes selected from differential expression analysis were already well-known just as genes involved in heart diseases. For example, the top five (by *p*-value) differentially expressed genes (DEGs) in the list are all linked to cardiovascular atypicalness: T Cell Receptor Alpha Constant (*TRAC*) was assigned by Human Phenotype Ontology (HPO) as a gene involved in abnormality of the cardiovascular system, while the Human Protein Atlas (*HPA*) defines F13A1, PROS1 and CP as “candidate cardiovascular disease genes”; Cysteine Rich Secretory Protein LCCL Domain Containing 2 (*CRISPLD2*) was described as gene involved in the promotion of cardiac ischemia/reperfusion injury [19]. We performed qRT-PCR for the selected four most significantly target genes: *MYH6*, *NPPA*, *MT-RNR1*, and *NEAT1*, both in DCM and HS. The selected targets showed similar expression trends to those that were observed in the analyses described in Section 3.1, finding that *MYH6* and *NEAT1* were down-regulated (FC = −1.47 and −1.53 times, respectively), while *MT-RNR1* and *NPPA* were up-regulated (FC = 2.05 and 1.61 times, respectively) in DCM patients compared to HS (Figure 4). Moreover, *MYL4* detected by both bioinformatic and ML analysis, was already validated in a previous study [11], while *ATP2A2* is reported to be associated to “arrhythmogenic right ventricular cardiomyopathy” pathway by KEGG.

### 3.4. Results Integration Using PPI Network Analysis 

**Figure 5:**
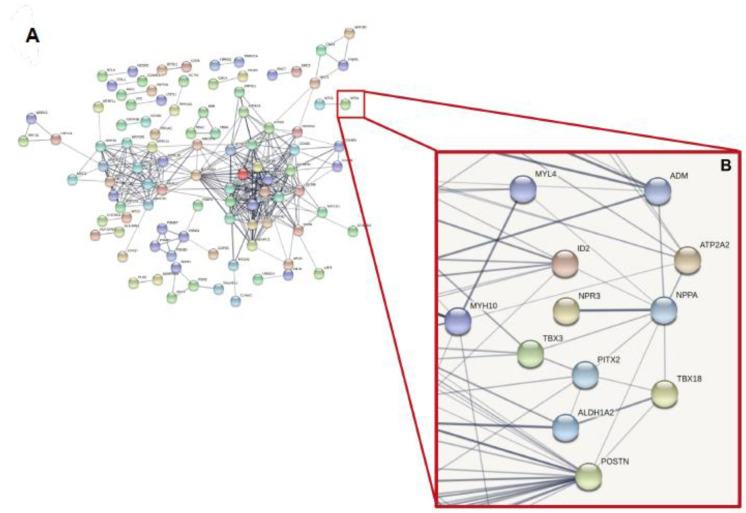
Protein–protein interaction networks functional enrichment analysis for the differential expressed genes and NPPA gene selected from correlation analysis with clinical features. (**A**) Physical interaction with a STRING score >0.7; (**B**) non-physical interaction (from text mining and databases) of NPPA with differential expressed genes.

**Figure 6:**
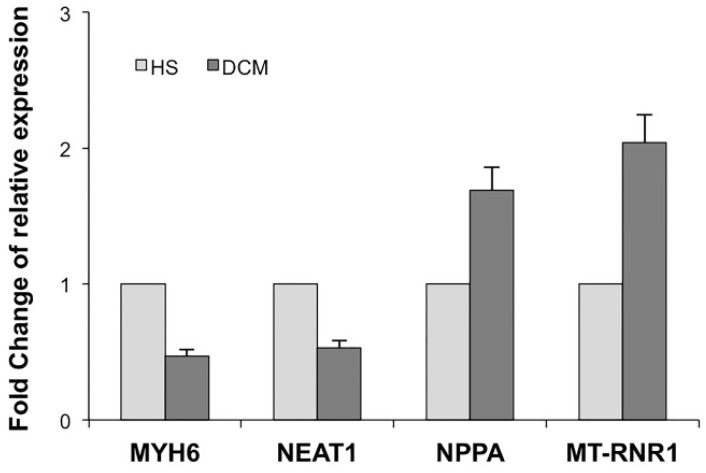
Correlation plot of 13 selected genes (from ML analysis) with seven clinical features. The circled values have a significant correlation (*p* < 0.05).

To create a match between bioinformatics and ML results we used the STRING database to perform a PPI network analysis using as input the most significant DEGs (443 genes) and the two protein-coding genes identified by the machine learning approach (*NNPA* and *MYH6*). The resulting network contained the subset of proteins that formed physical interactions with at least one other member in the list. Figure 5 underlined the physical interaction of *NPPA* with *NPR3*, the atrial natriuretic peptide receptor 3, which regulates blood volume and pressure, pulmonary hypertension, and cardiac function as well as some metabolic and growth processes (score 0.89). In addition, several interesting associations were classified as *Textmining* interactions. In particular we noticed the ATPase Ca++ Transporting Cardiac Muscle Slow Twitch 2 (*ATP2A2*) gene, selected by both our bioinformatic and ML approach, was involved in cardiac conduction and Calcium Regulation in the Cardiac Cell (score 0.58); T-Box Transcription Factor 18 (*TBX18*), transcriptional repressor was involved in developmental processes of a variety of tissues and organs, including the heart and coronary vessels, the ureter, and the vertebral column (score 0.48); Adrenomedullin (*ADM*) was involved in regulation of the force of heart contraction and in congestive heart failure (score 0.55); Periostin (*POSTN*),which encodes a secreted extracellular matrix protein that functions in tissue development and regeneration, including wound healing, and ventricular remodeling following myocardial infarction and associated with myocardial infarction (score 0.41); and Paired Like Homeodomain 2 (*PITX2*), which plays a critical role in the intermediate steps controlling left–right asymmetry, cardiac morphogenesis, and embryonic rotation (score 0.46). Data obtained was validated by qRT-PCR (Figure 6).

### 3.5. GO Analyses for a More Whole Biological Picture

We associated differentially expressed mRNAs with two structured networks (Biological Process—BP and Molecular Function—MF). Only GO categories with *p* < 0.05 were considered and the first 20 are plotted in Figure 2C–F, ordered by increasing *p*-value. All genes, resulting in 1318 over-expressed genes, were specifically 1158 terms for BP (Table S3) and 160 terms for MF (Table S4), whereas 674 under-expressed genes included 495 terms for BP (Table S5) and 176 terms for MF (Table S6). The most significant BP terms in over-expressed genes were especially involved in tissue/organ development and in signal responses, such as “cell adhesion” (Figure 2C). Instead, the most represented MF terms were linked to binding and structural activity molecular functions (“protein binding”, “collagen binding”, “extracellular matrix structural constituent”, etc.) (Figure 2D). The most significant BP terms in under-expressed genes were especially involved in metabolic, cellular respiration, and mitochondrial BP (Figure 1E). This was evident also in MF terms, clustering into “catalytic activity”, “oxidoreductase activity” and “NADH dehydrogenase activity” (Figure 2F). The GO analysis also showed many over-expressed genes linked to BP involved in the cardiac tissue development, differentiation, and morphogenesis (“cardiac development and differentiation”). Some under-expressed genes were also associated with regulation of heart rate and contraction. All genes for each GO term were showed in Tables S3–S6.

## 4. Discussion

**Figure 7:**
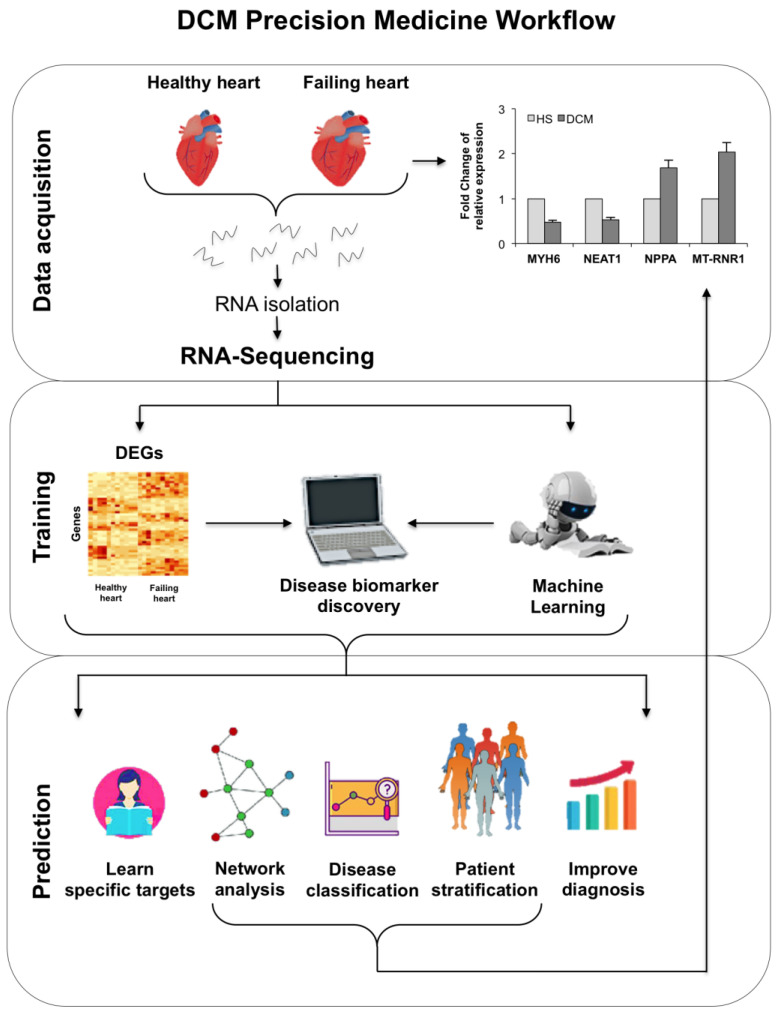
DCM Precision Medicine WorkflowAll *p*-value-filtered DEGs (443) inferred on *NPPA* protein coding gene, which was correlated with echocardiographic parameters, was used to perform Protein-Protein Interaction network. The picture shows String analysis.

In this study, we identify four four main altered target genes, *NPPA, MYH6* (protein-coding gene), *MT-RNR1* (a rRNA-coding gene deregulated in mitochondria) and *NEAT1* (gold lncRNA). Moreover, our analysis showed that a set of 48 genes was able to cluster our samples in HS and pathologic subject groups. In order to identify targets that could allow the individuating of the fundamental target-point in DCM status, we used rare samples from transplanted myocardial tissues. Applying both traditional RNA-seq bioinformatic analyses, we evaluated the difference in gene expression [11], whereas through an original ML approach, we improved the number of possible gene targets selected and involved in DCM patients. Differential expression analysis is generally based on the assumption that a relatively small group of genes are clearly deregulated between the pathological condition compared to healthy status. To characterize different phenotypes, genes interact with each other by regulating multiple processes at the same time, thus generating some phenotypes that can depend on complex non-linear relationships. This approach is not necessarily based on “chance”. Learning the first cascades of deregulated signals would therefore allow us to gain a more complete understanding of the phenotype of interest and could help discover regulated transcriptional genes involved in complex biological processes [12]. Therefore, starting from the count table, and from the generation of a DEG list, we applied ML and bioinformatic approaches, which allowed us to selectively differentiate HS and DCM groups. Moreover, through the ML analysis, we observed that most genes were aggregated into few big homogeneous clusters (Figure 7). Specifically, in the our preliminary study, for the first time and on hard to acquire samples from transplanted tissues, we highlighted that *MT-RNR1*, one of two gold putative genes selected by our ML framework, was up-regulated in DCM patients compared to HS [20]. *MT-RNR1* mutations have been associated with a rare genetic condition that can affect multiple body parts, including skeletal muscles, the heart, the brain, or the liver. Common clinical manifestations include myopathy, hypotonia, and encephalomyopathy, lactic acidosis, and hypertrophic cardiomyopathy [21]. Mitochondrial DNA (mtDNA) is a small independent circular genome in humans [22]. It is particularly vulnerable to reactive oxygen species (ROS), which are established determinants of DNA methylation alterations, since mitochondria lack protective histones and have a relatively inefficient DNA repair system [22]. Although mtDNA methylation has been observed for years, methylation mechanism in the mtDNA genome has been rarely and inconsistently studied [23]. We also observed that *NPPA* over-expression generated heart intricate PPI network interactions. Recently, it has been reported that the size and systolic and diastolic function of LV and RV over time, and their rate of change, are associated with the risk of transplantation and mortality in infant DCM [6]. Therefore, changes in these parameters could be useful for predicting clinical outcomes. Interestingly, our analyses reported that the *NPPA* expression level was positively correlated with the LVESD (Rho = 0.96, *p* = 0.0004) (Table S7) and that there was a physical interaction between *NPPA* and *NPR3* (the atrial natriuretic peptide receptor 3), as shown by PPI analysis. The *NPPA* gene encodes the atrial natriuretic peptide (*ANP*), which is a key member of the natriuretic peptide family [24]. In particular, the *ANP* lowers blood pressure through several mechanisms of actions in the kidney where it increases vascular permeability, inducing relaxation of vascular smooth muscle cells (VSMCs). Literature data also report that mutations in the *NPPA* gene are linked to atrial fibrillation [25] and our results confirm that GO terms and *NPPA*-enriched KEGG pathways are essential in HF. Therefore, *NPPA* represents an essential gene-associated disease to use as a potential therapeutic target in DCM. Finally, both approaches revealed a significant alteration of many lncRNA, in agreement with our previous report [11]. Although gene expression profiling studies have revealed that lncRNAs are regulated in a tissue- and cell-type specific manner, few cardiac lncRNAs have been studied. Specifically, the literature has reported that a multivariate statistical analysis of large cohorts, on 106 myocardial infarction (MI) patients and 85 controls, indicate that *NEAT1* levels were altered in the peripheral blood mononuclear cells (PBMCs) (*p* = 0.001) by post-MI status, independent of statin intake, LVEF, LDL- or HDL-cholesterol, or age [26]. Furthermore, *NEAT1* silencing in VSMCs resulted in enhanced expression of SM-specific genes and attenuated VSMC proliferation and migration. Conversely, over-expression of *NEAT1* in VSMCs showed the opposite effects. These in vitro findings were further supported by in vivo studies, in which *NEAT1* knockout mice exhibited significantly decreased neo-intima formation following vascular injury, due to attenuated VSMC proliferation [27]. Recently, Zou G. et al. demonstrated that the *NEAT1*/miR-140-5p/HDAC4 axis was altered in DCM mice [28]. Notable, for the first time, we observed and validated that *NEAT1* was under-expressed in DCM patient tissues and there was a significant association between the expression levels of *NEAT1* and LVEDD (Rho = 0.73, *p* = 0.05) according to severe functional status (NYHA-classIII) (Table S7)**.** Despite this, *NEAT1* functions still remain largely unknown, this important lncRNA could decrease the transcription of miR-140-5p, thus positively regulating histone deacetylase4 (HDAC4) expression [19]. It could represent an important DCM clinical-diagnostic target to highlight HF severity. 

## 5. Conclusions

In conclusion, including an original ML method in a classic RNA-seq bioinformatic analysis, we showed that some additional features could emerge, and it is possible to draw out a more complete biological picture of the pathophysiology of HF [29]. Although a limitation of the study may be the low sample size due to the type of biospecimen, as these samples are rare, the study represents a significant contribution in the research area investigated.

## Figures and Tables

**Table 1:** Primers sequences for RNA-seq validation experiments.

Primer Name	Forward	Reverse	Product Size (bp)	RefSeq Accession Number
*MYH6*	CTGGCCCTTCAACTACAGAA	TGTTCATCTCGATCTGCACG	196	NM_002471
*NPPA*	GCTTCCTCCTTTTACTGGCAT	CTTCTTCATTCGGCTCACTGA	180	NM_006172
*MT-RNR1*	CCACGATCAAAAGGAACAAGC	CTCTTTACGCCGGCTTCTATT	208	NC_012920.1
*NEAT1*	TGTGTAGGTGGGGAGTACTTT	CACTTAGACCCAAATCCCAGG	179	NR_131012

**Table 2:** Clinical characteristics of patients.

	DCM	Controls	*p* Value
	(*n* = 11)	(*n* = 11)	
Age	49.36 ± 16.10	30.64 ± 13.06	0.007
Sex (% number of male)	73.00%	63.60%	0.690
BMI	23.49 ± 3.12	n.a.	-
Hemoglobin (mg/mL)	13.54 ± 1.89	n.a.	-
Hematocrit (%)	39.30 ± 4.19	n.a.	-
Total cholesterol (mg/dL)	143.30 ± 48.60	n.a.	-
Echocardiographic parameters			
Left ventricular end-diastolic diameter (mm)	7.05 ± 0.72	n.a.	-
Left ventricular end-systolic diameter (mm)	6.13 ± 0.81	n.a.	-
NYHA class, number of patients			
III	8	n.a.	-
IV	3	n.a.	-

## Data Availability

Not applicable.

## Supplementary Materials

The following are available online at https://www.mdpi.com/article/10.3390/genes12121946/s1, Figure S1: Heatmap expression counts of DCM and HS patients after normalization step and batch effect correction; Figure S2: Piechart reporting the gene aggregations after clustering step; Figure S3: Heatmap of expression levels for all DCM and HS patients after clustering step; Figure S4: Boxplot of expression levels for retained genes after clustering filter; Figure S5: Boxplot of expression levels for selected genes after clustering, considering the fold change threshold of at least 0.5 between HS and DCM samples; Table S1: Differential expressed genes (DEGs) with *p* value < 0.01; Table S2: Protein-coding and ncRNA; Table S3: Gene Ontology Biological Process for over-expressed genes; Table S4: Gene Ontology Molecular Function for over-expressed genes; Table S5: Gene Ontology Biological Process for under-expressed genes; Table S6: Gene Ontology Molecular Function for under-expressed genes; Table S7: Correlation analysis between DCM-related genes with clinical and echocardiographic parameters in function of severe functional status (NYHA-class III).
