# Supplementary material for: Machine Learning and Bioinformatics Framework Integration to Potential Familial DCM-Related Markers Discovery
Source: Genes (Basel). 2021 Dec 2;12(12):1946. doi: 10.3390/genes12121946 (PMC8701745; doi:10.3390/genes12121946)
Supplement: Supplementary file 1 [file genes-12-01946-s001.zip › genes-1482169-SI.pdf]

Methods

Custom Decision Tree analysis

The cohort of the study included n.22 elements (n.11 healthy and n.11 DCM individuals), derived from two our independent RNA-seq experiments. In order to mitigate the batch effect and library biases, we applied the normalization. As shown in **Figure S1**, the normalization was reasonably effective in reducing batch effect even if some residual trace remains noticeable. Since we had a small, but valuable sampling, and ML algorithms works with a large number of samples to analyze, we used leave-one-out cross-validation approach for training and testing. In short, we repeated the training procedure n.22 times using n-1 samples as training examples and tested the classifier with the remaining one. The advantage of this approach in our setting is two fold: on the one hand, it allows to use the widest possible set of samples for training without sacrificing testing, on the other hand, it allows to estimate the independence of the training phase from the specific (random) choice of the examples. A further constraint, we handled to let the classifier be of practical usefulness was that of prioritizing, in the training phase, genes with an expression profile that can be measured indifferently with NGS and qPCR. In particular, the two sample groups induced by the bisection in a branch of the decision tree should have profiles different enough to be easily separated even with cheap low sensitive techniques. To this scope, before the actual training phase, we performed an ad-hoc feature selection. We assumed and verified that most of the genes have expression as low as they couldn't be used as biomarkers. Setting a cutoff threshold could potentially include/exclude genes with similar expression profiles only on the basis of a negligible distance from the threshold. We solved this problem by clustering genes and using centroids as representatives of all cluster members. This ensures that genes with similar expression are both either filtered or retained. We used a conservative threshold requiring the maximum value of the centroid on a sample to be higher than the lowest 15% of the expression interval. As per clustering, we used the k-means algorithm endowed with the MacQueen procedure described in to select initial centroids [MacQueen, 1967]. We used a large value for the number k (as large as k=50) of clusters to ensure high within-cluster homogeneity and applied a second (weak) filtering in cascade after clustering. For each gene, we computed the average expression of the healthy and DCM populations, and then we removed elements with Fold Change (FC) <0.5. It is important to note that this is by no means the same as holding only DEGs. In fact, the use of average values in the computation introduces a certain degree of slackness

allowing also non-DEGs to pass the filter. Moreover, a threshold as low as 0.5 makes this filtering rather permissive. The rationale of this second filter is that of balancing two needs: on the one hand letting the classifier be free to select genes according to its policy and, on the other hand preventing the noise of NGS data and the limited number of examples to deceive or bias the classifier. If, on the one side, the leave-one-out cross-validation approach offers the possibility of using all the samples either for training and testing, all the independent training procedures do not guarantee to converge to the same model. In the presence of small datasets, these models can be very different causing the impossibility of distilling a general one. In order to mitigate this phenomenon, we applied a further filter in cascade to the two described above. In short, we counted for each gene the number of times it passed the first two filters and retained only genes with counts higher or equals to 20. Downstream feature selection, we used decision trees as a classifier. The huge advantage of this algorithm stands in its natural interpretation as a series of binary choices that narrow the number of compliant samples until reaching a state where only elements of the same class remain. Classifying a new sample reduces to verifying the constraints and assigning the corresponding class. We tested two different implementations: a standard one from the sci kit-learn python library (https://scikit-learn.org) [Quinlan, 1986] and our version, where we modified the splitting criterion so as to balances the information gain, namely the decrease of entropy of a random variable (healthy/DCM) observing another random variable (the chosen gene), and the FC computed only among the samples involved in the branch of the tree.

References

MacQueen J.B. Some methods for classification and analysis of multivariate observations. In Proceedings of the 5th Berkeley Symposium on Mathematical Statistics and Probability, volume 1, pages 281–297, 1967.

Quinlan, J.Ross. "Induction of decisiontrees." Machine learning 1.1 (1986): 81-106.

Suppementary Figure legend

Figure S1. Heatmap expression counts of DCM and HS patients after normalization step and batch effect correction.

Figure S2. Piechart reporting the gene aggregations after clustering step.

Figure S3. Heatmap of expression levels for all DCM and HS patients after clustering step.

Figure S4. Boxplot of expression levels for retained genes after clustering filter.

Figure S5. Boxplot of expression levels for selected genes after clustering, considering the fold change threshold of at least 0.5 between HS and DCM samples.

Figure S6. Machine learning (ML) workflow: decision tree algorithm

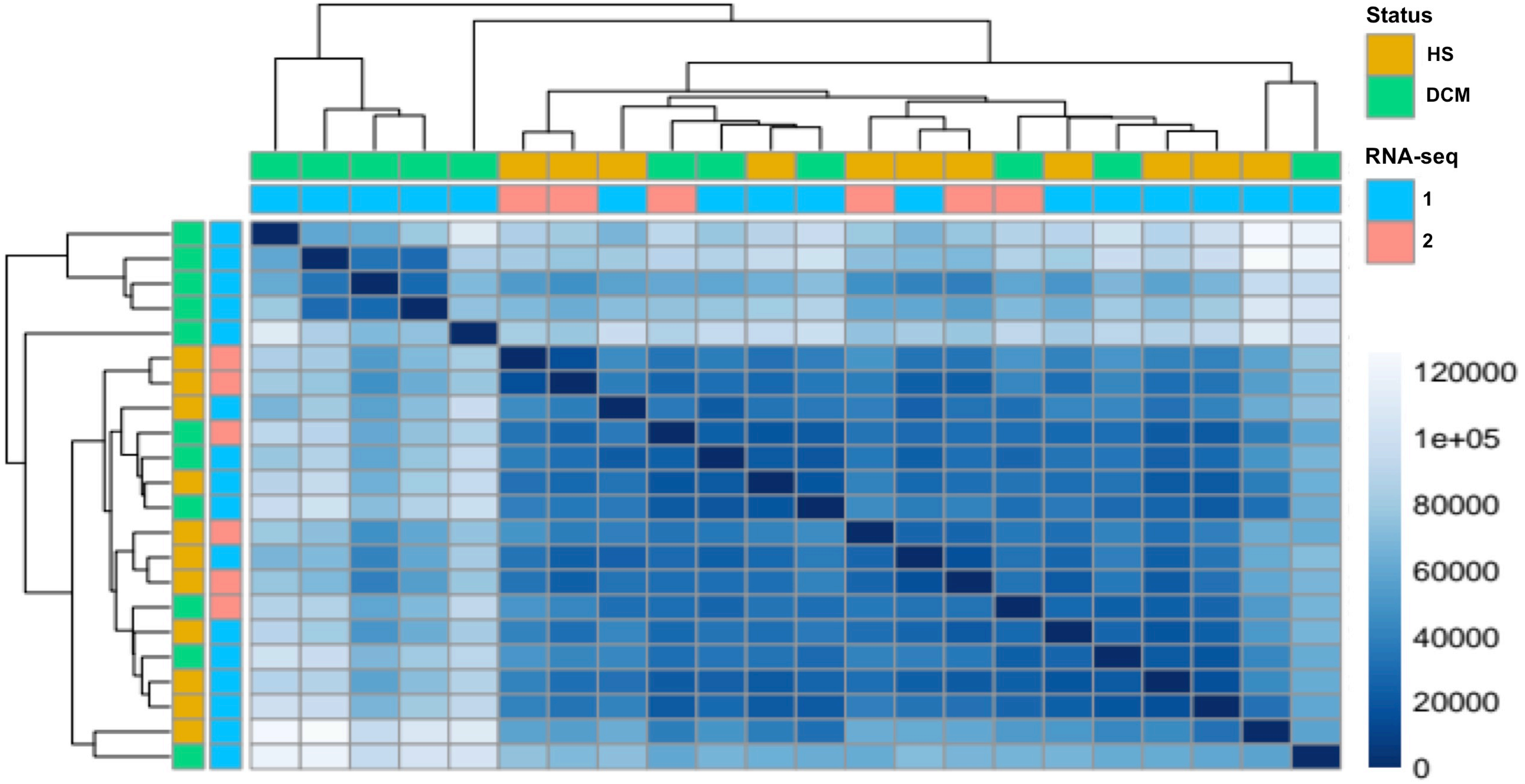

Status
HS
DCM
RNA-seq
1
2
120000
1e+05
80000
60000
40000
20000
0

Figure S1

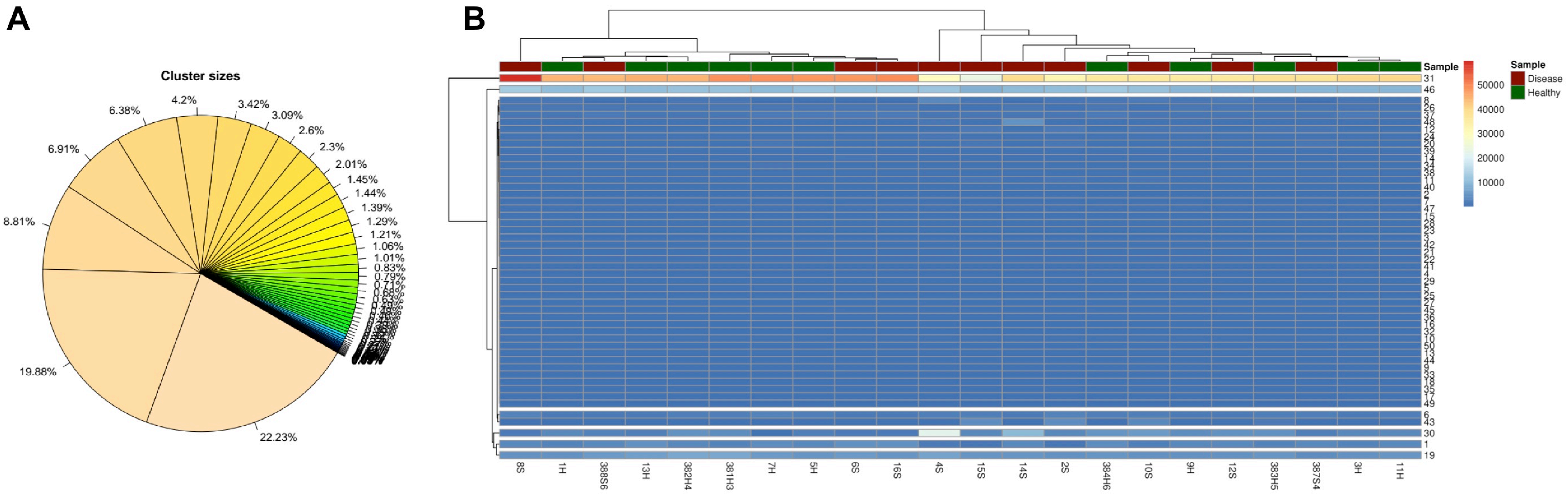
A
Cluster sizes
B
Sample
Disease
Healthy
50000
40000
30000
20000
10000

Figure S2

Figure S3

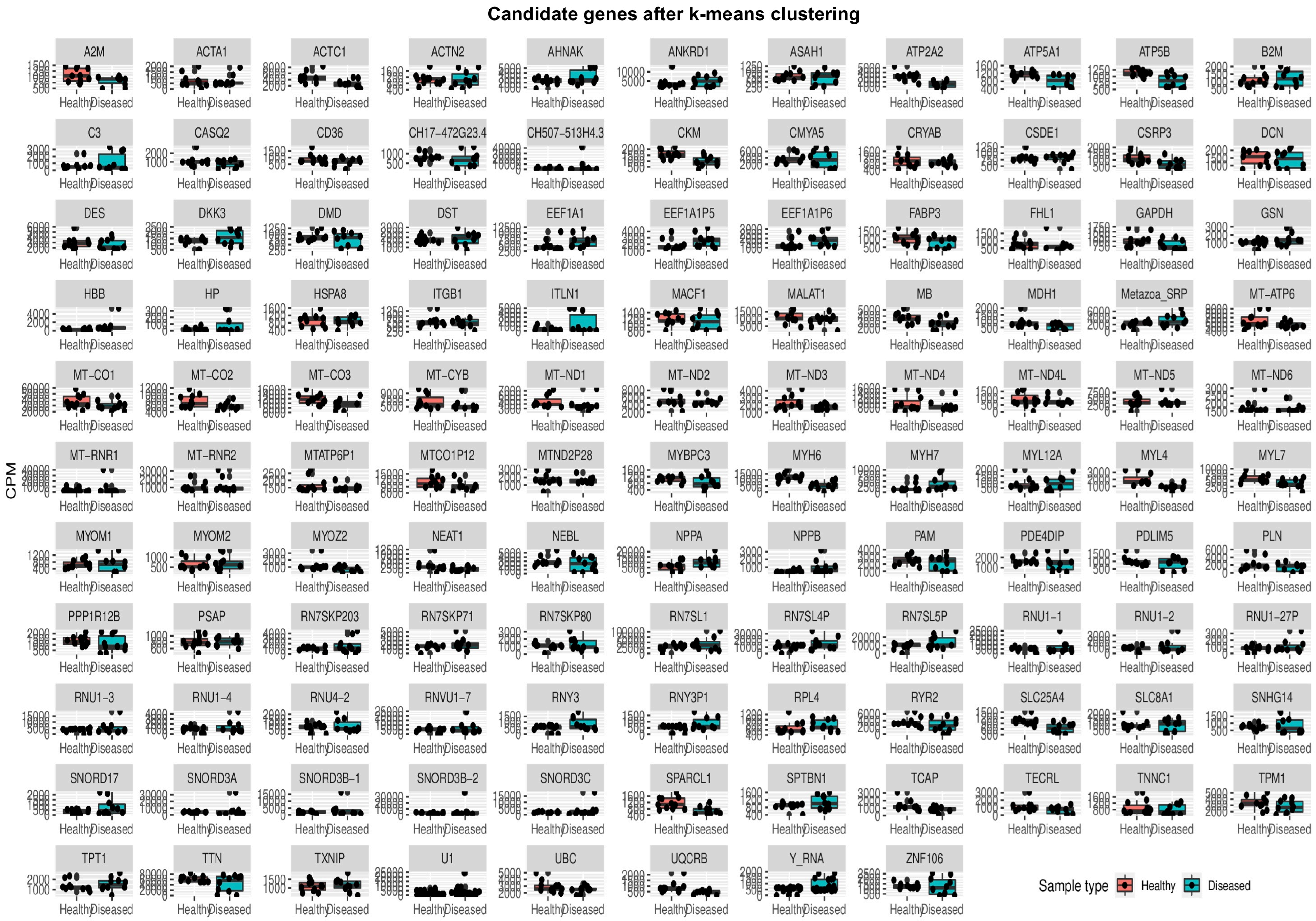
Candidate genes after k-means clustering
CPM
A2M
ACTA1
ACTC1
ACTN2
AHNAK
ANKRD1
ASAH1
ATP2A2
ATP5A1
ATP5B
B2M
C3
CASQ2
CD36
CH17-472G23.4
CH507-513H4.3
CKM
CMYA5
CRYAB
CSDE1
CSRP3
DCN
DES
DKK3
DMD
DST
EEF1A1
EEF1A1P5
EEF1A1P6
FABP3
FHL1
GAPDH
GSN
HBB
HP
HSPA8
ITGB1
ITLN1
MACF1
MALAT1
MB
MDH1
Metazoa_SRP
MT-ATP6
MT-CO1
MT-CO2
MT-CO3
MT-CYB
MT-ND1
MT-ND2
MT-ND3
MT-ND4
MT-ND4L
MT-ND5
MT-ND6
MT-RNR1
MT-RNR2
MTATP6P1
MTCO1P12
MTND2P28
MYBPC3
MYH6
MYH7
MYL12A
MYL4
MYL7
MYOM1
MYOM2
MYOZ2
NEAT1
NEBL
NPPA
NPPB
PAM
PDE4DIP
PDLIM5
PLN
PPP1R12B
PSAP
RN7SKP203
RN7SKP71
RN7SKP80
RN7SL1
RN7SL4P
RN7SL5P
RNU1-1
RNU1-2
RNU1-27P
RNU1-3
RNU1-4
RNU4-2
RNVU1-7
RNY3
RNY3P1
RPL4
RYR2
SLC25A4
SLC8A1
SNHG14
SNORD17
SNORD3A
SNORD3B-1
SNORD3B-2
SNORD3C
SPARCL1
SPTBN1
TCAP
TECRL
TNNC1
TPM1
TPT1
TTN
TXNIP
U1
UBC
UQCRB
Y_RNA
ZNF106
Healthy
Diseased
Sample type
Healthy
Diseased

Figure S4

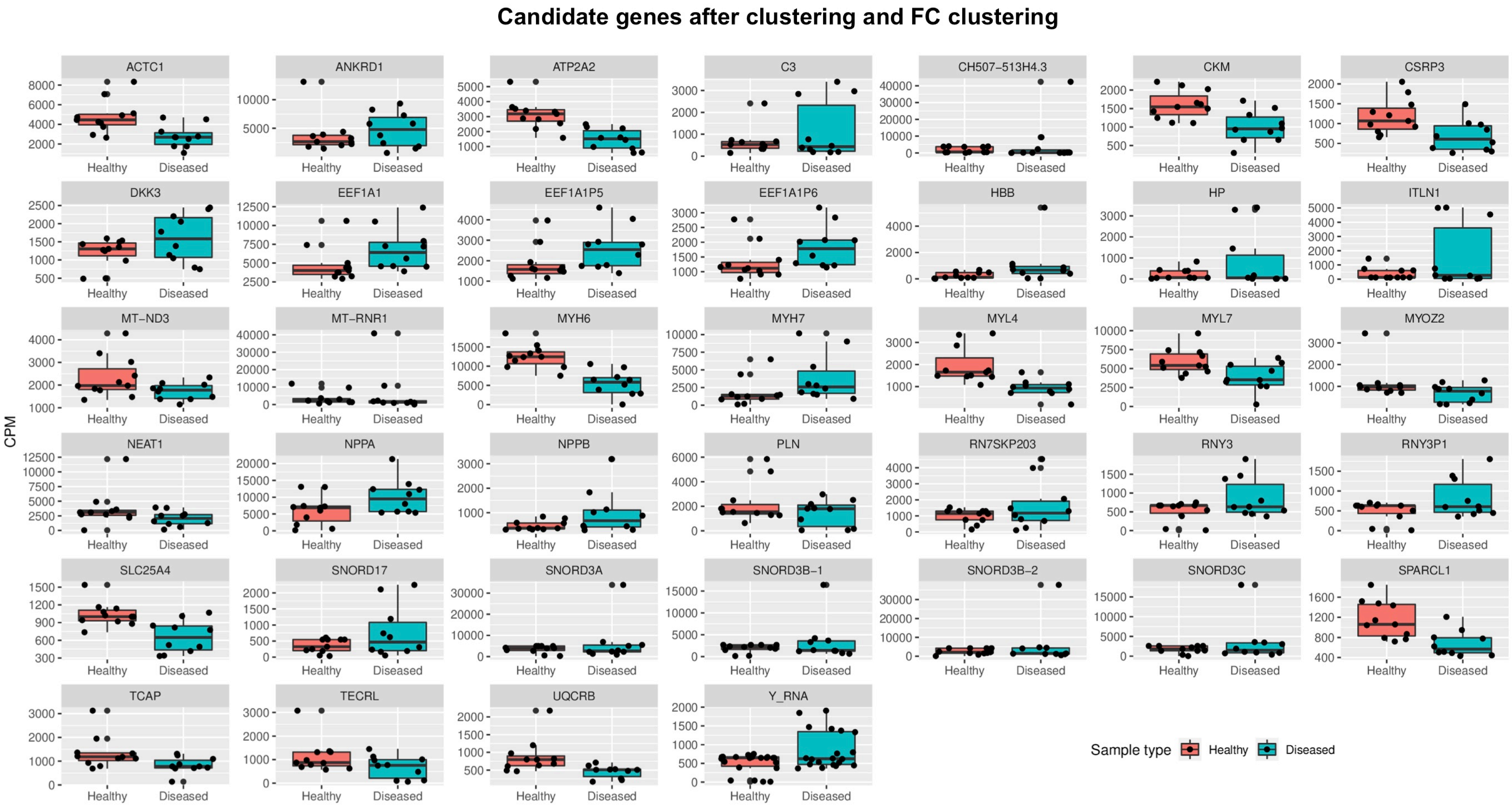
Candidate genes after clustering and FC clustering
CPM
ACTC1
ANKRD1
ATP2A2
C3
CH507−513H4.3
CKM
CSRP3
DKK3
EEF1A1
EEF1A1P5
EEF1A1P6
HBB
HP
ITLN1
MT−ND3
MT−RNR1
MYH6
MYH7
MYL4
MYL7
MYOZ2
NEAT1
NPPA
NPPB
PLN
RN7SKP203
RNY3
RNY3P1
SLC25A4
SNORD17
SNORD3A
SNORD3B−1
SNORD3B−2
SNORD3C
SPARCL1
TCAP
TECRL
UQCRB
Y_RNA
Healthy
Diseased
Sample type
Healthy
Diseased

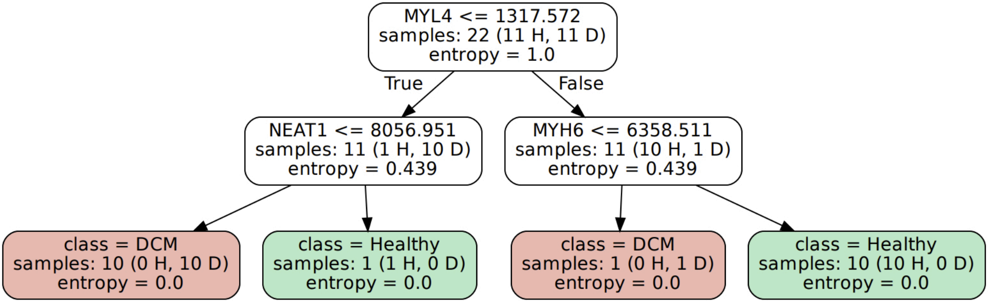
MYL4 <= 1317.572
samples: 22 (11 H, 11 D)
entropy = 1.0
True
False
NEAT1 <= 8056.951
samples: 11 (1 H, 10 D)
entropy = 0.439
MYH6 <= 6358.511
samples: 11 (10 H, 1 D)
entropy = 0.439
class = DCM
samples: 10 (0 H, 10 D)
entropy = 0.0
class = Healthy
samples: 1 (1 H, 0 D)
entropy = 0.0
class = DCM
samples: 1 (0 H, 1 D)
entropy = 0.0
class = Healthy
samples: 10 (10 H, 0 D)
entropy = 0.0

Table S1

Gene biotype	Gene name	logFC	P Value	FDR
	**Up-regulated**			
Unprocessed pseudogene	AC138969.2	0,499	0,048	0,380
	AC138866.2	0,478	0,020	0,274
	HLA-H	0,424	0,098	0,489
	AC131392.1	0,409	0,041	0,360
	AC139272.1	0,406	0,024	0,292
	AC138866.1	0,330	0,099	0,489
	SRGAP2D	0,262	0,296	0,706
	TCAF1P1	0,217	0,344	0,739
	AC011330.1	0,211	0,242	0,659
	AC140134.1	0,184	0,323	0,724
	AC145138.1	0,149	0,390	0,768
	CR381670.1	0,100	0,669	0,907
	AC131392.2	0,093	0,788	0,948
	MTND2P28	0,084	0,800	0,950
	WASH7P	0,060	0,769	0,942
	AC083899.1	0,048	0,783	0,946
	WASH9P	0,047	0,811	0,954
	TNXA	0,044	0,889	0,978
	**Down-regulated**			
	AC233968.1	-0,013	0,951	0,993
	MTATP6P1	-0,018	0,938	0,992
	NSFP1	-0,029	0,884	0,977
	MTND1P23	-0,050	0,854	0,970
	SMG1P6	-0,063	0,784	0,946
	MTCO1P12	-0,136	0,579	0,870
	DPY19L1P1	-0,137	0,648	0,899
	AC233699.1	-0,169	0,474	0,817
	MTCO3P12	-0,172	0,429	0,792
	MTCO2P12	-0,277	0,298	0,709
	SHLD2P3	-0,311	0,110	0,504
	CBWD4P	-0,323	0,172	0,589
	AC244669.2	-0,374	0,165	0,580
	AQP7P3	-0,825	0,021	0,281
	CNTNAP3P2	-0,983	0,027	0,303
	**Up-regulated**			
	HYDIN2	0,757	0,005	0,153
	GVINP1	0,363	0,149	0,559
	GUSBP2	0,344	0,118	0,518
	LRRC37A4P	0,317	0,328	0,727
	CES1P1	0,293	0,371	0,756
	NBPF25P	0,276	0,100	0,489
	AC127502.1	0,264	0,394	0,771
	ROCK1P1	0,263	0,171	0,588
	GUSBP3	0,256	0,167	0,585
	NBPF8	0,240	0,213	0,634
	GTF2IP1	0,221	0,145	0,555
	GOLGA2P7	0,201	0,296	0,706
	USP32P3	0,201	0,552	0,856
	SBDSP1	0,177	0,238	0,656

	SEC22B4P	0,171	0,298	0,709
	GUSBP1	0,164	0,315	0,719
	GOLGA6L5P	0,161	0,399	0,774
	AC084357.3	0,152	0,445	0,799
	AC138409.2	0,130	0,453	0,806
	FKBP9P1	0,125	0,478	0,819
	SLC35E2A	0,124	0,516	0,838
	EP400P1	0,115	0,633	0,892
	AC092070.2	0,114	0,494	0,826
	CSPG4P12	0,112	0,618	0,888
	WASH2P	0,110	0,563	0,862
	BMS1P7	0,104	0,612	0,887
	RRN3P1	0,085	0,593	0,879
	CEP170P1	0,064	0,647	0,899
	RPL23AP82	0,044	0,792	0,948
	NSUN5P1	0,041	0,846	0,967
	GTF2IRD2P1	0,039	0,953	0,994
	WHAMMP3	0,031	0,837	0,964
	STAG3L1	0,025	0,915	0,984
	AC009053.1	0,025	0,915	0,984
	FRG1CP	0,022	0,897	0,979
	LINC01145	0,021	0,904	0,981
	UBE2Q2P1	0,002	0,989	1,000
	AMZ2P1	0,000	0,999	1,000
	**Down-regulated**			
	TXLNGY	-0,004	0,993	1,000
	GTF2H2B	-0,012	0,953	0,994
	STAG3L3	-0,013	0,956	0,994
	AC026273.1	-0,013	0,992	1,000
	CCT6P3	-0,025	0,883	0,977
	MFSD14C	-0,031	0,870	0,974
	AL353743.1	-0,036	0,857	0,970
**Transcribed unprocessed pseudogene**	WASH3P	-0,040	0,826	0,959
	ZNF542P	-0,050	0,778	0,945
	AL669831.1	-0,055	0,818	0,955
	AC006453.2	-0,056	0,808	0,953
	UBBP4	-0,062	0,715	0,925
	BMS1P1	-0,063	0,696	0,917
	SEPTIN7P2	-0,063	0,686	0,913
	AC244154.1	-0,073	0,713	0,924
	CCDC144B	-0,075	0,811	0,954
	AC138393.1	-0,076	0,681	0,911
	LSP1P4	-0,077	0,733	0,931
	AC093752.1	-0,117	0,434	0,795
	AC006001.3	-0,120	0,535	0,850
	WHAMMP2	-0,121	0,507	0,833
	AL390728.4	-0,138	0,483	0,821
	SMG1P7	-0,138	0,503	0,832
	AC241952.1	-0,143	0,625	0,889
	FAM157A	-0,144	0,581	0,871
	PARGP1	-0,157	0,462	0,811
	SDHAP1	-0,157	0,426	0,791
	STAG3L2	-0,158	0,453	0,806

	FRG1HP	-0,159	0,355	0,746
	SDHAP3	-0,174	0,310	0,715
	SUZ12P1	-0,187	0,325	0,724
	SMG1P5	-0,191	0,380	0,761
	SMG1P2	-0,195	0,340	0,736
	ZNF767P	-0,203	0,264	0,678
	SVIL-AS1	-0,205	0,134	0,540
	SMG1P1	-0,211	0,388	0,767
	DPY19L2P2	-0,216	0,475	0,818
	SMG1P3	-0,219	0,332	0,730
	DPY19L2P1	-0,223	0,596	0,880
	AC106795.1	-0,225	0,244	0,661
	AL732372.2	-0,226	0,483	0,821
	SMG1P4	-0,231	0,320	0,721
	STAG3L4	-0,303	0,095	0,484
	ZNF658B	-0,312	0,171	0,588
	PDCD6IPP1	-0,324	0,066	0,431
	AC132008.2	-0,329	0,171	0,588
	SHLD2P1	-0,342	0,081	0,464
	PGM5P2	-0,359	0,179	0,600
	LRRC37A16P	-0,370	0,056	0,402
	DPY19L2P4	-0,408	0,197	0,621
	HERC2P9	-0,418	0,116	0,514
	AC245297.1	-0,451	0,137	0,545
	ZNF252P	-0,454	0,002	0,110
	HERC2P3	-0,532	0,074	0,450
	HERC2P2	-0,569	0,044	0,369
	PDCD6IPP2	-0,597	0,001	0,064
	BMS1P10	-0,657	0,021	0,281
**Transcribed unitary pseudogene**	**Up-regulated**			
	CUTALP	0,714	0,020	0,276
	C22orf46	0,339	0,106	0,499
	AHSA2P	0,167	0,291	0,702
	CMAHP	0,085	0,715	0,925
	TP73-AS1	0,056	0,817	0,955
	ZNRD1ASP	0,045	0,833	0,963
	**Down-regulated**			
	PI4KAP2	-0,014	0,945	0,993
	CRYZL2P	-0,170	0,376	0,758
	LINC02210	-0,236	0,272	0,685
	ZNF271P	-0,260	0,134	0,540
	AFG3L1P	-0,301	0,104	0,495
**Transcribed processed**	**Up-regulated**			
	GTF2IP4	0,248	0,114	0,512
	RPS27AP16	0,220	0,364	0,753
	RPL13AP7	0,217	0,333	0,730
	AC243919.1	0,212	0,378	0,761
	CCDC144CP	0,154	0,672	0,908
	ZNF37BP	0,151	0,468	0,813
	AC135983.2	0,105	0,713	0,924
	RPL6P27	0,065	0,788	0,948
	PDIA3P1	0,047	0,743	0,934
	AC073869.1	0,037	0,860	0,971

**pseudogene**	CENPBD1P1	0,017	0,980	0,998
	PTENP1	0,003	0,986	0,999
	**Down-regulated**			
	AP006222.1	-0,035	0,855	0,970
	HMGB1P5	-0,060	0,773	0,944
	AC004057.1	-0,104	0,758	0,938
	FTH1P10	-0,150	0,551	0,856
	TMEM14EP	-0,161	0,387	0,766
	AC020904.2	-0,205	0,358	0,747
	WASH5P	-0,372	0,203	0,625
	NPY6R	-0,379	0,247	0,661
**TR_C_gene**	**Down-regulated**			
	TRAC	-1,388	0,000	0,000
**TEC**	**Up-regulated**			
	AP001528.2	0,587	0,038	0,346
	AC093908.1	0,112	0,745	0,935
	AC027290.2	0,093	0,802	0,951
	**Down-regulated**			
	AC000123.3	-0,031	0,871	0,974
	Z99129.4	-0,089	0,691	0,915
	AC131649.2	-0,108	0,693	0,916
	AL662795.2	-0,154	0,412	0,782
	AC009948.4	-0,160	0,647	0,899
	AP000648.4	-0,203	0,415	0,784
	AL136164.3	-0,222	0,343	0,738
	AC009951.4	-0,256	0,309	0,715
	AL353763.2	-0,452	0,114	0,512
	AC093909.6	-0,542	0,004	0,135
**snRNA**	**Up-regulated**			
	RNU5A-1	1,383	0,067	0,434
	RNU2-64P	0,877	0,147	0,556
	RNU1-3	0,761	0,238	0,656
	U1	0,723	0,248	0,661
	RNVU1-7	0,719	0,250	0,663
	RNU1-1	0,712	0,253	0,666
	U1	0,657	0,269	0,682
	RNU1-2	0,651	0,276	0,689
	RNU1-27P	0,628	0,290	0,701
	U1	0,625	0,287	0,700
	RNU1-4	0,624	0,290	0,701
	RNU5B-1	0,589	0,250	0,663
	RNU6-1	0,576	0,108	0,503
	RNU1-28P	0,555	0,357	0,747
	RNU6-5P	0,530	0,137	0,545
	RNU6-6P	0,504	0,157	0,569
	RNVU1-18	0,502	0,397	0,774
	RNU6-33P	0,494	0,153	0,565
	RNU6-3P	0,480	0,157	0,569
	RNU4-2	0,448	0,444	0,798
	RNU6-2	0,437	0,213	0,634
	RNU6-7	0,424	0,203	0,625
	RNU6-4P	0,415	0,226	0,646
	RNU6-9	0,415	0,197	0,621

	RNU6-36P	0,409	0,241	0,659
	**Down-regulated**			
	RNU4-1	-0,081	0,863	0,973
**snoRNA**	Up-regulated			
	SNORA74B	2,980	0,033	0,331
	SNORD3B-2	1,867	0,060	0,416
	SNORD17	1,472	0,054	0,394
	SNORD3C	1,389	0,099	0,489
	SNORD3A	1,215	0,118	0,517
	SNORD3B-1	1,175	0,118	0,517
	SNORA74A	1,135	0,072	0,447
	SNORA53	1,033	0,122	0,525
	SNORD15B	0,849	0,104	0,495
	SNORA23	0,688	0,167	0,585
	SNORA73B	0,634	0,123	0,525
	SNORD94	0,608	0,266	0,679
	SNORA12	0,463	0,279	0,690
	SNORA54	0,291	0,460	0,810
	SNORA79B	0,093	0,755	0,937
	SNORA49	0,002	0,994	1,000
**scRNA**	**Down-regulated**			
	BCYRN1	-0,214	0,652	0,899
**scaRNA**	**Up-regulated**			
	SCARNA5	0,888	0,041	0,362
	SCARNA6	0,636	0,046	0,377
	SCARNA21	0,589	0,115	0,513
	SCARNA7	0,006	0,986	0,999
**rRNA pseudogene**	**Up-regulated**			
	RNA5SP202	0,696	0,163	0,578
	RNA5SP226	0,632	0,255	0,668
	RNA5SP389	0,613	0,267	0,680
	RNA5SP370	0,611	0,274	0,687
	RNA5SP145	0,425	0,399	0,774
	RNA5SP429	0,088	0,836	0,963
	**Up-regulated**			
	HP	3,248	0,033	0,331
	SULF1	3,147	0,009	0,192
	HPR	3,129	0,015	0,242
	ITLN1	3,050	0,010	0,206
	HBB	2,946	0,005	0,150
	ID4	2,946	0,000	0,009
	PKHD1L1	2,720	0,039	0,351
	KCNT2	2,563	0,007	0,171
	NPR3	2,476	0,000	0,009
	CP	2,358	0,000	0,003
	CDH11	2,340	0,003	0,128
	FRAS1	2,220	0,003	0,115
	HBA1	2,104	0,001	0,073
	HBA2	2,088	0,001	0,064
	PTGS1	1,983	0,001	0,085
	INMT	1,926	0,004	0,138
	FAM107B	1,818	0,008	0,180

POSTN	1,800	0,006	0,167
TBX18	1,794	0,002	0,100
OMD	1,744	0,002	0,091
EFEMP1	1,680	0,003	0,113
ALDH1A2	1,664	0,000	0,029
SMYD2	1,635	0,000	0,009
CFH	1,634	0,001	0,061
NR4A2	1,612	0,004	0,143
SLC39A8	1,576	0,008	0,190
ASPN	1,564	0,001	0,080
FLRT2	1,550	0,013	0,232
FMOD	1,490	0,000	0,020
HLA-DQA1	1,474	0,000	0,039
ITGB8	1,469	0,065	0,431
DGKI	1,464	0,000	0,049
TNC	1,445	0,001	0,064
SLC41A2	1,444	0,014	0,235
OGN	1,415	0,022	0,285
SLC7A2	1,375	0,013	0,232
SERPINE1	1,354	0,002	0,091
UCHL1	1,345	0,000	0,049
MYH10	1,341	0,000	0,047
SIK1	1,340	0,002	0,094
ECM2	1,318	0,002	0,111
MXRA5	1,308	0,010	0,203
SIK1B	1,297	0,002	0,111
ZNF385B	1,297	0,002	0,094
LEPR	1,275	0,007	0,177
FOS	1,243	0,035	0,336
IGFBP3	1,243	0,005	0,147
TEF	1,234	0,000	0,039
DUSP1	1,233	0,007	0,174
ZNF331	1,233	0,013	0,228
PLIN1	1,231	0,159	0,574
DAPK1	1,220	0,025	0,295
GRIN2A	1,220	0,009	0,196
CD200	1,203	0,056	0,403
HLA-DPB1	1,202	0,000	0,032
PTPRF	1,181	0,097	0,485
MYH7	1,181	0,060	0,415
EPHA3	1,173	0,001	0,087
F2R	1,170	0,000	0,020
HSPA2	1,165	0,003	0,123
COL3A1	1,159	0,025	0,295
CDON	1,151	0,042	0,362
HLA-DPA1	1,143	0,001	0,078
CCN2	1,134	0,027	0,303
PLEKHA6	1,133	0,001	0,073
COL8A1	1,126	0,000	0,029
NOX4	1,113	0,002	0,094
TNFAIP3	1,112	0,023	0,285
CSRNP1	1,111	0,006	0,162
HES1	1,097	0,001	0,090

SLC6A6	1,090	0,013	0,228
PDGFD	1,077	0,003	0,124
CRIM1	1,077	0,017	0,251
LBH	1,066	0,000	0,029
PLXDC2	1,063	0,004	0,135
SMAD6	1,049	0,003	0,113
ID2	1,049	0,007	0,176
CGNL1	1,040	0,024	0,295
FN1	1,039	0,002	0,112
GAS1	1,030	0,015	0,235
PER2	1,025	0,000	0,047
BGN	1,023	0,007	0,177
ADGRG6	1,021	0,032	0,326
NR4A3	1,021	0,302	0,711
PTPRQ	1,013	0,084	0,472
PROS1	1,009	0,000	0,002
KDR	0,994	0,072	0,446
LRP5	0,986	0,002	0,110
TIMP1	0,983	0,066	0,431
SLIT3	0,981	0,012	0,225
TM4SF1	0,979	0,090	0,480
MTURN	0,973	0,000	0,028
LUM	0,972	0,004	0,133
DUSP6	0,971	0,001	0,076
FHL2	0,966	0,014	0,235
FZD7	0,964	0,000	0,002
SMAD7	0,960	0,001	0,081
SPP1	0,954	0,044	0,370
C4B	0,952	0,113	0,511
SMAD9	0,949	0,009	0,196
SESN3	0,949	0,038	0,346
GCNT2	0,949	0,001	0,087
NPPB	0,943	0,028	0,308
NR4A1	0,941	0,054	0,394
C3	0,934	0,146	0,556
ADM	0,932	0,002	0,112
PHGDH	0,929	0,027	0,308
DPT	0,928	0,002	0,096
ROBO1	0,925	0,002	0,100
FMO2	0,923	0,013	0,228
ZFHX4	0,923	0,016	0,244
PHLPP2	0,923	0,025	0,295
ZNF676	0,919	0,003	0,113
LTBP2	0,913	0,003	0,128
COL14A1	0,903	0,013	0,228
GPD1	0,898	0,269	0,682
SLC4A4	0,898	0,067	0,434
PTGIS	0,891	0,097	0,485
PRKCA	0,885	0,008	0,187
C4A	0,881	0,125	0,527
PTPN13	0,880	0,032	0,327
COLQ	0,879	0,015	0,235
ATF3	0,877	0,205	0,625

EGR1	0,873	0,109	0,503
SPRY4	0,872	0,009	0,192
MICAL2	0,871	0,000	0,034
MYO1D	0,867	0,002	0,091
PHLDA1	0,867	0,007	0,172
VAT1L	0,865	0,003	0,116
PRSS23	0,864	0,007	0,174
ROR1	0,863	0,000	0,020
SPOCK2	0,861	0,014	0,235
SELP	0,860	0,011	0,215
CXADR	0,857	0,040	0,356
ABAT	0,855	0,009	0,193
NFASC	0,853	0,017	0,251
COL21A1	0,849	0,001	0,087
SLC20A1	0,849	0,052	0,388
ADCY6	0,846	0,010	0,207
PLIN4	0,844	0,111	0,506
PER3	0,842	0,002	0,094
HLA-DRB1	0,834	0,004	0,141
CXCL2	0,832	0,164	0,579
LIPE	0,824	0,138	0,546
TMEM98	0,819	0,003	0,116
LOX	0,818	0,047	0,379
TLL1	0,816	0,100	0,489
MXRA8	0,808	0,004	0,143
SKIL	0,804	0,035	0,336
IFI6	0,799	0,002	0,106
NCKIPSD	0,797	0,020	0,278
PTPN14	0,791	0,014	0,233
MYO5B	0,789	0,016	0,244
ARAP2	0,786	0,068	0,437
ZNF208	0,786	0,010	0,207
CYP27A1	0,784	0,001	0,083
UBA7	0,776	0,002	0,107
PNMA8A	0,776	0,003	0,128
ARFGEF3	0,775	0,062	0,422
MATN2	0,774	0,057	0,405
CD9	0,769	0,001	0,087
PDE1A	0,765	0,013	0,232
TENM2	0,761	0,075	0,452
COBLL1	0,759	0,092	0,482
PLXNA4	0,757	0,093	0,484
HLA-DRA	0,757	0,023	0,285
BTN3A1	0,756	0,003	0,123
CHRDL1	0,754	0,100	0,489
PDGFC	0,753	0,013	0,231
PIK3IP1	0,749	0,006	0,166
TSC22D3	0,748	0,086	0,473
ANTXR1	0,745	0,006	0,171
CRYM	0,744	0,076	0,453
SPARC	0,739	0,018	0,263
PER1	0,738	0,068	0,437
PLSCR4	0,733	0,027	0,305

MASP1	0,731	0,050	0,387
ANKRD50	0,730	0,015	0,239
GBP3	0,730	0,035	0,337
GATM	0,730	0,007	0,171
ITGA8	0,729	0,154	0,566
ISLR	0,727	0,035	0,336
PKD1	0,726	0,033	0,331
COL16A1	0,725	0,008	0,186
CCND1	0,724	0,001	0,087
SOX9	0,724	0,022	0,284
BHLHE41	0,722	0,000	0,019
HLA-DRB5	0,721	0,013	0,231
CD74	0,720	0,052	0,389
MEST	0,719	0,234	0,655
PROCR	0,718	0,171	0,588
DOK5	0,717	0,007	0,173
BTN3A2	0,714	0,014	0,235
FMNL3	0,713	0,005	0,148
SERTAD4	0,711	0,004	0,135
PAPSS2	0,709	0,035	0,336
ITGB5	0,708	0,002	0,096
SEMA3C	0,707	0,115	0,513
SDC3	0,705	0,035	0,336
C2CD2	0,700	0,091	0,481
TRANK1	0,698	0,009	0,197
HSPG2	0,695	0,095	0,484
CCNYL1	0,695	0,046	0,377
IGFBP7	0,693	0,005	0,147
ARHGAP18	0,689	0,158	0,571
NPPA	0,687	0,180	0,600
NEO1	0,687	0,061	0,416
AQP9	0,686	0,169	0,587
NAP1L3	0,685	0,020	0,274
PRICKLE1	0,685	0,000	0,046
DNAJB1	0,678	0,000	0,055
ANOS1	0,677	0,034	0,334
CARMIL1	0,675	0,013	0,232
IGFBP2	0,674	0,056	0,403
NEB	0,674	0,252	0,666
HTRA1	0,674	0,001	0,061
ATP9A	0,674	0,028	0,309
TMEM71	0,673	0,062	0,418
KDELR3	0,671	0,001	0,073
THBS2	0,670	0,102	0,492
RAB31	0,670	0,025	0,295
MMD	0,669	0,095	0,484
BIRC3	0,669	0,204	0,625
PRKD1	0,668	0,057	0,407
RAB11FIP1	0,667	0,001	0,081
GLT8D2	0,666	0,014	0,234
TBC1D2B	0,664	0,050	0,383
EVC	0,662	0,031	0,322
OAS3	0,660	0,024	0,291

VASH1	0,660	0,011	0,210
CPXM2	0,659	0,026	0,300
ATP2B1	0,658	0,041	0,361
TM7SF2	0,658	0,013	0,232
MET	0,650	0,145	0,555
FAM102B	0,649	0,034	0,332
ANGPTL2	0,649	0,019	0,271
B4GALNT3	0,649	0,026	0,301
IFIT2	0,648	0,028	0,311
SGK1	0,644	0,147	0,556
CACHD1	0,639	0,112	0,507
PLA2R1	0,639	0,038	0,349
AEBP1	0,639	0,024	0,291
ARHGAP1	0,639	0,018	0,257
GEM	0,638	0,098	0,488
DDR1	0,634	0,006	0,160
MGP	0,632	0,029	0,316
XPC	0,629	0,001	0,061
PLEKHH2	0,629	0,042	0,364
ATOH8	0,627	0,088	0,476
RGS2	0,627	0,115	0,514
C10orf71	0,626	0,123	0,525
PLXNB2	0,625	0,005	0,152
ATP7B	0,625	0,002	0,094
LRIG3	0,623	0,020	0,278
RARRES2	0,623	0,105	0,496
KLF4	0,620	0,054	0,394
VIM	0,618	0,065	0,429
SPON1	0,618	0,075	0,452
CHGB	0,617	0,208	0,629
FGF9	0,615	0,010	0,207
VGLL3	0,615	0,194	0,618
STAT4	0,612	0,175	0,595
GPX8	0,611	0,006	0,161
NFIX	0,608	0,051	0,387
ADIPOQ	0,607	0,457	0,808
ETV5	0,607	0,003	0,123
COLGALT2	0,606	0,038	0,348
UNC5B	0,604	0,025	0,295
LMO3	0,603	0,158	0,571
HMCN1	0,602	0,101	0,491
C7	0,601	0,070	0,441
REEP1	0,600	0,041	0,361
MAF	0,599	0,040	0,356
ZNF704	0,599	0,005	0,153
COL1A2	0,598	0,121	0,521
NYNRIN	0,597	0,028	0,309
HNMT	0,595	0,113	0,511
PLCE1	0,594	0,017	0,253
GRIP2	0,592	0,094	0,484
USP11	0,591	0,005	0,157
NLRP1	0,591	0,033	0,330
AOC3	0,589	0,236	0,656

Protein coding	COL15A1	0,588	0,072	0,446
	DOCK10	0,588	0,059	0,414
	EFNB2	0,588	0,052	0,388
	ERN1	0,587	0,029	0,315
	ATP1B4	0,585	0,246	0,661
	HMGN2	0,584	0,002	0,106
	FGL2	0,583	0,078	0,459
	CPED1	0,580	0,017	0,251
	HBEGF	0,579	0,078	0,459
	ODC1	0,579	0,007	0,177
	MORC4	0,577	0,019	0,269
	AZGP1	0,576	0,071	0,443
	AMOT	0,574	0,045	0,372
	CCDC80	0,573	0,170	0,588
	THBS3	0,573	0,035	0,336
	JAML	0,572	0,114	0,512
	ADAMTS2	0,572	0,107	0,501
	MMP16	0,570	0,079	0,460
	CSF1R	0,569	0,083	0,468
	RPS4Y1	0,569	0,422	0,789
	ENG	0,568	0,081	0,464
	RAB34	0,568	0,027	0,303
	OLFML1	0,568	0,047	0,379
	SLC16A2	0,565	0,054	0,395
	BCL2L11	0,565	0,004	0,138
	SERPINB9	0,563	0,072	0,447
	ELMSAN1	0,561	0,046	0,378
	LDB1	0,561	0,042	0,362
	CYBRD1	0,559	0,087	0,475
	FAXDC2	0,558	0,011	0,210
	BCL7A	0,557	0,013	0,232
	LRATD2	0,556	0,051	0,388
	MRC2	0,556	0,067	0,434
	BTN3A3	0,553	0,045	0,374
	SNX33	0,550	0,026	0,300
	GAB2	0,549	0,009	0,193
	FKBP10	0,548	0,038	0,347
	MTRNR2L12	0,547	0,236	0,655
	APOB	0,546	0,234	0,655
	TMCO3	0,545	0,000	0,013
	AMOTL2	0,545	0,079	0,460
	SETBP1	0,545	0,102	0,493
	LTBP3	0,545	0,019	0,271
	REL	0,543	0,175	0,595
	SERPINE2	0,543	0,081	0,464
	SLC16A12	0,542	0,008	0,190
	MX1	0,541	0,021	0,283
	PTPRD	0,539	0,203	0,625
	SLIT2	0,538	0,051	0,388
	GADD45A	0,537	0,012	0,222
	DKK3	0,537	0,064	0,426
	NFKBIZ	0,536	0,232	0,653
	EXT1	0,534	0,001	0,087

ULK2	0,534	0,000	0,039
ARRDC4	0,531	0,047	0,380
ALPK3	0,530	0,096	0,484
TMSB4X	0,528	0,091	0,481
CHPF	0,528	0,034	0,332
NFKB1	0,522	0,094	0,484
XPR1	0,522	0,096	0,484
SERP1	0,521	0,097	0,486
STK17B	0,518	0,158	0,571
LAMA4	0,518	0,056	0,402
MPP6	0,517	0,191	0,616
COPZ2	0,516	0,001	0,064
GLS	0,516	0,074	0,450
IL16	0,514	0,072	0,447
TRIM4	0,513	0,015	0,241
ORMDL3	0,513	0,010	0,207
COL5A2	0,512	0,080	0,463
PPP1R10	0,512	0,042	0,362
GXYLT2	0,512	0,024	0,291
COL1A1	0,510	0,199	0,623
PRDM5	0,510	0,023	0,290
DCHS1	0,509	0,187	0,611
NRP2	0,509	0,049	0,380
TMEM140	0,508	0,019	0,268
CDR2	0,508	0,003	0,123
EEF2	0,507	0,021	0,284
PATZ1	0,506	0,020	0,278
LTBP4	0,505	0,025	0,295
KLF6	0,504	0,151	0,562
SDC4	0,503	0,052	0,389
ZBTB47	0,502	0,023	0,285
GOLM1	0,502	0,037	0,344
VWF	0,501	0,084	0,472
KLHL3	0,500	0,042	0,365
RAB23	0,498	0,036	0,341
IGSF10	0,497	0,231	0,652
AHNAK	0,496	0,110	0,505
ID3	0,494	0,008	0,186
ZNF395	0,494	0,035	0,336
PRKY	0,494	0,381	0,761
SEPTIN11	0,493	0,060	0,415
SLC30A1	0,493	0,064	0,426
HACD2	0,493	0,173	0,592
SIPA1L1	0,492	0,030	0,318
AC240274.1	0,492	0,083	0,468
NFKBIA	0,491	0,095	0,484
SNTB2	0,491	0,110	0,504
MXD4	0,491	0,031	0,322
RAB11FIP3	0,491	0,014	0,235
TENM3	0,490	0,049	0,381
RAI14	0,489	0,056	0,403
F3	0,489	0,022	0,285
SVEP1	0,489	0,176	0,595

KIAA0556	0,488	0,014	0,233
ZC3HAV1	0,488	0,030	0,319
RNF152	0,486	0,064	0,425
IFT52	0,486	0,029	0,312
NT5E	0,486	0,031	0,320
CUL7	0,485	0,023	0,287
HLF	0,484	0,008	0,183
MAP4	0,483	0,055	0,401
ERRFI1	0,483	0,335	0,732
TBC1D9	0,483	0,030	0,319
ANO1	0,482	0,052	0,390
FIBIN	0,482	0,021	0,280
APOE	0,480	0,186	0,610
APAF1	0,480	0,021	0,283
FUT10	0,480	0,037	0,345
MYPN	0,480	0,156	0,568
FPR3	0,479	0,129	0,533
GNG12	0,478	0,086	0,474
PRKCI	0,478	0,074	0,450
TTC39B	0,477	0,043	0,369
VPS8	0,477	0,066	0,431
PPP1R16B	0,476	0,025	0,295
CTSO	0,474	0,011	0,214
KCNC4	0,474	0,115	0,514
NCOA7	0,474	0,094	0,484
RGPD2	0,474	0,222	0,644
LENG8	0,473	0,099	0,489
STAT1	0,473	0,026	0,298
VGLL4	0,472	0,018	0,255
PPP1R15B	0,470	0,103	0,495
ALDH18A1	0,470	0,008	0,190
TTC39C	0,469	0,096	0,484
BICD1	0,469	0,012	0,218
CTSK	0,468	0,102	0,493
DCAF12	0,468	0,042	0,362
MAN1C1	0,468	0,055	0,401
BACE1	0,468	0,013	0,232
TMEM8B	0,468	0,054	0,395
KIF3B	0,467	0,012	0,225
MR1	0,467	0,006	0,164
KIF13A	0,467	0,002	0,107
DDX60	0,466	0,037	0,345
ARHGEF26	0,466	0,181	0,601
ENAH	0,465	0,002	0,110
RANBP17	0,465	0,047	0,379
MEIS2	0,464	0,088	0,477
SLC66A3	0,463	0,033	0,330
CASC4	0,463	0,046	0,377
OBSL1	0,463	0,044	0,370
CRTAP	0,463	0,011	0,215
PHLDB2	0,462	0,029	0,313
GGTA1P	0,462	0,053	0,392
GOLGA3	0,462	0,030	0,319

BCHE	0,461	0,346	0,740
CCNG2	0,461	0,117	0,516
TNRC18	0,458	0,092	0,481
UGDH	0,458	0,190	0,614
RBMS3	0,457	0,027	0,305
PTPRS	0,457	0,331	0,730
ITPRIPL2	0,457	0,024	0,291
ANXA4	0,456	0,050	0,384
MGLL	0,455	0,062	0,422
WASF2	0,455	0,087	0,475
H6PD	0,455	0,085	0,472
SLC39A10	0,455	0,074	0,450
GSN	0,454	0,157	0,569
ZNF483	0,453	0,022	0,285
CYTH3	0,453	0,011	0,210
RSRP1	0,451	0,030	0,319
MDFIC	0,451	0,073	0,450
PTN	0,448	0,044	0,369
NAALADL2	0,448	0,054	0,394
TK2	0,448	0,021	0,283
ITIH5	0,446	0,089	0,478
EEF1A1	0,446	0,123	0,525
OLFML3	0,445	0,139	0,546
STAT2	0,445	0,059	0,415
SMC4	0,444	0,044	0,369
PRDM11	0,444	0,059	0,414
RANBP10	0,444	0,037	0,344
TRIB1	0,443	0,236	0,655
LZTFL1	0,443	0,049	0,383
PDE8B	0,442	0,103	0,493
CMTM4	0,442	0,074	0,450
ADPRHL1	0,442	0,151	0,563
ZMYM3	0,441	0,066	0,432
TMTC2	0,441	0,130	0,535
NOTCH2	0,440	0,062	0,422
SGMS2	0,439	0,206	0,626
GPRASP1	0,439	0,014	0,234
SHTN1	0,438	0,064	0,427
RCAN1	0,438	0,031	0,321
DCTD	0,437	0,003	0,116
EMB	0,437	0,327	0,726
FGF2	0,437	0,175	0,594
ESYT1	0,437	0,104	0,496
SQSTM1	0,435	0,046	0,377
USP12	0,435	0,071	0,446
HIST1H2AC	0,435	0,091	0,481
OSGIN2	0,435	0,013	0,232
MAPK10	0,434	0,159	0,572
NHSL1	0,434	0,023	0,290
LASP1	0,434	0,098	0,488
TGIF1	0,432	0,095	0,484
PCDH9	0,432	0,066	0,431
PLA2G15	0,432	0,067	0,434

BNIP3L	0,432	0,101	0,491
PRELP	0,431	0,237	0,656
WDR78	0,431	0,086	0,474
PDCD4	0,431	0,049	0,383
HLA-A	0,430	0,014	0,233
GYPC	0,430	0,037	0,344
FBLN2	0,430	0,224	0,646
CPE	0,429	0,089	0,478
ESR1	0,429	0,043	0,368
IL1R1	0,428	0,217	0,638
HLA-E	0,428	0,014	0,234
MARCKS	0,427	0,097	0,485
TXNDC16	0,426	0,062	0,418
NES	0,425	0,134	0,540
RGPD1	0,424	0,252	0,666
PKD2	0,424	0,074	0,450
POLR2A	0,424	0,121	0,522
CHPF2	0,424	0,033	0,330
OAT	0,424	0,095	0,484
PAPSS1	0,423	0,071	0,446
ANGPT1	0,423	0,334	0,731
IL6R	0,421	0,217	0,638
ALDH3A2	0,421	0,052	0,391
GUCY1A1	0,421	0,074	0,451
VWA5A	0,421	0,092	0,481
ITGA3	0,420	0,025	0,295
DGKG	0,420	0,078	0,458
PACS1	0,420	0,093	0,483
ELMOD3	0,419	0,082	0,467
TLE1	0,419	0,126	0,528
NMNAT2	0,419	0,274	0,687
CCDC3	0,418	0,176	0,596
STMN1	0,418	0,030	0,319
RFTN2	0,418	0,093	0,483
CSRP1	0,417	0,142	0,551
RPL3	0,417	0,159	0,572
IGIP	0,416	0,003	0,117
MRNIP	0,416	0,035	0,336
SEC31A	0,416	0,000	0,035
ZNF727	0,416	0,133	0,539
RASA4	0,415	0,177	0,596
ENO2	0,415	0,107	0,501
CTTN	0,414	0,009	0,191
CCN1	0,414	0,372	0,756
BAMBI	0,413	0,021	0,283
BICC1	0,413	0,167	0,585
MCL1	0,412	0,083	0,469
LTBP1	0,412	0,004	0,133
PABPC1	0,411	0,080	0,463
YAP1	0,411	0,030	0,319
APLF	0,411	0,091	0,481
MFAP4	0,410	0,155	0,568
DDAH1	0,409	0,044	0,370

EFR3B	0,409	0,159	0,572
ZMAT1	0,409	0,038	0,347
MOAP1	0,408	0,026	0,297
TULP3	0,408	0,060	0,416
CLMP	0,408	0,346	0,740
DUSP5	0,408	0,235	0,655
DLG5	0,406	0,180	0,600
CIITA	0,405	0,217	0,638
HLA-DMA	0,405	0,085	0,472
ZSCAN31	0,405	0,150	0,560
KLHL11	0,405	0,067	0,434
TENT5C	0,404	0,106	0,500
ARMCX2	0,404	0,004	0,133
ABHD14B	0,404	0,053	0,394
PTPN21	0,404	0,019	0,266
IRF6	0,404	0,063	0,425
MEPCE	0,403	0,029	0,315
FAS	0,402	0,189	0,614
KLF11	0,402	0,034	0,332
AP3M2	0,402	0,017	0,253
SLC40A1	0,401	0,172	0,589
YWHAQ	0,401	0,003	0,114
EFNA1	0,401	0,023	0,290
EMC10	0,400	0,064	0,427
ST8SIA6	0,400	0,042	0,364
GPR34	0,400	0,355	0,746
NOTCH1	0,399	0,131	0,536
LIX1L	0,399	0,025	0,295
SYNJ2	0,399	0,033	0,330
FAM114A1	0,398	0,008	0,180
CAD	0,398	0,136	0,544
ACCS	0,397	0,133	0,539
CNNM2	0,397	0,025	0,295
GFPT1	0,397	0,014	0,233
LIN9	0,397	0,102	0,492
TOB1	0,396	0,032	0,324
FGF1	0,396	0,126	0,527
COL18A1	0,396	0,037	0,345
MID1	0,396	0,115	0,512
FZD4	0,395	0,168	0,587
RASA4B	0,395	0,185	0,609
ITGB3BP	0,395	0,053	0,393
LAMB2	0,395	0,131	0,536
ETV3	0,395	0,010	0,203
XAF1	0,395	0,128	0,532
HIST1H4E	0,394	0,260	0,674
ALDH6A1	0,393	0,048	0,380
SREBF2	0,393	0,130	0,535
NUMA1	0,391	0,069	0,441
CEP250	0,390	0,069	0,438
NEK6	0,389	0,098	0,489
KDM3B	0,389	0,043	0,367
SNX30	0,389	0,048	0,380

ARHGEF40	0,389	0,119	0,519
SPEF2	0,388	0,058	0,409
C1orf198	0,386	0,035	0,336
EPB41L2	0,386	0,071	0,445
RPL10	0,386	0,066	0,432
EFHC1	0,386	0,040	0,355
ST6GAL1	0,385	0,066	0,432
PLA2G2A	0,385	0,471	0,816
TERF2IP	0,385	0,001	0,073
APOL6	0,384	0,374	0,757
EZR	0,384	0,062	0,418
SNX18	0,384	0,055	0,401
UBE2Q2	0,384	0,025	0,297
EHD3	0,383	0,077	0,456
MAN1B1	0,383	0,041	0,358
PAWR	0,383	0,281	0,692
TNFRSF21	0,383	0,275	0,687
JUNB	0,383	0,270	0,684
ZDHHC14	0,383	0,086	0,474
SHANK3	0,382	0,144	0,553
WWP2	0,382	0,039	0,351
PARP12	0,382	0,021	0,279
TMEM9	0,382	0,023	0,290
CELSR1	0,381	0,114	0,512
COL6A6	0,381	0,346	0,740
CES1	0,381	0,252	0,666
DDAH2	0,381	0,069	0,438
SYTL2	0,381	0,104	0,495
CNRIP1	0,381	0,072	0,446
PCDH18	0,380	0,384	0,764
DIXDC1	0,380	0,012	0,219
HLA-F	0,380	0,024	0,290
USP22	0,380	0,070	0,442
SPECC1	0,379	0,030	0,317
MYO18A	0,378	0,199	0,623
HNRNPA1P48	0,378	0,014	0,234
SHROOM3	0,377	0,119	0,518
ATP8B2	0,377	0,088	0,477
CASK	0,376	0,025	0,297
RNF24	0,376	0,226	0,646
EVL	0,376	0,030	0,318
GIGYF1	0,376	0,070	0,441
KIRREL1	0,375	0,195	0,618
TTLL3	0,373	0,140	0,549
PLCG1	0,372	0,117	0,516
PLEKHG3	0,371	0,135	0,542
DOCK1	0,371	0,006	0,169
BRD3	0,371	0,146	0,556
ZNF699	0,370	0,067	0,434
ILDR2	0,370	0,126	0,527
CSNK1G3	0,370	0,135	0,541
FTL	0,369	0,245	0,661
SLC38A10	0,369	0,056	0,403

ID1	0,369	0,054	0,394
RPL15	0,368	0,083	0,468
NBPF1	0,368	0,178	0,598
SCRN1	0,367	0,017	0,253
TLE4	0,367	0,022	0,285
UBE2L6	0,367	0,096	0,484
TBX20	0,367	0,248	0,661
RPS27	0,367	0,085	0,473
SNAP47	0,367	0,117	0,516
SAMD9	0,367	0,210	0,631
INPP5F	0,367	0,017	0,254
SORBS2	0,367	0,232	0,653
ZMAT3	0,366	0,119	0,518
DNAL1	0,366	0,008	0,188
PTPRA	0,365	0,006	0,171
MNDA	0,364	0,428	0,792
PYGL	0,363	0,407	0,779
MYOF	0,362	0,163	0,578
ZNF124	0,362	0,196	0,620
DZIP1	0,362	0,111	0,506
FBXL20	0,361	0,061	0,417
LIMA1	0,361	0,113	0,510
LIPA	0,360	0,256	0,669
NEMP1	0,360	0,053	0,392
NAV2	0,360	0,132	0,538
MECOM	0,360	0,034	0,332
MEOX2	0,359	0,092	0,482
MSH3	0,359	0,036	0,342
HMGN1	0,358	0,015	0,235
PJA1	0,358	0,117	0,515
JOSD1	0,358	0,063	0,425
CCDC7	0,358	0,163	0,578
ICK	0,358	0,049	0,381
G0S2	0,357	0,484	0,822
MED12	0,357	0,128	0,532
UBTF	0,356	0,035	0,336
SGIP1	0,355	0,054	0,394
CCDC117	0,355	0,027	0,305
TNPO2	0,355	0,108	0,502
CES2	0,353	0,074	0,450
RETREG3	0,353	0,056	0,402
RASGEF1B	0,353	0,125	0,527
NISCH	0,353	0,065	0,431
CEP164	0,352	0,107	0,501
AHCYL2	0,352	0,051	0,388
RETREG2	0,352	0,043	0,368
JUN	0,352	0,115	0,512
SEMA4C	0,351	0,095	0,484
TNS3	0,351	0,094	0,484
INF2	0,351	0,126	0,528
SVIL	0,351	0,133	0,539
TM7SF3	0,350	0,020	0,278
DYNC2H1	0,350	0,151	0,562

ARL6IP1	0,349	0,215	0,637
TIMP2	0,349	0,124	0,526
RPL9	0,349	0,130	0,535
EPB41L1	0,348	0,141	0,550
UPF1	0,348	0,092	0,482
ZFP36L1	0,347	0,176	0,596
RNF207	0,347	0,245	0,661
CDK6	0,347	0,182	0,604
THRA	0,346	0,078	0,459
NAF1	0,346	0,187	0,612
CAVIN1	0,345	0,116	0,514
TRAF5	0,345	0,290	0,702
SPTBN1	0,345	0,126	0,527
ZNF577	0,344	0,024	0,293
NR1D2	0,344	0,123	0,525
PARP8	0,343	0,073	0,448
UBE2H	0,343	0,020	0,274
CDH2	0,343	0,186	0,610
ADAM32	0,343	0,178	0,599
SPTAN1	0,342	0,125	0,527
RNF216	0,342	0,021	0,282
CREBL2	0,342	0,072	0,446
KIAA0753	0,341	0,072	0,446
NFE2L2	0,341	0,051	0,388
EDEM1	0,341	0,053	0,393
NFATC2	0,341	0,054	0,396
IL13RA1	0,340	0,128	0,532
UNG	0,340	0,057	0,405
TRAF3	0,340	0,036	0,339
MAN2B2	0,340	0,046	0,377
MTMR3	0,339	0,066	0,432
DLGAP4	0,339	0,150	0,562
ZFP36L2	0,339	0,202	0,624
IDS	0,339	0,085	0,472
RPL13	0,339	0,140	0,547
ARHGEF9	0,339	0,092	0,481
CYLD	0,338	0,016	0,249
ZC3H4	0,338	0,101	0,491
PPFIA4	0,338	0,255	0,668
CYP2U1	0,338	0,010	0,207
EYA4	0,338	0,432	0,794
FREM1	0,338	0,157	0,569
CAMK2B	0,337	0,149	0,559
FOXK1	0,337	0,222	0,645
FAM177A1	0,337	0,068	0,436
CYSTM1	0,336	0,110	0,505
PTPRJ	0,336	0,155	0,568
KMT2A	0,335	0,097	0,485
ARSB	0,335	0,052	0,391
ZNF697	0,335	0,031	0,321
SYNE1	0,335	0,138	0,546
VPS51	0,334	0,100	0,489
BBS4	0,333	0,058	0,409

SRGAP2C	0,333	0,196	0,620
PTMA	0,333	0,227	0,647
MYH7B	0,332	0,223	0,646
DAP	0,332	0,058	0,411
HOOK3	0,331	0,038	0,347
RPL12	0,331	0,150	0,562
TBL2	0,331	0,077	0,455
ATP10D	0,331	0,028	0,309
CCDC171	0,330	0,087	0,476
HLA-C	0,329	0,096	0,484
GBP1	0,328	0,281	0,693
METTL7A	0,328	0,410	0,782
NBPF14	0,328	0,141	0,549
TBC1D9B	0,328	0,201	0,624
ZC3H7B	0,327	0,162	0,577
PYGB	0,327	0,177	0,598
CASP1	0,326	0,192	0,616
CCDC115	0,326	0,064	0,426
SSR4	0,326	0,137	0,545
CDKN1B	0,326	0,007	0,171
IFIH1	0,325	0,142	0,551
NMRK1	0,325	0,124	0,525
ATP13A3	0,325	0,178	0,598
MAP3K5	0,325	0,157	0,569
TNIP1	0,325	0,078	0,459
SMURF2	0,324	0,039	0,352
RXRB	0,323	0,053	0,394
NPTXR	0,323	0,319	0,721
CPNE5	0,323	0,359	0,749
TMEM47	0,323	0,065	0,431
ATL3	0,322	0,295	0,706
HERPUD1	0,322	0,061	0,416
TOB2	0,322	0,110	0,505
FUCA1	0,321	0,112	0,507
DUSP16	0,321	0,023	0,285
CFAP69	0,320	0,291	0,702
IPP	0,320	0,047	0,379
SLC25A6	0,319	0,089	0,478
CTSF	0,319	0,072	0,446
SMARCC2	0,319	0,123	0,525
TMOD3	0,319	0,153	0,565
KANK1	0,319	0,090	0,481
RSPH3	0,319	0,046	0,377
NBPF10	0,319	0,082	0,467
PPP1R15A	0,319	0,118	0,518
LNX2	0,319	0,019	0,269
PXK	0,318	0,025	0,295
PIP4K2A	0,318	0,157	0,569
SFXN3	0,317	0,089	0,479
GCC1	0,317	0,095	0,484
DPY19L3	0,317	0,200	0,623
IFT122	0,317	0,109	0,503
NBPF26	0,316	0,173	0,591

ADH1C	0,316	0,446	0,799
TSC2	0,316	0,160	0,575
CNTRL	0,316	0,107	0,501
P4HTM	0,316	0,059	0,415
CAT	0,316	0,175	0,595
PLEC	0,316	0,206	0,626
SCRN2	0,316	0,218	0,639
RFWD3	0,316	0,107	0,501
EIF3L	0,316	0,121	0,521
IFIT3	0,315	0,220	0,643
ZCCHC14	0,315	0,079	0,460
NRAP	0,315	0,338	0,735
COG7	0,314	0,097	0,485
ZNFX1	0,314	0,076	0,452
MAML2	0,314	0,172	0,590
PDE3B	0,314	0,366	0,755
MGAT4B	0,313	0,047	0,379
ZER1	0,313	0,123	0,525
LPL	0,312	0,405	0,777
MYO18B	0,312	0,381	0,761
MYOM2	0,311	0,370	0,756
MAPT	0,311	0,444	0,798
PIK3CG	0,310	0,180	0,600
DOCK11	0,310	0,500	0,829
RPS9	0,310	0,214	0,635
ZNF862	0,310	0,131	0,536
RNF38	0,310	0,025	0,295
ODF2	0,309	0,040	0,352
YOD1	0,309	0,335	0,732
TTC8	0,309	0,089	0,478
EBF3	0,309	0,086	0,475
TCTN3	0,309	0,027	0,305
MAP3K3	0,308	0,102	0,492
THAP12	0,308	0,091	0,481
SLC25A20	0,308	0,143	0,551
TTC5	0,307	0,086	0,474
TCF7L2	0,307	0,123	0,525
FRMD6	0,307	0,302	0,712
SEPTIN8	0,307	0,044	0,371
MAP2	0,307	0,206	0,626
IFT88	0,307	0,108	0,502
PPP1R21	0,307	0,014	0,234
TBC1D12	0,307	0,147	0,556
NECTIN3	0,306	0,192	0,616
CPNE2	0,306	0,115	0,513
CAMSAP1	0,306	0,028	0,311
CFAP70	0,306	0,154	0,566
NUAK1	0,306	0,119	0,518
OGFRL1	0,306	0,265	0,678
PDE5A	0,305	0,316	0,720
EFNA5	0,305	0,085	0,472
CCDC8	0,304	0,103	0,493
RNF19B	0,303	0,153	0,565

ZCCHC24	0,303	0,101	0,491
RFX7	0,303	0,038	0,349
TOM1L2	0,303	0,246	0,661
GPRASP2	0,303	0,066	0,431
SMARCD3	0,302	0,064	0,426
SLC35F5	0,302	0,167	0,585
NOP53	0,302	0,147	0,556
SMARCC1	0,301	0,033	0,330
LMCD1	0,301	0,323	0,723
KDM5B	0,301	0,072	0,446
HEYL	0,301	0,288	0,700
SLK	0,301	0,125	0,527
EDNRA	0,300	0,198	0,622
MYO10	0,300	0,261	0,675
TTLL7	0,300	0,085	0,472
ZNF496	0,300	0,100	0,489
RPL36	0,300	0,232	0,653
TRAF3IP1	0,300	0,096	0,484
ZFPM2	0,300	0,041	0,361
PGPEP1	0,300	0,085	0,472
SIRT3	0,300	0,135	0,542
DSE	0,299	0,332	0,730
RPS21	0,299	0,100	0,489
ZXDB	0,299	0,091	0,481
IER5	0,299	0,143	0,551
KANK2	0,299	0,110	0,504
SEC24A	0,299	0,076	0,453
COPG1	0,299	0,025	0,295
PLA2G4C	0,299	0,110	0,505
SRSF8	0,299	0,008	0,190
SLC23A2	0,299	0,133	0,539
FNBP1	0,298	0,141	0,550
MAGED1	0,298	0,051	0,388
CEBPB	0,297	0,234	0,654
TASP1	0,297	0,088	0,477
PRPF6	0,297	0,091	0,481
SLC17A5	0,296	0,058	0,410
TWSG1	0,296	0,132	0,537
POGZ	0,296	0,130	0,534
INTS3	0,296	0,116	0,514
NFRKB	0,296	0,099	0,489
LMTK2	0,296	0,205	0,625
TRPS1	0,296	0,226	0,646
PDPR	0,295	0,243	0,660
LONRF2	0,295	0,278	0,690
ATP2B4	0,295	0,118	0,518
SLC35D1	0,295	0,047	0,379
STX6	0,294	0,025	0,295
ARF3	0,294	0,139	0,547
CXCL12	0,294	0,090	0,481
JAK2	0,294	0,333	0,730
ADNP	0,294	0,013	0,232
CLUAP1	0,294	0,046	0,377

TCTA	0,294	0,102	0,492
VOPP1	0,293	0,055	0,401
KDM5D	0,293	0,596	0,880
ANKAR	0,293	0,065	0,431
ZFYVE21	0,293	0,094	0,484
ATF6B	0,293	0,128	0,532
SNX1	0,293	0,035	0,336
UNC5C	0,292	0,327	0,726
DCUN1D3	0,292	0,310	0,715
TNXB	0,292	0,460	0,810
IFT80	0,292	0,193	0,617
EIF2AK3	0,292	0,274	0,687
CEP89	0,292	0,136	0,544
NCAPD2	0,291	0,142	0,551
TTC3	0,291	0,023	0,285
FAM171B	0,291	0,263	0,677
KLF10	0,291	0,426	0,791
SRGAP2B	0,291	0,249	0,662
GNG2	0,291	0,228	0,648
SPATA20	0,291	0,249	0,662
ASB1	0,291	0,083	0,468
HEPH	0,291	0,114	0,511
TKT	0,290	0,452	0,805
CYB5R1	0,290	0,091	0,481
POLR1E	0,290	0,094	0,484
SPRYD3	0,290	0,124	0,525
TRMT112	0,289	0,116	0,514
COLEC12	0,289	0,307	0,714
STAT5B	0,289	0,112	0,507
ANXA1	0,289	0,400	0,774
ZNF264	0,289	0,039	0,351
VKORC1L1	0,289	0,234	0,655
ATXN1	0,289	0,081	0,464
TBC1D8	0,289	0,181	0,602
RPL19	0,289	0,140	0,547
IFT57	0,288	0,114	0,512
PLS3	0,288	0,229	0,649
ERLIN1	0,288	0,136	0,544
ADAMTS7	0,288	0,177	0,596
ARMCX3	0,288	0,039	0,351
ALDH5A1	0,287	0,138	0,546
RPL7A	0,287	0,159	0,572
FZD1	0,287	0,104	0,496
ARPIN	0,287	0,198	0,622
LETMD1	0,287	0,033	0,330
PRRC2B	0,286	0,395	0,771
STRN	0,286	0,133	0,540
CPT2	0,286	0,129	0,534
SPTB	0,286	0,423	0,789
KIF13B	0,286	0,051	0,388
HNRNPA1	0,286	0,038	0,348
STAM	0,286	0,025	0,295
SLC35E2B	0,286	0,171	0,588

SUMF2	0,286	0,155	0,568
RAP2B	0,286	0,256	0,669
DYNC1LI2	0,286	0,014	0,233
CPPED1	0,285	0,060	0,416
GPR108	0,285	0,111	0,506
ZSCAN18	0,285	0,108	0,502
KIFC3	0,285	0,148	0,558
RPL18	0,285	0,192	0,616
TNFRSF10B	0,284	0,203	0,625
GPC4	0,284	0,344	0,739
TMEM43	0,284	0,072	0,446
SLC16A4	0,284	0,147	0,556
TRIM27	0,284	0,022	0,285
ARHGAP32	0,284	0,213	0,634
MT-ND5	0,284	0,530	0,846
EOGT	0,283	0,153	0,565
SETD7	0,283	0,012	0,219
ARMH3	0,283	0,081	0,466
TRIM56	0,283	0,239	0,657
COPA	0,283	0,008	0,180
FBXO33	0,283	0,211	0,632
FAU	0,283	0,162	0,578
ZNF740	0,283	0,087	0,475
UBN2	0,283	0,074	0,450
TF	0,282	0,687	0,914
ZNF83	0,281	0,048	0,380
SMN1	0,281	0,106	0,500
LGALS8	0,281	0,134	0,540
C11orf95	0,281	0,119	0,518
PARD3B	0,281	0,116	0,514
TUBGCP6	0,280	0,191	0,616
AKR1C3	0,280	0,417	0,784
PLXND1	0,280	0,263	0,677
COL5A1	0,280	0,320	0,721
LIMCH1	0,280	0,093	0,483
RNF40	0,280	0,196	0,620
PPFIBP1	0,279	0,105	0,497
CNNM3	0,279	0,177	0,596
BHLHE40	0,279	0,357	0,747
EVI5	0,279	0,101	0,491
RPS6	0,279	0,213	0,634
BCR	0,279	0,331	0,729
SLC12A4	0,279	0,180	0,600
RCC2	0,278	0,224	0,646
XRRA1	0,278	0,204	0,625
SAMD9L	0,278	0,277	0,689
ZNF275	0,278	0,126	0,527
DSEL	0,278	0,395	0,771
RBM24	0,277	0,383	0,763
ARIH2	0,277	0,141	0,550
RPL10A	0,277	0,283	0,695
NIPAL2	0,277	0,122	0,522
NREP	0,277	0,353	0,744

PHLDB1	0,276	0,347	0,742
DNMT3A	0,276	0,119	0,518
SPIRE1	0,276	0,095	0,484
GMCL1	0,276	0,112	0,507
TMEM94	0,276	0,258	0,672
ZNF616	0,276	0,143	0,551
BICRAL	0,276	0,093	0,483
NEDD9	0,275	0,335	0,732
CDC42BPB	0,275	0,254	0,667
SERF1B	0,275	0,114	0,512
HACD3	0,275	0,113	0,510
SPPL3	0,275	0,056	0,404
TMEM248	0,275	0,048	0,380
CALM3	0,275	0,187	0,611
WWTR1	0,275	0,133	0,538
RNF115	0,274	0,139	0,546
SERF1A	0,274	0,117	0,517
TSC22D2	0,273	0,190	0,614
ASAP2	0,273	0,293	0,703
TOR1AIP2	0,272	0,202	0,624
AFAP1L1	0,272	0,076	0,452
NAAA	0,272	0,147	0,556
GOLGA2	0,271	0,098	0,487
RPL13A	0,271	0,253	0,666
XIRP2	0,271	0,570	0,865
CCND2	0,271	0,188	0,613
ALDH4A1	0,271	0,274	0,687
FAH	0,270	0,420	0,787
NEK4	0,270	0,080	0,461
SEPTIN6	0,269	0,119	0,518
SUPT5H	0,269	0,185	0,608
APMAP	0,269	0,191	0,616
DAPK3	0,269	0,265	0,678
RPS19	0,269	0,276	0,689
CCDC28A	0,268	0,147	0,556
SELENOP	0,268	0,368	0,756
MPHOSPH8	0,268	0,028	0,309
SGTB	0,268	0,125	0,527
FUBP3	0,268	0,075	0,452
PDE2A	0,268	0,287	0,700
SEMA4D	0,268	0,163	0,578
NSMCE1	0,268	0,149	0,559
MYO1C	0,268	0,255	0,668
ZBTB4	0,268	0,165	0,580
LZTS2	0,267	0,109	0,504
WWC3	0,267	0,100	0,489
CD4	0,267	0,322	0,723
PDCD7	0,267	0,136	0,544
MAU2	0,267	0,171	0,588
ARF4	0,267	0,107	0,501
DCAKD	0,266	0,142	0,551
GNL1	0,266	0,126	0,528
PDE7B	0,266	0,235	0,655

BET1L	0,266	0,178	0,598
RPS14	0,266	0,212	0,634
SAMD12	0,266	0,129	0,534
KHSRP	0,266	0,207	0,628
OTUD5	0,265	0,083	0,468
CDK4	0,265	0,104	0,496
NIPSNAP1	0,265	0,140	0,549
HERPUD2	0,265	0,047	0,379
FAT4	0,265	0,097	0,485
OAS2	0,265	0,156	0,568
ZNF471	0,265	0,095	0,484
SYNPO2L	0,265	0,416	0,784
ADAR	0,265	0,117	0,517
SCAI	0,264	0,061	0,417
PFKL	0,264	0,214	0,634
ATXN1L	0,264	0,153	0,565
CUL4B	0,264	0,126	0,528
NOP9	0,264	0,121	0,521
FMR1	0,264	0,081	0,464
AKNA	0,263	0,355	0,746
DYNC2LI1	0,263	0,100	0,489
STS	0,263	0,084	0,472
ATP11A	0,263	0,293	0,704
OGT	0,263	0,060	0,416
ABCC1	0,263	0,275	0,687
TNFRSF12A	0,263	0,527	0,844
GATA6	0,263	0,049	0,381
TBC1D8B	0,262	0,099	0,489
IFT43	0,262	0,116	0,514
CMKLR1	0,262	0,208	0,629
RFFL	0,262	0,194	0,618
DISP1	0,262	0,180	0,600
ABI1	0,262	0,053	0,392
BDH2	0,262	0,190	0,614
HGSNAT	0,262	0,142	0,551
TP53INP2	0,262	0,323	0,724
TSPYL2	0,262	0,178	0,599
RPS3	0,261	0,217	0,638
TLN1	0,261	0,294	0,705
SART3	0,261	0,061	0,416
ALDH9A1	0,261	0,100	0,489
MBNL3	0,261	0,397	0,774
SLC4A2	0,261	0,293	0,703
CYTH2	0,261	0,113	0,509
ARMC10	0,261	0,064	0,427
VSTM4	0,260	0,242	0,659
YPEL2	0,260	0,070	0,441
USP19	0,260	0,205	0,625
PRKCH	0,260	0,047	0,379
HDAC5	0,260	0,138	0,546
WIPF2	0,259	0,167	0,585
NBPF9	0,259	0,168	0,587
DAXX	0,259	0,187	0,611

TGFBRAP1	0,258	0,117	0,516
INPP5D	0,258	0,246	0,661
RPL18A	0,258	0,257	0,670
PDK3	0,258	0,077	0,455
RPL35A	0,258	0,233	0,654
FAM156B	0,258	0,109	0,503
C6orf62	0,257	0,104	0,496
SBF1	0,257	0,245	0,661
ERLEC1	0,257	0,087	0,475
THBS4	0,257	0,313	0,718
SLC30A7	0,257	0,266	0,679
SLC38A2	0,256	0,491	0,824
NAB1	0,256	0,197	0,621
B4GALT4	0,256	0,140	0,549
MAPK4	0,255	0,441	0,797
TACC2	0,255	0,472	0,816
PRKAR1A	0,255	0,316	0,719
AAMP	0,255	0,124	0,525
GNAQ	0,253	0,251	0,665
CDK8	0,253	0,139	0,546
SLC25A27	0,253	0,168	0,585
EFEMP2	0,253	0,117	0,517
SERPING1	0,253	0,352	0,744
SMN2	0,253	0,185	0,608
NBL1	0,253	0,313	0,717
SPRED2	0,253	0,154	0,566
RNH1	0,253	0,273	0,685
PWWP3A	0,252	0,137	0,545
SLC30A5	0,252	0,087	0,475
SH2B1	0,252	0,199	0,623
MTMR12	0,252	0,054	0,394
SEPTIN2	0,252	0,039	0,351
ZMYM4	0,252	0,020	0,278
NBPF20	0,252	0,161	0,577
PEAK1	0,252	0,132	0,537
RPS3A	0,252	0,322	0,723
CEP104	0,251	0,107	0,502
EDC4	0,251	0,302	0,712
EIF2AK2	0,251	0,139	0,547
TLR1	0,251	0,368	0,756
MT-ATP8	0,251	0,510	0,835
JMY	0,251	0,210	0,631
AMMECR1	0,251	0,189	0,613
IRF2BP2	0,251	0,039	0,352
ARAP1	0,251	0,209	0,630
ZNF793	0,251	0,217	0,638
EZH1	0,250	0,110	0,505
MAPK8IP3	0,250	0,246	0,661
SPRY2	0,249	0,082	0,467
ARHGAP21	0,249	0,104	0,495
USP30	0,249	0,162	0,577
SLC2A12	0,249	0,425	0,791
MCC	0,249	0,196	0,620

CCNY	0,248	0,014	0,234
SH3D19	0,248	0,095	0,484
WDR35	0,248	0,153	0,565
ENDOD1	0,248	0,117	0,516
PGM2	0,248	0,332	0,730
GTPBP1	0,248	0,219	0,641
HEXB	0,248	0,096	0,484
FAM156A	0,248	0,131	0,536
GABPB2	0,248	0,054	0,395
EAF1	0,247	0,205	0,625
CMPK1	0,247	0,186	0,610
SOX6	0,247	0,126	0,527
PTPDC1	0,246	0,175	0,595
ZBTB34	0,246	0,102	0,492
CDK19	0,246	0,139	0,546
NPR1	0,246	0,498	0,828
CERS2	0,246	0,249	0,662
OGA	0,246	0,072	0,446
SCN4B	0,246	0,291	0,702
SAMD4A	0,246	0,313	0,718
LAMB1	0,245	0,317	0,720
RAB22A	0,245	0,179	0,599
ADH1B	0,245	0,679	0,911
MPPE1	0,245	0,185	0,609
SULF2	0,244	0,159	0,572
ABCA2	0,244	0,266	0,679
SKI	0,244	0,387	0,766
SLFN5	0,243	0,267	0,680
ZKSCAN1	0,243	0,073	0,447
SEC16A	0,243	0,320	0,721
GPR107	0,243	0,116	0,514
AIFM2	0,243	0,409	0,781
RASSF5	0,243	0,205	0,625
RHOBTB1	0,243	0,034	0,332
TMEM170A	0,242	0,148	0,557
DDX42	0,242	0,034	0,333
ABHD18	0,242	0,294	0,705
GID8	0,242	0,049	0,381
CASP7	0,242	0,264	0,678
ABR	0,242	0,330	0,729
CUL9	0,241	0,288	0,700
PBX1	0,241	0,231	0,651
PRMT9	0,241	0,220	0,642
DHODH	0,241	0,169	0,587
TOR1AIP1	0,241	0,024	0,291
TSR2	0,241	0,078	0,459
PHACTR2	0,240	0,176	0,595
ZBTB5	0,240	0,200	0,623
SH3RF1	0,240	0,176	0,596
REST	0,240	0,071	0,446
UBE2Z	0,240	0,088	0,476
FOXJ2	0,240	0,227	0,647
CSNK1G1	0,240	0,155	0,568

CPD	0,240	0,096	0,484
FNDC3A	0,239	0,245	0,661
SLC30A4	0,239	0,147	0,556
TTC28	0,239	0,263	0,677
SMOC2	0,239	0,207	0,628
PDE1C	0,239	0,456	0,807
RPS29	0,239	0,346	0,740
TRAFD1	0,239	0,080	0,461
HLA-B	0,239	0,260	0,674
MLLT6	0,238	0,280	0,692
ARHGEF2	0,238	0,202	0,624
UACA	0,238	0,231	0,652
PNMA1	0,238	0,128	0,532
TCF25	0,238	0,191	0,616
CUX1	0,238	0,227	0,647
RPS8	0,238	0,318	0,721
NBPF15	0,238	0,157	0,569
MICAL3	0,238	0,447	0,800
PPM1F	0,237	0,189	0,613
TBC1D19	0,237	0,128	0,531
NOTCH2NLA	0,237	0,270	0,683
ZFP90	0,237	0,071	0,443
MGST1	0,237	0,673	0,908
APLNR	0,237	0,593	0,879
PHC1	0,236	0,278	0,690
PHF8	0,236	0,244	0,661
SERTAD2	0,236	0,079	0,460
TRIM22	0,236	0,316	0,719
SLC25A16	0,236	0,158	0,571
URB1	0,235	0,246	0,661
PLAGL1	0,235	0,238	0,656
MANBA	0,235	0,061	0,416
GXYLT1	0,235	0,162	0,578
TRIB2	0,234	0,091	0,481
CHST3	0,234	0,244	0,660
MED29	0,234	0,149	0,559
MBTD1	0,234	0,068	0,436
ZSWIM8	0,234	0,288	0,700
BACE2	0,234	0,243	0,660
PPARG	0,233	0,609	0,885
NACA	0,233	0,247	0,661
CCDC50	0,233	0,136	0,544
TMEM87B	0,233	0,300	0,710
HGS	0,233	0,200	0,624
SH3BP4	0,233	0,137	0,545
PTK2B	0,233	0,411	0,782
INTS1	0,232	0,236	0,655
WDR6	0,232	0,225	0,646
TPD52L2	0,232	0,060	0,415
SMIM14	0,232	0,223	0,646
RHOB	0,232	0,255	0,668
RETSAT	0,232	0,521	0,842
CEP162	0,232	0,191	0,616

COMMD6	0,231	0,338	0,734
CPT1A	0,231	0,238	0,656
OSBP	0,231	0,073	0,448
FAM160A2	0,231	0,204	0,625
AFAP1	0,231	0,300	0,710
BBS9	0,231	0,061	0,416
SUPT6H	0,231	0,197	0,621
CEP78	0,231	0,163	0,578
PCDHGC3	0,231	0,426	0,791
ZNF844	0,231	0,230	0,649
HNRNPA1L2	0,230	0,093	0,483
PPP4R3B	0,230	0,165	0,580
IP6K1	0,230	0,221	0,643
RPL30	0,230	0,281	0,692
NEDD4	0,230	0,256	0,669
NR3C1	0,230	0,102	0,493
STK4	0,229	0,173	0,591
ZNF623	0,229	0,094	0,484
SAMD4B	0,229	0,342	0,737
PAIP2B	0,229	0,454	0,806
CALHM5	0,229	0,253	0,666
ZMYND8	0,229	0,189	0,613
TGFBR1	0,229	0,293	0,703
BCL2L2	0,229	0,079	0,460
SPATS2	0,229	0,251	0,664
RIC8A	0,229	0,125	0,527
KCTD18	0,229	0,179	0,600
UBTD2	0,228	0,203	0,625
MINDY1	0,227	0,285	0,697
SH3TC2	0,227	0,373	0,756
LRRCC1	0,227	0,249	0,662
ADO	0,227	0,118	0,518
PRKAR2B	0,227	0,654	0,899
ECE1	0,227	0,324	0,724
AKR7A2	0,227	0,158	0,570
NOTCH4	0,227	0,369	0,756
DNAH1	0,226	0,423	0,789
DHX38	0,226	0,250	0,664
WDFY1	0,226	0,221	0,644
DGAT2	0,226	0,732	0,931
RRN3	0,226	0,118	0,517
KIZ	0,226	0,140	0,549
ZBTB25	0,226	0,183	0,605
SPECC1L	0,225	0,112	0,507
ATG13	0,225	0,143	0,551
NPR2	0,225	0,306	0,713
OSBPL2	0,225	0,167	0,585
ZNF514	0,225	0,095	0,484
PDXK	0,225	0,383	0,763
ZDBF2	0,225	0,188	0,613
IP6K2	0,225	0,176	0,595
MOSPD2	0,225	0,193	0,617
GOLGA8N	0,225	0,217	0,638

DHTKD1	0,225	0,271	0,684
FBLN5	0,224	0,423	0,789
MED13	0,224	0,132	0,537
SYVN1	0,224	0,301	0,711
BSDC1	0,224	0,215	0,637
DIPK2A	0,224	0,296	0,706
FAM3C	0,224	0,152	0,565
ARRB1	0,224	0,371	0,756
CDH13	0,224	0,248	0,662
NEU3	0,224	0,199	0,623
STK38L	0,223	0,516	0,838
MAGED2	0,223	0,173	0,592
CACUL1	0,223	0,064	0,427
EHBP1L1	0,223	0,213	0,634
RECK	0,223	0,213	0,634
DPYD	0,223	0,394	0,770
ANXA5	0,223	0,340	0,736
ALAD	0,223	0,270	0,683
CAP2	0,222	0,317	0,720
SGSM2	0,222	0,248	0,661
CCN5	0,222	0,555	0,858
LATS2	0,222	0,195	0,618
FARP2	0,222	0,129	0,534
ANKH	0,222	0,137	0,546
TJP1	0,222	0,072	0,446
GLG1	0,221	0,247	0,661
SNRNP200	0,221	0,290	0,701
GARNL3	0,221	0,277	0,690
BAZ1B	0,221	0,101	0,491
BBS7	0,220	0,215	0,637
FAM13A	0,220	0,305	0,712
LRRFIP1	0,220	0,079	0,460
TAF1	0,219	0,126	0,527
ZFP14	0,219	0,178	0,599
TMEM209	0,219	0,199	0,623
UNC45A	0,219	0,268	0,680
ZFYVE27	0,219	0,201	0,624
KIAA1324L	0,219	0,246	0,661
KDM1B	0,219	0,151	0,562
CBFB	0,219	0,171	0,588
PLCL1	0,218	0,120	0,519
EIF3F	0,218	0,069	0,441
BCOR	0,218	0,280	0,692
EIF4B	0,218	0,265	0,678
UHMK1	0,218	0,260	0,674
SLX4IP	0,218	0,258	0,672
TCEANC2	0,218	0,260	0,674
DVL3	0,218	0,238	0,656
TAOK2	0,218	0,198	0,622
CLEC14A	0,218	0,086	0,474
MYO15B	0,217	0,411	0,782
PI4K2B	0,217	0,235	0,655
SLC25A37	0,217	0,433	0,795

ACTG1	0,217	0,437	0,796
PEG3	0,217	0,368	0,756
DTX3L	0,217	0,380	0,761
PNRC1	0,217	0,167	0,585
TMEM150C	0,217	0,340	0,736
CYP20A1	0,217	0,144	0,554
RPS27A	0,217	0,382	0,763
SOCS7	0,216	0,253	0,666
UTP25	0,216	0,154	0,566
RRAS2	0,216	0,349	0,743
ZNF627	0,216	0,211	0,632
TMED8	0,215	0,139	0,546
RBM22	0,215	0,091	0,481
TSPAN3	0,215	0,208	0,629
EPB41L4A	0,215	0,147	0,556
SNX5	0,215	0,087	0,475
KMT2D	0,215	0,565	0,862
ADNP2	0,214	0,134	0,540
ARL13B	0,214	0,247	0,661
ZNF827	0,214	0,236	0,655
CBX5	0,214	0,199	0,622
FCHO2	0,214	0,288	0,700
RELN	0,214	0,681	0,911
GABBR1	0,214	0,345	0,740
PCOLCE2	0,213	0,273	0,686
KDM4A	0,213	0,275	0,688
LRRC8D	0,213	0,235	0,655
IFRD1	0,213	0,283	0,695
URGCP	0,213	0,144	0,553
EPB41	0,213	0,186	0,610
MBP	0,213	0,207	0,628
TGFB3	0,213	0,238	0,656
GANAB	0,213	0,262	0,676
KBTBD7	0,212	0,188	0,613
JRKL	0,212	0,218	0,640
WBP1L	0,212	0,315	0,718
TANGO2	0,212	0,333	0,730
GLRB	0,212	0,245	0,661
IPO5	0,212	0,191	0,616
PLEKHM3	0,212	0,170	0,588
PYGO2	0,212	0,287	0,700
SIN3B	0,212	0,286	0,699
ZNF667	0,212	0,298	0,709
RPS15A	0,212	0,390	0,768
ZFYVE1	0,211	0,231	0,652
P4HB	0,211	0,205	0,625
ATF1	0,211	0,185	0,608
NBR1	0,211	0,067	0,434
GATD1	0,211	0,243	0,660
AQP7	0,211	0,516	0,838
KIDINS220	0,211	0,048	0,380
BTD	0,211	0,264	0,678
TMEM18	0,211	0,210	0,631

HP1BP3	0,211	0,126	0,527
PRDX4	0,211	0,229	0,649
TANC2	0,210	0,244	0,660
MAP3K2	0,210	0,270	0,683
ARHGEF17	0,210	0,318	0,721
STX16	0,210	0,176	0,596
FBXW11	0,210	0,047	0,380
NAPEPLD	0,210	0,240	0,659
PCLO	0,210	0,303	0,712
HOOK2	0,209	0,390	0,768
CAMLG	0,209	0,234	0,655
IMPDH2	0,209	0,265	0,678
OAZ2	0,209	0,205	0,625
PPP2R1B	0,209	0,384	0,764
TRIM38	0,209	0,360	0,750
SLC27A1	0,209	0,350	0,743
AKAP11	0,209	0,070	0,441
MFN2	0,208	0,389	0,768
TPR	0,208	0,116	0,514
WEE1	0,208	0,378	0,761
ACVR2B	0,208	0,225	0,646
TECPR2	0,208	0,320	0,721
TP53INP1	0,208	0,326	0,725
TNKS2	0,208	0,197	0,621
IBA57	0,208	0,384	0,764
EIF3CL	0,207	0,296	0,707
DPYSL3	0,207	0,498	0,828
UNC119B	0,207	0,201	0,624
GOPC	0,207	0,148	0,557
ADA2	0,207	0,524	0,842
SUMF1	0,206	0,184	0,608
DIPK1A	0,206	0,404	0,776
NFYA	0,206	0,166	0,583
SNRK	0,206	0,120	0,519
RASAL2	0,206	0,171	0,588
RNF180	0,205	0,244	0,661
DENND1A	0,205	0,258	0,671
KIAA1328	0,205	0,235	0,655
STAT6	0,205	0,332	0,730
SARAF	0,205	0,135	0,542
KMT2E	0,205	0,074	0,451
ENTPD7	0,205	0,445	0,799
TIAM2	0,205	0,297	0,708
SMG6	0,205	0,403	0,775
CNN2	0,205	0,361	0,750
CALD1	0,204	0,296	0,707
BTG1	0,204	0,155	0,568
PURA	0,204	0,153	0,565
UBXN7	0,204	0,169	0,587
ZNF462	0,204	0,302	0,711
ATP7A	0,203	0,112	0,507
EIF3C	0,203	0,310	0,715
RPS12	0,203	0,445	0,799

ANKRD1	0,203	0,653	0,899
DDX6	0,203	0,127	0,530
C1GALT1	0,203	0,234	0,655
ZNF805	0,203	0,089	0,478
CBX6	0,203	0,361	0,750
CBFA2T2	0,203	0,274	0,687
APH1A	0,203	0,127	0,530
FAM8A1	0,203	0,254	0,667
RPTOR	0,203	0,336	0,732
POMT1	0,202	0,230	0,649
HS2ST1	0,202	0,291	0,702
MPRIP	0,202	0,290	0,701
EIF2D	0,202	0,078	0,459
ZNF525	0,202	0,344	0,739
EP300	0,202	0,402	0,774
SGSM1	0,202	0,550	0,856
CLSTN1	0,202	0,371	0,756
THRB	0,202	0,204	0,625
PHTF1	0,202	0,221	0,644
PPIP5K1	0,202	0,170	0,588
ATP5IF1	0,202	0,248	0,661
TSPYL1	0,202	0,098	0,488
SSH1	0,202	0,336	0,732
IQCB1	0,202	0,188	0,613
RPL39	0,201	0,346	0,740
CKAP4	0,201	0,167	0,584
SMARCA2	0,201	0,092	0,482
JADE2	0,201	0,352	0,744
ANO6	0,201	0,327	0,726
ARID5B	0,200	0,535	0,850
CD81	0,200	0,337	0,732
GALNT18	0,200	0,201	0,624
ZBTB1	0,200	0,172	0,590
GNL3L	0,200	0,323	0,723
DNAJB2	0,200	0,254	0,667
EDIL3	0,200	0,666	0,906
SLC26A9	0,200	0,472	0,816
RPRD2	0,200	0,211	0,632
CELSR2	0,200	0,614	0,887
OSGEP	0,200	0,193	0,617
MCM7	0,199	0,320	0,721
SPTY2D1	0,199	0,169	0,587
DOCK6	0,199	0,423	0,789
OTUD7B	0,199	0,177	0,596
CTPS2	0,199	0,208	0,629
MTHFD1	0,198	0,212	0,633
ANKRD52	0,198	0,444	0,798
ZNF263	0,198	0,239	0,657
SNRNP70	0,198	0,253	0,666
MAPKBP1	0,198	0,437	0,796
TOR3A	0,198	0,323	0,723
PARM1	0,198	0,414	0,783
TMEM45A	0,197	0,441	0,797

RYBP	0,197	0,220	0,642
SAT1	0,197	0,442	0,798
SMARCD2	0,197	0,437	0,796
CAND1	0,197	0,267	0,679
IFNAR1	0,197	0,300	0,710
ARFGAP2	0,197	0,198	0,622
PIP4K2B	0,197	0,189	0,614
SHQ1	0,197	0,151	0,562
MAGI3	0,197	0,226	0,646
RPL37	0,197	0,398	0,774
ZNF429	0,197	0,304	0,712
ZSCAN30	0,197	0,310	0,715
GTF2I	0,197	0,181	0,602
AZIN1	0,196	0,329	0,727
ANKFY1	0,196	0,226	0,646
SEMA6D	0,196	0,190	0,614
NDN	0,196	0,185	0,608
CTIF	0,195	0,375	0,758
FRK	0,195	0,274	0,687
PRNP	0,195	0,235	0,655
SIRT1	0,195	0,177	0,597
WDR44	0,195	0,138	0,546
C20orf194	0,195	0,276	0,689
SNX17	0,195	0,161	0,577
ADAMTSL3	0,195	0,402	0,774
AKT3	0,195	0,182	0,603
RBM15B	0,194	0,258	0,673
SIN3A	0,194	0,259	0,673
FAM53B	0,194	0,382	0,763
TMEM159	0,194	0,224	0,646
PCF11	0,194	0,163	0,578
LMO4	0,194	0,326	0,725
POLR3C	0,194	0,228	0,648
ZNF592	0,194	0,356	0,746
CCP110	0,194	0,231	0,651
TENT4B	0,194	0,220	0,643
ADGRA3	0,193	0,087	0,475
MARF1	0,193	0,219	0,641
ERLIN2	0,193	0,169	0,587
CTNND1	0,193	0,250	0,664
SNX29	0,192	0,401	0,774
CTDSP2	0,192	0,345	0,740
ARHGEF10L	0,192	0,339	0,735
CCNDBP1	0,192	0,081	0,464
PRRC1	0,192	0,265	0,678
HECTD2	0,192	0,290	0,701
ZNF362	0,192	0,311	0,715
EEF1B2	0,192	0,362	0,751
TRIM28	0,192	0,391	0,768
CAVIN4	0,191	0,529	0,846
NUP50	0,191	0,134	0,540
NOMO3	0,191	0,440	0,797
BLMH	0,191	0,190	0,614

MEGF8	0,191	0,424	0,789
KLC4	0,191	0,301	0,711
EML4	0,191	0,353	0,744
MSN	0,191	0,265	0,679
SUPT16H	0,190	0,051	0,388
NBPF11	0,190	0,278	0,690
VPS39	0,190	0,234	0,655
AHCYL1	0,190	0,246	0,661
ARID4B	0,189	0,100	0,489
ZNF728	0,189	0,344	0,739
SRGAP2	0,189	0,428	0,792
F11R	0,189	0,417	0,784
DHCR24	0,189	0,680	0,911
USP5	0,189	0,305	0,713
POLE	0,189	0,460	0,810
MAGI1	0,189	0,234	0,655
GNG7	0,188	0,448	0,802
HEG1	0,188	0,401	0,774
FGGY	0,188	0,351	0,744
SWT1	0,188	0,383	0,764
SMS	0,188	0,225	0,646
ZCCHC7	0,188	0,286	0,699
FLNC	0,188	0,678	0,911
ZNF626	0,188	0,311	0,715
FAM118A	0,188	0,489	0,824
TMEM135	0,188	0,410	0,782
FAM214A	0,188	0,267	0,680
VANGL1	0,187	0,326	0,725
GOLGA8O	0,187	0,310	0,715
NONO	0,187	0,190	0,614
GNAI1	0,187	0,464	0,812
LGALSL	0,187	0,171	0,588
ARCN1	0,187	0,059	0,414
RP2	0,187	0,396	0,773
DGKH	0,187	0,386	0,766
ERMP1	0,187	0,357	0,747
TMEM131L	0,186	0,178	0,599
NBPF12	0,186	0,405	0,777
BAZ2A	0,186	0,480	0,819
IRS1	0,186	0,298	0,709
WDR27	0,186	0,307	0,714
CDK14	0,186	0,260	0,674
TMEM263	0,186	0,295	0,706
PBX2	0,186	0,315	0,718
CSNK2A2	0,186	0,277	0,689
TRIM45	0,185	0,412	0,782
CTDSP1	0,185	0,310	0,715
CHST15	0,185	0,537	0,850
TMPO	0,185	0,339	0,735
TMF1	0,185	0,203	0,625
OXCT1	0,185	0,490	0,824
ZNF737	0,185	0,296	0,707
CYFIP1	0,185	0,337	0,732

STX5	0,184	0,286	0,699
DELE1	0,184	0,269	0,682
ZNF460	0,184	0,239	0,657
GEMIN4	0,184	0,369	0,756
GATAD2B	0,183	0,457	0,808
SNED1	0,183	0,601	0,883
RRP12	0,183	0,465	0,812
UVRAG	0,183	0,205	0,625
SORL1	0,183	0,462	0,811
DPY19L1	0,183	0,305	0,713
ZNF217	0,183	0,458	0,809
RPS6KA2	0,182	0,426	0,791
ZC3H13	0,182	0,052	0,388
HS1BP3	0,182	0,362	0,751
CCDC18	0,182	0,294	0,705
KIAA0825	0,182	0,311	0,716
TCF12	0,182	0,150	0,562
PTPN9	0,182	0,259	0,673
RCBTB2	0,181	0,251	0,665
MAP3K8	0,181	0,616	0,887
RNF217	0,181	0,410	0,782
SFT2D2	0,181	0,337	0,732
CBL	0,181	0,357	0,747
PARVA	0,181	0,330	0,729
LSM14A	0,181	0,066	0,432
RPS5	0,180	0,498	0,828
IL1RAP	0,180	0,398	0,774
RPS18	0,180	0,486	0,822
TXNIP	0,180	0,391	0,768
TTC12	0,180	0,340	0,736
ERAP1	0,180	0,278	0,690
JUND	0,180	0,332	0,730
AFF4	0,180	0,193	0,617
PACSIN3	0,180	0,497	0,827
ODF2L	0,180	0,364	0,753
GBF1	0,179	0,387	0,766
RPS4X	0,179	0,366	0,755
UHRF1BP1L	0,179	0,261	0,675
TPCN1	0,179	0,513	0,837
PI4KB	0,179	0,320	0,721
OFD1	0,179	0,183	0,606
RPL5	0,179	0,436	0,796
RAPGEF5	0,179	0,380	0,761
ITGA1	0,179	0,457	0,808
STIM2	0,179	0,380	0,761
FAM160B1	0,179	0,257	0,670
ABCB10	0,179	0,236	0,655
BCAS3	0,178	0,198	0,622
MBD1	0,178	0,210	0,631
RPS11	0,178	0,425	0,791
SLC2A3	0,178	0,647	0,899
ZNF426	0,178	0,096	0,484
IFT27	0,178	0,395	0,771

KHNYN	0,178	0,363	0,753
MSR1	0,178	0,549	0,856
HIST1H4D	0,177	0,656	0,900
TBCK	0,177	0,218	0,638
HIST1H4C	0,177	0,505	0,832
RORA	0,177	0,361	0,750
TOP3A	0,177	0,357	0,747
RECQL	0,177	0,385	0,764
VPS52	0,177	0,273	0,685
VAMP2	0,177	0,344	0,739
CRY2	0,177	0,388	0,767
JUP	0,176	0,412	0,782
DYNC1H1	0,176	0,381	0,761
SPEN	0,176	0,394	0,771
TMED4	0,176	0,107	0,501
HDAC9	0,176	0,399	0,774
RNF213	0,176	0,493	0,826
NSD3	0,176	0,189	0,613
NGRN	0,176	0,163	0,578
PXDNL	0,176	0,554	0,858
RPS20	0,176	0,477	0,819
MCFD2	0,175	0,263	0,676
SLC25A44	0,175	0,404	0,776
PML	0,175	0,384	0,764
PARP9	0,175	0,348	0,742
TMEM50A	0,175	0,198	0,622
METTL8	0,175	0,279	0,691
CCZ1	0,175	0,281	0,692
DCUN1D2	0,174	0,528	0,845
RPSA	0,174	0,444	0,798
SND1	0,174	0,288	0,700
CASC3	0,174	0,273	0,685
LCA5	0,174	0,404	0,776
EFCAB14	0,174	0,111	0,506
PTBP3	0,174	0,436	0,796
TPRG1L	0,174	0,230	0,651
ARMCX6	0,174	0,269	0,682
SLC22A5	0,174	0,409	0,781
CFAP44	0,174	0,384	0,764
N4BP2	0,174	0,309	0,715
DGKZ	0,173	0,373	0,756
PXYLP1	0,173	0,395	0,771
HDAC6	0,173	0,335	0,732
MPDZ	0,173	0,200	0,624
ZNF417	0,173	0,161	0,577
POFUT1	0,173	0,239	0,657
TAPBP	0,173	0,412	0,782
PARP4	0,173	0,155	0,567
TRAPPC2L	0,173	0,273	0,685
GPRIN3	0,173	0,359	0,749
SYNRG	0,173	0,153	0,565
UPF3A	0,173	0,170	0,588
ATP6V0A1	0,172	0,228	0,648

TBC1D5	0,172	0,242	0,659
RPL29	0,172	0,387	0,766
DNAJC6	0,172	0,444	0,798
TMED5	0,172	0,425	0,791
TNS2	0,172	0,480	0,819
QSER1	0,172	0,162	0,577
ATP6AP2	0,172	0,291	0,703
GOLPH3L	0,172	0,228	0,648
SCLT1	0,172	0,445	0,799
GATAD1	0,172	0,232	0,653
COL4A4	0,172	0,600	0,882
FAM120C	0,172	0,299	0,710
PSMB8	0,172	0,516	0,838
CTR9	0,171	0,119	0,518
FECH	0,171	0,291	0,703
TENT4A	0,171	0,303	0,712
DDB1	0,171	0,165	0,581
HSP90AB1	0,171	0,325	0,724
FCGRT	0,171	0,463	0,812
TNRC6B	0,171	0,437	0,796
PRKDC	0,170	0,208	0,629
RPLP2	0,170	0,515	0,837
CD2BP2	0,170	0,337	0,732
HUWE1	0,170	0,431	0,794
CYP2J2	0,170	0,622	0,888
IVD	0,170	0,341	0,736
PTPN3	0,170	0,351	0,744
BTBD8	0,170	0,317	0,720
TBC1D20	0,170	0,251	0,665
DGCR2	0,170	0,378	0,761
GAK	0,170	0,389	0,767
ZBTB37	0,169	0,304	0,712
ZNF841	0,169	0,233	0,654
STK17A	0,169	0,268	0,681
SUDS3	0,169	0,090	0,481
KLHL36	0,169	0,335	0,732
PSME1	0,168	0,244	0,661
NR2F2	0,168	0,388	0,767
ZNF608	0,168	0,373	0,756
CEP120	0,168	0,135	0,542
PRRC2A	0,168	0,538	0,850
SEC62	0,168	0,332	0,730
LIMD1	0,168	0,371	0,756
SAP30L	0,168	0,176	0,596
WDPCP	0,168	0,227	0,647
BRD1	0,168	0,370	0,756
NEK3	0,168	0,344	0,739
RBBP9	0,168	0,353	0,744
WDTC1	0,168	0,493	0,826
GRB2	0,168	0,225	0,646
SSBP2	0,167	0,137	0,546
HOMER1	0,167	0,711	0,923
TMEM237	0,167	0,253	0,666

HFM1	0,167	0,624	0,889
ZFC3H1	0,167	0,391	0,769
INTS2	0,166	0,169	0,587
ZFYVE26	0,166	0,353	0,744
BTRC	0,166	0,201	0,624
ZNF251	0,166	0,369	0,756
ACBD3	0,166	0,249	0,662
CAMKK2	0,166	0,372	0,756
PPP2R3B	0,166	0,444	0,798
C12orf49	0,166	0,329	0,728
GCN1	0,166	0,418	0,784
PRMT7	0,165	0,388	0,767
RIPOR1	0,165	0,468	0,813
SP1	0,165	0,346	0,740
ZNF516	0,165	0,365	0,754
SPRED1	0,165	0,380	0,761
RPL28	0,165	0,463	0,812
UBE4B	0,165	0,413	0,782
POLI	0,165	0,255	0,668
MTMR1	0,165	0,241	0,659
SEPTIN10	0,165	0,296	0,706
MAN2C1	0,164	0,448	0,802
ZNF568	0,164	0,304	0,712
NCBP3	0,164	0,282	0,693
RPL34	0,164	0,514	0,837
DCTN1	0,164	0,378	0,761
ACAD8	0,164	0,322	0,723
NFATC3	0,164	0,283	0,695
PURB	0,163	0,227	0,647
RBL2	0,163	0,140	0,549
CBX7	0,163	0,365	0,754
GPX1	0,163	0,441	0,797
ADAM22	0,163	0,375	0,757
SPAG9	0,163	0,191	0,616
MEF2D	0,162	0,465	0,812
PLEKHA8	0,162	0,339	0,736
WBP2	0,162	0,313	0,717
TBC1D22A	0,162	0,404	0,776
TRIQK	0,162	0,526	0,844
NCL	0,162	0,188	0,613
CEP126	0,162	0,390	0,768
ING4	0,162	0,319	0,721
CRPPA	0,162	0,506	0,832
FYB1	0,161	0,604	0,883
CERK	0,161	0,208	0,629
PRMT2	0,161	0,161	0,577
FBXO7	0,161	0,124	0,525
DYNLT1	0,161	0,244	0,660
RPL41	0,161	0,471	0,816
ANXA2	0,161	0,572	0,867
SPATA7	0,161	0,327	0,726
DCP2	0,161	0,348	0,742
BNC2	0,161	0,680	0,911

MSI2	0,160	0,353	0,744
TRAK2	0,160	0,426	0,791
SEC63	0,160	0,206	0,627
SEC24D	0,160	0,445	0,799
MEX3C	0,160	0,292	0,703
LMO7	0,160	0,511	0,836
FSTL3	0,160	0,630	0,892
PDGFA	0,160	0,322	0,723
TSKU	0,160	0,729	0,930
RAB2B	0,159	0,248	0,661
SH3BGRL2	0,159	0,421	0,788
ALG13	0,159	0,276	0,688
IFT172	0,159	0,330	0,729
LYST	0,159	0,389	0,768
PTPN11	0,159	0,320	0,721
IMPACT	0,159	0,320	0,721
ACSL5	0,159	0,458	0,809
CHP1	0,158	0,253	0,666
EEA1	0,158	0,305	0,713
COX6A1	0,158	0,449	0,802
GPRC5A	0,158	0,804	0,951
ARMCX1	0,158	0,204	0,625
SCYL2	0,158	0,385	0,764
MIB1	0,158	0,396	0,772
SLC35C2	0,158	0,371	0,756
EIF2S3	0,158	0,288	0,700
AMBRA1	0,157	0,366	0,755
SLC35E3	0,157	0,435	0,796
EPAS1	0,157	0,435	0,795
CSAD	0,157	0,477	0,819
RGS12	0,157	0,467	0,813
BTG2	0,157	0,688	0,914
VPS4B	0,157	0,254	0,667
TERF2	0,157	0,368	0,756
SHISA5	0,156	0,453	0,806
CHAMP1	0,156	0,467	0,813
WDR37	0,156	0,291	0,702
NPIPB11	0,156	0,517	0,838
NOTCH2NLC	0,156	0,462	0,811
RALBP1	0,156	0,095	0,484
PLPP5	0,156	0,364	0,753
GSTA4	0,156	0,400	0,774
MINK1	0,156	0,467	0,813
ITGB1BP1	0,156	0,583	0,872
KANTR	0,156	0,439	0,797
FBL	0,155	0,488	0,823
UBA52	0,155	0,414	0,783
KLHL20	0,155	0,241	0,659
TRRAP	0,155	0,505	0,832
TIE1	0,155	0,479	0,819
PUM1	0,155	0,184	0,606
ZNF652	0,155	0,326	0,725
PLBD1	0,155	0,324	0,724

SERTAD3	0,155	0,419	0,785
RAB33B	0,154	0,266	0,679
PRKCSH	0,154	0,463	0,812
TARBP1	0,154	0,297	0,708
MYH14	0,154	0,486	0,822
R3HDM2	0,154	0,442	0,797
TMOD2	0,154	0,490	0,824
ATMIN	0,154	0,111	0,506
ATN1	0,154	0,548	0,855
LPIN2	0,154	0,442	0,797
SYNGR2	0,154	0,307	0,714
JAM3	0,154	0,305	0,713
ZNF891	0,154	0,376	0,758
MAPK1	0,154	0,095	0,484
RPL35	0,154	0,544	0,855
LIN7C	0,154	0,432	0,794
LIMS1	0,153	0,337	0,732
TBL1X	0,153	0,479	0,819
DENND10	0,153	0,229	0,649
DHX16	0,153	0,353	0,744
GALNT10	0,153	0,407	0,778
RPL4	0,153	0,500	0,829
WBP11	0,153	0,220	0,643
RPA2	0,153	0,240	0,658
ERCC6	0,153	0,230	0,649
SPIN1	0,153	0,118	0,517
HNRNPUL1	0,153	0,472	0,816
RPS23	0,153	0,552	0,856
GALK2	0,153	0,283	0,695
CREB5	0,153	0,614	0,887
CHD3	0,153	0,551	0,856
DCAF16	0,153	0,244	0,660
RBBP4	0,152	0,191	0,616
ZNF266	0,152	0,370	0,756
PGR	0,152	0,311	0,715
CANX	0,152	0,336	0,732
BRD8	0,152	0,225	0,646
CEP290	0,152	0,400	0,774
KALRN	0,152	0,346	0,740
CTCF	0,151	0,246	0,661
RBSN	0,151	0,140	0,547
FGFR1OP	0,151	0,440	0,797
CC2D2A	0,151	0,335	0,732
SKA2	0,151	0,367	0,755
ACSF2	0,151	0,528	0,845
RPL22	0,151	0,488	0,823
KCTD7	0,151	0,374	0,756
GK5	0,151	0,419	0,785
TANC1	0,151	0,298	0,709
AMMECR1L	0,151	0,351	0,744
FUT11	0,151	0,353	0,744
DHX40	0,151	0,345	0,739
MLXIP	0,151	0,556	0,858

GOLIM4	0,151	0,260	0,674
ASB7	0,151	0,412	0,782
SOCS5	0,151	0,225	0,646
DIP2A	0,151	0,407	0,779
BCL9	0,151	0,533	0,848
FRMD4B	0,151	0,491	0,825
PPP4R1	0,151	0,241	0,659
LYZ	0,151	0,689	0,914
ATG14	0,150	0,304	0,712
RAB14	0,150	0,221	0,644
XPO7	0,150	0,163	0,578
WDR19	0,150	0,464	0,812
LRRC57	0,150	0,434	0,795
SF3B3	0,150	0,249	0,662
TCF20	0,150	0,472	0,816
CHST10	0,150	0,493	0,826
AHNAK2	0,150	0,678	0,911
ARNT	0,150	0,278	0,690
PPP5C	0,150	0,332	0,730
KANSL3	0,150	0,343	0,738
C12orf45	0,150	0,339	0,735
YTHDC1	0,150	0,084	0,472
MYOM1	0,150	0,674	0,908
CRYL1	0,150	0,392	0,769
MXRA7	0,150	0,315	0,719
NCKAP5	0,149	0,519	0,840
ANKRD36	0,149	0,384	0,764
NPIPB5	0,149	0,552	0,856
CHID1	0,149	0,434	0,795
CAPN2	0,149	0,310	0,715
CSF1	0,148	0,607	0,885
ANP32B	0,148	0,384	0,764
MDC1	0,148	0,572	0,867
SOS1	0,148	0,347	0,740
CA13	0,148	0,410	0,781
ACTR1B	0,148	0,333	0,731
ADIPOR2	0,148	0,383	0,764
ZNF846	0,148	0,456	0,807
USP6NL	0,148	0,255	0,668
KAT14	0,148	0,422	0,789
RNF169	0,148	0,289	0,701
ZNF304	0,148	0,372	0,756
POF1B	0,147	0,634	0,893
GPN2	0,147	0,461	0,810
NID2	0,147	0,517	0,838
VIRMA	0,147	0,396	0,772
MPP7	0,147	0,374	0,757
EXOSC1	0,147	0,399	0,774
RBCK1	0,147	0,376	0,758
TSPAN6	0,146	0,490	0,824
MEAF6	0,146	0,187	0,612
KLHDC10	0,146	0,201	0,624
PLXNA2	0,146	0,648	0,899

CELF1	0,146	0,361	0,750
HSPA4L	0,146	0,505	0,832
YEATS2	0,146	0,281	0,692
DCLRE1C	0,146	0,373	0,756
SF3B2	0,146	0,419	0,785
C8orf33	0,146	0,297	0,708
LRCH3	0,146	0,239	0,657
POGLUT3	0,146	0,404	0,776
ZNF557	0,146	0,421	0,788
POLDIP3	0,146	0,414	0,784
CARNMT1	0,146	0,455	0,806
ZNF543	0,146	0,502	0,830
AKAP12	0,146	0,608	0,885
VPS18	0,146	0,458	0,809
OS9	0,145	0,326	0,725
FAT1	0,145	0,392	0,769
SAFB2	0,145	0,325	0,724
GUK1	0,145	0,465	0,812
GM2A	0,145	0,259	0,673
RIOK3	0,145	0,456	0,807
USP54	0,145	0,554	0,858
ANGEL1	0,145	0,493	0,826
STAU1	0,145	0,208	0,629
PLEKHA3	0,144	0,206	0,627
RNF214	0,144	0,459	0,809
RAPGEF6	0,144	0,432	0,794
MOGS	0,144	0,441	0,797
ZNF470	0,144	0,327	0,726
SMARCA5	0,144	0,310	0,715
ADGRL3	0,144	0,349	0,743
MAPRE2	0,144	0,373	0,756
SRPK2	0,144	0,166	0,584
CFAP36	0,144	0,362	0,751
SURF4	0,144	0,290	0,701
PXDN	0,144	0,629	0,891
PHF2	0,143	0,489	0,824
EML3	0,143	0,557	0,858
ANKRD33B	0,143	0,649	0,899
PLXNB1	0,143	0,496	0,827
BMI1	0,143	0,413	0,782
CENPC	0,143	0,411	0,782
APH1B	0,143	0,353	0,744
SLC44A1	0,142	0,451	0,804
CRK	0,142	0,247	0,661
CXorf56	0,142	0,343	0,738
ARPC5	0,142	0,527	0,844
SOS2	0,142	0,263	0,676
MSRB3	0,142	0,197	0,621
SLC45A4	0,142	0,397	0,773
CHD2	0,142	0,307	0,714
KLHDC1	0,142	0,351	0,744
ITM2B	0,142	0,416	0,784
C14orf180	0,142	0,589	0,875

RALA	0,142	0,288	0,700
EBF1	0,142	0,653	0,899
ZNF532	0,141	0,287	0,699
BARD1	0,141	0,534	0,849
COQ4	0,141	0,450	0,803
HDLBP	0,141	0,292	0,703
SLN	0,141	0,729	0,930
HIST2H2AC	0,141	0,578	0,870
SLBP	0,141	0,396	0,772
DIDO1	0,141	0,341	0,737
SUSD6	0,141	0,471	0,816
CORO1C	0,141	0,370	0,756
UBR7	0,141	0,266	0,679
B4GAT1	0,140	0,433	0,795
NHSL2	0,140	0,544	0,855
MYH9	0,140	0,557	0,858
ZNF175	0,140	0,396	0,772
FCHSD2	0,140	0,284	0,696
EFCAB13	0,140	0,458	0,809
MAPK3	0,140	0,433	0,795
SLF2	0,140	0,377	0,760
ATF7IP	0,140	0,210	0,631
PLPBP	0,140	0,163	0,578
CROT	0,140	0,390	0,768
ENPP1	0,140	0,430	0,793
HELZ	0,139	0,203	0,625
PLOD2	0,139	0,681	0,912
WHAMM	0,139	0,465	0,812
RBMS2	0,139	0,479	0,819
MLYCD	0,139	0,629	0,891
MAP4K5	0,139	0,432	0,794
NEK9	0,139	0,343	0,739
GTF2A1	0,139	0,311	0,716
SSR2	0,139	0,472	0,816
RBM20	0,139	0,680	0,911
TBC1D32	0,139	0,479	0,819
FAM110B	0,139	0,406	0,777
RPL27A	0,139	0,454	0,806
B4GALT1	0,138	0,578	0,870
SSR1	0,138	0,352	0,744
ADCY5	0,138	0,630	0,892
ELP2	0,138	0,242	0,659
NCAPD3	0,138	0,454	0,806
PAN3	0,138	0,330	0,729
SEC24C	0,138	0,524	0,842
MSL2	0,138	0,341	0,736
ARMCX4	0,137	0,433	0,795
SFMBT2	0,137	0,315	0,719
POLR2G	0,137	0,360	0,750
QTRT2	0,137	0,443	0,798
JCAD	0,137	0,444	0,799
DOCK2	0,137	0,618	0,887
SMCR8	0,137	0,549	0,856

METTL16	0,137	0,355	0,745
JPT2	0,137	0,345	0,740
SH3GLB2	0,137	0,415	0,784
PRPF8	0,137	0,515	0,837
TAP1	0,136	0,530	0,846
RASA3	0,136	0,556	0,858
ZNF318	0,136	0,477	0,819
NOMO2	0,136	0,521	0,841
PRKD3	0,136	0,385	0,765
LRRC41	0,136	0,410	0,782
FNIP2	0,136	0,306	0,713
DHDDS	0,136	0,398	0,774
ALMS1	0,135	0,250	0,663
PHRF1	0,135	0,469	0,815
BRD2	0,135	0,391	0,769
LINS1	0,135	0,428	0,792
ZDHHC3	0,135	0,234	0,655
GOLGA8B	0,135	0,493	0,826
FIGNL1	0,135	0,467	0,813
EXOC4	0,134	0,208	0,629
AGBL5	0,134	0,496	0,827
KIF16B	0,134	0,286	0,699
ANKZF1	0,134	0,390	0,768
EXOC6B	0,134	0,465	0,812
CCNI	0,134	0,382	0,762
IGF1R	0,134	0,615	0,887
FTO	0,134	0,170	0,588
NUB1	0,133	0,193	0,617
PTPRC	0,133	0,680	0,911
PGRMC1	0,133	0,602	0,883
RBMX	0,133	0,307	0,714
TFDP2	0,133	0,225	0,646
ATXN2L	0,133	0,604	0,884
SF3A1	0,133	0,523	0,842
AKR1C2	0,133	0,786	0,948
ASH1L	0,133	0,208	0,629
GMPS	0,133	0,238	0,657
PRR14L	0,133	0,247	0,661
DNAJA4	0,133	0,609	0,885
VPS41	0,133	0,246	0,661
PFKP	0,133	0,468	0,813
FAM122B	0,133	0,347	0,742
RNASEH1	0,133	0,366	0,755
VPS35L	0,132	0,265	0,678
U2AF2	0,132	0,426	0,791
TMEM64	0,132	0,465	0,812
KRBOX4	0,132	0,489	0,824
GCC2	0,132	0,303	0,712
SRRM1	0,132	0,353	0,744
PPP2R1A	0,132	0,526	0,844
PSIP1	0,132	0,261	0,675
UTY	0,132	0,770	0,943
UBE2E2	0,132	0,402	0,775

HPS1	0,132	0,455	0,807
HSPH1	0,132	0,623	0,889
ZMYM1	0,132	0,395	0,771
MYEF2	0,132	0,466	0,812
SELENOF	0,132	0,517	0,838
RACK1	0,132	0,576	0,870
AHR	0,132	0,653	0,899
ELOA	0,131	0,317	0,720
B3GNT2	0,131	0,444	0,799
ASXL2	0,131	0,350	0,744
PI4K2A	0,131	0,465	0,812
FYCO1	0,131	0,606	0,885
RGP1	0,131	0,492	0,825
GTF3C1	0,131	0,482	0,821
CSPG4	0,131	0,713	0,924
ZNF14	0,131	0,468	0,814
ZNF570	0,131	0,375	0,758
REEP3	0,131	0,508	0,833
VPS11	0,131	0,358	0,747
SYT11	0,130	0,504	0,832
RHOBTB2	0,130	0,623	0,889
UMPS	0,130	0,453	0,806
CLNS1A	0,130	0,251	0,665
ATP9B	0,130	0,261	0,676
SYK	0,130	0,608	0,885
SNIP1	0,130	0,460	0,810
SAFB	0,130	0,380	0,761
NOSTRIN	0,130	0,374	0,757
RABGAP1	0,130	0,180	0,600
KLF3	0,130	0,522	0,842
DCK	0,130	0,508	0,833
TMEM260	0,130	0,428	0,792
CAP1	0,130	0,555	0,858
YLPM1	0,129	0,408	0,780
MAPK6	0,129	0,649	0,899
IRF2	0,129	0,420	0,787
KLHL15	0,129	0,486	0,822
ZNF28	0,129	0,424	0,789
TTF2	0,129	0,458	0,809
LRP10	0,129	0,502	0,830
BOD1L1	0,129	0,258	0,672
RXRA	0,129	0,585	0,872
BBX	0,129	0,195	0,618
FOXO3	0,129	0,467	0,813
DDX23	0,129	0,388	0,767
AKT2	0,128	0,500	0,829
SETD2	0,128	0,213	0,634
AKT1	0,128	0,555	0,858
BTAF1	0,128	0,506	0,832
ZMIZ1	0,127	0,649	0,899
HSPA12A	0,127	0,527	0,844
DIP2B	0,127	0,286	0,699
CPEB3	0,127	0,515	0,837

MAN2B1	0,127	0,593	0,879
GTF3C4	0,127	0,358	0,748
ST13	0,127	0,340	0,736
C2orf49	0,127	0,348	0,743
MKRN1	0,127	0,306	0,713
RPL23A	0,127	0,603	0,883
GTF2F1	0,127	0,486	0,822
LARP1	0,127	0,596	0,880
GPR161	0,127	0,550	0,856
PHC3	0,127	0,349	0,743
PACS2	0,127	0,515	0,837
CD302	0,127	0,550	0,856
PPFIA1	0,126	0,353	0,744
KDELR1	0,126	0,468	0,814
MIS12	0,126	0,421	0,788
UBA1	0,126	0,461	0,811
HIPK2	0,126	0,710	0,923
NRP1	0,126	0,464	0,812
DGKD	0,126	0,622	0,888
GOSR1	0,126	0,282	0,693
TNS1	0,126	0,698	0,918
SPAST	0,125	0,416	0,784
FBXO9	0,125	0,298	0,709
TNKS1BP1	0,125	0,634	0,893
UFM1	0,125	0,569	0,865
PUS10	0,125	0,480	0,819
AIF1	0,125	0,616	0,887
CDH5	0,125	0,541	0,853
PLPP1	0,125	0,472	0,816
HMGCL	0,125	0,372	0,756
PCBP2	0,124	0,357	0,747
RPS6KA3	0,124	0,578	0,870
ADSS	0,124	0,526	0,844
CAAP1	0,124	0,446	0,800
TMEM167B	0,124	0,421	0,788
SIRPA	0,124	0,568	0,864
ZMYND11	0,124	0,192	0,616
CYFIP2	0,124	0,428	0,792
MAN2A1	0,124	0,510	0,835
MAOB	0,124	0,504	0,832
TOMM20	0,124	0,214	0,634
MAN1A2	0,124	0,478	0,819
NET1	0,124	0,639	0,894
DDX58	0,124	0,478	0,819
CC2D1B	0,124	0,492	0,826
CERT1	0,123	0,354	0,744
PPWD1	0,123	0,401	0,774
KDM2A	0,123	0,512	0,837
AP3M1	0,123	0,235	0,655
INPPL1	0,123	0,561	0,860
ZNF107	0,123	0,461	0,811
COG3	0,123	0,323	0,724
RAD54L2	0,123	0,548	0,855

ZNF117	0,123	0,689	0,914
MINDY3	0,123	0,535	0,850
MBIP	0,123	0,442	0,797
TASOR	0,123	0,417	0,784
FAM53C	0,123	0,438	0,797
NBN	0,123	0,379	0,761
PPIL4	0,123	0,409	0,781
STK35	0,123	0,571	0,867
CDC42SE1	0,122	0,487	0,823
FKBP9	0,122	0,464	0,812
ZNF76	0,122	0,515	0,837
VCAM1	0,122	0,744	0,934
FBXO38	0,122	0,247	0,661
ARGLU1	0,122	0,457	0,808
EPC2	0,122	0,216	0,637
YPEL5	0,122	0,455	0,806
AMOTL1	0,122	0,419	0,786
ANKLE2	0,122	0,461	0,811
SEC22C	0,121	0,311	0,715
SMAD4	0,121	0,320	0,721
DDX3Y	0,121	0,813	0,954
CMTM6	0,121	0,505	0,832
CDK5RAP2	0,121	0,357	0,747
ABI3BP	0,121	0,604	0,883
MPP5	0,121	0,392	0,769
UBQLN2	0,121	0,464	0,812
RPS16	0,121	0,594	0,879
RPL38	0,121	0,455	0,806
NSD2	0,121	0,384	0,764
SLC39A9	0,121	0,416	0,784
FKBP8	0,121	0,571	0,867
HDAC3	0,120	0,494	0,826
LIN54	0,120	0,435	0,795
EIF3A	0,120	0,231	0,651
TEX10	0,120	0,468	0,813
SCN1B	0,120	0,387	0,766
PHF12	0,120	0,618	0,887
PHF19	0,120	0,563	0,861
NOTCH3	0,120	0,652	0,899
S100A10	0,120	0,682	0,912
HACD4	0,120	0,485	0,822
ZNF160	0,120	0,396	0,772
INVS	0,120	0,460	0,810
BPTF	0,120	0,373	0,756
PSAP	0,119	0,486	0,822
USF2	0,119	0,409	0,781
PYGO1	0,119	0,500	0,830
WDR5	0,119	0,524	0,842
EPHX1	0,119	0,745	0,935
ZCCHC8	0,119	0,425	0,791
SH3BP5L	0,119	0,532	0,847
ALDH1A1	0,119	0,741	0,933
TBC1D16	0,119	0,704	0,921

SCARB1	0,119	0,668	0,907
NDFIP1	0,119	0,292	0,703
C9orf78	0,119	0,369	0,756
TMEM219	0,119	0,508	0,833
CORIN	0,119	0,708	0,923
ATXN7L3	0,118	0,524	0,842
TRIM26	0,118	0,513	0,837
NADSYN1	0,118	0,467	0,813
EIF3G	0,118	0,496	0,827
POM121C	0,118	0,639	0,894
COX15	0,118	0,355	0,745
AGO1	0,118	0,545	0,855
ZNF397	0,118	0,295	0,705
TANGO6	0,117	0,548	0,856
CHD8	0,117	0,422	0,789
YY1	0,117	0,350	0,744
FGD6	0,117	0,497	0,827
OSBPL8	0,117	0,607	0,885
NPHP3	0,117	0,498	0,828
ATXN3	0,117	0,348	0,742
IQGAP1	0,117	0,573	0,867
FLNA	0,117	0,670	0,907
SERINC5	0,117	0,449	0,802
BROX	0,117	0,419	0,786
NPC2	0,117	0,506	0,832
GPX4	0,117	0,636	0,893
TUG1	0,117	0,304	0,712
ELF1	0,117	0,479	0,819
TLN2	0,117	0,670	0,907
APBB2	0,116	0,367	0,755
ZNHIT6	0,116	0,375	0,757
PWWP2A	0,116	0,407	0,779
LIG3	0,116	0,536	0,850
IFT46	0,116	0,471	0,816
HNRNPLL	0,116	0,456	0,807
TMEM68	0,115	0,514	0,837
MTR	0,115	0,385	0,764
MFSD14B	0,115	0,371	0,756
UBE2O	0,115	0,605	0,884
HNRNPA0	0,115	0,316	0,720
REPS1	0,115	0,249	0,662
SRSF9	0,115	0,521	0,841
PTBP1	0,115	0,552	0,856
FOXO4	0,115	0,565	0,862
USP46	0,115	0,601	0,883
TRA2A	0,115	0,391	0,769
BZW1	0,115	0,652	0,899
NUFIP2	0,115	0,451	0,804
CDKN2C	0,114	0,556	0,858
DCAF17	0,114	0,374	0,757
NIPA1	0,114	0,496	0,827
IRF1	0,114	0,661	0,903
ACSL4	0,114	0,590	0,876

BMERB1	0,114	0,447	0,800
MCM9	0,114	0,470	0,815
SIK2	0,114	0,593	0,879
GGA1	0,114	0,561	0,860
GON4L	0,114	0,379	0,761
TNKS	0,114	0,344	0,739
ZNF197	0,114	0,279	0,690
MCM6	0,114	0,463	0,811
EPHX2	0,114	0,534	0,849
ZNF880	0,114	0,423	0,789
ZNF850	0,113	0,554	0,858
PRKAB2	0,113	0,546	0,855
ZNF717	0,113	0,477	0,819
RBL1	0,113	0,568	0,864
SNRPN	0,113	0,657	0,901
ZNF81	0,113	0,446	0,800
GBP4	0,113	0,740	0,933
IPO8	0,113	0,232	0,653
LAMA3	0,113	0,716	0,925
SLC35B3	0,113	0,458	0,809
HECTD3	0,112	0,569	0,865
IL10RA	0,112	0,704	0,921
NT5C2	0,112	0,485	0,822
AP3B1	0,112	0,275	0,687
STYX	0,112	0,490	0,824
MYC	0,112	0,731	0,931
PPP3R1	0,112	0,436	0,796
MRPL49	0,112	0,496	0,827
ANP32E	0,112	0,635	0,893
HPS5	0,112	0,466	0,812
DLG1	0,112	0,428	0,792
COPRS	0,112	0,414	0,783
KLHL13	0,112	0,565	0,862
RHOA	0,112	0,357	0,747
LMNA	0,112	0,652	0,899
NEXN	0,112	0,705	0,921
UBXN6	0,112	0,508	0,833
DHFR	0,111	0,658	0,901
RPS15	0,111	0,582	0,872
PKP2	0,111	0,674	0,908
TRIM44	0,111	0,320	0,721
TFCP2	0,111	0,436	0,796
ZNF621	0,111	0,426	0,791
TUB	0,111	0,608	0,885
GOLGA6L9	0,111	0,601	0,883
CTNNA1	0,111	0,391	0,769
ZFP1	0,111	0,545	0,855
DPH7	0,111	0,571	0,866
PFDN5	0,111	0,553	0,857
MTMR4	0,110	0,475	0,818
RBM10	0,110	0,548	0,856
NPIPA9	0,110	0,555	0,858
ZKSCAN8	0,110	0,278	0,690

CIZ1	0,110	0,516	0,838
SDCCAG8	0,110	0,412	0,782
SECISBP2L	0,110	0,281	0,693
CAPRIN1	0,110	0,264	0,678
C1S	0,110	0,689	0,914
ATRN	0,110	0,322	0,723
FBXO31	0,110	0,544	0,855
IL33	0,110	0,710	0,923
PCNX4	0,110	0,496	0,827
ABTB2	0,110	0,641	0,896
ANKRD11	0,109	0,649	0,899
IVNS1ABP	0,109	0,583	0,872
MEF2A	0,109	0,544	0,855
CEP95	0,109	0,412	0,782
JRK	0,109	0,664	0,905
EAPP	0,109	0,545	0,855
LRRK2	0,109	0,512	0,837
KLF9	0,109	0,378	0,761
ITFG2	0,109	0,612	0,887
SEC22B	0,109	0,478	0,819
TXLNA	0,109	0,555	0,858
SRA1	0,109	0,462	0,811
RNASEL	0,109	0,564	0,862
FBXO11	0,108	0,501	0,830
RESF1	0,108	0,628	0,891
STRIP1	0,108	0,538	0,850
CACNA2D1	0,108	0,577	0,870
CHMP1B	0,108	0,344	0,739
LRRC58	0,108	0,520	0,841
TMEM129	0,108	0,488	0,823
ANKIB1	0,108	0,350	0,744
IFT74	0,108	0,536	0,850
MIS18BP1	0,107	0,565	0,862
TFIP11	0,107	0,551	0,856
BMPR2	0,107	0,436	0,796
RDH13	0,107	0,658	0,901
ZNF287	0,107	0,545	0,855
MED22	0,107	0,608	0,885
TLK1	0,107	0,373	0,756
APPBP2	0,107	0,423	0,789
RTF1	0,107	0,451	0,804
FUS	0,107	0,560	0,859
AAK1	0,107	0,618	0,887
NFATC2IP	0,107	0,573	0,867
ACAP2	0,107	0,524	0,842
CBX1	0,107	0,358	0,748
DHX57	0,107	0,424	0,789
RANBP1	0,107	0,534	0,849
ZNF585A	0,107	0,524	0,843
RAB29	0,107	0,488	0,823
FBXO25	0,107	0,411	0,782
KIAA1671	0,106	0,675	0,908
TMBIM6	0,106	0,460	0,810

STRN4	0,106	0,582	0,872
AK4	0,106	0,503	0,831
PLD3	0,106	0,592	0,878
ITGA9	0,106	0,603	0,883
IQCE	0,106	0,619	0,888
USP9Y	0,105	0,831	0,962
ARL5B	0,105	0,686	0,914
SLFN11	0,105	0,585	0,872
DOCK8	0,105	0,704	0,921
KHDRBS1	0,105	0,332	0,730
CPSF7	0,105	0,537	0,850
LATS1	0,105	0,474	0,817
SENP1	0,105	0,518	0,839
HNRNPK	0,105	0,329	0,727
ARMH4	0,105	0,637	0,893
NOMO1	0,104	0,637	0,893
TBX5	0,104	0,639	0,894
TTC7A	0,104	0,623	0,889
STXBP4	0,104	0,474	0,817
PELI2	0,104	0,642	0,897
SATB1	0,104	0,506	0,832
CPNE1	0,104	0,608	0,885
BRAF	0,104	0,335	0,732
WDR48	0,104	0,484	0,821
SETDB2	0,103	0,484	0,821
ZNF133	0,103	0,565	0,862
AHI1	0,103	0,580	0,871
KBTBD2	0,103	0,506	0,832
LRP1	0,103	0,785	0,947
NMT2	0,103	0,417	0,784
CCDC146	0,103	0,627	0,890
MORC3	0,103	0,699	0,918
ARHGAP15	0,103	0,665	0,905
ZNF585B	0,103	0,454	0,806
LEPROTL1	0,103	0,502	0,830
CHD6	0,103	0,356	0,747
NIPBL	0,103	0,314	0,718
KDSR	0,103	0,372	0,756
MED14	0,103	0,361	0,750
RUFY2	0,102	0,489	0,824
SNAPC1	0,102	0,607	0,885
PIP5K1A	0,102	0,537	0,850
DDHD2	0,102	0,302	0,712
LARS2	0,102	0,561	0,860
RPLP1	0,102	0,637	0,893
NEK1	0,102	0,486	0,822
CPVL	0,102	0,756	0,937
SELENBP1	0,102	0,787	0,948
PIK3CB	0,102	0,327	0,725
ANKRD6	0,102	0,572	0,867
ASCC2	0,102	0,538	0,850
EYA3	0,101	0,481	0,820
SNX19	0,101	0,485	0,822

LEMD2	0,101	0,558	0,858
UBXN2A	0,101	0,350	0,743
PDE4B	0,101	0,729	0,930
SEC61A1	0,101	0,530	0,846
SACS	0,101	0,676	0,909
WWC2	0,101	0,518	0,839
MECP2	0,101	0,636	0,893
ECHDC2	0,101	0,558	0,858
FAM160B2	0,101	0,555	0,858
PRUNE1	0,101	0,560	0,859
DOP1A	0,101	0,485	0,822
RAP1B	0,101	0,593	0,879
MYL12B	0,101	0,607	0,885
PITHD1	0,101	0,422	0,789
ACVR1	0,101	0,510	0,835
CCNL2	0,101	0,480	0,819
PAK1	0,101	0,587	0,875
SRI	0,101	0,463	0,812
POLR2M	0,100	0,602	0,883
FER	0,100	0,602	0,883
PMS2	0,100	0,568	0,864
POLR2C	0,100	0,373	0,756
ZNF561	0,100	0,400	0,774
CIRBP	0,100	0,475	0,818
GUSB	0,100	0,537	0,850
ANKRD13C	0,100	0,533	0,848
ZNF573	0,100	0,617	0,887
GPAM	0,100	0,911	0,983
RSPRY1	0,099	0,402	0,775
CLSTN2	0,099	0,784	0,946
RSBN1	0,099	0,416	0,784
BACH1	0,099	0,631	0,892
RPS13	0,099	0,624	0,889
CAMK2G	0,099	0,570	0,866
ZNF791	0,099	0,352	0,744
FOXO3B	0,099	0,580	0,870
IDH1	0,099	0,785	0,947
KPNA4	0,099	0,520	0,841
ZNF677	0,099	0,570	0,865
PARN	0,099	0,330	0,729
SLC37A3	0,099	0,476	0,818
SLC25A23	0,099	0,676	0,909
ERCC8	0,099	0,542	0,853
RRAS	0,098	0,649	0,899
SOGA1	0,098	0,756	0,938
RAB30	0,098	0,477	0,819
G3BP1	0,098	0,398	0,774
TRAM1	0,098	0,669	0,907
GPNMB	0,098	0,631	0,892
TCF4	0,098	0,557	0,858
NFAT5	0,098	0,580	0,870
EPS8	0,098	0,564	0,862
SYMPK	0,098	0,628	0,891

GOLGA8R	0,098	0,664	0,905
SMURF1	0,098	0,568	0,864
DCAF8	0,098	0,504	0,832
KDM1A	0,097	0,322	0,723
FGFR1OP2	0,097	0,619	0,888
PPIB	0,097	0,635	0,893
HERC4	0,097	0,561	0,860
S100PBP	0,097	0,515	0,837
MAP7D3	0,097	0,333	0,730
PPP3CA	0,097	0,627	0,890
ARID2	0,097	0,361	0,750
DROSHA	0,097	0,440	0,797
MED28	0,097	0,515	0,837
YIPF5	0,097	0,607	0,885
ROCK1	0,097	0,384	0,764
KDM5A	0,097	0,373	0,756
ZNF37A	0,096	0,415	0,784
SRRM2	0,096	0,748	0,936
ACOT11	0,096	0,792	0,948
ZSCAN29	0,096	0,585	0,872
AKR1C1	0,096	0,810	0,954
ZNF445	0,096	0,636	0,893
DSTYK	0,096	0,543	0,854
RBM5	0,096	0,479	0,819
BCL6B	0,096	0,653	0,899
CA5B	0,096	0,644	0,898
MYOCD	0,096	0,689	0,914
NUDT21	0,096	0,596	0,880
RPL32	0,095	0,684	0,913
TMEM144	0,095	0,690	0,915
SRP72	0,095	0,536	0,850
GNB1	0,095	0,471	0,816
FAM120AOS	0,095	0,303	0,712
RPL8	0,095	0,630	0,891
ZNF140	0,095	0,583	0,872
SGK3	0,095	0,483	0,821
SWAP70	0,095	0,454	0,806
RBBP6	0,095	0,483	0,821
ZNF609	0,095	0,613	0,887
MYO9B	0,095	0,658	0,902
GRIPAP1	0,095	0,551	0,856
SFPQ	0,095	0,550	0,856
SULT1A1	0,095	0,659	0,902
RAB8B	0,095	0,724	0,928
FAM120A	0,095	0,384	0,764
MON2	0,095	0,545	0,855
CARS2	0,095	0,480	0,819
CTSD	0,095	0,712	0,923
XPO1	0,095	0,459	0,809
SACM1L	0,095	0,563	0,862
ITPR2	0,095	0,613	0,887
RERE	0,094	0,617	0,887
ARHGEF6	0,094	0,485	0,822

AP1M1	0,094	0,566	0,863
ACTN4	0,094	0,636	0,893
PLA2G12A	0,094	0,484	0,822
FIP1L1	0,094	0,584	0,872
ST3GAL1	0,094	0,597	0,881
IRGQ	0,094	0,643	0,897
ATXN7L3B	0,094	0,392	0,769
HLTF	0,094	0,663	0,904
SREK1IP1	0,094	0,596	0,880
SDF4	0,093	0,551	0,856
INPP4B	0,093	0,773	0,944
ACAD10	0,093	0,629	0,891
NCBP2	0,093	0,362	0,751
ZNF430	0,093	0,605	0,884
DPF2	0,093	0,653	0,899
ZNF154	0,093	0,615	0,887
VMA21	0,093	0,578	0,870
IRAK1BP1	0,093	0,635	0,893
FOXJ3	0,093	0,410	0,782
ZNF551	0,093	0,644	0,897
OAF	0,093	0,717	0,925
KMT5B	0,093	0,280	0,692
YIPF3	0,093	0,491	0,824
HECTD4	0,093	0,672	0,908
TGOLN2	0,093	0,541	0,852
NAIP	0,092	0,644	0,897
RIPK1	0,092	0,635	0,893
KPNB1	0,092	0,424	0,789
ZNF260	0,092	0,476	0,819
ARHGEF37	0,092	0,584	0,872
PIP4K2C	0,092	0,654	0,900
CSGALNACT2	0,092	0,490	0,824
TMEM170B	0,092	0,665	0,905
OXSR1	0,092	0,384	0,764
IKZF5	0,092	0,502	0,830
ANTXR2	0,091	0,533	0,848
AFTPH	0,091	0,513	0,837
GGCX	0,091	0,617	0,887
CDKN2AIP	0,091	0,514	0,837
POLR2D	0,091	0,557	0,858
TXNRD3	0,091	0,626	0,889
SERAC1	0,091	0,615	0,887
NPM1	0,091	0,717	0,925
ACVR2A	0,090	0,512	0,837
RMDN3	0,090	0,628	0,891
RSF1	0,090	0,429	0,792
PAPPA	0,090	0,782	0,946
PAMR1	0,090	0,715	0,925
UBA2	0,090	0,576	0,869
OTUD4	0,090	0,637	0,894
PRRC2C	0,090	0,564	0,862
DYNLL1	0,090	0,689	0,914
THRAP3	0,090	0,428	0,792

SHC1	0,090	0,656	0,900
PTPN18	0,090	0,636	0,893
FZD6	0,090	0,544	0,855
NIPAL3	0,090	0,563	0,862
DR1	0,090	0,441	0,797
GNB4	0,089	0,665	0,905
ORC5	0,089	0,588	0,875
PEX26	0,089	0,607	0,885
PELI1	0,089	0,624	0,889
VIPAS39	0,089	0,551	0,856
TMEM123	0,089	0,625	0,889
GPATCH8	0,089	0,633	0,892
CCZ1B	0,089	0,593	0,879
BAP1	0,089	0,575	0,868
MS4A7	0,089	0,796	0,948
ITSN1	0,089	0,661	0,903
CD274	0,089	0,621	0,888
PITRM1	0,089	0,497	0,827
AATF	0,089	0,538	0,850
ARHGEF11	0,089	0,695	0,917
DERL1	0,089	0,478	0,819
HSPBAP1	0,089	0,668	0,907
PPIL2	0,089	0,617	0,887
ACBD5	0,088	0,581	0,871
GPR135	0,088	0,695	0,917
ABITRAM	0,088	0,614	0,887
ZNF507	0,088	0,353	0,744
RNMT	0,088	0,524	0,842
CDV3	0,088	0,526	0,844
CNOT1	0,088	0,366	0,755
ERV3-1	0,088	0,757	0,938
AEBP2	0,088	0,568	0,864
RO60	0,088	0,650	0,899
RSAD2	0,088	0,782	0,946
PRPF3	0,087	0,443	0,798
SMARCD1	0,087	0,656	0,900
KDM4C	0,087	0,466	0,812
LRP4	0,087	0,794	0,948
RPL11	0,087	0,716	0,925
CCAR2	0,087	0,636	0,893
ZNF587	0,087	0,674	0,908
EXOSC6	0,087	0,630	0,892
DAZAP2	0,087	0,388	0,767
ZNF549	0,087	0,676	0,909
NEU1	0,087	0,612	0,886
PGM3	0,087	0,696	0,917
GMPR2	0,087	0,436	0,796
SLC33A1	0,087	0,611	0,886
CLK3	0,087	0,617	0,887
IRAK3	0,087	0,796	0,948
RC3H1	0,087	0,565	0,862
ARHGDIB	0,086	0,612	0,887
TPT1	0,086	0,744	0,934

SH3BGRL	0,086	0,736	0,932
CCDC14	0,086	0,578	0,870
DOP1B	0,086	0,695	0,917
TOM1	0,086	0,632	0,892
FAM168B	0,086	0,491	0,824
ZBTB20	0,086	0,542	0,853
SCYL1	0,086	0,572	0,867
ACTA1	0,086	0,850	0,968
HCFC1	0,086	0,756	0,937
ERGIC3	0,086	0,619	0,888
CETN2	0,086	0,702	0,919
UBL3	0,086	0,550	0,856
NRBP1	0,085	0,454	0,806
EDEM3	0,085	0,584	0,872
HBP1	0,085	0,616	0,887
PXMP4	0,085	0,693	0,917
RAP1GDS1	0,085	0,555	0,858
RNF170	0,085	0,460	0,810
ZNF641	0,085	0,443	0,798
AMY2B	0,085	0,634	0,893
CAPN1	0,085	0,651	0,899
C16orf72	0,085	0,524	0,842
PI16	0,085	0,824	0,958
L3HYPDH	0,084	0,639	0,894
USP3	0,084	0,499	0,829
KLHL5	0,084	0,563	0,861
TIPARP	0,084	0,735	0,932
RGPD4	0,084	0,652	0,899
NCSTN	0,084	0,565	0,862
STAG1	0,084	0,528	0,845
ENC1	0,084	0,793	0,948
LGALS3	0,084	0,657	0,901
WIPF1	0,084	0,595	0,880
RPS2	0,084	0,631	0,892
ANKRD36B	0,084	0,588	0,875
UBAP2L	0,084	0,633	0,892
KLF13	0,083	0,718	0,925
CLCN5	0,083	0,670	0,907
NAT10	0,083	0,667	0,906
TM9SF4	0,083	0,584	0,872
ARF1	0,083	0,538	0,850
ATG2B	0,083	0,485	0,822
ZFYVE9	0,083	0,436	0,796
INPP4A	0,083	0,592	0,879
CLEC2D	0,083	0,648	0,899
AFDN	0,083	0,597	0,881
SEC14L1	0,083	0,644	0,897
GLYR1	0,083	0,631	0,892
FBXO8	0,083	0,623	0,889
CEP170	0,083	0,448	0,802
TNPO3	0,082	0,382	0,763
MTPN	0,082	0,560	0,860
CFAP97	0,082	0,668	0,907

ATF7	0,082	0,651	0,899
MYADM	0,082	0,740	0,933
ARHGEF12	0,082	0,528	0,845
SERINC3	0,082	0,428	0,792
BTBD7	0,082	0,505	0,832
MCRS1	0,082	0,602	0,883
CSNK1E	0,082	0,622	0,888
YWHAH	0,082	0,598	0,881
CITED2	0,082	0,538	0,850
DMTF1	0,082	0,527	0,844
CPQ	0,082	0,529	0,846
ZBTB21	0,082	0,726	0,929
SEC23B	0,082	0,682	0,912
PABPC4	0,082	0,623	0,889
LMAN2L	0,082	0,652	0,899
CLPTM1L	0,082	0,624	0,889
ZNF75D	0,081	0,619	0,888
BRAP	0,081	0,521	0,842
SLU7	0,081	0,535	0,850
XPO5	0,081	0,610	0,885
INSR	0,081	0,705	0,921
TDRD7	0,081	0,541	0,853
ELP1	0,081	0,471	0,816
TOM1L1	0,081	0,653	0,899
NTN4	0,081	0,631	0,892
ACO2	0,081	0,741	0,933
EIF2AK1	0,081	0,521	0,842
SEMA6C	0,081	0,749	0,936
FNIP1	0,081	0,491	0,825
RFX5	0,081	0,644	0,898
SLC25A39	0,081	0,663	0,904
SLC38A9	0,081	0,583	0,872
CHMP1A	0,081	0,654	0,900
AP2A1	0,081	0,665	0,905
KREMEN1	0,080	0,764	0,941
POLR3D	0,080	0,661	0,903
CENPB	0,080	0,673	0,908
DUSP26	0,080	0,726	0,930
MX2	0,080	0,705	0,921
RHOU	0,079	0,827	0,960
SYS1	0,079	0,636	0,893
N4BP1	0,079	0,575	0,868
MIA3	0,079	0,442	0,798
CHURC1	0,079	0,712	0,923
DCLK2	0,079	0,715	0,925
KIF20B	0,079	0,703	0,921
GHR	0,079	0,790	0,948
TBL1XR1	0,079	0,692	0,915
ZNF845	0,079	0,638	0,894
ZCCHC9	0,079	0,623	0,889
FTH1	0,079	0,625	0,889
GTF3C2	0,079	0,700	0,919
DENND5A	0,079	0,603	0,883

CNP	0,079	0,680	0,911
ACOX1	0,079	0,451	0,804
SIK3	0,079	0,695	0,917
PPM1D	0,079	0,663	0,904
EPHA7	0,079	0,764	0,941
RASA2	0,078	0,651	0,899
ATXN2	0,078	0,648	0,899
SLC25A5	0,078	0,492	0,826
EP400	0,078	0,718	0,925
MPEG1	0,078	0,751	0,936
B2M	0,078	0,749	0,936
ZNF274	0,078	0,619	0,888
DHX8	0,078	0,524	0,843
DDX17	0,078	0,670	0,907
ITGA5	0,078	0,721	0,927
DNPEP	0,078	0,606	0,885
DGKE	0,078	0,615	0,887
FAM168A	0,078	0,651	0,899
MDM2	0,078	0,688	0,914
SGCD	0,078	0,758	0,938
PPFIBP2	0,078	0,626	0,889
ALG2	0,078	0,574	0,868
NUP188	0,078	0,671	0,908
TMCC1	0,078	0,508	0,833
ENTPD5	0,077	0,671	0,908
GORASP2	0,077	0,553	0,857
KIFAP3	0,077	0,683	0,913
PDK2	0,077	0,711	0,923
SPTLC1	0,077	0,578	0,870
WASHC5	0,077	0,419	0,785
HELB	0,077	0,729	0,930
OGFOD1	0,077	0,534	0,850
ASH2L	0,077	0,514	0,837
DST	0,077	0,729	0,930
HMG20A	0,077	0,583	0,872
MICU3	0,077	0,720	0,926
CTNNB1	0,077	0,461	0,811
RBM27	0,077	0,541	0,852
EIF3D	0,077	0,611	0,886
ZNF451	0,077	0,654	0,899
PHF10	0,077	0,582	0,872
GLT8D1	0,077	0,449	0,802
MAEA	0,076	0,652	0,899
ZC3H7A	0,076	0,513	0,837
POC1B	0,076	0,579	0,870
USP1	0,076	0,641	0,896
TCAF1	0,076	0,661	0,903
EXTL3	0,076	0,711	0,923
SSPN	0,076	0,728	0,930
WTAP	0,076	0,695	0,917
RSL1D1	0,076	0,478	0,819
TPD52	0,076	0,647	0,899
ATL2	0,076	0,550	0,856

TADA3	0,076	0,691	0,915
ENOX2	0,076	0,565	0,862
GIMAP8	0,076	0,722	0,928
NUP160	0,076	0,524	0,842
SCAP	0,075	0,714	0,924
EFCAB7	0,075	0,731	0,931
RELL1	0,075	0,748	0,936
FBXW5	0,075	0,688	0,914
HMBOX1	0,075	0,566	0,863
ALS2CL	0,075	0,777	0,945
BUD31	0,075	0,686	0,914
SEC11A	0,075	0,678	0,911
NUP153	0,075	0,546	0,855
RNF138	0,075	0,736	0,932
CAND2	0,075	0,796	0,948
GLE1	0,075	0,591	0,878
TSC1	0,075	0,669	0,907
BRWD3	0,075	0,604	0,883
SCAMP2	0,075	0,650	0,899
ATF7IP2	0,074	0,676	0,909
CEP68	0,074	0,582	0,872
ADD1	0,074	0,684	0,913
RRAGC	0,074	0,609	0,885
SDC2	0,074	0,675	0,908
TAOK3	0,074	0,457	0,808
TIMP4	0,074	0,889	0,978
MBOAT2	0,074	0,766	0,941
NAXD	0,074	0,707	0,923
IMMP2L	0,074	0,694	0,917
INPP5K	0,074	0,637	0,893
BCL10	0,074	0,647	0,899
PRDM4	0,074	0,622	0,888
CCDC174	0,073	0,665	0,905
SH2B3	0,073	0,689	0,914
NBEAL1	0,073	0,649	0,899
S100A6	0,073	0,782	0,946
ELOVL1	0,073	0,651	0,899
MAP3K11	0,073	0,765	0,941
KLHL22	0,073	0,757	0,938
CDKN1A	0,073	0,813	0,954
PDCL	0,073	0,613	0,887
PHF3	0,073	0,650	0,899
ZNF398	0,073	0,676	0,909
CUL4A	0,073	0,620	0,888
MSMO1	0,073	0,752	0,936
HNRNPH1	0,073	0,684	0,913
RASSF8	0,073	0,733	0,931
CDC14B	0,073	0,549	0,856
SLC35B4	0,073	0,652	0,899
EXT2	0,072	0,514	0,837
NECAP2	0,072	0,717	0,925
ZNF718	0,072	0,754	0,937
MIPEP	0,072	0,722	0,928

ZNF8	0,072	0,756	0,938
PCGF5	0,072	0,693	0,916
AP2B1	0,071	0,541	0,853
MBOAT1	0,071	0,728	0,930
MAX	0,071	0,586	0,873
CUTA	0,071	0,663	0,904
KDELR2	0,071	0,613	0,887
LAS1L	0,071	0,679	0,911
JDP2	0,071	0,712	0,923
CCNJ	0,071	0,715	0,925
ELMO1	0,071	0,692	0,916
PDXDC1	0,071	0,559	0,859
CSRNP2	0,071	0,667	0,906
RICTOR	0,071	0,783	0,946
AP4B1	0,070	0,723	0,928
LDB2	0,070	0,623	0,889
BEX4	0,070	0,659	0,902
WASL	0,070	0,557	0,858
SAP130	0,070	0,740	0,933
HAUS3	0,070	0,660	0,903
BRD9	0,070	0,647	0,899
UBQLN1	0,070	0,533	0,849
UNC13B	0,070	0,566	0,863
PCMTD2	0,070	0,609	0,885
CNN3	0,070	0,739	0,933
PITPNB	0,070	0,627	0,890
DSG2	0,070	0,609	0,885
ABI2	0,070	0,641	0,896
ALG10B	0,070	0,663	0,904
CCDC93	0,070	0,662	0,904
TMEM30A	0,070	0,672	0,908
PIGM	0,070	0,734	0,932
PHACTR4	0,069	0,654	0,900
DPYSL2	0,069	0,710	0,923
GNAS	0,069	0,698	0,918
DDX59	0,069	0,598	0,881
TAOK1	0,069	0,551	0,856
SPEG	0,069	0,736	0,932
ACTN1	0,069	0,765	0,941
MGA	0,069	0,514	0,837
SPCS3	0,069	0,724	0,928
PRKAB1	0,069	0,740	0,933
ZNF527	0,069	0,701	0,919
XPO6	0,069	0,710	0,923
TMEM128	0,069	0,704	0,921
FAM120B	0,069	0,530	0,846
VPS13B	0,069	0,553	0,857
GOLGB1	0,069	0,588	0,875
MAP3K1	0,069	0,719	0,925
CPEB4	0,069	0,777	0,945
GSTP1	0,068	0,751	0,936
DNAJA1	0,068	0,741	0,933
ZNF780A	0,068	0,642	0,897

TRUB1	0,068	0,807	0,953
TMEM181	0,068	0,592	0,879
KLHL9	0,068	0,640	0,895
INTS6	0,068	0,739	0,933
BLOC1S2	0,067	0,750	0,936
DUSP11	0,067	0,670	0,907
WDR82	0,067	0,586	0,874
TCP11L1	0,067	0,748	0,936
MSL1	0,067	0,673	0,908
LEMD3	0,067	0,619	0,888
C12orf29	0,067	0,773	0,944
MTM1	0,067	0,601	0,883
DENND4A	0,067	0,682	0,912
CTC1	0,067	0,771	0,943
ZNF84	0,067	0,611	0,886
LONP1	0,067	0,732	0,931
CCNT1	0,067	0,601	0,883
TMBIM4	0,067	0,687	0,914
TAF1D	0,067	0,745	0,935
PRIMPOL	0,067	0,671	0,908
SNX12	0,067	0,652	0,899
TRIM33	0,067	0,568	0,864
EFR3A	0,066	0,636	0,893
GBA	0,066	0,719	0,925
ITPRID2	0,066	0,620	0,888
EPB41L5	0,066	0,531	0,847
GPALPP1	0,066	0,598	0,881
ALDH2	0,066	0,736	0,932
SURF6	0,066	0,742	0,933
ATM	0,066	0,695	0,917
PSKH1	0,066	0,736	0,932
NUDCD3	0,066	0,674	0,908
TMEM127	0,066	0,708	0,923
UNC50	0,066	0,600	0,882
MCAM	0,066	0,755	0,937
MIGA1	0,066	0,653	0,899
SLC16A1	0,066	0,803	0,951
ZSCAN25	0,066	0,774	0,944
GOLGA8Q	0,065	0,779	0,945
CLCC1	0,065	0,592	0,879
TEX261	0,065	0,648	0,899
RNF41	0,065	0,606	0,885
SEMA3B	0,065	0,778	0,945
TAB3	0,065	0,555	0,858
PTPN1	0,065	0,684	0,913
RNF111	0,065	0,625	0,889
LRP6	0,065	0,531	0,847
SP110	0,065	0,761	0,940
MTDH	0,064	0,598	0,881
ZNF229	0,064	0,742	0,933
RRBP1	0,064	0,797	0,949
SENP2	0,064	0,561	0,860
GNAI2	0,064	0,697	0,918

FLNB	0,064	0,798	0,949
CREBBP	0,064	0,794	0,948
ZHX1	0,064	0,742	0,933
DGLUCY	0,064	0,773	0,944
ZNF268	0,064	0,741	0,933
MOB1A	0,064	0,682	0,912
MN1	0,064	0,809	0,953
ATP2C1	0,064	0,713	0,924
CMTR1	0,064	0,702	0,919
SLC41A3	0,064	0,710	0,923
HEATR6	0,064	0,740	0,933
RNF4	0,063	0,700	0,918
ST3GAL6	0,063	0,791	0,948
SYNJ1	0,063	0,679	0,911
AAAS	0,063	0,729	0,930
MT-ND6	0,063	0,768	0,942
DDI2	0,063	0,729	0,930
EIF5A2	0,063	0,715	0,925
RPL6	0,063	0,796	0,948
MT-ND2	0,063	0,801	0,950
PPP1R12C	0,063	0,794	0,948
KIAA0930	0,063	0,730	0,930
ZBTB33	0,063	0,706	0,922
SAE1	0,063	0,572	0,867
PPP2R5D	0,063	0,765	0,941
KCTD2	0,063	0,740	0,933
ARFIP1	0,063	0,702	0,919
DERL2	0,063	0,643	0,897
HNRNPL	0,063	0,673	0,908
TRPC4AP	0,063	0,615	0,887
H2AFJ	0,063	0,787	0,948
NELFE	0,063	0,685	0,913
INTU	0,063	0,698	0,918
GORASP1	0,062	0,741	0,933
EIF5	0,062	0,633	0,893
USP48	0,062	0,579	0,870
VPS53	0,062	0,793	0,948
RHOBTB3	0,062	0,676	0,909
IFT81	0,062	0,649	0,899
SYNCRIP	0,062	0,639	0,894
EIF2AK4	0,062	0,499	0,828
MAP2K6	0,062	0,810	0,954
ZBED1	0,062	0,749	0,936
SRBD1	0,062	0,710	0,923
KAT2A	0,062	0,759	0,939
ZHX3	0,062	0,791	0,948
AKR1A1	0,061	0,730	0,930
RPS10	0,061	0,785	0,947
MTHFD2L	0,061	0,748	0,936
IFI44	0,061	0,793	0,948
TOR1A	0,061	0,677	0,910
FAF2	0,061	0,598	0,882
AKAP10	0,061	0,578	0,870

XIAP	0,061	0,567	0,864
GPBP1L1	0,061	0,453	0,806
OSTC	0,061	0,815	0,954
PSMD8	0,061	0,760	0,939
ZNF136	0,061	0,696	0,917
HMGXB4	0,061	0,706	0,922
LPGAT1	0,061	0,671	0,908
HTATSF1	0,061	0,631	0,892
SMPD4	0,061	0,735	0,932
GIT1	0,061	0,757	0,938
POGLUT1	0,061	0,733	0,931
ARHGAP17	0,060	0,784	0,946
ZSCAN12	0,060	0,751	0,936
PACRGL	0,060	0,719	0,925
MARCH3	0,060	0,767	0,942
CLIC5	0,060	0,810	0,954
TMEM87A	0,060	0,689	0,914
EXOC2	0,060	0,652	0,899
SNX9	0,060	0,639	0,894
GALNT7	0,060	0,675	0,908
STEAP2	0,060	0,782	0,946
NUPR1	0,060	0,792	0,948
TMEM192	0,060	0,613	0,887
CERS5	0,059	0,713	0,924
UBAP2	0,059	0,664	0,905
CLIP4	0,059	0,701	0,919
SPG11	0,059	0,555	0,858
SP3	0,059	0,734	0,932
NAP1L2	0,059	0,812	0,954
STXBP1	0,059	0,781	0,946
ERC1	0,059	0,709	0,923
BBS2	0,059	0,657	0,901
KCNAB1	0,059	0,712	0,923
PPCS	0,059	0,749	0,936
HPS4	0,059	0,757	0,938
MACO1	0,059	0,627	0,890
SSRP1	0,058	0,707	0,922
PPP6C	0,058	0,622	0,889
SELENOM	0,058	0,756	0,938
YWHAB	0,058	0,584	0,872
ARHGAP12	0,058	0,736	0,932
MAFG	0,058	0,738	0,933
SASH1	0,057	0,799	0,949
DTX3	0,057	0,723	0,928
MGAT4A	0,057	0,717	0,925
ELMO2	0,057	0,712	0,923
RBBP8	0,057	0,803	0,951
TBC1D31	0,057	0,808	0,953
WRN	0,057	0,617	0,887
KMT2C	0,057	0,718	0,925
RRP1B	0,057	0,735	0,932
CARD6	0,057	0,774	0,944
MAP3K13	0,057	0,685	0,913

ZNF594	0,057	0,748	0,936
PHF1	0,057	0,692	0,916
HRNR	0,057	0,891	0,978
PDPK1	0,057	0,753	0,937
RAD23B	0,057	0,625	0,889
WDR45B	0,057	0,705	0,921
SOX4	0,056	0,827	0,960
SSX2IP	0,056	0,814	0,954
SPOP	0,056	0,614	0,887
CLTA	0,056	0,727	0,930
GOLGA8A	0,056	0,796	0,948
AKAP7	0,056	0,728	0,930
ZNF662	0,056	0,774	0,944
CPEB2	0,056	0,749	0,936
RPL23	0,056	0,809	0,953
HSDL2	0,056	0,789	0,948
ZNF211	0,056	0,732	0,931
IL6ST	0,056	0,833	0,963
MYL12A	0,056	0,891	0,978
EXTL2	0,056	0,776	0,945
ITSN2	0,056	0,606	0,885
PIK3C2B	0,056	0,778	0,945
SLC35E1	0,056	0,740	0,933
UNC45B	0,056	0,838	0,964
MYLIP	0,056	0,729	0,930
CNOT6L	0,056	0,699	0,918
KIF1A	0,056	0,852	0,970
SRRT	0,056	0,736	0,932
SENP6	0,056	0,691	0,915
BCAT1	0,056	0,878	0,977
ZSWIM7	0,055	0,758	0,938
INTS14	0,055	0,680	0,911
CD55	0,055	0,874	0,976
PCCB	0,055	0,785	0,947
SMIM7	0,055	0,712	0,923
SH3YL1	0,055	0,698	0,918
E2F3	0,055	0,800	0,950
POU2F1	0,055	0,696	0,917
TARS2	0,055	0,763	0,941
ITPR1	0,055	0,765	0,941
MAP1LC3B	0,055	0,621	0,888
GALNT11	0,054	0,575	0,868
ZNF382	0,054	0,736	0,932
FAM149B1	0,054	0,688	0,914
DAB2IP	0,054	0,801	0,950
IFT22	0,054	0,758	0,938
ARL10	0,054	0,800	0,950
MCM3AP	0,054	0,792	0,948
LRCH1	0,054	0,659	0,902
RPL24	0,054	0,744	0,934
UBE2Q1	0,054	0,705	0,921
POGK	0,054	0,694	0,917
UBQLN4	0,054	0,742	0,933

STK10	0,054	0,797	0,949
CCDC126	0,054	0,848	0,968
SP100	0,053	0,684	0,913
BCAR3	0,053	0,855	0,970
NLRC5	0,053	0,844	0,967
SCAF8	0,053	0,674	0,908
CCSAP	0,053	0,712	0,923
RASSF3	0,053	0,732	0,931
EXOC7	0,053	0,755	0,937
KIAA0355	0,053	0,742	0,933
CSPP1	0,052	0,706	0,922
CPLANE1	0,052	0,737	0,933
SEL1L	0,052	0,665	0,905
MAP2K5	0,052	0,761	0,940
PISD	0,052	0,795	0,948
ZHX2	0,052	0,790	0,948
APOLD1	0,052	0,899	0,980
ZNF277	0,052	0,780	0,946
RANBP9	0,052	0,697	0,918
GIGYF2	0,052	0,568	0,864
TMEM214	0,052	0,756	0,938
NPIPA5	0,052	0,809	0,953
TRAM2	0,052	0,760	0,939
AKAP6	0,052	0,874	0,975
WDR46	0,051	0,815	0,954
ATG16L1	0,051	0,833	0,963
DYRK3	0,051	0,823	0,958
PAN2	0,051	0,801	0,950
SETX	0,051	0,661	0,903
ITM2C	0,051	0,746	0,935
VPS26B	0,051	0,796	0,948
SPTLC3	0,051	0,777	0,945
HNRNPU	0,051	0,717	0,925
SUN1	0,051	0,757	0,938
C2CD3	0,051	0,863	0,973
SHOC1	0,051	0,841	0,965
DGCR6L	0,051	0,817	0,955
EGLN3	0,051	0,877	0,976
SLTM	0,051	0,708	0,923
FAM189A2	0,051	0,846	0,967
ATG4B	0,051	0,821	0,957
FAM111A	0,050	0,796	0,948
HLCS	0,050	0,769	0,942
ZBTB40	0,050	0,798	0,949
AGAP4	0,050	0,797	0,949
SEC23IP	0,050	0,634	0,893
RCC1L	0,050	0,772	0,944
PHKA2	0,050	0,781	0,946
RPS6KB1	0,050	0,656	0,900
RNF123	0,050	0,840	0,965
BAG5	0,050	0,655	0,900
RPLP0	0,050	0,848	0,968
MTF1	0,050	0,793	0,948

GAS6	0,050	0,769	0,942
ANAPC1	0,050	0,599	0,882
MED24	0,050	0,774	0,944
SLC10A7	0,050	0,785	0,947
LARP1B	0,050	0,753	0,937
NCOA2	0,049	0,661	0,903
STXBP5	0,049	0,774	0,944
ZNF132	0,049	0,860	0,971
TYK2	0,049	0,871	0,974
MYO6	0,049	0,714	0,924
BRK1	0,049	0,705	0,921
ZNF611	0,049	0,788	0,948
TCEA1	0,049	0,724	0,928
MYO1E	0,049	0,739	0,933
PCBD2	0,049	0,797	0,949
MBD2	0,049	0,750	0,936
AGPAT5	0,049	0,766	0,941
STX17	0,048	0,621	0,888
CDS2	0,048	0,768	0,942
GLUD1	0,048	0,606	0,885
KLHDC8B	0,048	0,783	0,946
C15orf41	0,048	0,828	0,961
EIF4H	0,048	0,633	0,892
SUSD1	0,048	0,801	0,950
MIER1	0,048	0,760	0,939
SLC29A1	0,048	0,785	0,947
RSBN1L	0,048	0,712	0,923
TMEM164	0,048	0,800	0,950
CDC42SE2	0,048	0,765	0,941
CDC73	0,048	0,682	0,912
RNF114	0,047	0,754	0,937
ADAM17	0,047	0,773	0,944
ALKBH8	0,047	0,783	0,946
IL11RA	0,047	0,770	0,943
NOL4L	0,047	0,856	0,970
RNF20	0,047	0,675	0,908
GLOD4	0,047	0,721	0,927
CLASP1	0,047	0,741	0,933
PEA15	0,047	0,790	0,948
DFFA	0,047	0,783	0,946
SRPRA	0,047	0,725	0,928
ARF6	0,047	0,763	0,941
ANAPC4	0,046	0,699	0,918
GOLGA1	0,046	0,785	0,947
L3MBTL2	0,046	0,858	0,970
QRICH1	0,046	0,726	0,930
POR	0,046	0,836	0,963
INO80	0,046	0,753	0,937
BAZ1A	0,046	0,836	0,963
ZNF736	0,046	0,742	0,933
DOCK7	0,046	0,722	0,927
UIMC1	0,046	0,746	0,935
KMT5A	0,046	0,796	0,948

IPO7	0,046	0,795	0,948
ASAP1	0,046	0,788	0,948
SPIN3	0,046	0,812	0,954
PPT1	0,046	0,754	0,937
NQO1	0,046	0,904	0,981
ATP8A1	0,045	0,733	0,931
PAFAH1B2	0,045	0,669	0,907
TRIP12	0,045	0,638	0,894
THSD4	0,045	0,897	0,979
SUPT20H	0,045	0,688	0,914
SEMA3G	0,045	0,837	0,963
DENND6A	0,045	0,764	0,941
NF1	0,045	0,724	0,928
MOB1B	0,045	0,780	0,946
PNPLA2	0,045	0,863	0,973
GAB1	0,045	0,775	0,944
PHKB	0,045	0,709	0,923
GDAP2	0,045	0,763	0,941
SMAP2	0,045	0,853	0,970
SMG8	0,045	0,801	0,950
SYNE3	0,045	0,905	0,981
SEC13	0,044	0,708	0,923
EHD4	0,044	0,808	0,953
SUGT1	0,044	0,729	0,930
PRPF38A	0,044	0,706	0,922
MED1	0,044	0,747	0,936
CEP112	0,044	0,713	0,924
PCYOX1	0,044	0,729	0,930
TRIM25	0,044	0,812	0,954
ZNF510	0,044	0,735	0,932
TCTN1	0,044	0,724	0,928
FOXO1	0,044	0,843	0,966
MED17	0,044	0,852	0,970
RIC1	0,043	0,784	0,946
GRK2	0,043	0,791	0,948
TBC1D13	0,043	0,891	0,978
ZNF528	0,043	0,778	0,945
FYN	0,043	0,800	0,950
ATXN10	0,043	0,732	0,931
ZNF440	0,043	0,807	0,953
GTF2B	0,043	0,834	0,963
NPIPB12	0,043	0,860	0,971
IFI44L	0,043	0,901	0,980
NUMB	0,043	0,769	0,942
SLC49A4	0,043	0,809	0,953
C1orf216	0,043	0,838	0,964
KLHL42	0,043	0,774	0,944
OLFML2A	0,043	0,864	0,973
SBNO1	0,043	0,699	0,918
SETDB1	0,042	0,800	0,950
SCAF11	0,042	0,709	0,923
ST8SIA4	0,042	0,881	0,977
RAP2A	0,042	0,836	0,963

CALCOCO1	0,042	0,778	0,945
SYNPO	0,042	0,854	0,970
AQP1	0,042	0,921	0,987
GZF1	0,042	0,765	0,941
SMIM15	0,042	0,823	0,958
DEF8	0,042	0,831	0,962
USP9X	0,042	0,736	0,932
SYF2	0,042	0,814	0,954
CEP350	0,042	0,686	0,914
AGO3	0,042	0,814	0,954
H3F3B	0,041	0,807	0,953
DHX9	0,041	0,708	0,923
GRIK2	0,041	0,899	0,980
MMS19	0,041	0,754	0,937
SIKE1	0,041	0,774	0,944
GRK5	0,041	0,853	0,970
BRD7	0,041	0,617	0,887
TOPORS	0,041	0,812	0,954
C1RL	0,041	0,891	0,978
DESI2	0,041	0,795	0,948
CRCP	0,041	0,879	0,977
ANKRD36C	0,041	0,794	0,948
ZNF12	0,041	0,723	0,928
KDM7A	0,041	0,801	0,950
HPS3	0,041	0,758	0,938
ALPK1	0,041	0,836	0,963
ELK3	0,041	0,771	0,943
ABCF3	0,041	0,781	0,946
RAB11FIP2	0,041	0,777	0,945
RPL14	0,040	0,835	0,963
CAMK2D	0,040	0,846	0,967
C2orf68	0,040	0,821	0,957
CLCN6	0,040	0,877	0,976
NFX1	0,040	0,710	0,923
CIB1	0,040	0,810	0,954
ADIPOR1	0,040	0,781	0,946
RBFOX2	0,040	0,714	0,924
SNRNP40	0,040	0,825	0,959
PGRMC2	0,040	0,790	0,948
STON1	0,040	0,906	0,981
ZNF134	0,040	0,788	0,948
ZNF383	0,040	0,782	0,946
KIF5B	0,040	0,823	0,958
PIGT	0,040	0,792	0,948
SLC14A1	0,040	0,895	0,979
EHD2	0,040	0,886	0,978
HEMK1	0,040	0,882	0,977
CREB3	0,039	0,818	0,955
API5	0,039	0,743	0,934
WDFY3	0,039	0,779	0,945
PTK2	0,039	0,690	0,915
PLVAP	0,039	0,896	0,979
XPNPEP3	0,039	0,813	0,954

DNAH11	0,039	0,870	0,974
RNF145	0,039	0,814	0,954
E2F6	0,039	0,793	0,948
DUSP14	0,039	0,869	0,974
VPS54	0,039	0,788	0,948
SAR1A	0,039	0,799	0,949
HSD17B11	0,038	0,879	0,977
KRIT1	0,038	0,833	0,962
ANKRD12	0,038	0,815	0,954
PLEKHM1	0,038	0,861	0,971
BTF3	0,038	0,764	0,941
ZEB2	0,038	0,750	0,936
PNPO	0,038	0,869	0,974
CLK2	0,038	0,861	0,971
YIPF4	0,038	0,732	0,931
GABPA	0,038	0,847	0,967
KLHL12	0,038	0,730	0,930
ZNF468	0,038	0,829	0,961
POM121	0,038	0,881	0,977
UTRN	0,038	0,781	0,946
NPHP1	0,038	0,821	0,957
VRK3	0,037	0,905	0,981
SIDT2	0,037	0,888	0,978
YTHDF1	0,037	0,857	0,970
SORT1	0,037	0,824	0,958
AC138969.1	0,037	0,856	0,970
GPATCH11	0,037	0,832	0,962
MCM3	0,037	0,981	0,998
OTUD3	0,037	0,846	0,967
TAF1B	0,037	0,836	0,963
NOP14	0,037	0,832	0,962
NMRK2	0,037	0,904	0,981
GNPDA1	0,037	0,795	0,948
PLEKHM2	0,037	0,827	0,960
BFAR	0,037	0,745	0,934
ZNF419	0,037	0,878	0,977
RBM12	0,037	0,757	0,938
MPV17	0,036	0,792	0,948
LARP4B	0,036	0,833	0,962
ZMYM5	0,036	0,798	0,949
GSAP	0,036	0,783	0,946
DENND2C	0,036	0,835	0,963
PHLPP1	0,036	0,888	0,978
CLUH	0,036	0,881	0,977
HNRNPUL2	0,036	0,816	0,954
HNRNPR	0,036	0,772	0,944
GORAB	0,036	0,825	0,959
MEA1	0,036	0,836	0,963
TP53BP2	0,036	0,792	0,948
ICE1	0,035	0,711	0,923
ZNF519	0,035	0,870	0,974
TOMM7	0,035	0,852	0,970
ICE2	0,035	0,749	0,936

PFN2	0,035	0,811	0,954
USP33	0,035	0,830	0,962
MT-ND4	0,035	0,895	0,979
ZNF281	0,035	0,817	0,955
NFIB	0,035	0,764	0,941
NCOR1	0,034	0,791	0,948
USP37	0,034	0,808	0,953
NPLOC4	0,034	0,834	0,963
PARD3	0,034	0,710	0,923
XPO4	0,034	0,807	0,953
TSN	0,034	0,767	0,941
EEF1AKMT2	0,034	0,839	0,964
CLEC2B	0,034	0,900	0,980
ERO1A	0,034	0,794	0,948
COMT	0,034	0,880	0,977
ABL2	0,034	0,894	0,979
ITPRIP	0,034	0,899	0,980
SMC5	0,034	0,841	0,965
GAPVD1	0,034	0,789	0,948
FIGN	0,034	0,781	0,946
RNF6	0,034	0,823	0,958
ATG4C	0,034	0,853	0,970
TBC1D4	0,034	0,871	0,974
NUDCD2	0,034	0,821	0,957
FAM241A	0,034	0,857	0,970
SEC61B	0,034	0,882	0,977
SERBP1	0,034	0,831	0,962
STK24	0,033	0,856	0,970
PSMD5	0,033	0,749	0,936
MED15	0,033	0,860	0,971
RGL1	0,033	0,842	0,965
ZNF394	0,033	0,889	0,978
NUS1	0,033	0,828	0,960
SETD4	0,033	0,884	0,977
ERCC3	0,033	0,939	0,992
SMC3	0,033	0,783	0,946
SUPT7L	0,033	0,855	0,970
ZNF235	0,033	0,887	0,978
EXOG	0,033	0,896	0,979
PRKAA2	0,033	0,939	0,992
SET	0,033	0,807	0,953
TRIP11	0,033	0,789	0,948
RBM23	0,033	0,794	0,948
ZNF506	0,033	0,830	0,962
VCL	0,032	0,859	0,971
AHCY	0,032	0,906	0,982
BCAT2	0,032	0,874	0,975
CSTF3	0,032	0,789	0,948
MTX3	0,032	0,803	0,951
CLN8	0,032	0,859	0,971
EXOSC7	0,032	0,840	0,964
METTL4	0,032	0,828	0,961
MYCBP2	0,032	0,835	0,963

PKIA	0,032	0,920	0,986
DENND1B	0,032	0,890	0,978
ANO2	0,032	0,851	0,969
CRYBG3	0,032	0,825	0,959
CNOT6	0,032	0,792	0,948
ADAT1	0,032	0,886	0,978
FNDC3B	0,032	0,795	0,948
KLHL8	0,032	0,836	0,963
SBDS	0,032	0,829	0,961
TERF1	0,032	0,785	0,947
NAGK	0,032	0,896	0,979
TAGLN2	0,031	0,896	0,979
ZNF226	0,031	0,807	0,953
YWHAZ	0,031	0,832	0,962
HNRNPM	0,031	0,793	0,948
DCAF7	0,031	0,860	0,971
NPIPB3	0,031	0,905	0,981
GTF2H1	0,031	0,859	0,971
SART1	0,031	0,877	0,976
PPARA	0,031	0,890	0,978
TP53BP1	0,031	0,807	0,953
SRSF5	0,031	0,810	0,954
ZNF529	0,031	0,838	0,964
RLF	0,031	0,864	0,973
TUT4	0,030	0,809	0,953
RAB35	0,030	0,936	0,991
ZXDC	0,030	0,872	0,975
TUBGCP2	0,030	0,842	0,965
CDK12	0,030	0,852	0,969
RNF185	0,030	0,872	0,975
TMUB2	0,030	0,907	0,982
MON1B	0,030	0,933	0,991
CLK1	0,030	0,895	0,979
SLC26A2	0,030	0,839	0,964
BAG6	0,030	0,881	0,977
HNRNPA3	0,030	0,801	0,950
HNRNPH3	0,030	0,827	0,960
RASA1	0,030	0,818	0,955
VAMP7	0,030	0,870	0,974
NPIPA3	0,030	0,931	0,990
ZNF512	0,030	0,814	0,954
SH3GLB1	0,030	0,841	0,965
LAMTOR4	0,030	0,880	0,977
ZNF354C	0,030	0,849	0,968
NEDD4L	0,030	0,890	0,978
NHLRC3	0,030	0,861	0,971
SGCA	0,030	0,889	0,978
CHST11	0,029	0,834	0,963
EIF4G2	0,029	0,834	0,963
TRAF6	0,029	0,825	0,959
CWC22	0,029	0,858	0,971
ANKRD28	0,029	0,852	0,970
ERGIC1	0,029	0,832	0,962

NFYB	0,029	0,867	0,974
SAV1	0,029	0,809	0,953
PLEKHO1	0,029	0,880	0,977
YY1AP1	0,029	0,815	0,954
SORBS1	0,029	0,908	0,983
ZNF267	0,029	0,891	0,978
INO80D	0,029	0,772	0,944
DYM	0,029	0,753	0,937
ARF5	0,029	0,884	0,977
MSTO1	0,029	0,931	0,990
RPAP2	0,029	0,774	0,944
SRSF2	0,029	0,854	0,970
ZNF441	0,028	0,909	0,983
ZNF322	0,028	0,868	0,974
PRICKLE2	0,028	0,892	0,979
MEDAG	0,028	0,955	0,994
NSD1	0,028	0,793	0,948
USP40	0,028	0,799	0,950
RLIM	0,028	0,815	0,954
POLR1D	0,028	0,824	0,958
SGPL1	0,028	0,894	0,979
AIDA	0,028	0,849	0,968
RPL31	0,028	0,921	0,987
RFC1	0,028	0,840	0,964
CELF2	0,028	0,832	0,962
DRG2	0,028	0,941	0,992
TXNRD1	0,027	0,896	0,979
LRIG2	0,027	0,849	0,968
ATP6V1A	0,027	0,862	0,972
GPR180	0,027	0,881	0,977
CASP2	0,027	0,941	0,992
PTPN4	0,027	0,875	0,976
ILF3	0,027	0,870	0,974
ACIN1	0,027	0,872	0,975
PPP2CB	0,027	0,785	0,947
VCAN	0,027	0,931	0,990
NSUN6	0,027	0,845	0,967
PPP4R3A	0,026	0,824	0,958
LRRC8B	0,026	0,889	0,978
PANK3	0,026	0,879	0,977
NDST1	0,026	0,924	0,988
LRRC59	0,026	0,876	0,976
LANCL2	0,026	0,912	0,984
ZNF546	0,026	0,848	0,968
ZNF10	0,026	0,870	0,974
RAPGEF2	0,026	0,822	0,958
TRAPPC10	0,025	0,857	0,970
KAT7	0,025	0,866	0,973
VARS	0,025	0,909	0,983
AGPAT2	0,025	0,923	0,987
FCF1	0,025	0,822	0,958
MPHOSPH10	0,025	0,806	0,953
ZFP30	0,025	0,876	0,976

ZNF814	0,025	0,901	0,980
CDK13	0,025	0,847	0,968
FGFR1	0,025	0,905	0,981
UBP1	0,025	0,817	0,955
ETV1	0,025	0,930	0,990
VCP	0,025	0,858	0,971
CREB3L2	0,025	0,898	0,980
DPY19L4	0,025	0,918	0,985
DNAJB6	0,025	0,865	0,973
SPOPL	0,025	0,897	0,979
NHLRC2	0,025	0,901	0,980
SCARB2	0,024	0,829	0,962
COA5	0,024	0,867	0,973
LCMT1	0,024	0,947	0,993
ASPH	0,024	0,909	0,983
ELP3	0,024	0,801	0,950
TUT7	0,024	0,851	0,969
SMIM3	0,024	0,932	0,990
FAM210B	0,024	0,829	0,962
HAUS6	0,024	0,851	0,969
SLC2A13	0,024	0,903	0,981
INSIG2	0,024	0,880	0,977
NKAP	0,024	0,874	0,975
SGMS1	0,024	0,863	0,973
ZNF45	0,024	0,891	0,978
ATP6V0E2	0,024	0,920	0,986
WARS2	0,024	0,848	0,968
EIF4G3	0,024	0,841	0,965
ZBTB38	0,024	0,814	0,954
PSMG2	0,024	0,866	0,973
CTBS	0,024	0,895	0,979
SPATA5	0,023	0,893	0,979
CDYL	0,023	0,896	0,979
NCBP1	0,023	0,824	0,958
HMGCR	0,023	0,881	0,977
RREB1	0,023	0,898	0,980
RAB21	0,023	0,878	0,977
VWA8	0,023	0,897	0,979
MADD	0,023	0,919	0,986
USP25	0,023	0,875	0,976
GIPC1	0,023	0,901	0,980
FIBP	0,023	0,942	0,992
IQSEC1	0,022	0,922	0,987
ZNF540	0,022	0,888	0,978
FRY	0,022	0,895	0,979
SERF2	0,022	0,918	0,985
TENT2	0,022	0,873	0,975
ZNF559	0,022	0,887	0,978
AKIRIN2	0,022	0,902	0,980
ZNF131	0,022	0,846	0,967
GSPT2	0,022	0,918	0,985
UTP18	0,022	0,890	0,978
CTNND2	0,022	0,949	0,993

RBM12B	0,022	0,878	0,977
KIAA2013	0,021	0,975	0,998
TRIOBP	0,021	0,898	0,980
FBXW2	0,021	0,861	0,971
ZNF180	0,021	0,903	0,980
TUBGCP3	0,021	0,965	0,996
RIN2	0,021	0,855	0,970
NAPB	0,021	0,929	0,990
SLC35A5	0,021	0,904	0,981
UPP1	0,021	0,920	0,986
GOLPH3	0,021	0,866	0,973
ERP29	0,021	0,878	0,977
ACTR2	0,021	0,869	0,974
INIP	0,021	0,887	0,978
USP4	0,021	0,864	0,973
SLC35A3	0,020	0,886	0,978
GLB1	0,020	0,934	0,991
ZNF776	0,020	0,883	0,977
ABCC4	0,020	0,927	0,989
ZNF766	0,020	0,877	0,976
LLPH	0,020	0,892	0,979
C22orf39	0,020	0,901	0,980
DDOST	0,020	0,887	0,978
ANO10	0,020	0,902	0,980
CORO6	0,020	0,906	0,982
ABHD12	0,020	0,904	0,981
ZNF92	0,020	0,909	0,983
MT-ND4L	0,020	0,962	0,995
TTC7B	0,020	0,913	0,984
FBXL4	0,020	0,886	0,978
SUZ12	0,020	0,881	0,977
UBE2G1	0,020	0,868	0,974
PARP14	0,020	0,928	0,989
ARL15	0,020	0,912	0,984
CD2AP	0,020	0,916	0,984
MRPL10	0,019	0,956	0,994
MFAP1	0,019	0,893	0,979
SRSF6	0,019	0,896	0,979
PM20D2	0,019	0,911	0,983
RYK	0,019	0,863	0,972
TEP1	0,019	0,928	0,989
FBXO40	0,019	0,954	0,994
PITPNA	0,019	0,900	0,980
AHCTF1	0,019	0,872	0,975
NUDT5	0,019	0,896	0,979
SENP5	0,019	0,861	0,972
DCAF1	0,019	0,987	1,000
C21orf91	0,019	0,938	0,992
UHRF2	0,019	0,872	0,975
LAMA5	0,019	0,925	0,988
FKTN	0,019	0,883	0,977
ENO1	0,018	0,937	0,992
PDZD2	0,018	0,933	0,991

LONP2	0,018	0,868	0,974
ACAD9	0,018	0,910	0,983
RANBP3	0,018	0,905	0,981
CAVIN2	0,018	0,928	0,989
HUS1	0,018	0,987	1,000
EEF1D	0,018	0,926	0,989
ARMC2	0,018	0,940	0,992
ZNF91	0,018	0,933	0,991
TTLL5	0,018	0,963	0,995
HERC1	0,018	0,906	0,982
POLA1	0,018	0,844	0,967
SEMA5A	0,018	0,900	0,980
FSD1L	0,017	0,943	0,993
AGPS	0,017	0,929	0,990
DHX33	0,017	0,947	0,993
ZNF544	0,017	0,910	0,983
ACOX2	0,017	0,975	0,998
MAP3K7	0,017	0,901	0,980
STK11	0,017	0,914	0,984
TMEM185A	0,017	0,920	0,986
ERBIN	0,017	0,917	0,985
UBR5	0,017	0,863	0,973
PDK4	0,017	0,977	0,998
MXI1	0,017	0,896	0,979
SLC4A1AP	0,017	0,879	0,977
MRE11	0,017	0,868	0,974
GPS1	0,017	0,924	0,988
GCLC	0,017	0,879	0,977
ARID4A	0,017	0,897	0,979
VAMP3	0,017	0,903	0,981
CSTF2T	0,017	0,898	0,980
PTGES3	0,017	0,910	0,983
REV1	0,017	0,892	0,979
MAP9	0,017	0,938	0,992
NIFK	0,016	0,938	0,992
PSEN1	0,016	0,883	0,977
BICD2	0,016	0,945	0,993
EPS15	0,016	0,881	0,977
EHMT1	0,016	0,929	0,990
ERI1	0,016	0,930	0,990
CIR1	0,016	0,913	0,984
GATC	0,016	0,936	0,991
RAB28	0,016	0,914	0,984
NMT1	0,016	0,910	0,983
SLC39A7	0,016	0,895	0,979
ALDH7A1	0,016	0,902	0,980
NRBF2	0,016	0,921	0,987
XPOT	0,016	0,924	0,988
TRAF3IP2	0,015	0,936	0,991
ASXL1	0,015	0,936	0,991
UTP20	0,015	0,876	0,976
SDAD1	0,015	0,894	0,979
CD164	0,015	0,945	0,993

PDE12	0,015	0,915	0,984
TBPL1	0,015	0,933	0,991
RBM48	0,015	0,939	0,992
USP36	0,015	0,944	0,993
DGCR8	0,015	0,999	1,000
CRNKL1	0,015	0,936	0,991
STIM1	0,015	0,945	0,993
FKBP7	0,015	0,936	0,991
TM9SF3	0,015	0,924	0,988
ARIH1	0,015	0,900	0,980
PIK3R4	0,015	0,875	0,976
SPIDR	0,015	0,929	0,990
ZNF678	0,015	0,937	0,992
ZNF333	0,014	0,991	1,000
EIF4A3	0,014	0,989	1,000
ELAVL1	0,014	0,940	0,992
AMFR	0,014	0,918	0,985
NDEL1	0,014	0,938	0,992
ECHDC3	0,014	0,962	0,995
IK	0,014	0,921	0,987
BAG4	0,014	0,935	0,991
SOD2	0,014	0,949	0,993
ZNF521	0,014	1,000	1,000
IGF2BP2	0,014	0,981	0,998
NVL	0,014	0,927	0,989
ACTR3	0,013	0,945	0,993
ZNF234	0,013	0,935	0,991
ENPP2	0,013	0,965	0,996
GTPBP2	0,013	0,936	0,991
CEP85	0,013	0,963	0,995
VPS16	0,013	0,997	1,000
QDPR	0,013	0,940	0,992
SUN2	0,013	0,958	0,995
KLF8	0,013	0,951	0,993
TMEM14C	0,013	0,934	0,991
ATP6V1H	0,013	0,950	0,993
GLRX2	0,013	0,933	0,991
MOSMO	0,013	0,916	0,985
MYL3	0,012	0,981	0,998
APLP2	0,012	0,930	0,990
GSK3B	0,012	0,914	0,984
TADA2A	0,012	0,945	0,993
HSPA4	0,012	0,943	0,993
AGO2	0,012	0,956	0,994
TMX1	0,012	0,954	0,994
PALM2-AKAP2	0,012	0,940	0,992
GTDC1	0,012	0,915	0,984
LNPEP	0,012	0,934	0,991
RBM33	0,012	0,928	0,989
HIF1A	0,011	0,949	0,993
PTPRM	0,011	0,950	0,993
FAM92A	0,011	0,945	0,993
CDKL5	0,011	0,943	0,993

SOCS4	0,011	0,955	0,994
CTBP1	0,011	0,939	0,992
RBMXL1	0,011	0,915	0,984
TAB2	0,011	0,920	0,986
AFG3L2	0,011	0,945	0,993
BMPR1A	0,011	0,932	0,990
SMG1	0,011	0,946	0,993
PAF1	0,011	0,958	0,995
HIST2H2BE	0,011	0,954	0,994
MBD5	0,011	0,923	0,987
ST3GAL5	0,011	0,974	0,998
TTC27	0,011	0,936	0,991
ZNF562	0,010	0,915	0,984
RTN3	0,010	0,957	0,995
RGL2	0,010	0,950	0,993
COL4A5	0,010	0,960	0,995
CD84	0,010	0,974	0,998
MTA3	0,010	0,940	0,992
MAP4K4	0,010	0,936	0,991
TOP1	0,010	0,948	0,993
SIAH2	0,010	0,953	0,994
TAF5L	0,010	0,978	0,998
EIF3E	0,010	0,973	0,998
ATP11C	0,010	0,969	0,997
NAV1	0,010	0,974	0,998
MAVS	0,010	0,967	0,996
EARS2	0,009	0,956	0,994
PARP1	0,009	0,938	0,992
PARP11	0,009	0,944	0,993
RPL27	0,009	0,960	0,995
SMYD5	0,009	0,993	1,000
ZNF808	0,009	0,953	0,994
XPA	0,009	0,945	0,993
DENR	0,009	0,936	0,991
PBX3	0,009	0,944	0,993
GNA11	0,009	0,964	0,995
SLC12A7	0,009	0,964	0,995
RPS28	0,009	0,975	0,998
MSH6	0,009	0,936	0,991
ZNF569	0,008	0,965	0,996
M6PR	0,008	0,946	0,993
SEPSECS	0,008	0,962	0,995
MED13L	0,008	0,960	0,995
TMED10	0,008	0,950	0,993
ABHD2	0,008	0,964	0,995
SON	0,008	0,946	0,993
EIF1AX	0,008	0,963	0,995
CD47	0,008	0,961	0,995
ARHGAP23	0,008	0,973	0,998
SEC61A2	0,008	0,999	1,000
DNAJC10	0,008	0,962	0,995
ZNF236	0,008	0,979	0,998
HDAC7	0,008	0,970	0,997

USP7	0,008	0,949	0,993
TMEM33	0,008	0,961	0,995
RBM15	0,007	0,963	0,995
PDCD6IP	0,007	0,948	0,993
PDZD8	0,007	0,950	0,993
BRPF3	0,007	0,975	0,998
DNAJB12	0,007	0,957	0,995
ATG7	0,007	0,998	1,000
MDM4	0,007	0,967	0,996
FOCAD	0,007	0,964	0,995
COPB1	0,007	0,967	0,996
GTF2IRD2B	0,007	0,962	0,995
DHX35	0,007	0,967	0,996
TRAPPC11	0,007	0,950	0,993
SPAG16	0,007	0,971	0,998
ZBTB10	0,007	0,967	0,996
KLF12	0,007	0,957	0,995
APOBEC3C	0,007	0,972	0,998
AMIGO1	0,007	0,964	0,995
GEMIN5	0,007	0,955	0,994
NUP54	0,007	0,964	0,995
RAB5A	0,007	0,953	0,994
COG6	0,007	0,977	0,998
SUMO2	0,007	0,960	0,995
HNRNPD	0,007	0,953	0,994
BRCC3	0,006	0,963	0,995
MDN1	0,006	0,967	0,996
UBXN4	0,006	0,969	0,997
RPS7	0,006	0,977	0,998
BBS10	0,006	0,979	0,998
HMGXB3	0,006	0,971	0,997
RIOK1	0,006	0,967	0,996
ATP8B1	0,006	0,971	0,997
NIBAN2	0,006	0,978	0,998
PES1	0,006	0,974	0,998
HABP4	0,006	0,966	0,996
EPHA4	0,006	0,982	0,998
MMP14	0,006	0,983	0,998
ZNF566	0,005	0,969	0,997
MAML1	0,005	0,979	0,998
SOBP	0,005	0,974	0,998
HARS2	0,005	0,998	1,000
MYLK	0,005	0,986	0,999
ARL8A	0,005	0,986	0,999
NTRK2	0,005	0,983	0,998
THUMPD1	0,005	0,968	0,997
MOB3C	0,005	0,974	0,998
DHX36	0,005	0,962	0,995
ARFGEF1	0,005	0,967	0,996
DUT	0,005	0,968	0,997
ARL8B	0,005	0,971	0,998
SYAP1	0,005	0,971	0,997
DYNLT3	0,005	0,986	1,000

FAM217B	0,005	0,978	0,998
ZNF660	0,005	0,978	0,998
VCPIP1	0,005	0,971	0,998
TRIO	0,004	0,979	0,998
STRADB	0,004	0,982	0,998
PUM2	0,004	0,968	0,997
PIK3C3	0,004	0,976	0,998
KLHL24	0,004	0,988	1,000
ANGEL2	0,004	0,981	0,998
AP1G1	0,004	0,970	0,997
POLD3	0,004	0,983	0,998
RCBTB1	0,004	0,981	0,998
RAB2A	0,004	0,983	0,998
HEATR5A	0,004	0,972	0,998
B4GALT2	0,004	0,984	0,998
BNIP2	0,003	0,982	0,998
DDX41	0,003	0,982	0,998
AXL	0,003	0,989	1,000
CWF19L1	0,003	0,974	0,998
GBP2	0,003	0,990	1,000
ETNK1	0,003	0,988	1,000
ZC3H14	0,003	0,977	0,998
CTSS	0,003	0,991	1,000
PAPOLG	0,003	0,979	0,998
WAC	0,003	0,974	0,998
FAM172A	0,003	0,982	0,998
SMNDC1	0,003	0,978	0,998
RAB3GAP1	0,003	0,979	0,998
RAB3GAP2	0,003	0,976	0,998
COPB2	0,003	0,981	0,998
CHD9	0,003	0,985	0,999
TNRC6A	0,003	0,988	1,000
YTHDF2	0,003	0,978	0,998
EXOC3	0,003	0,986	0,999
KIF3A	0,003	0,983	0,998
LDAH	0,003	0,987	1,000
DDX19B	0,003	0,994	1,000
RAPH1	0,002	0,991	1,000
PIBF1	0,002	0,988	1,000
MSANTD3	0,002	0,989	1,000
MT1E	0,002	0,996	1,000
AGO4	0,002	0,988	1,000
TNPO1	0,002	0,992	1,000
TSPO	0,002	0,994	1,000
KDM4B	0,002	0,993	1,000
NTN1	0,002	0,996	1,000
NCK1	0,002	0,993	1,000
SCAF4	0,001	0,995	1,000
ZNF558	0,001	0,996	1,000
RNF187	0,001	0,994	1,000
METTL14	0,001	0,992	1,000
WSB1	0,001	0,994	1,000
RAB7A	0,001	0,993	1,000

MED23	0,001	0,994	1,000
EXOSC10	0,001	0,994	1,000
UPF2	0,001	0,995	1,000
NOL8	0,001	0,995	1,000
PPP2CA	0,001	0,996	1,000
MME	0,001	0,999	1,000
RALGAPB	0,001	0,996	1,000
NRAS	0,000	0,998	1,000
LMAN2	0,000	0,998	1,000
SS18	0,000	0,997	1,000
RAB11FIP5	0,000	0,999	1,000
SLC3A2	0,000	0,998	1,000
WDR59	0,000	0,999	1,000
POP5	0,000	0,999	1,000
RCOR1	0,000	0,999	1,000
GOLGA6L4	0,000	1,000	1,000
LRRC47	0,000	0,999	1,000
PRDM2	0,000	1,000	1,000
PMS1	0,000	0,999	1,000
GRB10	0,000	0,999	1,000
AGGF1	0,000	0,998	1,000
TMEM184B	0,000	0,998	1,000
MTF2	0,000	0,997	1,000
**Down-regulated**			
VPS26A	-0,001	0,997	1,000
CREB1	-0,001	0,996	1,000
VAPB	-0,001	0,996	1,000
TRPM7	-0,001	0,996	1,000
PREB	-0,001	0,996	1,000
SMAD1	-0,001	0,999	1,000
DCAF5	-0,001	0,995	1,000
KIF2A	-0,001	0,995	1,000
FBXL3	-0,001	0,996	1,000
TMEM109	-0,001	0,996	1,000
IFNGR2	-0,001	0,995	1,000
ILF2	-0,001	0,993	1,000
MCMBP	-0,001	0,992	1,000
CASP3	-0,001	0,996	1,000
PEF1	-0,001	0,994	1,000
RAB6A	-0,001	0,990	1,000
GSR	-0,001	0,992	1,000
ZNF317	-0,001	0,992	1,000
TNNI3	-0,001	0,997	1,000
PDE8A	-0,001	0,992	1,000
PTPN12	-0,002	0,993	1,000
TRAPPC2	-0,002	0,998	1,000
RARS2	-0,002	0,988	1,000
MFSD4B	-0,002	0,994	1,000
KIAA1191	-0,002	0,986	0,999
SPG7	-0,002	0,992	1,000
ICA1L	-0,002	0,989	1,000
NR1H2	-0,002	0,989	1,000
XRN1	-0,002	0,988	1,000

ZPR1	-0,002	0,995	1,000
FNBP1L	-0,002	0,988	1,000
VPS35	-0,002	0,988	1,000
LEPROT	-0,002	0,985	0,999
SLC44A2	-0,002	0,990	1,000
PKN2	-0,003	0,987	1,000
FAM228B	-0,003	0,994	1,000
PRMT5	-0,003	0,983	0,998
CCDC90B	-0,003	0,987	1,000
FKBP14	-0,003	0,986	0,999
SEPTIN7	-0,003	0,990	1,000
SLC38A7	-0,003	0,987	1,000
PNRC2	-0,003	0,989	1,000
ARFGEF2	-0,003	0,981	0,998
SH3RF2	-0,003	0,988	1,000
TRMT5	-0,003	0,982	0,998
NCAM1	-0,003	0,989	1,000
USP14	-0,003	0,975	0,998
PIEZO1	-0,003	0,991	1,000
DIS3L	-0,004	0,975	0,998
CKAP2	-0,004	0,984	0,999
RAB1B	-0,004	0,984	0,999
RALGAPA1	-0,004	0,978	0,998
PCYT1A	-0,004	0,979	0,998
ERI3	-0,004	0,972	0,998
GABRE	-0,004	0,991	1,000
GALT	-0,004	0,976	0,998
ZNF671	-0,004	0,977	0,998
ATP6V1C1	-0,004	0,975	0,998
ADI1	-0,004	0,976	0,998
SKIV2L	-0,005	0,981	0,998
ZNF420	-0,005	0,981	0,998
RALY	-0,005	0,974	0,998
GSS	-0,005	0,966	0,996
PABPN1	-0,005	0,977	0,998
KIF1B	-0,005	0,980	0,998
TMEM41A	-0,005	0,965	0,996
MARCH8	-0,005	0,972	0,998
KAT6A	-0,005	0,974	0,998
NUP62	-0,005	0,976	0,998
GGA2	-0,005	0,975	0,998
CREBRF	-0,005	0,982	0,998
PCDHGB7	-0,005	0,981	0,998
ZFP36	-0,005	0,989	1,000
DARS2	-0,005	0,961	0,995
ATF2	-0,005	0,979	0,998
PPME1	-0,005	0,962	0,995
SLC38A6	-0,005	0,982	0,998
PROSER1	-0,005	0,964	0,995
YWHAG	-0,005	0,971	0,998
LHFPL2	-0,006	0,981	0,998
TFE3	-0,006	0,979	0,998
ERCC4	-0,006	0,996	1,000

TSTD3	-0,006	0,979	0,998
KLC1	-0,006	0,971	0,998
RPS6KC1	-0,006	0,965	0,996
HDAC1	-0,006	0,955	0,994
EPN2	-0,006	0,981	0,998
CHMP3	-0,006	0,958	0,995
MLLT10	-0,006	0,969	0,997
PYCR2	-0,006	0,963	0,995
DISC1	-0,006	0,976	0,998
MYH11	-0,006	0,985	0,999
NUBPL	-0,006	0,973	0,998
HAUS2	-0,006	0,997	1,000
ANKRA2	-0,006	0,990	1,000
PIH1D1	-0,006	0,972	0,998
INPP5B	-0,006	0,968	0,997
PHB2	-0,007	0,960	0,995
IPO11	-0,007	0,963	0,995
NDFIP2	-0,007	0,970	0,997
ACSS3	-0,007	0,963	0,995
CAPRIN2	-0,007	0,946	0,993
PUS7L	-0,007	0,972	0,998
ZBTB11	-0,007	0,962	0,995
EXD2	-0,007	0,937	0,992
PHF14	-0,007	0,959	0,995
TIMP3	-0,007	0,978	0,998
NXF1	-0,008	0,965	0,996
CLEC16A	-0,008	0,961	0,995
LDLRAD4	-0,008	0,963	0,995
SETD5	-0,008	0,969	0,997
SPTLC2	-0,008	0,961	0,995
ZNF586	-0,008	0,959	0,995
IPO9	-0,008	0,947	0,993
C11orf54	-0,008	0,972	0,998
MAGI2	-0,008	0,950	0,993
KIAA0100	-0,008	0,958	0,995
UBXN1	-0,008	0,958	0,995
UBN1	-0,008	0,967	0,996
ERMARD	-0,008	0,953	0,994
ZNF250	-0,008	0,953	0,994
CARD8	-0,008	0,952	0,994
SEPHS1	-0,009	0,977	0,998
DYRK1A	-0,009	0,948	0,993
SLC15A4	-0,009	0,959	0,995
UBB	-0,009	0,959	0,995
SMG7	-0,009	0,957	0,995
FAM174A	-0,009	0,995	1,000
ZNF106	-0,009	0,978	0,998
ABHD4	-0,009	0,955	0,994
AP1B1	-0,009	0,964	0,995
HNRNPDL	-0,009	0,946	0,993
DENND4C	-0,009	0,951	0,993
ENSA	-0,009	0,933	0,991
EPM2AIP1	-0,010	0,953	0,994

ANKRD44	-0,010	0,957	0,995
SLC25A38	-0,010	0,964	0,995
ZDHHC20	-0,010	0,959	0,995
MAGEF1	-0,010	0,935	0,991
COPS7B	-0,010	0,949	0,993
MTAP	-0,010	0,960	0,995
MYO9A	-0,010	0,931	0,990
DOCK3	-0,010	0,969	0,997
RIF1	-0,010	0,949	0,993
WDR47	-0,010	0,961	0,995
RNF7	-0,010	0,996	1,000
NAP1L4	-0,010	0,928	0,989
PGAM1	-0,010	0,934	0,991
UMAD1	-0,010	0,977	0,998
CANT1	-0,010	0,947	0,993
GABPB1	-0,010	0,979	0,998
WDR61	-0,010	0,945	0,993
FZD3	-0,010	0,977	0,998
AGT	-0,011	0,952	0,994
CCDC6	-0,011	0,948	0,993
ASCC3	-0,011	0,947	0,993
CLIC1	-0,011	0,957	0,995
WFS1	-0,011	0,959	0,995
AFF3	-0,011	0,943	0,993
STT3B	-0,011	0,945	0,993
EPHB4	-0,011	0,955	0,994
TOP2B	-0,011	0,930	0,990
MTA2	-0,011	0,948	0,993
GCFC2	-0,012	0,934	0,991
LMBR1L	-0,012	0,950	0,993
SLC39A6	-0,012	0,928	0,989
DENND11	-0,012	0,933	0,991
PICALM	-0,012	0,942	0,992
GNE	-0,012	0,905	0,981
MIER3	-0,012	0,950	0,993
EHBP1	-0,012	0,954	0,994
FP565260.6	-0,012	0,927	0,989
POLR3B	-0,012	0,974	0,998
ZNF711	-0,012	0,959	0,995
FRMD4A	-0,013	0,963	0,995
BIRC6	-0,013	0,925	0,988
WAPL	-0,013	0,906	0,982
NOL9	-0,013	0,938	0,992
SDE2	-0,013	0,969	0,997
CIAO1	-0,013	0,890	0,978
KIAA1109	-0,013	0,918	0,985
FBXO32	-0,013	0,968	0,997
ZNF782	-0,013	0,934	0,991
CSDE1	-0,013	0,937	0,992
HSF1	-0,013	0,942	0,993
REPIN1	-0,013	0,933	0,991
LAMP1	-0,013	0,926	0,989
TRA2B	-0,013	0,925	0,988

KCTD20	-0,014	0,925	0,989
GOLT1B	-0,014	0,951	0,993
ACER3	-0,014	0,947	0,993
TUBGCP5	-0,014	0,901	0,980
SMCHD1	-0,014	0,936	0,991
ALDH1L2	-0,014	0,949	0,993
WDR55	-0,014	0,931	0,990
SEPTIN4	-0,014	0,942	0,993
ZFAND3	-0,014	0,927	0,989
FAM193A	-0,014	0,942	0,993
PIK3CA	-0,014	0,928	0,989
UGGT1	-0,014	0,900	0,980
RGPD3	-0,014	0,924	0,988
PRPF19	-0,014	0,943	0,993
ARRDC3	-0,014	0,959	0,995
MAP2K4	-0,015	0,928	0,989
TRAPPC9	-0,015	0,941	0,992
DIMT1	-0,015	0,967	0,996
NECAP1	-0,015	0,912	0,984
C11orf58	-0,015	0,904	0,981
NIN	-0,015	0,938	0,992
ILKAP	-0,015	0,919	0,986
KRT8	-0,015	0,965	0,996
FAM76B	-0,015	0,997	1,000
STN1	-0,015	0,938	0,992
FGD5	-0,015	0,947	0,993
PDS5A	-0,015	0,918	0,985
BOD1	-0,015	0,953	0,994
PPP2R5C	-0,015	0,884	0,977
GPCPD1	-0,015	0,929	0,990
ITPR3	-0,015	0,957	0,995
BCKDHB	-0,015	0,970	0,997
HTT	-0,015	0,940	0,992
HNRNPC	-0,015	0,908	0,983
LAPTM4B	-0,015	0,948	0,993
ASB3	-0,015	0,994	1,000
TTC14	-0,015	0,923	0,987
MFGE8	-0,015	0,941	0,992
CARHSP1	-0,016	0,953	0,994
KIAA1143	-0,016	0,909	0,983
PPP1R8	-0,016	0,954	0,994
GATAD2A	-0,016	0,935	0,991
WIPI2	-0,016	0,891	0,978
OMA1	-0,016	0,944	0,993
CARF	-0,016	0,969	0,997
UBR4	-0,016	0,948	0,993
LSS	-0,016	0,951	0,993
ACTN2	-0,016	0,954	0,994
GPATCH2L	-0,016	0,899	0,980
LSM14B	-0,016	0,911	0,983
RAB9A	-0,016	0,962	0,995
PBRM1	-0,016	0,889	0,978
OSER1	-0,016	0,995	1,000

SFMBT1	-0,016	0,909	0,983
TRIM52	-0,016	0,921	0,987
IFIT1	-0,016	0,948	0,993
EIF1	-0,017	0,907	0,982
VRK1	-0,017	0,944	0,993
RPL7	-0,017	0,954	0,994
RPS25	-0,017	0,943	0,993
TSPAN9	-0,017	0,945	0,993
MXD1	-0,017	0,960	0,995
HSPA14	-0,017	0,930	0,990
HEATR1	-0,017	0,914	0,984
PSMD10	-0,017	0,941	0,992
NAP1L1	-0,017	0,920	0,986
MAST4	-0,017	0,918	0,985
CASP10	-0,017	0,914	0,984
ZC3H11B	-0,017	0,913	0,984
MTMR14	-0,018	0,907	0,982
USP13	-0,018	0,939	0,992
NUCKS1	-0,018	0,887	0,978
TARSL2	-0,018	0,921	0,987
RGCC	-0,018	0,939	0,992
PAFAH1B1	-0,018	0,859	0,971
KEAP1	-0,018	0,913	0,984
BUB3	-0,018	0,956	0,994
ANP32A	-0,018	0,904	0,981
PPP1CA	-0,018	0,909	0,983
MITD1	-0,018	0,961	0,995
SMG5	-0,018	0,928	0,989
CCDC91	-0,018	0,908	0,983
JTB	-0,018	0,914	0,984
DDX27	-0,018	0,902	0,980
TRMT1L	-0,018	0,990	1,000
KDM5C	-0,018	0,927	0,989
DBNL	-0,019	0,911	0,983
RNF2	-0,019	0,914	0,984
ANKRD10	-0,019	0,920	0,986
TXLNB	-0,019	0,952	0,994
GCLM	-0,019	0,922	0,987
RBMX2	-0,019	0,943	0,993
WDR70	-0,019	0,927	0,989
PGAM4	-0,019	0,885	0,977
DCTN5	-0,019	0,876	0,976
ZNF701	-0,019	0,950	0,993
ARL5A	-0,019	0,931	0,990
IARS2	-0,019	0,855	0,970
WDR13	-0,019	0,916	0,985
SNRPB	-0,019	0,910	0,983
KANSL1L	-0,019	0,881	0,977
TBRG1	-0,020	0,860	0,971
DHX30	-0,020	0,900	0,980
ABCF1	-0,020	0,900	0,980
BEND7	-0,020	0,912	0,984
COMMD2	-0,020	0,913	0,984

ELF2	-0,020	0,886	0,978
USF1	-0,020	0,889	0,978
C6orf47	-0,020	0,917	0,985
ABHD5	-0,020	0,933	0,991
RTL6	-0,020	0,900	0,980
IGBP1	-0,020	0,914	0,984
GDI1	-0,020	0,904	0,981
SNAP23	-0,020	0,977	0,998
FAM193B	-0,020	0,905	0,981
EFNB1	-0,020	0,931	0,990
PRPF38B	-0,021	0,885	0,978
FAM20B	-0,021	0,818	0,956
HDGFL3	-0,021	0,865	0,973
RAD21	-0,021	0,858	0,971
PHIP	-0,021	0,898	0,980
KCTD12	-0,021	0,919	0,985
CEP97	-0,021	0,973	0,998
ZNF121	-0,021	0,854	0,970
RCN2	-0,021	0,891	0,978
PRKAR2A	-0,021	0,862	0,972
PEX19	-0,021	0,896	0,979
SYNDIG1	-0,021	0,908	0,983
ZBTB41	-0,021	0,938	0,992
FABP4	-0,021	0,972	0,998
POLR3A	-0,022	0,871	0,974
SUCO	-0,022	0,889	0,978
USF3	-0,022	0,877	0,976
ARID1A	-0,022	0,934	0,991
ZNF606	-0,022	0,875	0,976
TENT5A	-0,022	0,874	0,976
OBSCN	-0,022	0,951	0,993
DIS3L2	-0,022	0,867	0,973
MED6	-0,023	0,917	0,985
GLIPR1	-0,023	0,927	0,989
EXOSC3	-0,023	0,902	0,980
RPL21	-0,023	0,914	0,984
ITCH	-0,023	0,847	0,967
TOR1B	-0,023	0,895	0,979
APOL1	-0,023	0,940	0,992
PCDH12	-0,023	0,889	0,978
TMOD1	-0,023	0,918	0,985
TTC17	-0,023	0,854	0,970
PWP1	-0,023	0,887	0,978
NAA16	-0,023	0,889	0,978
CNTLN	-0,023	0,944	0,993
GNA13	-0,023	0,887	0,978
SLC9B2	-0,023	0,900	0,980
ADPGK	-0,023	0,872	0,975
SAMD8	-0,024	0,876	0,976
CUL3	-0,024	0,840	0,965
PAQR3	-0,024	0,899	0,980
RUNDC1	-0,024	0,870	0,974
CDIPT	-0,024	0,885	0,977

AVL9	-0,024	0,854	0,970
IARS	-0,024	0,811	0,954
CHKA	-0,024	0,907	0,982
VAT1	-0,024	0,909	0,983
EML1	-0,024	0,824	0,958
DNAJC1	-0,024	0,894	0,979
PRMT3	-0,024	0,896	0,979
EMC1	-0,024	0,882	0,977
ATOX1	-0,024	0,881	0,977
CTTNBP2NL	-0,025	0,899	0,980
DAAM1	-0,025	0,864	0,973
LYPLAL1	-0,025	0,932	0,991
SPATA6	-0,025	0,893	0,979
NOC2L	-0,025	0,882	0,977
MAPK14	-0,025	0,853	0,970
TAF2	-0,025	0,858	0,970
ACO1	-0,025	0,889	0,978
ZNF700	-0,025	0,926	0,989
CCSER2	-0,025	0,876	0,976
OTUD1	-0,025	0,947	0,993
ZFP62	-0,026	0,926	0,989
SNX25	-0,026	0,849	0,968
PEX11B	-0,026	0,886	0,978
TBC1D14	-0,026	0,843	0,966
RELA	-0,026	0,883	0,977
WNK2	-0,026	0,938	0,992
TMEM131	-0,026	0,837	0,963
IL32	-0,026	0,940	0,992
SCFD2	-0,026	0,867	0,973
POLR3F	-0,026	0,940	0,992
ESYT2	-0,026	0,837	0,963
SRSF1	-0,026	0,895	0,979
FLCN	-0,026	0,895	0,979
IMPAD1	-0,026	0,833	0,962
ZNF329	-0,026	0,896	0,979
BLOC1S5	-0,026	0,943	0,993
DDX18	-0,026	0,857	0,970
CHM	-0,026	0,870	0,974
TIMM10B	-0,026	0,857	0,970
FAM91A1	-0,026	0,891	0,978
KATNBL1	-0,026	0,884	0,977
STAG2	-0,027	0,896	0,979
PHPT1	-0,027	0,877	0,976
VPS28	-0,027	0,879	0,977
GID4	-0,027	0,834	0,963
SMAD5	-0,027	0,878	0,977
CHMP2B	-0,027	0,871	0,974
RC3H2	-0,027	0,830	0,962
SZRD1	-0,027	0,873	0,975
ZDHHC5	-0,027	0,840	0,965
GOLGA5	-0,027	0,837	0,963
CCDC186	-0,027	0,866	0,973
FAM199X	-0,027	0,829	0,961

ZNF761	-0,028	0,902	0,980
MBD4	-0,028	0,839	0,964
C3orf38	-0,028	0,865	0,973
JAK1	-0,028	0,817	0,955
GTF2H2C	-0,028	0,866	0,973
PHKA1	-0,028	0,874	0,975
NSRP1	-0,028	0,840	0,965
COPS7A	-0,029	0,821	0,958
FAM76A	-0,029	0,840	0,965
USP39	-0,029	0,810	0,954
PDGFRB	-0,029	0,915	0,984
EIF4A2	-0,029	0,887	0,978
MAPK8	-0,029	0,856	0,970
HIVEP1	-0,029	0,822	0,958
CDCA7L	-0,029	0,879	0,977
TGS1	-0,029	0,893	0,979
R3HCC1L	-0,029	0,924	0,988
BOC	-0,029	0,907	0,982
CD44	-0,029	0,929	0,990
HEXA	-0,029	0,806	0,953
TMED2	-0,029	0,861	0,971
CD99L2	-0,029	0,860	0,971
ZNF587B	-0,029	0,864	0,973
CCPG1	-0,029	0,870	0,974
ATAD2	-0,029	0,875	0,976
ZZEF1	-0,029	0,884	0,977
ELMOD2	-0,029	0,902	0,980
TRIM2	-0,029	0,840	0,965
PDIA3	-0,030	0,870	0,974
ZNF875	-0,030	0,864	0,973
MYBPC3	-0,030	0,918	0,985
SPATS2L	-0,030	0,776	0,945
TP53I11	-0,030	0,915	0,984
SMARCA4	-0,030	0,888	0,978
DNAJB14	-0,030	0,901	0,980
DENND5B	-0,030	0,856	0,970
SS18L1	-0,030	0,848	0,968
BRWD1	-0,030	0,860	0,971
NFIC	-0,030	0,913	0,984
ZNF655	-0,030	0,823	0,958
FBXO28	-0,030	0,854	0,970
ZNF800	-0,030	0,845	0,967
JAZF1	-0,030	0,945	0,993
DIP2C	-0,030	0,871	0,974
TCEAL3	-0,030	0,868	0,974
PTEN	-0,030	0,885	0,977
MAP3K20	-0,030	0,891	0,978
HECTD1	-0,031	0,788	0,948
ZNF614	-0,031	0,931	0,990
PREPL	-0,031	0,889	0,978
NUCB1	-0,031	0,883	0,977
RPP14	-0,031	0,836	0,963
DNMBP	-0,031	0,881	0,977

Protein coding	BCL2L1	-0,031	0,879	0,977
	TFG	-0,031	0,788	0,948
	MYDGF	-0,031	0,905	0,981
	MACF1	-0,031	0,841	0,965
	ATAD2B	-0,031	0,819	0,956
	NARS2	-0,031	0,883	0,977
	PLEKHA2	-0,031	0,844	0,967
	MCU	-0,031	0,800	0,950
	KIAA1841	-0,031	0,867	0,973
	FAM114A2	-0,032	0,851	0,969
	GGNBP2	-0,032	0,797	0,949
	EMP1	-0,032	0,900	0,980
	UTP23	-0,032	0,930	0,990
	PLEKHA5	-0,032	0,848	0,968
	EWSR1	-0,032	0,799	0,950
	GATA4	-0,032	0,870	0,974
	GNB2	-0,032	0,879	0,977
	ERCC6L2	-0,032	0,763	0,941
	GABARAPL2	-0,032	0,783	0,946
	ADGRE5	-0,032	0,891	0,978
	ROBO4	-0,032	0,892	0,978
	YTHDF3	-0,032	0,816	0,954
	GNAI3	-0,032	0,796	0,948
	CCDC66	-0,032	0,832	0,962
	C15orf40	-0,032	0,839	0,964
	LAMC1	-0,032	0,853	0,970
	NCOA5	-0,032	0,857	0,970
	KLHL2	-0,033	0,845	0,967
	CWF19L2	-0,033	0,824	0,958
	WIPI1	-0,033	0,855	0,970
	MVP	-0,033	0,888	0,978
	LGALS1	-0,033	0,908	0,982
	CLTC	-0,033	0,777	0,945
	PRKACB	-0,033	0,804	0,951
	TMEM41B	-0,033	0,827	0,960
	SRFBP1	-0,033	0,883	0,977
	CCDC127	-0,033	0,794	0,948
	RNF139	-0,033	0,838	0,964
	HIF3A	-0,033	0,928	0,989
	RPS27L	-0,033	0,869	0,974
	GINM1	-0,034	0,884	0,977
	KLHDC3	-0,034	0,831	0,962
	PARP2	-0,034	0,811	0,954
	WASHC4	-0,034	0,866	0,973
	MINDY2	-0,034	0,848	0,968
	CD151	-0,034	0,868	0,974
	MSH2	-0,034	0,863	0,972
	EIF3H	-0,034	0,772	0,944
	AMZ2	-0,034	0,830	0,962
	CCNC	-0,034	0,845	0,967
	CSGALNACT1	-0,034	0,844	0,967
	PANK2	-0,034	0,835	0,963
	TBC1D15	-0,034	0,830	0,962

LCOR	-0,034	0,734	0,932
ZNF550	-0,034	0,831	0,962
CGGBP1	-0,035	0,812	0,954
RBBP7	-0,035	0,721	0,927
KIAA0319L	-0,035	0,846	0,967
PHF11	-0,035	0,866	0,973
KDM3A	-0,035	0,773	0,944
GTF2IRD2	-0,035	0,820	0,957
METTL3	-0,035	0,697	0,918
NCOR2	-0,035	0,875	0,976
SMARCAD1	-0,035	0,834	0,963
SOCS2	-0,035	0,844	0,967
WDR20	-0,035	0,805	0,952
PHF6	-0,035	0,822	0,958
TSR1	-0,035	0,778	0,945
RTCB	-0,035	0,735	0,932
ZNF654	-0,036	0,858	0,971
NSMCE3	-0,036	0,813	0,954
STX7	-0,036	0,813	0,954
CRKL	-0,036	0,769	0,942
C1R	-0,036	0,902	0,980
WDR11	-0,036	0,796	0,948
DTWD1	-0,036	0,783	0,946
KAT6B	-0,036	0,761	0,939
IPMK	-0,036	0,872	0,975
RALGAPA2	-0,036	0,846	0,967
C5orf24	-0,036	0,811	0,954
FBXO30	-0,036	0,819	0,956
SF1	-0,036	0,835	0,963
BAZ2B	-0,036	0,766	0,941
ANKRD17	-0,036	0,736	0,932
NRIP1	-0,036	0,869	0,974
PNISR	-0,036	0,790	0,948
UBE3A	-0,036	0,779	0,945
CNOT11	-0,037	0,764	0,941
ZNF138	-0,037	0,853	0,970
G3BP2	-0,037	0,741	0,933
COP1	-0,037	0,771	0,943
NPEPPS	-0,037	0,787	0,948
PAK2	-0,038	0,812	0,954
ZNF227	-0,038	0,845	0,967
SUMO3	-0,038	0,706	0,922
GIT2	-0,038	0,805	0,952
MMS22L	-0,038	0,855	0,970
FARP1	-0,038	0,849	0,968
DIAPH2	-0,038	0,846	0,967
LIN52	-0,038	0,845	0,967
ESF1	-0,038	0,826	0,960
DHX29	-0,038	0,822	0,958
NOLC1	-0,038	0,768	0,942
CAPZA1	-0,038	0,835	0,963
CRTC3	-0,038	0,855	0,970
EHD1	-0,038	0,881	0,977

MTOR	-0,038	0,821	0,958
EEF1A2	-0,038	0,893	0,979
LUC7L3	-0,038	0,777	0,945
TRIM32	-0,038	0,792	0,948
TCEAL9	-0,038	0,855	0,970
TMED9	-0,039	0,821	0,957
NUTF2	-0,039	0,753	0,937
ZNF418	-0,039	0,839	0,964
MYOM3	-0,039	0,902	0,980
SCMH1	-0,039	0,781	0,946
ZC3H8	-0,039	0,811	0,954
PMPCA	-0,039	0,804	0,952
TYW3	-0,039	0,845	0,967
TLCD5	-0,039	0,849	0,968
MYO5A	-0,039	0,771	0,943
DMXL2	-0,039	0,811	0,954
NUP98	-0,039	0,734	0,931
CHD4	-0,039	0,810	0,954
IST1	-0,039	0,652	0,899
RABEP1	-0,039	0,748	0,936
HIP1	-0,039	0,854	0,970
ZC3H6	-0,040	0,804	0,952
PCNP	-0,040	0,799	0,950
RMC1	-0,040	0,815	0,954
SH3PXD2A	-0,040	0,869	0,974
MRPL43	-0,040	0,803	0,951
VAMP4	-0,040	0,800	0,950
BSG	-0,040	0,861	0,971
SETD3	-0,040	0,757	0,938
ELK4	-0,040	0,791	0,948
CHD1	-0,040	0,876	0,976
ANK1	-0,040	0,915	0,984
MRFAP1L1	-0,040	0,759	0,939
ZNF347	-0,040	0,782	0,946
DIAPH1	-0,040	0,865	0,973
OTULIN	-0,040	0,797	0,949
SF3A3	-0,040	0,766	0,941
RAE1	-0,040	0,764	0,941
VPS45	-0,040	0,812	0,954
SNX11	-0,040	0,825	0,959
ANXA7	-0,041	0,729	0,930
RNPS1	-0,041	0,791	0,948
STK3	-0,041	0,780	0,946
YME1L1	-0,041	0,769	0,942
MZT1	-0,041	0,865	0,973
POLR1B	-0,041	0,820	0,957
DPY19L2	-0,041	0,915	0,984
RAC1	-0,041	0,763	0,941
ZYG11B	-0,041	0,796	0,948
MYO1B	-0,041	0,779	0,945
PIKFYVE	-0,041	0,754	0,937
INTS10	-0,042	0,758	0,938
RPA1	-0,042	0,762	0,940

ORMDL1	-0,042	0,783	0,946
MAPKAPK5	-0,042	0,697	0,917
VEZF1	-0,042	0,670	0,907
SPRY1	-0,042	0,858	0,971
NPAT	-0,042	0,782	0,946
RNF11	-0,042	0,787	0,948
RALGPS1	-0,042	0,855	0,970
ISCA2	-0,042	0,819	0,956
FHOD3	-0,042	0,894	0,979
RRAGA	-0,042	0,694	0,917
POLD2	-0,042	0,836	0,963
TPGS2	-0,042	0,752	0,936
DNAJB4	-0,042	0,888	0,978
ZNF629	-0,042	0,844	0,967
SLC12A6	-0,042	0,765	0,941
WDR43	-0,043	0,795	0,948
ALS2	-0,043	0,815	0,954
HELQ	-0,043	0,856	0,970
PNN	-0,043	0,681	0,911
TRAP1	-0,043	0,826	0,959
USP28	-0,043	0,865	0,973
EIF3B	-0,043	0,759	0,939
PCBP4	-0,043	0,825	0,959
RNPEPL1	-0,043	0,860	0,971
C14orf28	-0,043	0,848	0,968
ASS1	-0,043	0,885	0,977
COPG2	-0,043	0,772	0,944
RNF144A	-0,043	0,832	0,962
HEXIM1	-0,043	0,785	0,947
TMTC3	-0,044	0,834	0,963
AAGAB	-0,044	0,803	0,951
MKRN2	-0,044	0,748	0,936
PPP1R7	-0,044	0,725	0,929
BTBD10	-0,044	0,798	0,949
PMEPA1	-0,044	0,870	0,974
KBTBD6	-0,044	0,787	0,948
RPL37A	-0,044	0,842	0,965
IKBKB	-0,044	0,805	0,952
TYW5	-0,044	0,808	0,953
STAT5A	-0,044	0,882	0,977
CNOT4	-0,044	0,711	0,923
HCFC2	-0,044	0,832	0,962
STARD4	-0,045	0,880	0,977
SRPRB	-0,045	0,769	0,942
ROCK2	-0,045	0,760	0,939
GPR22	-0,045	0,913	0,984
PPP1CB	-0,045	0,834	0,963
ZBTB44	-0,045	0,725	0,929
PIGC	-0,045	0,842	0,965
TSPAN31	-0,045	0,681	0,911
NPIPB4	-0,045	0,877	0,976
STT3A	-0,045	0,696	0,917
MIA2	-0,045	0,739	0,933

ZNF7	-0,045	0,757	0,938
NUP155	-0,045	0,822	0,958
PPP3CB	-0,045	0,681	0,911
ANKRD40	-0,045	0,777	0,945
BCLAF1	-0,045	0,809	0,953
ALCAM	-0,045	0,865	0,973
ZFY	-0,045	0,917	0,985
RASGRP1	-0,046	0,831	0,962
IKZF2	-0,046	0,869	0,974
SLC25A30	-0,046	0,854	0,970
KIAA0586	-0,046	0,723	0,928
GALNT1	-0,046	0,809	0,953
SP4	-0,046	0,765	0,941
DYRK2	-0,046	0,811	0,954
C4orf3	-0,047	0,800	0,950
CNPPD1	-0,047	0,755	0,937
PFKFB3	-0,047	0,922	0,987
NENF	-0,047	0,784	0,946
VEGFB	-0,047	0,815	0,954
TDP2	-0,047	0,729	0,930
LPP	-0,047	0,747	0,935
ANO5	-0,047	0,882	0,977
CAPN7	-0,047	0,763	0,941
FAM219A	-0,047	0,789	0,948
TWF1	-0,047	0,870	0,974
RABL3	-0,047	0,772	0,944
TAF15	-0,047	0,739	0,933
PI4KA	-0,047	0,832	0,962
USH2A	-0,047	0,873	0,975
TGM2	-0,047	0,841	0,965
WDR45	-0,047	0,723	0,928
NFIA	-0,047	0,734	0,931
NAA35	-0,047	0,664	0,905
MRPL19	-0,048	0,653	0,899
TOX4	-0,048	0,668	0,907
AP2M1	-0,048	0,720	0,926
NEMF	-0,048	0,722	0,928
EIF5B	-0,048	0,790	0,948
FBXW4	-0,048	0,806	0,953
LMBRD1	-0,048	0,718	0,925
RUNX1T1	-0,048	0,773	0,944
LUC7L	-0,048	0,778	0,945
SLC35B2	-0,048	0,696	0,917
C2CD5	-0,048	0,717	0,925
HAGH	-0,049	0,784	0,946
ATP6AP1	-0,049	0,741	0,933
SPR	-0,049	0,808	0,953
BPNT1	-0,049	0,756	0,937
TRAPPC4	-0,049	0,784	0,946
NF2	-0,049	0,803	0,951
ARHGAP10	-0,049	0,630	0,892
BORCS7	-0,049	0,817	0,955
SBF2	-0,049	0,623	0,889

GRAMD4	-0,049	0,819	0,956
EIF4E2	-0,049	0,621	0,888
RBBP5	-0,049	0,748	0,936
TASOR2	-0,049	0,700	0,919
MAPK1IP1L	-0,049	0,727	0,930
UBFD1	-0,049	0,749	0,936
CENPJ	-0,049	0,811	0,954
FIG4	-0,050	0,823	0,958
ZNF283	-0,050	0,775	0,944
FBXO21	-0,050	0,734	0,931
GALC	-0,050	0,737	0,933
USPL1	-0,050	0,775	0,944
PBXIP1	-0,050	0,817	0,955
CDC5L	-0,050	0,789	0,948
SFT2D1	-0,050	0,780	0,946
PHF21A	-0,050	0,740	0,933
PIK3R1	-0,050	0,856	0,970
ZNF17	-0,050	0,796	0,948
NSUN2	-0,050	0,666	0,905
ZNF100	-0,050	0,781	0,946
LRBA	-0,050	0,683	0,913
INTS11	-0,051	0,739	0,933
PALB2	-0,051	0,809	0,953
KAT8	-0,051	0,728	0,930
BET1	-0,051	0,796	0,948
PSMC4	-0,051	0,689	0,914
TARDBP	-0,051	0,675	0,908
TFDP1	-0,051	0,725	0,929
CPSF2	-0,051	0,687	0,914
TMEM39A	-0,051	0,649	0,899
CDC23	-0,051	0,771	0,943
ENOPH1	-0,051	0,792	0,948
PAXBP1	-0,051	0,721	0,927
NOA1	-0,051	0,788	0,948
CYB5R3	-0,052	0,777	0,945
CHMP5	-0,052	0,816	0,954
DNAJC24	-0,052	0,806	0,953
CPSF6	-0,052	0,690	0,915
GGA3	-0,052	0,778	0,945
ERBB4	-0,052	0,863	0,973
HMGB2	-0,052	0,827	0,960
TULP4	-0,052	0,700	0,918
ABHD13	-0,052	0,778	0,945
UBE3B	-0,052	0,781	0,946
DCTN3	-0,052	0,710	0,923
MFSD8	-0,052	0,793	0,948
TINF2	-0,052	0,716	0,925
DPP8	-0,052	0,652	0,899
BDP1	-0,052	0,681	0,911
ZFR	-0,053	0,684	0,913
DDX56	-0,053	0,729	0,930
LACC1	-0,053	0,821	0,957
NOC3L	-0,053	0,798	0,949

KCTD3	-0,053	0,733	0,931
SLMAP	-0,053	0,866	0,973
ZNF706	-0,053	0,691	0,915
GSTO1	-0,053	0,742	0,933
THADA	-0,053	0,675	0,908
JAG1	-0,053	0,795	0,948
DHCR7	-0,053	0,814	0,954
SNRPD3	-0,053	0,713	0,924
EIF2B1	-0,054	0,655	0,900
SC5D	-0,054	0,770	0,943
UBE2I	-0,054	0,713	0,924
ZNF26	-0,054	0,752	0,936
LARGE1	-0,054	0,817	0,955
RIC8B	-0,054	0,679	0,911
AKAP13	-0,054	0,813	0,954
GTF2E2	-0,054	0,753	0,937
RFX3	-0,054	0,799	0,950
NUP214	-0,054	0,756	0,937
UFL1	-0,054	0,836	0,963
COMMD8	-0,054	0,846	0,967
DUSP19	-0,055	0,771	0,943
UBR2	-0,055	0,641	0,896
TSPAN18	-0,055	0,853	0,970
NSA2	-0,055	0,800	0,950
RANGAP1	-0,055	0,784	0,946
GRPEL2	-0,055	0,787	0,948
PDIA6	-0,055	0,689	0,914
FEZ1	-0,055	0,763	0,941
DCTN4	-0,055	0,584	0,872
RBM41	-0,055	0,790	0,948
URI1	-0,055	0,631	0,892
HACE1	-0,055	0,682	0,912
ZRANB3	-0,055	0,795	0,948
PRKN	-0,055	0,778	0,945
IREB2	-0,055	0,611	0,886
ATF6	-0,056	0,594	0,879
CRYBG1	-0,056	0,848	0,968
KATNAL1	-0,056	0,719	0,925
GLTP	-0,056	0,695	0,917
POLR2E	-0,056	0,769	0,942
RGPD8	-0,056	0,683	0,913
CLIP1	-0,056	0,667	0,906
TMBIM1	-0,056	0,787	0,948
C6orf120	-0,056	0,809	0,953
CKS1B	-0,056	0,795	0,948
RGS3	-0,056	0,768	0,942
ZNF548	-0,056	0,729	0,930
FAM200B	-0,056	0,750	0,936
KDM6A	-0,056	0,766	0,941
KCNJ8	-0,056	0,689	0,914
SPG21	-0,056	0,582	0,872
NFYC	-0,056	0,738	0,933
LAIR1	-0,056	0,851	0,969

FIS1	-0,056	0,793	0,948
RGPD6	-0,057	0,684	0,913
DDX24	-0,057	0,695	0,917
ZNF432	-0,057	0,721	0,927
TTC13	-0,057	0,757	0,938
NUP133	-0,057	0,656	0,900
PLEKHO2	-0,057	0,809	0,953
TXLNG	-0,058	0,711	0,923
FPGT	-0,058	0,803	0,951
PDS5B	-0,058	0,680	0,911
THAP5	-0,058	0,717	0,925
PAAF1	-0,058	0,699	0,918
KLF7	-0,058	0,776	0,945
HIF1AN	-0,058	0,686	0,913
PREP	-0,058	0,608	0,885
PAIP2	-0,058	0,637	0,893
CMTR2	-0,058	0,717	0,925
NARS	-0,058	0,756	0,938
SECISBP2	-0,058	0,702	0,919
PATL1	-0,058	0,738	0,933
CRY1	-0,058	0,845	0,967
ARHGAP35	-0,059	0,758	0,938
DDR2	-0,059	0,763	0,941
RUFY1	-0,059	0,681	0,911
DMXL1	-0,059	0,659	0,902
ACSL3	-0,059	0,670	0,907
RNF168	-0,059	0,619	0,888
PUF60	-0,059	0,743	0,934
CCT4	-0,059	0,618	0,888
PCSK7	-0,059	0,823	0,958
ZNF224	-0,059	0,769	0,942
FBH1	-0,059	0,708	0,923
RBM8A	-0,059	0,668	0,907
ABCD4	-0,059	0,766	0,941
ZEB1	-0,059	0,746	0,935
RAB5B	-0,059	0,625	0,889
SLC25A51	-0,059	0,749	0,936
WDR1	-0,060	0,696	0,917
EED	-0,060	0,762	0,941
IRF2BPL	-0,060	0,741	0,933
UBA6	-0,060	0,792	0,948
BMT2	-0,060	0,830	0,962
UBE3C	-0,060	0,629	0,891
ZNF33A	-0,060	0,626	0,890
DCAF10	-0,060	0,668	0,907
THSD7A	-0,060	0,839	0,964
SMC1A	-0,060	0,733	0,931
VPS26C	-0,060	0,526	0,844
POLR3E	-0,060	0,643	0,897
LRPAP1	-0,060	0,736	0,932
SEH1L	-0,060	0,731	0,931
EFL1	-0,060	0,692	0,916
ACSL1	-0,061	0,878	0,977

HDAC4	-0,061	0,755	0,937
RTTN	-0,061	0,616	0,887
SLC6A8	-0,061	0,791	0,948
DCAF11	-0,061	0,623	0,889
CCDC88A	-0,061	0,546	0,855
ZNF326	-0,061	0,661	0,903
ETAA1	-0,061	0,747	0,936
MARCH2	-0,061	0,782	0,946
JMJD1C	-0,061	0,745	0,935
NPIPB13	-0,061	0,816	0,954
DNAJC5	-0,061	0,724	0,928
MICU1	-0,061	0,535	0,850
CRLF3	-0,061	0,708	0,923
SRSF10	-0,061	0,684	0,913
UBE2W	-0,061	0,811	0,954
FUCA2	-0,062	0,664	0,905
GBE1	-0,062	0,750	0,936
VSIR	-0,062	0,813	0,954
OARD1	-0,062	0,778	0,945
CSNK2A1	-0,062	0,558	0,858
RGPD5	-0,062	0,663	0,904
FAM49A	-0,062	0,739	0,933
GTF2H2	-0,062	0,754	0,937
RPN1	-0,062	0,671	0,908
HIPK1	-0,062	0,651	0,899
FYTTD1	-0,062	0,753	0,937
PPIL3	-0,062	0,753	0,937
SEC24B	-0,062	0,594	0,879
NUP35	-0,062	0,764	0,941
PTCD3	-0,062	0,751	0,936
DCUN1D4	-0,062	0,733	0,931
G2E3	-0,063	0,701	0,919
VPS72	-0,063	0,685	0,913
SCPEP1	-0,063	0,708	0,923
SLC46A3	-0,063	0,679	0,911
PRPSAP2	-0,063	0,693	0,917
CCAR1	-0,063	0,524	0,842
APC	-0,063	0,694	0,917
ZNF354B	-0,063	0,747	0,936
FUBP1	-0,063	0,694	0,917
SMU1	-0,063	0,545	0,855
METTL2B	-0,063	0,625	0,889
ZNF778	-0,063	0,681	0,911
IFIT5	-0,063	0,769	0,942
DDRGK1	-0,063	0,720	0,926
MRPS27	-0,064	0,585	0,873
FSBP	-0,064	0,787	0,948
BIRC2	-0,064	0,744	0,934
OSTF1	-0,064	0,685	0,913
HDAC8	-0,064	0,655	0,900
SMC6	-0,064	0,639	0,894
ORC2	-0,064	0,641	0,896
ECHS1	-0,064	0,760	0,939

UTP3	-0,064	0,704	0,921
METTL15	-0,065	0,674	0,908
TOGARAM1	-0,065	0,652	0,899
ARPC1A	-0,065	0,750	0,936
TTC30A	-0,065	0,741	0,933
ARHGAP24	-0,065	0,659	0,902
GUCY1B1	-0,065	0,678	0,911
ZNF765	-0,065	0,708	0,923
DCTN2	-0,065	0,601	0,883
DAG1	-0,065	0,772	0,944
APPL2	-0,065	0,699	0,918
PPP2R5E	-0,065	0,585	0,872
YKT6	-0,065	0,565	0,862
FLI1	-0,065	0,644	0,897
USP34	-0,065	0,670	0,907
TCHP	-0,065	0,705	0,921
TMED3	-0,066	0,716	0,925
CUEDC2	-0,066	0,726	0,929
CEPT1	-0,066	0,689	0,914
SMAD2	-0,066	0,590	0,877
ING5	-0,066	0,651	0,899
ADAM10	-0,066	0,636	0,893
DDX50	-0,066	0,717	0,925
HNRNPF	-0,066	0,635	0,893
ZNF3	-0,066	0,574	0,868
AC118549.1	-0,067	0,703	0,921
APEX1	-0,067	0,648	0,899
STK25	-0,067	0,659	0,902
MOK	-0,067	0,717	0,925
WDR41	-0,067	0,617	0,887
FBN1	-0,067	0,751	0,936
PACSIN2	-0,067	0,651	0,899
VAPA	-0,067	0,620	0,888
CAPZB	-0,067	0,651	0,899
CWC25	-0,067	0,679	0,911
TAF7	-0,067	0,620	0,888
RBM26	-0,067	0,564	0,862
WRNIP1	-0,067	0,562	0,860
TSTD2	-0,067	0,668	0,907
NCK2	-0,067	0,644	0,897
CALR	-0,067	0,716	0,925
ADH5	-0,068	0,752	0,936
UROD	-0,068	0,692	0,916
WLS	-0,068	0,569	0,865
PRPF4B	-0,068	0,623	0,889
NPC1	-0,068	0,646	0,899
PCNX1	-0,068	0,550	0,856
PKP4	-0,068	0,623	0,889
NAA20	-0,068	0,721	0,927
NDRG3	-0,068	0,619	0,888
SNX13	-0,068	0,724	0,928
AP4E1	-0,068	0,640	0,895
HAND2	-0,068	0,640	0,895

SNW1	-0,068	0,600	0,882
CRELD1	-0,069	0,731	0,931
FAM171A1	-0,069	0,763	0,941
HPRT1	-0,069	0,742	0,933
MORC2	-0,069	0,746	0,935
ARID1B	-0,069	0,691	0,915
HSPA8	-0,069	0,780	0,946
CMYA5	-0,069	0,876	0,976
CPNE8	-0,069	0,729	0,930
ZNF256	-0,069	0,689	0,914
AAR2	-0,069	0,650	0,899
PLOD3	-0,069	0,743	0,934
KXD1	-0,069	0,656	0,900
THAP6	-0,069	0,687	0,914
PARG	-0,069	0,694	0,917
EFTUD2	-0,069	0,658	0,901
TMEM173	-0,070	0,719	0,925
SGPP1	-0,070	0,729	0,930
KPNA1	-0,070	0,552	0,856
AQR	-0,070	0,563	0,861
AP3S2	-0,070	0,638	0,894
CNOT8	-0,070	0,594	0,879
MAP1A	-0,070	0,828	0,961
UBAC2	-0,070	0,523	0,842
AASDH	-0,070	0,705	0,921
DYRK4	-0,070	0,726	0,930
EEF2K	-0,070	0,769	0,942
GPATCH2	-0,070	0,636	0,893
RTL8C	-0,070	0,694	0,917
TMEM167A	-0,071	0,712	0,923
ARPP19	-0,071	0,688	0,914
PSMD3	-0,071	0,636	0,893
MMGT1	-0,071	0,632	0,892
ATRX	-0,071	0,654	0,899
NUDT16	-0,071	0,670	0,907
TMEM246	-0,071	0,751	0,936
TBC1D23	-0,071	0,766	0,941
FOSL2	-0,071	0,816	0,954
NADK	-0,071	0,767	0,942
FRS2	-0,071	0,635	0,893
SNAP29	-0,071	0,612	0,887
TMEM138	-0,072	0,666	0,905
STX2	-0,072	0,615	0,887
NFE2L1	-0,072	0,751	0,936
CSNK1D	-0,072	0,718	0,925
KIAA1586	-0,072	0,753	0,937
DNAJC13	-0,072	0,474	0,817
RNLS	-0,073	0,699	0,918
NUDT4B	-0,073	0,711	0,923
PDAP1	-0,073	0,579	0,870
TIFA	-0,073	0,673	0,908
UBR1	-0,073	0,610	0,886
ATG12	-0,073	0,577	0,870

SLC9A9	-0,073	0,724	0,928
UBE2R2	-0,073	0,542	0,853
RCOR3	-0,073	0,653	0,899
LMAN1	-0,073	0,715	0,925
SINHCAF	-0,073	0,691	0,915
ZC2HC1A	-0,073	0,708	0,923
MIDN	-0,073	0,747	0,936
NCOA6	-0,074	0,730	0,930
SLC35F6	-0,074	0,715	0,925
ZNF638	-0,074	0,613	0,887
TMEM184C	-0,074	0,566	0,862
BORCS5	-0,074	0,672	0,908
THSD1	-0,074	0,637	0,893
UXS1	-0,074	0,560	0,859
FAM32A	-0,074	0,632	0,892
NAA50	-0,074	0,627	0,890
ZNF790	-0,074	0,689	0,914
RNF146	-0,074	0,641	0,896
NSMAF	-0,074	0,502	0,830
SRP68	-0,074	0,594	0,879
NEMP2	-0,074	0,675	0,908
DOK6	-0,074	0,613	0,887
GPAT3	-0,074	0,700	0,919
PRPF4	-0,075	0,628	0,890
C5orf51	-0,075	0,643	0,897
PANK1	-0,075	0,752	0,936
FAM50A	-0,075	0,685	0,913
LYNX1	-0,075	0,763	0,941
TM9SF2	-0,075	0,604	0,883
TBCB	-0,075	0,539	0,850
RBM7	-0,075	0,699	0,918
LYN	-0,075	0,774	0,944
RELCH	-0,075	0,529	0,846
RRM2B	-0,075	0,719	0,926
MALT1	-0,075	0,629	0,891
GRINA	-0,075	0,733	0,931
ZNF280D	-0,075	0,651	0,899
SNX2	-0,076	0,686	0,913
C17orf80	-0,076	0,622	0,888
SEC22A	-0,076	0,723	0,928
ZFP3	-0,076	0,632	0,892
RNF13	-0,076	0,696	0,917
MRTFB	-0,076	0,631	0,892
EIF4ENIF1	-0,076	0,616	0,887
CBY1	-0,076	0,673	0,908
HECW2	-0,076	0,674	0,908
MRFAP1	-0,076	0,495	0,827
OXA1L	-0,076	0,536	0,850
PLBD2	-0,076	0,733	0,931
MTO1	-0,076	0,552	0,856
NDUFAF3	-0,076	0,661	0,903
MT-ATP6	-0,076	0,743	0,934
OPTN	-0,076	0,503	0,831

LETM1	-0,076	0,716	0,925
ERO1B	-0,076	0,652	0,899
MAP2K1	-0,076	0,565	0,862
NSFL1C	-0,076	0,540	0,851
MEF2C	-0,076	0,684	0,913
PDP1	-0,077	0,778	0,945
HEATR5B	-0,077	0,496	0,827
DIS3	-0,077	0,617	0,887
CAMSAP2	-0,077	0,656	0,900
TRIP4	-0,077	0,656	0,900
NSUN3	-0,077	0,668	0,907
LTA4H	-0,077	0,529	0,846
USO1	-0,077	0,619	0,888
CDC16	-0,077	0,475	0,818
RALB	-0,077	0,416	0,784
SHMT2	-0,077	0,692	0,916
PRCP	-0,078	0,579	0,870
PFN1	-0,078	0,663	0,904
ATP6V0D1	-0,078	0,656	0,900
IL17RD	-0,078	0,666	0,906
EDEM2	-0,078	0,727	0,930
FOXP1	-0,078	0,640	0,895
GPBP1	-0,078	0,588	0,875
BCLAF3	-0,078	0,600	0,882
CCM2	-0,078	0,636	0,893
AL592183.1	-0,078	0,759	0,939
ATP6V0B	-0,078	0,575	0,868
DUSP3	-0,078	0,704	0,921
RBM25	-0,078	0,540	0,852
CD99	-0,078	0,673	0,908
CKAP5	-0,078	0,558	0,858
CDK11B	-0,079	0,608	0,885
PCM1	-0,079	0,605	0,884
GPAT4	-0,079	0,524	0,843
TRMT13	-0,079	0,637	0,893
TFRC	-0,079	0,765	0,941
BCL2	-0,079	0,699	0,918
UFC1	-0,079	0,643	0,897
TM2D1	-0,079	0,584	0,872
DICER1	-0,079	0,526	0,844
RBAK	-0,079	0,608	0,885
EPC1	-0,079	0,444	0,798
APPL1	-0,080	0,641	0,896
TRIR	-0,080	0,557	0,858
ZNF721	-0,080	0,513	0,837
MBTPS2	-0,080	0,633	0,892
TVP23B	-0,080	0,716	0,925
CDC42EP3	-0,080	0,652	0,899
PAXIP1	-0,080	0,664	0,905
TMEM230	-0,080	0,498	0,828
NBEAL2	-0,080	0,727	0,930
SERPINH1	-0,080	0,760	0,939
RBM39	-0,080	0,603	0,883

YBX3	-0,080	0,659	0,902
ZDHHC6	-0,081	0,602	0,883
RHBDD1	-0,081	0,479	0,819
ZBTB18	-0,081	0,592	0,878
HDAC11	-0,081	0,685	0,913
MT-ND1	-0,081	0,778	0,945
R3HDM1	-0,081	0,564	0,862
NBAS	-0,081	0,550	0,856
MPLKIP	-0,081	0,621	0,888
ZNF350	-0,081	0,701	0,919
TTC38	-0,081	0,594	0,879
CHFR	-0,082	0,664	0,905
HTRA3	-0,082	0,790	0,948
NOL6	-0,082	0,673	0,908
GRN	-0,082	0,702	0,919
FSIP2	-0,082	0,753	0,937
PTTG1IP	-0,082	0,411	0,782
RABGAP1L	-0,082	0,759	0,939
TRUB2	-0,082	0,594	0,879
NSF	-0,082	0,522	0,842
KATNA1	-0,082	0,640	0,895
SZT2	-0,082	0,763	0,941
ZBED4	-0,083	0,625	0,889
ADCY9	-0,083	0,716	0,925
BTF3L4	-0,083	0,573	0,867
LAPTM4A	-0,083	0,560	0,859
GPRC5B	-0,083	0,724	0,928
PDCD11	-0,083	0,605	0,884
RPS24	-0,083	0,740	0,933
ZNF148	-0,083	0,647	0,899
FAM204A	-0,083	0,538	0,850
TRMT2B	-0,083	0,532	0,848
RUVBL2	-0,083	0,680	0,911
ALG9	-0,084	0,697	0,917
TBCD	-0,084	0,624	0,889
MAK16	-0,084	0,666	0,906
CFL1	-0,084	0,618	0,888
ATG9A	-0,084	0,688	0,914
DOCK4	-0,084	0,577	0,870
MLPH	-0,084	0,763	0,941
ZNF555	-0,084	0,631	0,892
METTL9	-0,084	0,565	0,862
USP24	-0,084	0,500	0,830
DDX5	-0,084	0,599	0,882
HMGN4	-0,084	0,518	0,839
WDR7	-0,084	0,414	0,783
LAMTOR1	-0,085	0,632	0,892
FURIN	-0,085	0,651	0,899
AR	-0,085	0,598	0,882
LCP2	-0,085	0,724	0,928
PXDC1	-0,085	0,699	0,918
RDH10	-0,085	0,849	0,968
MATR3	-0,085	0,702	0,919

FAM185A	-0,085	0,670	0,907
NEPRO	-0,085	0,477	0,819
FKBP1C	-0,085	0,615	0,887
GRHPR	-0,085	0,583	0,872
DCP1A	-0,085	0,399	0,774
KNTC1	-0,085	0,646	0,899
PLOD1	-0,086	0,744	0,934
HSPA1B	-0,086	0,807	0,953
TET1	-0,086	0,632	0,892
TBCEL	-0,086	0,588	0,875
NOM1	-0,086	0,578	0,870
HARS	-0,086	0,439	0,797
CUL2	-0,086	0,495	0,826
ZSCAN26	-0,086	0,653	0,899
PDLIM3	-0,086	0,744	0,934
DPP9	-0,086	0,672	0,908
HM13	-0,086	0,638	0,894
HIVEP2	-0,086	0,598	0,881
ZNF254	-0,086	0,625	0,889
PIGG	-0,087	0,597	0,881
DTWD2	-0,087	0,670	0,907
GPHN	-0,087	0,517	0,838
DDHD1	-0,087	0,511	0,836
TCEA3	-0,087	0,604	0,883
CNIH4	-0,087	0,540	0,851
STON2	-0,087	0,657	0,901
PCGF3	-0,087	0,483	0,821
PKN1	-0,087	0,697	0,917
SYNJ2BP	-0,087	0,512	0,837
FAM98A	-0,088	0,551	0,856
CEP192	-0,088	0,651	0,899
TRAK1	-0,088	0,709	0,923
BRD4	-0,088	0,747	0,936
RRP8	-0,088	0,478	0,819
STMP1	-0,088	0,527	0,844
HIP1R	-0,088	0,699	0,918
FANCL	-0,088	0,640	0,895
CDC27	-0,088	0,557	0,858
FAHD2A	-0,088	0,559	0,859
TTBK2	-0,088	0,480	0,819
PPP2R2D	-0,088	0,430	0,793
EMSY	-0,088	0,455	0,806
TRIM24	-0,088	0,479	0,819
ZNF24	-0,088	0,544	0,855
CNOT9	-0,088	0,439	0,797
PHYH	-0,088	0,583	0,872
CNOT7	-0,089	0,600	0,883
RIMKLB	-0,089	0,515	0,837
XBP1	-0,089	0,618	0,887
STRN3	-0,089	0,604	0,883
UPRT	-0,089	0,628	0,891
BABAM2	-0,089	0,519	0,840
PPHLN1	-0,089	0,497	0,827

FBXO3	-0,089	0,578	0,870
DNAJC16	-0,089	0,459	0,810
RUSC2	-0,089	0,675	0,908
GTPBP10	-0,089	0,580	0,871
EXOSC2	-0,089	0,578	0,870
ABCB4	-0,089	0,739	0,933
GK	-0,089	0,704	0,921
FASTK	-0,089	0,654	0,900
PRKAG2	-0,090	0,750	0,936
INPP5A	-0,090	0,609	0,885
RB1	-0,090	0,518	0,839
POLR1A	-0,090	0,689	0,914
ZNF518A	-0,090	0,622	0,889
CLPX	-0,090	0,449	0,802
RDX	-0,090	0,550	0,856
CD93	-0,090	0,576	0,870
SPCS2	-0,090	0,505	0,832
MGAT1	-0,090	0,614	0,887
SIL1	-0,090	0,595	0,880
TRIM8	-0,090	0,707	0,923
NDC1	-0,090	0,627	0,890
SNX16	-0,090	0,719	0,925
POMGNT1	-0,090	0,581	0,871
SGTA	-0,090	0,737	0,933
ARPC4	-0,090	0,603	0,883
CNIH1	-0,090	0,646	0,899
RINT1	-0,090	0,612	0,887
FAM126B	-0,090	0,581	0,871
KHDC4	-0,091	0,571	0,866
PLXNC1	-0,091	0,662	0,904
ZNF292	-0,091	0,563	0,861
SSB	-0,091	0,548	0,855
PNPLA4	-0,091	0,596	0,880
NELFCD	-0,091	0,552	0,856
DOCK9	-0,091	0,588	0,875
TIAL1	-0,091	0,448	0,802
FAM219B	-0,092	0,595	0,879
ALG5	-0,092	0,655	0,900
SNX14	-0,092	0,500	0,829
RNF130	-0,092	0,523	0,842
CDKN1C	-0,092	0,690	0,915
TRAPPC8	-0,092	0,554	0,858
NSMCE4A	-0,092	0,508	0,833
ZNF720	-0,092	0,463	0,811
PPP1R12A	-0,092	0,507	0,832
OSBPL9	-0,092	0,435	0,796
GET3	-0,092	0,501	0,830
TMEM185B	-0,092	0,522	0,842
EIF4G1	-0,092	0,667	0,906
GLCE	-0,092	0,577	0,870
LRPPRC	-0,092	0,557	0,858
TOPBP1	-0,093	0,511	0,836
SNX24	-0,093	0,659	0,902

OTUB1	-0,093	0,574	0,868
ERP44	-0,093	0,431	0,794
NOL3	-0,093	0,562	0,860
RNGTT	-0,093	0,539	0,851
LDHD	-0,093	0,746	0,935
TFAM	-0,093	0,602	0,883
CCDC84	-0,093	0,608	0,885
MGAT5	-0,093	0,686	0,913
WDR33	-0,094	0,352	0,744
SNX27	-0,094	0,573	0,867
PEX5	-0,094	0,579	0,870
GAB3	-0,094	0,731	0,931
AARS	-0,094	0,577	0,870
ARPC2	-0,094	0,441	0,797
WNT2B	-0,094	0,600	0,882
DMAP1	-0,094	0,588	0,875
CBLL1	-0,094	0,536	0,850
NFS1	-0,094	0,533	0,848
ARHGEF3	-0,094	0,646	0,899
DMAC1	-0,094	0,626	0,889
MTERF4	-0,094	0,516	0,838
DEGS1	-0,094	0,472	0,816
ENTPD6	-0,094	0,585	0,872
USP32	-0,094	0,480	0,819
HERC6	-0,094	0,570	0,865
LHFPL6	-0,095	0,508	0,833
IMP4	-0,095	0,466	0,812
PRPF40A	-0,095	0,526	0,844
UTP4	-0,095	0,529	0,846
CYP3A5	-0,095	0,646	0,899
CTNNA3	-0,095	0,788	0,948
LUC7L2	-0,095	0,558	0,858
ZFYVE16	-0,095	0,652	0,899
H3F3A	-0,095	0,470	0,815
ZNHIT3	-0,096	0,642	0,897
LAMA2	-0,096	0,636	0,893
CCNT2	-0,096	0,547	0,855
CNNM4	-0,096	0,711	0,923
PDK1	-0,096	0,622	0,889
MLEC	-0,096	0,568	0,864
DERA	-0,096	0,636	0,893
UNK	-0,096	0,547	0,855
ZNF43	-0,097	0,494	0,826
COL4A3	-0,097	0,741	0,933
ETF1	-0,097	0,431	0,794
PRKCD	-0,097	0,694	0,917
MRTFA	-0,097	0,725	0,929
ZDHHC2	-0,097	0,621	0,888
PIGF	-0,097	0,661	0,903
CDC40	-0,097	0,542	0,853
PIAS2	-0,097	0,452	0,805
LONRF1	-0,097	0,517	0,838
TANK	-0,097	0,562	0,861

PPP2R2A	-0,097	0,420	0,787
SRP54	-0,097	0,515	0,837
FUT8	-0,097	0,531	0,847
HNRNPH2	-0,097	0,469	0,815
SELENON	-0,097	0,672	0,908
ECD	-0,097	0,490	0,824
FLII	-0,097	0,587	0,874
CCDC59	-0,098	0,620	0,888
SLC8A1	-0,098	0,800	0,950
EIF2B5	-0,098	0,393	0,770
RBM19	-0,098	0,568	0,864
LEO1	-0,098	0,581	0,871
PRPS1	-0,098	0,531	0,847
IAH1	-0,098	0,411	0,782
EXOC8	-0,098	0,445	0,799
PSME2	-0,098	0,496	0,827
AP4S1	-0,098	0,501	0,830
COL6A2	-0,098	0,698	0,918
RIT1	-0,099	0,484	0,822
DCN	-0,099	0,702	0,919
CABIN1	-0,099	0,629	0,891
PSMD2	-0,099	0,469	0,815
SRF	-0,099	0,606	0,885
GALM	-0,099	0,517	0,838
GADD45B	-0,099	0,742	0,933
GSPT1	-0,099	0,296	0,706
SOAT1	-0,099	0,612	0,886
PCMTD1	-0,099	0,651	0,899
CEP44	-0,099	0,556	0,858
SPART	-0,099	0,521	0,842
INTS8	-0,099	0,467	0,813
ZCCHC17	-0,099	0,554	0,858
PMPCB	-0,099	0,353	0,744
VPS13D	-0,099	0,538	0,850
DDX20	-0,100	0,542	0,853
VPS13C	-0,100	0,545	0,855
DNAJA2	-0,100	0,437	0,797
ZCCHC4	-0,100	0,556	0,858
PANK4	-0,100	0,577	0,870
BLOC1S6	-0,100	0,544	0,855
CHORDC1	-0,100	0,629	0,891
COG1	-0,100	0,540	0,852
EDRF1	-0,100	0,470	0,815
FOPNL	-0,100	0,532	0,847
VTI1B	-0,100	0,430	0,794
XRCC5	-0,100	0,551	0,856
FMNL2	-0,100	0,675	0,908
NCKAP1	-0,100	0,489	0,824
ICA1	-0,100	0,617	0,887
NLN	-0,100	0,556	0,858
LSM4	-0,100	0,506	0,832
RNF181	-0,100	0,613	0,887
CAPNS1	-0,100	0,482	0,821

COQ7	-0,101	0,494	0,826
FAM126A	-0,101	0,611	0,886
RBM4B	-0,101	0,477	0,819
POLG	-0,101	0,621	0,888
RRP7A	-0,101	0,701	0,919
CDC42	-0,101	0,453	0,806
ACADSB	-0,101	0,619	0,888
LYSMD3	-0,101	0,684	0,913
ELP5	-0,101	0,507	0,832
CTNNBIP1	-0,102	0,607	0,885
PPIG	-0,102	0,618	0,887
ZMYM6	-0,102	0,550	0,856
SGCB	-0,102	0,475	0,818
SUOX	-0,102	0,538	0,850
ZDHHC7	-0,102	0,460	0,810
SRGAP1	-0,102	0,619	0,888
FDFT1	-0,102	0,415	0,784
R3HCC1	-0,103	0,523	0,842
RAPGEF4	-0,103	0,604	0,883
HOMER2	-0,103	0,577	0,870
KPNA5	-0,103	0,620	0,888
STX18	-0,103	0,504	0,832
UBLCP1	-0,103	0,609	0,885
UBE4A	-0,103	0,312	0,716
SMAP1	-0,103	0,577	0,870
ZC3H12C	-0,103	0,371	0,756
NTAN1	-0,103	0,604	0,884
PCTP	-0,103	0,538	0,850
ZNF605	-0,103	0,475	0,818
PYURF	-0,103	0,547	0,855
SLC20A2	-0,103	0,617	0,887
DDX46	-0,103	0,340	0,736
AGK	-0,103	0,490	0,824
WDR36	-0,104	0,566	0,863
C1QA	-0,104	0,741	0,933
CLOCK	-0,104	0,439	0,797
SGCE	-0,104	0,532	0,847
VPS4A	-0,104	0,570	0,865
GPR157	-0,104	0,647	0,899
TCP11L2	-0,104	0,661	0,903
LBR	-0,104	0,536	0,850
DNAJB11	-0,104	0,599	0,882
DNAJC3	-0,104	0,536	0,850
HEBP1	-0,104	0,530	0,846
PPIA	-0,104	0,525	0,843
MGME1	-0,104	0,582	0,872
ECM1	-0,104	0,684	0,913
ARPC5L	-0,104	0,463	0,812
PINK1	-0,104	0,611	0,886
RPL26	-0,104	0,735	0,932
SNX4	-0,104	0,635	0,893
PPP1R12B	-0,104	0,771	0,943
TSG101	-0,105	0,315	0,718

ATPAF1	-0,105	0,482	0,821
FAM133B	-0,105	0,537	0,850
ARHGAP42	-0,105	0,542	0,853
MARCH6	-0,105	0,439	0,797
SMIM13	-0,105	0,521	0,842
MITF	-0,106	0,646	0,899
EPS15L1	-0,106	0,655	0,900
IDH3G	-0,106	0,504	0,832
POLR3GL	-0,106	0,551	0,856
MIGA2	-0,106	0,584	0,872
MAGOHB	-0,106	0,630	0,892
LPCAT2	-0,106	0,625	0,889
NR2C2	-0,106	0,494	0,826
CCDC68	-0,106	0,558	0,858
ZNF207	-0,106	0,502	0,830
LGR4	-0,106	0,487	0,823
LYRM4	-0,106	0,538	0,850
NAPG	-0,106	0,567	0,863
NASP	-0,106	0,440	0,797
NR2C1	-0,106	0,487	0,823
MTMR2	-0,106	0,441	0,797
UBE2A	-0,106	0,406	0,777
DNHD1	-0,107	0,673	0,908
ZKSCAN2	-0,107	0,538	0,850
TMA16	-0,107	0,609	0,885
SNRNP48	-0,107	0,466	0,812
RUNX1	-0,107	0,778	0,945
ERG	-0,107	0,442	0,797
DUSP27	-0,107	0,728	0,930
GDI2	-0,107	0,546	0,855
MFSD1	-0,107	0,539	0,850
USP47	-0,108	0,575	0,868
DZIP3	-0,108	0,570	0,865
MMRN2	-0,108	0,486	0,822
GOSR2	-0,108	0,354	0,744
ZNF567	-0,108	0,624	0,889
IRAK4	-0,108	0,507	0,832
CLASP2	-0,108	0,522	0,842
TADA2B	-0,108	0,552	0,856
ALDH1L1	-0,108	0,813	0,954
TNFAIP1	-0,108	0,508	0,833
KCTD1	-0,108	0,560	0,860
ZNF649	-0,108	0,575	0,868
CHML	-0,109	0,579	0,870
UBE2K	-0,109	0,390	0,768
PHTF2	-0,109	0,435	0,795
ADAM9	-0,109	0,568	0,864
MTFMT	-0,109	0,520	0,841
PEX13	-0,109	0,385	0,764
ACTL6A	-0,109	0,538	0,850
COX18	-0,109	0,468	0,813
SLC9A7	-0,109	0,589	0,876
PIN1	-0,109	0,544	0,855

TMEM168	-0,109	0,483	0,821
HACL1	-0,109	0,580	0,870
CUL1	-0,109	0,233	0,653
QKI	-0,109	0,559	0,859
VTI1A	-0,109	0,376	0,759
SCFD1	-0,109	0,585	0,873
MBNL1	-0,109	0,485	0,822
ZMYM2	-0,110	0,463	0,811
CHTOP	-0,110	0,328	0,726
GTF2H3	-0,110	0,545	0,855
ZFAND5	-0,110	0,474	0,817
PHF20	-0,110	0,312	0,716
CMAS	-0,110	0,402	0,775
COG4	-0,110	0,399	0,774
GYS1	-0,110	0,646	0,899
ANXA6	-0,110	0,564	0,862
SCCPDH	-0,110	0,546	0,855
PTMS	-0,110	0,681	0,911
PSMB4	-0,110	0,454	0,806
COL4A2	-0,110	0,653	0,899
ZDHHC11	-0,111	0,697	0,918
GABARAPL1	-0,111	0,470	0,815
CASZ1	-0,111	0,694	0,917
SMIM19	-0,111	0,522	0,842
MAGT1	-0,111	0,510	0,835
CDC37L1	-0,112	0,600	0,882
RNF103	-0,112	0,464	0,812
GOLGA4	-0,112	0,615	0,887
FBXO34	-0,112	0,349	0,743
NRF1	-0,112	0,411	0,782
SHPRH	-0,113	0,413	0,782
YES1	-0,113	0,471	0,816
CDK17	-0,113	0,555	0,858
ZNF493	-0,113	0,554	0,858
ELOB	-0,113	0,672	0,908
N4BP2L2	-0,113	0,590	0,877
JADE1	-0,113	0,487	0,823
GATD3A	-0,113	0,533	0,848
C5orf15	-0,113	0,489	0,824
SPCS1	-0,113	0,455	0,806
FAHD2B	-0,113	0,487	0,823
TCAIM	-0,113	0,493	0,826
MIEF1	-0,113	0,399	0,774
TMEM258	-0,113	0,481	0,820
LRRC8C	-0,114	0,431	0,794
SSBP3	-0,114	0,547	0,855
C1orf21	-0,114	0,512	0,837
ETS1	-0,114	0,493	0,826
RNF34	-0,114	0,413	0,782
CNOT10	-0,114	0,493	0,826
RCHY1	-0,114	0,496	0,827
RBM14	-0,114	0,603	0,883
ABLIM3	-0,115	0,600	0,882

CSTF1	-0,115	0,455	0,806
CCDC149	-0,115	0,423	0,789
ZNF770	-0,115	0,511	0,836
DLG3	-0,115	0,377	0,759
TNIK	-0,115	0,665	0,905
SUCLG2	-0,115	0,545	0,855
TFPI	-0,115	0,636	0,893
TIA1	-0,116	0,505	0,832
DAB2	-0,116	0,654	0,900
SCP2	-0,116	0,513	0,837
CLN5	-0,116	0,440	0,797
NT5C3A	-0,116	0,513	0,837
TNFSF10	-0,116	0,657	0,901
WDR26	-0,116	0,391	0,768
ANKRD13A	-0,116	0,302	0,712
RASSF4	-0,117	0,551	0,856
PHAX	-0,117	0,443	0,798
PDIK1L	-0,117	0,595	0,879
ABHD17B	-0,117	0,537	0,850
NCOA3	-0,117	0,362	0,751
GJC1	-0,117	0,592	0,879
SRSF3	-0,117	0,436	0,796
BMP2K	-0,117	0,464	0,812
PSMF1	-0,117	0,280	0,691
AKAP8	-0,117	0,430	0,793
ARFIP2	-0,118	0,429	0,792
NBEA	-0,118	0,584	0,872
ACP2	-0,118	0,473	0,817
PPP1R9A	-0,118	0,639	0,894
SLC30A6	-0,118	0,389	0,767
NIBAN1	-0,118	0,651	0,899
NAA30	-0,118	0,430	0,793
ERI2	-0,119	0,551	0,856
DMAC2L	-0,119	0,516	0,838
PAIP1	-0,119	0,339	0,736
PDSS2	-0,119	0,412	0,782
ITFG1	-0,119	0,464	0,812
OST4	-0,119	0,526	0,844
ZNF146	-0,119	0,460	0,810
NAPA	-0,119	0,526	0,844
ZNF44	-0,119	0,380	0,761
DDX10	-0,119	0,373	0,756
ZNF253	-0,120	0,494	0,826
APP	-0,120	0,430	0,793
INTS4	-0,120	0,359	0,749
HSPA1A	-0,120	0,757	0,938
ARHGEF7	-0,120	0,416	0,784
SLC16A7	-0,120	0,771	0,943
ZFAND1	-0,120	0,523	0,842
DDX60L	-0,120	0,486	0,822
MTERF2	-0,120	0,429	0,792
TLR4	-0,120	0,586	0,874
YIPF6	-0,120	0,429	0,792

TMEM245	-0,120	0,409	0,781
RAP2C	-0,120	0,385	0,764
STRADA	-0,120	0,587	0,875
FANCM	-0,121	0,458	0,809
NPIPA7	-0,121	0,567	0,864
PJA2	-0,121	0,557	0,858
BTN2A1	-0,121	0,349	0,743
ITPK1	-0,121	0,588	0,875
DNAJB9	-0,121	0,602	0,883
THOC1	-0,121	0,340	0,736
ANKRD20A3	-0,121	0,685	0,913
GAS7	-0,121	0,609	0,885
EIF3I	-0,121	0,332	0,730
C2CD6	-0,122	0,654	0,899
TAPT1	-0,122	0,369	0,756
MKNK2	-0,122	0,549	0,856
MLLT3	-0,122	0,462	0,811
FAF1	-0,122	0,266	0,679
ACOT2	-0,122	0,443	0,798
RRM1	-0,122	0,504	0,832
RPL7L1	-0,122	0,233	0,654
RIOK2	-0,122	0,574	0,868
PHF20L1	-0,122	0,496	0,827
PA2G4	-0,122	0,276	0,689
SLAIN2	-0,123	0,328	0,727
SLC1A5	-0,123	0,728	0,930
E2F4	-0,123	0,423	0,789
ELOVL5	-0,123	0,633	0,893
CADM1	-0,123	0,575	0,868
SMARCAL1	-0,123	0,489	0,824
RANBP2	-0,123	0,478	0,819
UBE2V2	-0,123	0,489	0,824
RHOJ	-0,123	0,489	0,824
SRSF11	-0,123	0,412	0,782
IMP3	-0,123	0,518	0,839
EXOC5	-0,123	0,497	0,827
ATG3	-0,123	0,369	0,756
ALKBH5	-0,123	0,435	0,795
ATP6V1B2	-0,123	0,254	0,667
LRRC8A	-0,123	0,557	0,858
ZNHIT1	-0,123	0,472	0,816
MGST3	-0,124	0,473	0,817
ACYP2	-0,124	0,515	0,837
NDRG1	-0,124	0,555	0,858
MYOT	-0,124	0,806	0,953
GPD2	-0,124	0,439	0,797
AKAP9	-0,124	0,481	0,819
PSEN2	-0,124	0,456	0,807
NMNAT1	-0,124	0,417	0,784
RCN1	-0,124	0,532	0,848
VSIG10	-0,124	0,514	0,837
COX11	-0,124	0,427	0,792
NDUFV1	-0,125	0,599	0,882

**Protein coding**	MORF4L2	-0,125	0,575	0,868
	STX12	-0,125	0,394	0,771
	HSPD1	-0,125	0,418	0,784
	ARL1	-0,125	0,545	0,855
	CEP295	-0,125	0,417	0,784
	MESD	-0,125	0,275	0,687
	ANK2	-0,125	0,596	0,880
	CNST	-0,125	0,346	0,740
	MCPH1	-0,125	0,341	0,736
	TEX2	-0,125	0,316	0,720
	TRPC1	-0,126	0,465	0,812
	CNOT2	-0,126	0,465	0,812
	PRR13	-0,126	0,475	0,818
	YTHDC2	-0,126	0,360	0,750
	PGAM5	-0,126	0,442	0,797
	AKAP1	-0,126	0,555	0,858
	COIL	-0,126	0,515	0,837
	CUEDC1	-0,126	0,449	0,802
	C1orf52	-0,126	0,494	0,826
	ZBTB6	-0,126	0,594	0,879
	SCO1	-0,126	0,286	0,699
	ALG8	-0,126	0,454	0,806
	SPPL2A	-0,126	0,376	0,758
	RSU1	-0,126	0,335	0,732
	ZNF622	-0,127	0,507	0,833
	TMEM183A	-0,127	0,386	0,766
	GCA	-0,127	0,683	0,913
	ASXL3	-0,127	0,503	0,831
	PCID2	-0,127	0,246	0,661
	MTERF1	-0,127	0,527	0,844
	ATRAID	-0,127	0,392	0,769
	KRAS	-0,127	0,401	0,774
	SYDE2	-0,127	0,499	0,829
	NPIPA2	-0,127	0,582	0,872
	TP53RK	-0,127	0,509	0,833
	ABHD10	-0,127	0,521	0,841
	MFAP3	-0,127	0,439	0,797
	CRAT	-0,127	0,521	0,842
	GRAMD2B	-0,127	0,413	0,782
	ZNF595	-0,128	0,418	0,784
	STK39	-0,128	0,301	0,711
	YBX1	-0,128	0,321	0,722
	URM1	-0,128	0,421	0,788
	CSTB	-0,128	0,372	0,756
	AASDHPPT	-0,128	0,485	0,822
	CCND3	-0,128	0,463	0,811
	RPA3	-0,128	0,547	0,855
	ERH	-0,128	0,395	0,771
	LARS	-0,128	0,411	0,782
	NSUN4	-0,128	0,379	0,761
	GPC1	-0,129	0,525	0,843
	CLPB	-0,129	0,479	0,819
	MORF4L1	-0,129	0,380	0,761

TACC1	-0,129	0,502	0,830
CRYAB	-0,129	0,624	0,889
AFF1	-0,130	0,365	0,754
CD46	-0,130	0,473	0,816
EIF3J	-0,130	0,381	0,761
SPRYD4	-0,130	0,434	0,795
FBLN1	-0,130	0,672	0,908
POLK	-0,130	0,467	0,813
NDUFAF8	-0,130	0,459	0,809
ZMAT2	-0,130	0,473	0,816
MAP4K3	-0,130	0,443	0,798
KYAT3	-0,130	0,547	0,855
BCAP31	-0,130	0,502	0,830
ACP1	-0,130	0,404	0,776
TSPYL5	-0,130	0,450	0,803
RNF121	-0,131	0,438	0,797
PPP1R18	-0,131	0,530	0,846
LRIG1	-0,131	0,543	0,855
TTI1	-0,131	0,325	0,724
CEP57	-0,131	0,518	0,839
MAPKAP1	-0,131	0,191	0,616
BCS1L	-0,131	0,400	0,774
FBXL7	-0,131	0,473	0,816
TTC33	-0,131	0,545	0,855
ARFRP1	-0,131	0,491	0,824
PIAS1	-0,131	0,236	0,655
CYP4V2	-0,131	0,534	0,849
MAGEH1	-0,132	0,440	0,797
SF3B1	-0,132	0,434	0,795
TRMT11	-0,132	0,400	0,774
PELO	-0,132	0,366	0,755
COL4A1	-0,132	0,573	0,867
LCORL	-0,132	0,507	0,832
CREG1	-0,132	0,432	0,794
PTPN20	-0,132	0,662	0,904
TMX3	-0,132	0,480	0,819
BMS1	-0,132	0,246	0,661
GSTK1	-0,132	0,362	0,751
PPM1A	-0,132	0,439	0,797
TMEM69	-0,132	0,434	0,795
SUGP2	-0,132	0,437	0,796
DBT	-0,133	0,430	0,793
RRAGB	-0,133	0,436	0,796
RARS	-0,133	0,582	0,872
KIF1BP	-0,133	0,339	0,735
CTDSPL2	-0,133	0,417	0,784
NOL10	-0,133	0,343	0,738
OXR1	-0,133	0,542	0,853
HIST1H1E	-0,133	0,751	0,936
RNF8	-0,133	0,316	0,720
PIGO	-0,134	0,504	0,832
GPM6B	-0,134	0,553	0,856
PLEKHA1	-0,134	0,475	0,818

PTAR1	-0,134	0,546	0,855
ALG11	-0,134	0,457	0,808
HK1	-0,134	0,465	0,812
KIF1C	-0,134	0,534	0,849
EIF6	-0,134	0,546	0,855
STARD3NL	-0,134	0,418	0,784
TM2D3	-0,134	0,288	0,700
ANAPC5	-0,134	0,149	0,559
SKP1	-0,134	0,446	0,800
SELENOS	-0,135	0,278	0,690
TRNAU1AP	-0,135	0,479	0,819
CEBPD	-0,135	0,653	0,899
METTL2A	-0,135	0,403	0,775
HIBCH	-0,135	0,215	0,637
GNL2	-0,135	0,341	0,737
TNFAIP2	-0,135	0,527	0,844
CEP57L1	-0,135	0,432	0,794
DDX52	-0,135	0,282	0,694
AP3D1	-0,135	0,421	0,788
SFXN1	-0,135	0,320	0,721
TCEAL4	-0,135	0,345	0,739
ZBTB43	-0,135	0,394	0,771
DCLRE1A	-0,135	0,448	0,802
DPP7	-0,136	0,602	0,883
GBA2	-0,136	0,486	0,822
STX3	-0,136	0,380	0,761
TRNT1	-0,136	0,367	0,755
BTBD9	-0,136	0,409	0,781
MTPAP	-0,136	0,250	0,664
CHUK	-0,136	0,365	0,754
OTOGL	-0,136	0,783	0,946
SHE	-0,136	0,409	0,781
RFLNB	-0,136	0,402	0,774
ATP6V0A2	-0,136	0,293	0,703
THOC2	-0,136	0,347	0,741
EPRS	-0,136	0,395	0,771
DSC1	-0,136	0,713	0,924
ACOT9	-0,136	0,304	0,712
FSTL1	-0,136	0,406	0,777
DNAH14	-0,137	0,524	0,842
SIAH1	-0,137	0,505	0,832
GPI	-0,137	0,464	0,812
LPAR1	-0,137	0,607	0,885
CREBZF	-0,137	0,463	0,812
FITM2	-0,137	0,609	0,885
UTP14A	-0,137	0,479	0,819
EHHADH	-0,137	0,517	0,838
PMM2	-0,137	0,499	0,829
TIPRL	-0,137	0,502	0,830
COQ10B	-0,137	0,486	0,822
KIF27	-0,137	0,448	0,802
TOMM22	-0,137	0,213	0,634
NACC2	-0,137	0,365	0,754

STXBP3	-0,137	0,491	0,824
AIG1	-0,138	0,321	0,721
THEM4	-0,138	0,352	0,744
APEH	-0,138	0,456	0,807
BTBD3	-0,138	0,378	0,761
TXNDC11	-0,138	0,303	0,712
UBE2G2	-0,138	0,225	0,646
ZNF439	-0,138	0,485	0,822
LRRC28	-0,138	0,357	0,747
PGGT1B	-0,138	0,435	0,796
TFB2M	-0,138	0,495	0,827
CHMP7	-0,138	0,298	0,709
AUH	-0,138	0,320	0,721
ZFX	-0,139	0,405	0,776
SAP18	-0,139	0,363	0,752
CACNA1C	-0,139	0,653	0,899
ZFAND6	-0,139	0,391	0,769
PDE4DIP	-0,139	0,616	0,887
ZNF772	-0,139	0,324	0,724
PCDHGA6	-0,139	0,624	0,889
ARHGAP27	-0,139	0,504	0,832
NUDCD1	-0,139	0,550	0,856
U2SURP	-0,139	0,367	0,755
STAM2	-0,139	0,428	0,792
LZIC	-0,139	0,274	0,687
ZKSCAN5	-0,139	0,400	0,774
YWHAE	-0,139	0,192	0,616
HINT3	-0,139	0,372	0,756
SLC35A4	-0,140	0,427	0,792
SMYD4	-0,140	0,393	0,770
EXOC1	-0,140	0,266	0,679
MIOS	-0,140	0,309	0,715
C2orf69	-0,140	0,416	0,784
AGPAT3	-0,140	0,428	0,792
CTSZ	-0,140	0,304	0,712
SREK1	-0,140	0,368	0,756
TPP2	-0,140	0,299	0,710
DENND2A	-0,140	0,630	0,892
PAPOLA	-0,140	0,293	0,703
RAB1A	-0,140	0,224	0,646
GCSH	-0,140	0,461	0,810
CRBN	-0,141	0,523	0,842
LANCL1	-0,141	0,455	0,807
PKIG	-0,141	0,439	0,797
ZBTB26	-0,141	0,497	0,827
LAPTM5	-0,141	0,614	0,887
MARK1	-0,141	0,334	0,731
WSB2	-0,141	0,279	0,690
USP53	-0,141	0,471	0,816
FAR1	-0,141	0,473	0,816
OSBPL3	-0,141	0,606	0,885
FNTA	-0,141	0,284	0,696
IRAK1	-0,141	0,483	0,821

QARS	-0,141	0,278	0,690
LPCAT1	-0,141	0,417	0,784
TRIM13	-0,141	0,297	0,708
ZNF680	-0,141	0,417	0,784
TMEM117	-0,141	0,445	0,799
IRS2	-0,142	0,545	0,855
TMEM65	-0,142	0,493	0,826
SSR3	-0,142	0,459	0,809
ODR4	-0,142	0,432	0,794
PPP4R2	-0,142	0,480	0,819
INSIG1	-0,142	0,574	0,868
ZFP28	-0,142	0,402	0,774
GANC	-0,142	0,241	0,659
N6AMT1	-0,142	0,366	0,755
SENP7	-0,142	0,404	0,776
NCOA1	-0,143	0,272	0,685
ICAM1	-0,143	0,615	0,887
PPP1R2	-0,143	0,445	0,799
PTGFRN	-0,143	0,429	0,793
LAMP2	-0,143	0,344	0,739
RBM45	-0,143	0,464	0,812
ERBB2	-0,143	0,569	0,865
ITGB1	-0,143	0,479	0,819
TCOF1	-0,143	0,493	0,826
FBXO22	-0,143	0,298	0,709
MOV10	-0,143	0,474	0,817
ADARB1	-0,144	0,333	0,730
RHOQ	-0,144	0,378	0,761
ZFP91	-0,144	0,182	0,604
EBNA1BP2	-0,144	0,330	0,729
DCUN1D1	-0,144	0,542	0,853
HYAL2	-0,144	0,548	0,855
H2AFV	-0,144	0,189	0,614
SEC31B	-0,144	0,495	0,826
NUP88	-0,145	0,276	0,689
RMND5B	-0,145	0,397	0,773
ATG5	-0,145	0,353	0,744
PCCA	-0,145	0,242	0,659
TUBA1B	-0,145	0,495	0,826
STAMBP	-0,145	0,300	0,710
RHOC	-0,145	0,436	0,796
RNF220	-0,145	0,325	0,724
UBL7	-0,145	0,368	0,756
CWC27	-0,145	0,330	0,729
CAV2	-0,145	0,478	0,819
NUP107	-0,145	0,368	0,756
REV3L	-0,145	0,211	0,632
PLAA	-0,146	0,255	0,668
FBXO42	-0,146	0,282	0,693
ASAH1	-0,146	0,592	0,879
LMOD2	-0,146	0,649	0,899
CMBL	-0,146	0,392	0,769
TSPYL4	-0,146	0,427	0,792

ZBTB7B	-0,146	0,447	0,802
DENND4B	-0,146	0,538	0,850
BBIP1	-0,147	0,303	0,712
CPSF3	-0,147	0,375	0,757
PPP3CC	-0,147	0,193	0,617
ANKRD20A1	-0,147	0,558	0,858
EPDR1	-0,147	0,375	0,757
TCIM	-0,147	0,515	0,837
MANEA	-0,147	0,524	0,843
AGAP1	-0,148	0,519	0,839
TARS	-0,148	0,378	0,761
PPP6R2	-0,148	0,350	0,744
RPAIN	-0,148	0,301	0,711
HOOK1	-0,148	0,602	0,883
SLC4A3	-0,148	0,558	0,858
CYB5R4	-0,148	0,320	0,721
TMEM203	-0,148	0,303	0,712
BIVM	-0,148	0,316	0,719
WDR3	-0,148	0,272	0,685
ADGRD1	-0,148	0,548	0,856
NEDD1	-0,148	0,468	0,813
CSNK1A1	-0,148	0,225	0,646
CEBPZ	-0,148	0,548	0,856
PRKRA	-0,148	0,310	0,715
ZRANB2	-0,149	0,388	0,767
EIF4E3	-0,149	0,239	0,657
ROMO1	-0,149	0,560	0,859
C5orf22	-0,149	0,336	0,732
SLC25A13	-0,149	0,317	0,720
CD63	-0,149	0,299	0,710
DDIT3	-0,150	0,449	0,802
ARPC3	-0,150	0,403	0,775
PEX2	-0,150	0,408	0,780
WDSUB1	-0,150	0,436	0,796
NUP58	-0,150	0,395	0,771
TIMM22	-0,150	0,323	0,724
ZNF431	-0,150	0,367	0,755
UBE2D2	-0,151	0,223	0,645
HMGB1	-0,151	0,482	0,821
THAP11	-0,151	0,359	0,749
TRIM23	-0,151	0,487	0,823
PPARGC1B	-0,151	0,537	0,850
PPP2R5A	-0,151	0,214	0,634
ITM2A	-0,151	0,502	0,830
SETMAR	-0,151	0,364	0,753
DPH5	-0,151	0,360	0,750
HDHD5	-0,151	0,351	0,744
TMEM106C	-0,152	0,254	0,666
LRRC32	-0,152	0,560	0,860
RTF2	-0,152	0,197	0,621
PAM	-0,152	0,575	0,868
FILIP1	-0,152	0,632	0,892
TUBGCP4	-0,152	0,405	0,776

STX4	-0,152	0,425	0,791
ELP4	-0,152	0,405	0,777
PLRG1	-0,152	0,464	0,812
KLHL28	-0,152	0,449	0,802
USP31	-0,152	0,325	0,725
ZNF664	-0,152	0,155	0,567
SRSF7	-0,152	0,331	0,730
MAP3K4	-0,153	0,201	0,624
ABCE1	-0,153	0,489	0,824
ZNF639	-0,153	0,242	0,659
GSKIP	-0,153	0,506	0,832
COA7	-0,153	0,384	0,764
SCYL3	-0,153	0,352	0,744
MT-CYB	-0,153	0,601	0,883
XPNPEP1	-0,153	0,211	0,632
RDH11	-0,153	0,305	0,713
ALDH1B1	-0,153	0,449	0,802
EPG5	-0,153	0,365	0,754
FKBP15	-0,153	0,125	0,527
ZNF780B	-0,153	0,441	0,797
PRKACA	-0,153	0,551	0,856
RB1CC1	-0,154	0,418	0,784
TMEM63A	-0,154	0,537	0,850
PAICS	-0,154	0,186	0,609
SHOC2	-0,154	0,360	0,750
ANKRD27	-0,154	0,195	0,618
GPN1	-0,154	0,340	0,736
SNAPC3	-0,154	0,229	0,649
MYL6	-0,154	0,488	0,824
CXorf40A	-0,154	0,359	0,748
FSD2	-0,154	0,643	0,897
DMPK	-0,154	0,492	0,826
ARL4A	-0,154	0,494	0,826
IFI27	-0,155	0,410	0,782
FAN1	-0,155	0,307	0,714
MED4	-0,155	0,318	0,721
SESTD1	-0,155	0,271	0,684
ZNF41	-0,155	0,358	0,748
DKC1	-0,155	0,237	0,656
EPM2A	-0,155	0,356	0,746
COL6A3	-0,155	0,581	0,872
COX7A2L	-0,155	0,198	0,622
TTC19	-0,155	0,173	0,592
RSL24D1	-0,155	0,574	0,868
PREX2	-0,155	0,349	0,743
HERC2	-0,155	0,498	0,828
GMFB	-0,155	0,592	0,878
GUF1	-0,155	0,261	0,675
JKAMP	-0,155	0,439	0,797
DNAJA3	-0,155	0,358	0,747
CXXC5	-0,156	0,253	0,666
DHRS4	-0,156	0,387	0,766
RNF150	-0,156	0,434	0,795

RBPMS	-0,156	0,405	0,776
ACVRL1	-0,156	0,438	0,797
CCNL1	-0,156	0,535	0,850
SNX6	-0,156	0,452	0,805
PUM3	-0,156	0,337	0,732
DEDD	-0,156	0,330	0,729
COPZ1	-0,156	0,221	0,643
PTCD2	-0,157	0,320	0,721
MIPOL1	-0,157	0,425	0,791
UBE2B	-0,157	0,377	0,760
TTC21B	-0,157	0,272	0,685
ISCU	-0,157	0,423	0,789
FRYL	-0,157	0,288	0,700
DOCK5	-0,157	0,301	0,711
PARP6	-0,157	0,304	0,712
UFSP2	-0,157	0,372	0,756
MTUS1	-0,157	0,341	0,736
EMP2	-0,157	0,385	0,765
RND3	-0,157	0,549	0,856
KLHL18	-0,158	0,298	0,709
MRPS14	-0,158	0,286	0,699
S100A11	-0,158	0,603	0,883
PEBP1	-0,158	0,239	0,657
ACBD6	-0,158	0,278	0,690
EID1	-0,158	0,370	0,756
ERCC1	-0,158	0,431	0,794
SLC9A8	-0,158	0,355	0,746
PYROXD1	-0,158	0,360	0,750
KPNA6	-0,158	0,277	0,690
NPIPA1	-0,158	0,537	0,850
RAD23A	-0,158	0,352	0,744
NRDE2	-0,158	0,336	0,732
HDAC2	-0,158	0,143	0,551
NOP58	-0,158	0,276	0,689
EIF3M	-0,159	0,373	0,756
NECTIN2	-0,159	0,483	0,821
THAP9	-0,159	0,514	0,837
ITGA7	-0,159	0,535	0,850
MARCH5	-0,159	0,243	0,660
RFTN1	-0,159	0,427	0,792
PPP6R3	-0,159	0,194	0,618
VPS37A	-0,159	0,334	0,731
RSRC2	-0,160	0,219	0,641
PTPN2	-0,160	0,353	0,744
PRMT1	-0,160	0,395	0,771
NAV3	-0,160	0,583	0,872
FAM122A	-0,160	0,341	0,736
ACVR1B	-0,160	0,437	0,796
USP45	-0,160	0,352	0,744
COX6A2	-0,160	0,587	0,874
MRPS30	-0,160	0,305	0,712
PSMD12	-0,160	0,254	0,666
PCBD1	-0,160	0,312	0,716

DYNC1I2	-0,160	0,277	0,690
BRI3	-0,161	0,334	0,731
PEX1	-0,161	0,357	0,747
TMCO1	-0,161	0,392	0,769
PRCC	-0,161	0,400	0,774
DIO2	-0,161	0,619	0,888
DLG2	-0,161	0,324	0,724
ILVBL	-0,162	0,414	0,784
DYNC1LI1	-0,162	0,189	0,614
TLCD4	-0,162	0,472	0,816
RBMS1	-0,162	0,290	0,701
AREL1	-0,162	0,267	0,680
NUP42	-0,162	0,384	0,764
DNAJC2	-0,163	0,263	0,677
RNF14	-0,163	0,325	0,725
ST5	-0,163	0,514	0,837
AIMP1	-0,163	0,354	0,745
FKBP1A	-0,163	0,205	0,625
PROX1	-0,163	0,610	0,886
TRIM37	-0,163	0,159	0,572
UXT	-0,163	0,312	0,717
GTF2H5	-0,163	0,390	0,768
AKAP8L	-0,163	0,343	0,738
CDKN2AIPNL	-0,163	0,327	0,726
LCLAT1	-0,163	0,406	0,777
ZC3H15	-0,163	0,449	0,802
TBRG4	-0,164	0,409	0,781
PIGN	-0,164	0,380	0,761
TNFRSF1A	-0,164	0,527	0,844
CCDC82	-0,164	0,274	0,687
ARAF	-0,164	0,328	0,727
MED8	-0,164	0,349	0,743
ZBTB16	-0,164	0,728	0,930
CTDSPL	-0,165	0,121	0,521
COG2	-0,165	0,219	0,642
BECN1	-0,165	0,181	0,602
CFLAR	-0,165	0,370	0,756
MRPL9	-0,165	0,401	0,774
GNA12	-0,165	0,454	0,806
HMGCS1	-0,166	0,308	0,714
JAM2	-0,166	0,308	0,714
NSL1	-0,166	0,190	0,616
ST7L	-0,166	0,277	0,690
LNX1	-0,166	0,354	0,744
CFAP20	-0,166	0,301	0,711
PLEKHF2	-0,166	0,310	0,715
MT-CO1	-0,166	0,545	0,855
TALDO1	-0,166	0,372	0,756
MTRR	-0,166	0,179	0,600
STK33	-0,166	0,548	0,856
BTBD6	-0,166	0,417	0,784
NOP56	-0,166	0,229	0,649
IGSF9B	-0,166	0,618	0,887

ZNF141	-0,167	0,327	0,726
SGCG	-0,167	0,593	0,879
MRPL44	-0,167	0,202	0,624
PRKAA1	-0,167	0,252	0,666
HIST1H1C	-0,167	0,614	0,887
UBE2J1	-0,168	0,335	0,732
ATR	-0,168	0,228	0,648
MARK2	-0,168	0,450	0,803
ACAA2	-0,168	0,310	0,715
GNS	-0,168	0,165	0,580
DDX3X	-0,168	0,365	0,754
KCTD10	-0,168	0,304	0,712
GIMAP4	-0,168	0,424	0,789
ZCCHC2	-0,168	0,330	0,729
DNAJC8	-0,168	0,296	0,707
SFSWAP	-0,169	0,380	0,761
FAM49B	-0,169	0,358	0,747
TRAPPC3	-0,169	0,111	0,506
FLOT2	-0,169	0,362	0,751
NUDT2	-0,169	0,476	0,818
FBXL5	-0,169	0,370	0,756
CETN3	-0,169	0,431	0,794
CTBP2	-0,169	0,193	0,617
TBCA	-0,169	0,368	0,756
KLHDC2	-0,169	0,161	0,577
APBB1	-0,169	0,371	0,756
USP8	-0,170	0,191	0,616
TLE3	-0,170	0,485	0,822
COL6A1	-0,170	0,389	0,767
IDI1	-0,170	0,506	0,832
UGGT2	-0,170	0,325	0,724
RABL6	-0,170	0,415	0,784
PXN	-0,170	0,490	0,824
DARS	-0,170	0,341	0,736
MOSPD1	-0,170	0,502	0,830
AUP1	-0,170	0,321	0,722
RIC3	-0,170	0,345	0,740
RRAD	-0,170	0,661	0,903
APOOL	-0,171	0,328	0,726
NOD1	-0,171	0,354	0,745
MAP2K2	-0,171	0,399	0,774
CHRAC1	-0,171	0,366	0,755
MBTPS1	-0,171	0,283	0,695
DBI	-0,171	0,416	0,784
IGF2R	-0,171	0,432	0,794
DES	-0,171	0,554	0,858
LIFR	-0,171	0,401	0,774
ADD3	-0,171	0,306	0,713
BLOC1S1	-0,172	0,372	0,756
NR3C2	-0,172	0,205	0,625
TRIP10	-0,172	0,305	0,712
CRYZL1	-0,172	0,232	0,653
POLR2J	-0,172	0,226	0,646

CEP70	-0,172	0,380	0,761
SCAMP3	-0,172	0,296	0,706
FAM135A	-0,172	0,319	0,721
FAM13B	-0,172	0,280	0,692
MYSM1	-0,172	0,225	0,646
UHRF1BP1	-0,172	0,244	0,661
RTCA	-0,172	0,181	0,603
LUZP1	-0,173	0,180	0,600
MARCH7	-0,173	0,428	0,792
MECR	-0,173	0,310	0,715
PSMG1	-0,173	0,375	0,758
JARID2	-0,173	0,312	0,716
SLC25A33	-0,173	0,455	0,806
CD109	-0,174	0,427	0,792
SNRPD2	-0,174	0,398	0,774
INTS9	-0,174	0,311	0,716
DNAJC18	-0,174	0,241	0,659
ZW10	-0,174	0,281	0,692
PRPF39	-0,174	0,293	0,704
NRN1	-0,174	0,339	0,735
TDRD3	-0,174	0,372	0,756
IBTK	-0,174	0,334	0,731
MTMR10	-0,174	0,238	0,656
NUDT9	-0,174	0,202	0,624
INKA2	-0,174	0,471	0,816
ETFA	-0,174	0,382	0,763
ABCD2	-0,174	0,540	0,852
ZDHHC4	-0,175	0,262	0,676
STK38	-0,175	0,289	0,701
KCNH2	-0,175	0,435	0,795
ACTR8	-0,175	0,310	0,715
MARS	-0,175	0,241	0,659
NR1H3	-0,175	0,471	0,816
MRRF	-0,175	0,115	0,513
GIMAP6	-0,175	0,266	0,679
SLF1	-0,175	0,349	0,743
TMEM9B	-0,175	0,204	0,625
HADH	-0,175	0,268	0,681
ECHDC1	-0,175	0,504	0,832
MRPL30	-0,175	0,260	0,674
BCL2L13	-0,175	0,199	0,623
RHOT1	-0,176	0,271	0,684
XIRP1	-0,176	0,712	0,923
PFKM	-0,176	0,415	0,784
KBTBD3	-0,176	0,470	0,815
ACAA1	-0,176	0,250	0,664
MLF2	-0,176	0,217	0,638
CDK18	-0,176	0,500	0,829
PLP2	-0,177	0,310	0,715
ARL14EP	-0,177	0,392	0,769
SNAPIN	-0,177	0,189	0,613
ATP11B	-0,177	0,295	0,706
CDKAL1	-0,177	0,183	0,605

NUP85	-0,177	0,210	0,631
CHMP4B	-0,178	0,248	0,662
STRBP	-0,178	0,176	0,596
GLMN	-0,178	0,380	0,761
ZNF484	-0,178	0,241	0,659
LRRC2	-0,178	0,712	0,923
CTDNEP1	-0,178	0,376	0,758
EXOSC9	-0,178	0,284	0,696
CNBP	-0,178	0,248	0,661
ZNF112	-0,178	0,379	0,761
AP3S1	-0,178	0,322	0,723
NLK	-0,179	0,147	0,556
FRMD5	-0,179	0,531	0,847
RBM3	-0,179	0,271	0,684
SHROOM4	-0,179	0,400	0,774
NUP43	-0,179	0,144	0,554
ZNF708	-0,179	0,200	0,623
GART	-0,179	0,093	0,483
LRRFIP2	-0,179	0,388	0,767
RSAD1	-0,179	0,192	0,616
HSPB8	-0,179	0,338	0,734
ZNF248	-0,179	0,208	0,629
TMEM165	-0,179	0,336	0,732
CZIB	-0,179	0,154	0,566
MCCC1	-0,179	0,237	0,656
SLC25A40	-0,179	0,341	0,736
MBNL2	-0,180	0,280	0,692
PFDN1	-0,180	0,195	0,618
CAB39	-0,180	0,200	0,623
MAF1	-0,180	0,299	0,709
C17orf75	-0,180	0,284	0,696
EPB41L4B	-0,180	0,514	0,837
PSPC1	-0,180	0,083	0,468
ZSWIM6	-0,180	0,338	0,735
MRPL52	-0,180	0,315	0,719
USP10	-0,180	0,129	0,534
DDX39B	-0,180	0,400	0,774
NMD3	-0,180	0,414	0,783
HSPA14	-0,180	0,350	0,743
PTPRK	-0,181	0,142	0,551
RWDD4	-0,181	0,324	0,724
SMIM8	-0,181	0,401	0,774
NDRG2	-0,181	0,220	0,643
AP1AR	-0,181	0,310	0,715
RAB18	-0,181	0,401	0,774
TTC23	-0,181	0,183	0,605
VPS25	-0,181	0,254	0,667
CBX3	-0,181	0,324	0,724
GSE1	-0,181	0,448	0,802
LRRTM2	-0,182	0,474	0,817
FOXN3	-0,182	0,292	0,703
WDR77	-0,182	0,216	0,638
RMND5A	-0,182	0,297	0,707

MRPL45	-0,182	0,198	0,622
TUBA1A	-0,182	0,311	0,716
RASSF2	-0,182	0,541	0,853
ZNF33B	-0,182	0,289	0,701
ANAPC13	-0,182	0,423	0,789
ARMC1	-0,183	0,242	0,659
NAXE	-0,183	0,167	0,585
ZNF302	-0,183	0,302	0,711
NME6	-0,183	0,274	0,687
FGD4	-0,183	0,235	0,655
TRMT6	-0,183	0,304	0,712
ELAC2	-0,183	0,222	0,644
CIPC	-0,183	0,318	0,721
RNPEP	-0,184	0,380	0,761
SLC31A1	-0,184	0,195	0,618
CHMP2A	-0,184	0,320	0,721
LRRC42	-0,184	0,281	0,692
BRCA1	-0,184	0,354	0,744
CDC14A	-0,184	0,351	0,744
GNPTAB	-0,184	0,303	0,712
FAM136A	-0,185	0,278	0,690
TMSB10	-0,185	0,447	0,800
PFKFB2	-0,185	0,613	0,887
AP2A2	-0,185	0,339	0,735
KRCC1	-0,185	0,363	0,752
CFDP1	-0,185	0,231	0,651
MTCH1	-0,185	0,240	0,658
JAGN1	-0,185	0,270	0,684
PCDHGB6	-0,185	0,435	0,796
TXN	-0,185	0,375	0,758
SDHC	-0,185	0,151	0,562
MKLN1	-0,185	0,195	0,619
PIGX	-0,185	0,314	0,718
CASP8AP2	-0,185	0,275	0,687
ARHGAP31	-0,185	0,392	0,769
CTNNBL1	-0,185	0,130	0,535
PPM1G	-0,185	0,199	0,622
GASK1B	-0,185	0,204	0,625
METTL17	-0,185	0,203	0,625
GJA1	-0,186	0,518	0,839
CECR2	-0,186	0,499	0,829
PPID	-0,186	0,218	0,638
TAF12	-0,186	0,319	0,721
STRIP2	-0,186	0,490	0,824
DNAJC7	-0,186	0,051	0,388
NKIRAS2	-0,186	0,282	0,693
MYNN	-0,186	0,371	0,756
KLHL21	-0,186	0,450	0,803
UBL4A	-0,186	0,262	0,676
TDG	-0,186	0,200	0,623
PSMB2	-0,186	0,096	0,484
PGM5	-0,186	0,390	0,768
SMARCB1	-0,187	0,333	0,730

FAM192A	-0,187	0,071	0,446
PCNA	-0,187	0,249	0,662
ATP6V0E1	-0,187	0,197	0,621
ANKRD9	-0,187	0,278	0,690
MCCC2	-0,187	0,217	0,638
ANXA11	-0,188	0,357	0,747
MKNK1	-0,188	0,195	0,618
VPS13A	-0,188	0,186	0,610
SNRNP27	-0,188	0,238	0,656
PSME4	-0,188	0,332	0,730
RPN2	-0,188	0,144	0,554
CNDP2	-0,188	0,256	0,669
WDFY2	-0,188	0,271	0,684
ZNF615	-0,188	0,304	0,712
RNPC3	-0,188	0,234	0,655
TMEM161B	-0,188	0,313	0,717
COX10	-0,188	0,335	0,732
BCL7B	-0,188	0,237	0,656
PIK3C2A	-0,189	0,176	0,596
CTPS1	-0,189	0,427	0,792
RILPL1	-0,189	0,331	0,729
ACADVL	-0,189	0,373	0,756
SRSF4	-0,189	0,209	0,630
FBXL17	-0,189	0,269	0,682
UBE2D3	-0,189	0,267	0,679
CDK9	-0,189	0,333	0,731
UBAP1	-0,189	0,144	0,553
C19orf53	-0,189	0,242	0,659
MSL3	-0,190	0,236	0,655
YIPF1	-0,190	0,262	0,676
TOMM70	-0,190	0,169	0,587
RP9	-0,190	0,307	0,714
PSMB1	-0,190	0,252	0,666
BAG1	-0,190	0,095	0,484
PRRG1	-0,190	0,242	0,659
TWISTNB	-0,190	0,391	0,768
SPATA13	-0,191	0,217	0,638
EDF1	-0,191	0,403	0,776
SAR1B	-0,191	0,162	0,578
RAD17	-0,191	0,258	0,672
IFITM1	-0,191	0,237	0,656
CD276	-0,191	0,399	0,774
PEX3	-0,191	0,285	0,698
HSPA13	-0,191	0,522	0,842
ISCA1	-0,191	0,210	0,631
TMED7	-0,192	0,480	0,819
WDR60	-0,192	0,211	0,632
CEP152	-0,192	0,353	0,744
RER1	-0,192	0,052	0,388
GPR183	-0,192	0,523	0,842
IGFBP5	-0,192	0,606	0,885
ANGPT2	-0,192	0,424	0,790
ASAP3	-0,192	0,257	0,670

EBAG9	-0,192	0,243	0,660
CAMK1D	-0,193	0,432	0,794
MAOA	-0,193	0,493	0,826
YAF2	-0,193	0,322	0,723
PHYKPL	-0,193	0,317	0,720
FCGR3A	-0,193	0,547	0,855
ACTB	-0,193	0,514	0,837
DPY30	-0,193	0,300	0,710
LARP4	-0,193	0,321	0,723
HSP90B1	-0,193	0,416	0,784
EIF5A	-0,193	0,222	0,645
NIT1	-0,194	0,226	0,646
POU5F2	-0,194	0,305	0,713
NT5DC1	-0,194	0,241	0,659
OSGEPL1	-0,194	0,248	0,661
CLK4	-0,194	0,289	0,701
RPL36AL	-0,194	0,471	0,816
SPRYD7	-0,194	0,265	0,678
GKAP1	-0,194	0,256	0,670
FXR2	-0,194	0,261	0,675
THAP1	-0,195	0,359	0,749
PPIE	-0,195	0,169	0,587
TM2D2	-0,195	0,192	0,616
NUP205	-0,195	0,094	0,484
POPDC2	-0,195	0,385	0,765
UBE2E1	-0,195	0,332	0,730
AGPAT4	-0,195	0,365	0,754
TNNT2	-0,195	0,556	0,858
VEZT	-0,195	0,357	0,747
PTPA	-0,195	0,296	0,706
TSHZ1	-0,196	0,263	0,676
PSME3	-0,196	0,107	0,501
NDUFB7	-0,196	0,460	0,810
UROS	-0,196	0,169	0,587
PLEKHB2	-0,196	0,057	0,407
TRDMT1	-0,196	0,270	0,683
PLIN5	-0,196	0,465	0,812
SH3BP2	-0,196	0,372	0,756
AFG1L	-0,197	0,339	0,735
SAP30BP	-0,197	0,108	0,502
ELL2	-0,197	0,415	0,784
CDC42BPA	-0,197	0,147	0,556
VPS36	-0,197	0,143	0,551
CCDC141	-0,197	0,703	0,921
MAPKAPK2	-0,197	0,315	0,719
TUFM	-0,197	0,288	0,700
TATDN3	-0,198	0,368	0,756
TPM4	-0,198	0,336	0,732
KIAA0232	-0,198	0,225	0,646
MNAT1	-0,198	0,247	0,661
FXR1	-0,198	0,248	0,661
SMPD1	-0,198	0,318	0,721
RFK	-0,198	0,259	0,673

UFD1	-0,198	0,140	0,547
DNM3	-0,198	0,258	0,671
MRPL42	-0,198	0,231	0,652
CHSY1	-0,198	0,292	0,703
SIPA1L2	-0,198	0,356	0,746
TRAPPC12	-0,199	0,203	0,625
MMAB	-0,199	0,211	0,632
ZFAND4	-0,199	0,220	0,643
DPM1	-0,199	0,429	0,792
WASF3	-0,199	0,308	0,715
HSPA9	-0,199	0,262	0,676
METTL25	-0,199	0,322	0,723
BLCAP	-0,199	0,166	0,583
UBA5	-0,199	0,179	0,600
LRCH2	-0,200	0,340	0,736
MAPRE1	-0,200	0,204	0,625
SCRN3	-0,200	0,297	0,707
CYTH1	-0,200	0,153	0,565
DYNLL2	-0,200	0,239	0,657
ACOT1	-0,200	0,262	0,676
SMIM11A	-0,200	0,177	0,596
C6orf89	-0,201	0,113	0,511
KLHL38	-0,201	0,611	0,886
SMDT1	-0,201	0,247	0,661
CGRRF1	-0,201	0,275	0,688
EXOC6	-0,201	0,307	0,714
FAM98B	-0,201	0,171	0,588
BNIP3	-0,201	0,300	0,710
MT-CO3	-0,201	0,389	0,767
VEGFA	-0,201	0,436	0,796
OAZ1	-0,201	0,299	0,710
SLC25A24	-0,201	0,231	0,651
CDC26	-0,201	0,320	0,721
EMC4	-0,201	0,160	0,575
GAREM1	-0,201	0,325	0,724
AKIRIN1	-0,201	0,165	0,582
GUCY1A2	-0,201	0,424	0,791
C1QC	-0,202	0,493	0,826
GUCD1	-0,202	0,214	0,634
TLK2	-0,202	0,049	0,381
LIG4	-0,202	0,287	0,699
LIN7A	-0,202	0,355	0,746
BVES	-0,202	0,453	0,806
PLXNA1	-0,202	0,400	0,774
FRA10AC1	-0,202	0,277	0,689
DAP3	-0,203	0,167	0,585
EIF4E	-0,203	0,409	0,781
OTUD6B	-0,203	0,411	0,782
CYP1B1	-0,203	0,502	0,830
SNX3	-0,203	0,162	0,577
PPP1R13L	-0,203	0,248	0,661
SLC25A11	-0,203	0,365	0,754
CINP	-0,204	0,230	0,650

DNM2	-0,204	0,321	0,722
ADGRL2	-0,204	0,236	0,655
CASP4	-0,204	0,410	0,782
DMD	-0,204	0,438	0,797
SRP19	-0,205	0,212	0,633
CLINT1	-0,205	0,095	0,484
SNTB1	-0,205	0,422	0,789
ANKS1A	-0,205	0,345	0,739
ULK3	-0,205	0,186	0,610
POLR2L	-0,206	0,332	0,730
NXPE3	-0,206	0,308	0,715
PPIP5K2	-0,206	0,253	0,666
NCKAP1L	-0,206	0,426	0,791
IPO13	-0,206	0,269	0,682
TRIM55	-0,206	0,480	0,819
ECH1	-0,206	0,439	0,797
ZNF518B	-0,206	0,148	0,557
ABLIM1	-0,206	0,395	0,771
LNPK	-0,207	0,182	0,604
CLPTM1	-0,207	0,225	0,646
COLGALT1	-0,207	0,208	0,629
ING3	-0,207	0,219	0,640
NIPSNAP3A	-0,207	0,438	0,797
RNF141	-0,207	0,325	0,725
AZI2	-0,207	0,193	0,617
CTSH	-0,207	0,196	0,621
COPS9	-0,207	0,213	0,634
TPP1	-0,207	0,224	0,646
FAM118B	-0,207	0,205	0,625
CDH19	-0,207	0,404	0,776
U2AF1L5	-0,207	0,192	0,616
KIN	-0,207	0,237	0,656
L3MBTL4	-0,207	0,241	0,659
ESCO1	-0,207	0,302	0,712
KCMF1	-0,208	0,029	0,314
ALKBH3	-0,208	0,281	0,692
ME3	-0,208	0,158	0,571
ABCA5	-0,208	0,265	0,679
THUMPD3	-0,208	0,083	0,468
RAN	-0,208	0,217	0,638
GSTM2	-0,208	0,320	0,721
PDLIM5	-0,209	0,452	0,805
STARD9	-0,209	0,436	0,796
SVIP	-0,209	0,369	0,756
VMP1	-0,209	0,616	0,887
SLC25A28	-0,209	0,173	0,591
RAPGEF3	-0,209	0,355	0,746
SLC9A6	-0,209	0,075	0,451
RMDN1	-0,209	0,160	0,575
ORC3	-0,209	0,399	0,774
TPST1	-0,210	0,224	0,646
CST3	-0,210	0,320	0,721
ITPKB	-0,210	0,350	0,744

MTFR1L	-0,210	0,233	0,654
MCEE	-0,210	0,324	0,724
VSNL1	-0,210	0,529	0,845
NGDN	-0,210	0,194	0,618
FHL1	-0,210	0,536	0,850
PSMD11	-0,210	0,060	0,415
PNKD	-0,211	0,247	0,661
EEF1AKNMT	-0,211	0,183	0,605
BTNL9	-0,211	0,553	0,856
NUDT4	-0,211	0,207	0,628
TNRC6C	-0,211	0,370	0,756
POLDIP2	-0,211	0,146	0,556
SARS	-0,211	0,112	0,508
CRIP2	-0,211	0,455	0,806
HIBADH	-0,211	0,081	0,464
DCXR	-0,211	0,331	0,730
FOXRED1	-0,212	0,241	0,659
ETFB	-0,212	0,432	0,794
UTP6	-0,212	0,105	0,497
NUCB2	-0,212	0,381	0,761
C19orf47	-0,212	0,307	0,714
GARS	-0,212	0,044	0,370
CCSER1	-0,212	0,469	0,815
BLVRB	-0,212	0,380	0,761
CDIP1	-0,213	0,310	0,715
PDZD11	-0,213	0,181	0,602
OCIAD1	-0,213	0,095	0,484
SUPV3L1	-0,213	0,087	0,475
LRP12	-0,213	0,125	0,527
SEC23A	-0,213	0,168	0,585
CDC37	-0,213	0,190	0,614
POLB	-0,214	0,193	0,617
TATDN1	-0,214	0,271	0,684
ZNF672	-0,214	0,191	0,616
FBXO45	-0,215	0,205	0,625
MRPL57	-0,215	0,123	0,525
GTF2A2	-0,215	0,169	0,587
SDF2	-0,215	0,123	0,525
NOL7	-0,215	0,090	0,480
U2AF1	-0,215	0,173	0,591
SF3B6	-0,215	0,393	0,769
RAB9B	-0,215	0,494	0,826
ACSS1	-0,215	0,354	0,744
MED21	-0,216	0,258	0,671
OXNAD1	-0,216	0,096	0,484
DLST	-0,216	0,183	0,605
TSC22D1	-0,216	0,107	0,502
PRKAG1	-0,216	0,171	0,588
SEC11C	-0,216	0,323	0,724
KIAA2026	-0,216	0,048	0,380
TMEM120A	-0,216	0,326	0,725
DCUN1D5	-0,216	0,160	0,575
CYB5B	-0,216	0,114	0,511

PPM1K	-0,216	0,615	0,887
MPZL1	-0,216	0,365	0,754
HIGD2A	-0,217	0,152	0,564
PDCD2	-0,217	0,086	0,473
HSD17B10	-0,217	0,245	0,661
SLC25A46	-0,217	0,303	0,712
CBR4	-0,217	0,086	0,474
MPZL2	-0,217	0,370	0,756
MAP7D1	-0,217	0,259	0,674
CALM1	-0,217	0,215	0,637
SRP14	-0,218	0,111	0,506
ABCB7	-0,218	0,118	0,518
TNNC1	-0,218	0,499	0,829
MFSD11	-0,218	0,091	0,481
WWP1	-0,218	0,066	0,431
PECAM1	-0,218	0,195	0,618
RNF10	-0,218	0,244	0,661
FEM1B	-0,219	0,146	0,556
PLEKHA7	-0,219	0,481	0,820
MAPK9	-0,219	0,025	0,295
ZRANB1	-0,219	0,175	0,594
NDUFA10	-0,219	0,153	0,565
TVP23C	-0,219	0,233	0,654
PEPD	-0,220	0,217	0,638
MYLK3	-0,220	0,469	0,815
RNF167	-0,220	0,164	0,579
GDE1	-0,220	0,076	0,454
SRPK1	-0,220	0,139	0,546
RPUSD4	-0,220	0,132	0,537
OGDH	-0,220	0,384	0,764
ANAPC11	-0,220	0,303	0,712
USP15	-0,221	0,320	0,721
MTMR6	-0,221	0,261	0,676
CCT6A	-0,221	0,173	0,592
COPS6	-0,221	0,144	0,554
KPNA3	-0,221	0,220	0,642
PRELID3B	-0,221	0,334	0,731
CCNG1	-0,221	0,357	0,747
CLU	-0,222	0,525	0,843
RAPGEF1	-0,222	0,332	0,730
TEX264	-0,222	0,226	0,646
RBX1	-0,222	0,178	0,599
TUBB	-0,223	0,263	0,677
TBK1	-0,223	0,219	0,641
REEP5	-0,223	0,121	0,522
RCSD1	-0,223	0,107	0,501
BRMS1L	-0,223	0,201	0,624
XRN2	-0,223	0,125	0,527
PDCL3	-0,223	0,223	0,646
CTNNAL1	-0,223	0,196	0,621
HSPB7	-0,223	0,399	0,774
HECA	-0,224	0,223	0,646
LTBR	-0,224	0,317	0,720

SNRPB2	-0,224	0,201	0,624
DCP1B	-0,224	0,508	0,833
CNEP1R1	-0,224	0,197	0,621
NDUFA3	-0,224	0,318	0,721
ZC3HC1	-0,224	0,192	0,616
ATP6V1G1	-0,224	0,243	0,660
TET2	-0,224	0,073	0,447
DMTN	-0,224	0,369	0,756
GPN3	-0,224	0,226	0,646
MRAS	-0,224	0,222	0,644
PSMC5	-0,224	0,223	0,645
PANX1	-0,225	0,182	0,604
XRCC6	-0,225	0,205	0,625
UBR3	-0,225	0,265	0,678
RBM6	-0,225	0,187	0,611
CIAPIN1	-0,225	0,057	0,405
SMIM11B	-0,225	0,178	0,599
SYNGR1	-0,225	0,264	0,678
TCERG1	-0,225	0,104	0,495
SLC48A1	-0,225	0,295	0,705
TIMM50	-0,226	0,224	0,646
FRMD3	-0,226	0,426	0,791
DSTN	-0,226	0,157	0,569
THYN1	-0,226	0,204	0,625
CCNH	-0,226	0,142	0,551
MRS2	-0,226	0,204	0,625
SLC2A11	-0,227	0,287	0,699
COMMD1	-0,227	0,208	0,629
CASD1	-0,227	0,301	0,711
IMMT	-0,227	0,201	0,624
MT-CO2	-0,227	0,384	0,764
TLR2	-0,227	0,529	0,845
MEGF9	-0,227	0,279	0,690
INTS6L	-0,227	0,228	0,648
DAAM2	-0,227	0,465	0,812
FANCC	-0,227	0,443	0,798
AP2S1	-0,227	0,234	0,654
TTC37	-0,228	0,416	0,784
ENTPD4	-0,228	0,143	0,551
NAE1	-0,228	0,177	0,597
AOPEP	-0,228	0,224	0,646
CYP7B1	-0,228	0,123	0,525
RAVER2	-0,228	0,058	0,411
GFM1	-0,228	0,172	0,589
INPP1	-0,228	0,123	0,525
CHD7	-0,229	0,226	0,646
GPR137B	-0,229	0,078	0,459
ACTR6	-0,229	0,393	0,769
DHX15	-0,229	0,156	0,568
POLE3	-0,229	0,134	0,540
DCAF6	-0,229	0,151	0,562
THUMPD2	-0,229	0,184	0,606
EPN1	-0,229	0,263	0,677

OSBPL6	-0,229	0,263	0,676
THAP2	-0,229	0,342	0,738
NIPA2	-0,230	0,123	0,525
USP38	-0,230	0,092	0,482
TSPAN7	-0,230	0,077	0,458
RAB10	-0,230	0,075	0,452
MSANTD4	-0,230	0,192	0,616
CDK16	-0,230	0,183	0,605
ARFGAP3	-0,230	0,093	0,484
S1PR1	-0,231	0,138	0,546
SSH2	-0,231	0,087	0,475
CCL2	-0,231	0,673	0,908
TXNL4A	-0,231	0,086	0,473
EMC8	-0,231	0,194	0,618
MAFB	-0,231	0,413	0,782
LPIN1	-0,231	0,274	0,687
ATE1	-0,231	0,146	0,556
ARL6IP5	-0,231	0,125	0,527
LYSMD4	-0,231	0,177	0,596
NDRG4	-0,231	0,455	0,807
POLR2B	-0,231	0,217	0,638
IFITM3	-0,231	0,440	0,797
DHRS7	-0,232	0,083	0,468
RUFY3	-0,232	0,073	0,447
EIF2B4	-0,232	0,116	0,514
SIRT5	-0,232	0,212	0,633
PCDHGA10	-0,232	0,392	0,769
UBXN2B	-0,232	0,120	0,521
ENTPD1	-0,232	0,144	0,554
SCD	-0,232	0,736	0,932
CAB39L	-0,232	0,230	0,649
SCAMP1	-0,232	0,369	0,756
POT1	-0,232	0,217	0,638
BTBD1	-0,233	0,372	0,756
ABCC9	-0,233	0,157	0,569
MLH1	-0,233	0,157	0,569
GTPBP4	-0,233	0,099	0,489
ZDHHC17	-0,233	0,243	0,660
RMND1	-0,234	0,140	0,547
P4HA1	-0,234	0,307	0,714
ACTR1A	-0,234	0,108	0,502
MARCH1	-0,234	0,425	0,791
CBWD5	-0,234	0,063	0,425
GNPDA2	-0,234	0,328	0,726
STX8	-0,234	0,157	0,569
SNX7	-0,234	0,171	0,588
ZNF415	-0,234	0,286	0,699
ADAM23	-0,234	0,337	0,732
S100A16	-0,234	0,233	0,654
FOXK2	-0,235	0,239	0,657
NIF3L1	-0,235	0,161	0,577
SOCS6	-0,235	0,198	0,622
ZNF644	-0,235	0,274	0,687

SUB1	-0,236	0,411	0,782
FEZ2	-0,236	0,125	0,527
LACTB2	-0,236	0,266	0,679
THNSL2	-0,236	0,297	0,708
HIPK3	-0,236	0,140	0,548
TXN2	-0,236	0,158	0,571
NEIL2	-0,237	0,130	0,535
PSMD1	-0,237	0,142	0,551
KAT2B	-0,237	0,175	0,595
PRPSAP1	-0,237	0,077	0,458
BAG3	-0,237	0,316	0,719
EMG1	-0,237	0,118	0,518
SERINC1	-0,237	0,304	0,712
FDPS	-0,238	0,083	0,468
GTF3C3	-0,238	0,202	0,624
SRRD	-0,238	0,193	0,617
PSMC3	-0,238	0,210	0,631
HDGF	-0,238	0,133	0,540
TECR	-0,238	0,237	0,656
HSD17B4	-0,238	0,109	0,504
GGPS1	-0,238	0,196	0,620
ZNF181	-0,238	0,228	0,648
ECPAS	-0,238	0,042	0,365
KRR1	-0,239	0,212	0,633
RPS26	-0,239	0,431	0,794
NPTN	-0,239	0,058	0,411
BID	-0,239	0,201	0,624
NSMCE2	-0,239	0,163	0,578
CDK5RAP1	-0,239	0,096	0,484
PDLIM1	-0,239	0,181	0,602
CCDC25	-0,239	0,209	0,629
RPP30	-0,240	0,135	0,542
ABCA6	-0,240	0,312	0,716
CRIPT	-0,240	0,104	0,495
RGN	-0,240	0,196	0,620
CPNE3	-0,240	0,256	0,669
RPRD1B	-0,241	0,105	0,497
RASGRP3	-0,241	0,324	0,724
ESRRA	-0,241	0,279	0,691
OPA1	-0,241	0,314	0,718
JPH1	-0,241	0,286	0,699
NARF	-0,241	0,103	0,495
SMARCE1	-0,241	0,085	0,473
PSMA5	-0,241	0,173	0,591
PRDX2	-0,241	0,294	0,705
RNASEH2B	-0,242	0,085	0,472
CLTB	-0,242	0,267	0,679
ZYX	-0,242	0,376	0,759
MLH3	-0,242	0,046	0,378
DPF3	-0,243	0,444	0,799
AK3	-0,243	0,183	0,605
DNTTIP2	-0,243	0,174	0,593
PNO1	-0,243	0,201	0,624

SDHA	-0,243	0,303	0,712
UEVLD	-0,243	0,164	0,579
CYB5A	-0,243	0,362	0,751
PHF5A	-0,244	0,193	0,617
BANF1	-0,244	0,195	0,619
PKM	-0,244	0,171	0,588
HGF	-0,244	0,479	0,819
NEURL1B	-0,244	0,251	0,665
CHCHD2	-0,244	0,078	0,459
ECI1	-0,244	0,217	0,638
MRPL24	-0,245	0,095	0,484
PIGP	-0,245	0,129	0,533
TTF1	-0,245	0,096	0,484
SCHIP1	-0,245	0,379	0,761
SLC25A36	-0,245	0,263	0,677
FAM169A	-0,245	0,373	0,756
EIF2A	-0,245	0,290	0,701
UBE2E3	-0,246	0,050	0,385
SAT2	-0,246	0,109	0,504
IGSF3	-0,246	0,242	0,659
ZCCHC10	-0,246	0,305	0,713
MTRF1	-0,246	0,209	0,630
OBI1	-0,246	0,247	0,661
EIF1B	-0,246	0,243	0,660
FKBP4	-0,246	0,194	0,618
TYW1	-0,246	0,053	0,394
SMC2	-0,246	0,205	0,625
GLIS3	-0,246	0,221	0,643
CS	-0,246	0,206	0,626
NAA15	-0,246	0,117	0,516
MARK3	-0,246	0,095	0,484
EIF2B2	-0,247	0,137	0,545
MYL9	-0,247	0,373	0,756
SUMO1	-0,247	0,146	0,556
MRPS35	-0,247	0,228	0,648
PERP	-0,247	0,178	0,599
RNF19A	-0,247	0,151	0,563
ZNF407	-0,247	0,022	0,285
RAMP3	-0,247	0,344	0,739
CD58	-0,247	0,189	0,614
FTSJ3	-0,247	0,119	0,518
PPP1R3B	-0,247	0,075	0,451
MAT2A	-0,247	0,320	0,721
BCCIP	-0,247	0,121	0,521
TSHZ2	-0,248	0,240	0,659
FADS3	-0,248	0,166	0,583
RIOX2	-0,248	0,118	0,518
DAD1	-0,248	0,203	0,625
DSC2	-0,248	0,077	0,456
APOL3	-0,248	0,174	0,593
WNK1	-0,248	0,226	0,646
TTL	-0,248	0,047	0,380
LYAR	-0,249	0,162	0,577

**Protein coding**	LIAS	-0,249	0,160	0,575
	RABGGTB	-0,249	0,245	0,661
	MCF2L	-0,249	0,299	0,710
	MUL1	-0,250	0,142	0,551
	PODN	-0,250	0,324	0,724
	NMNAT3	-0,250	0,151	0,562
	PLCG2	-0,250	0,129	0,532
	DNM1L	-0,250	0,131	0,535
	METAP1	-0,250	0,038	0,348
	MAN1A1	-0,250	0,368	0,756
	SLC12A2	-0,250	0,243	0,660
	CTSC	-0,250	0,318	0,721
	CUL5	-0,250	0,288	0,700
	TRMU	-0,250	0,106	0,500
	MMACHC	-0,250	0,167	0,585
	LYRM2	-0,250	0,052	0,388
	ARHGAP29	-0,251	0,056	0,404
	PTPRE	-0,251	0,189	0,614
	NDUFS1	-0,251	0,323	0,724
	LAMTOR3	-0,251	0,262	0,676
	UBL5	-0,251	0,145	0,554
	TRIM54	-0,251	0,314	0,718
	ACAT1	-0,252	0,343	0,738
	PTS	-0,252	0,236	0,655
	UQCC1	-0,252	0,109	0,503
	REXO2	-0,252	0,134	0,540
	JPH2	-0,253	0,394	0,771
	UBE2D4	-0,253	0,116	0,514
	TSNAX	-0,253	0,162	0,578
	RBM18	-0,253	0,246	0,661
	ASAH2B	-0,253	0,307	0,714
	ZNF75A	-0,253	0,070	0,442
	SELENOT	-0,253	0,256	0,669
	PTP4A2	-0,254	0,099	0,489
	NDUFAF6	-0,254	0,130	0,535
	LCP1	-0,254	0,441	0,797
	RAP1A	-0,254	0,123	0,525
	NTPCR	-0,254	0,058	0,410
	HYOU1	-0,254	0,284	0,696
	HNRNPA2B1	-0,254	0,045	0,375
	MGST2	-0,254	0,128	0,531
	PREX1	-0,254	0,383	0,764
	MED7	-0,254	0,161	0,577
	TMEM19	-0,255	0,091	0,481
	KANSL2	-0,255	0,050	0,383
	CAMTA1	-0,255	0,128	0,531
	UBC	-0,255	0,290	0,701
	B3GLCT	-0,256	0,263	0,676
	PIGS	-0,256	0,130	0,534
	CHIC1	-0,256	0,239	0,657
	SOX5	-0,256	0,153	0,565
	MRPL41	-0,256	0,242	0,659
	MSRB2	-0,257	0,153	0,565

POP4	-0,257	0,103	0,495
ITGB2	-0,257	0,380	0,761
NEGR1	-0,257	0,326	0,725
TAF8	-0,257	0,181	0,602
PLA2G5	-0,257	0,145	0,555
HSPB11	-0,257	0,184	0,606
CCDC85C	-0,258	0,224	0,646
CRADD	-0,258	0,110	0,505
TPK1	-0,258	0,221	0,643
SPHKAP	-0,258	0,526	0,844
HADHA	-0,258	0,141	0,550
ABRAXAS2	-0,258	0,155	0,568
GNL3	-0,258	0,145	0,555
PCDH17	-0,258	0,233	0,654
PLAAT3	-0,259	0,274	0,687
DHRSX	-0,260	0,106	0,499
NAGA	-0,260	0,156	0,568
ITGAV	-0,260	0,284	0,696
EXOSC8	-0,260	0,119	0,518
ANKRD26	-0,260	0,147	0,556
PLPP7	-0,260	0,193	0,617
RARRES1	-0,260	0,624	0,889
CISD2	-0,261	0,205	0,625
DNAJB5	-0,261	0,253	0,666
TXNDC15	-0,261	0,017	0,251
SLC7A6	-0,261	0,306	0,714
STAB1	-0,261	0,284	0,696
RHEB	-0,261	0,067	0,434
C18orf25	-0,261	0,154	0,566
IWS1	-0,261	0,027	0,305
MDM1	-0,261	0,153	0,565
SELENOI	-0,261	0,128	0,531
RPS19BP1	-0,261	0,209	0,630
OPA3	-0,261	0,238	0,657
CEBPG	-0,261	0,050	0,385
CCDC9B	-0,262	0,300	0,710
LMBR1	-0,262	0,026	0,300
NAA25	-0,262	0,054	0,395
BLVRA	-0,262	0,068	0,436
NNT	-0,263	0,349	0,743
MFF	-0,263	0,022	0,284
KIAA0513	-0,263	0,290	0,701
H2AFY	-0,263	0,049	0,383
PPP2R3C	-0,263	0,343	0,738
PALLD	-0,263	0,187	0,612
INTS13	-0,263	0,085	0,472
CEBPZOS	-0,264	0,054	0,394
ADAMTS5	-0,264	0,402	0,774
TAZ	-0,264	0,146	0,556
FERMT2	-0,264	0,084	0,470
RBPJ	-0,264	0,051	0,388
PTGR1	-0,264	0,091	0,481
NDUFA2	-0,264	0,219	0,640

PRKCE	-0,265	0,116	0,514
SLC7A1	-0,265	0,279	0,691
TUBA1C	-0,265	0,127	0,529
STARD8	-0,265	0,225	0,646
HCN1	-0,266	0,482	0,821
STRAP	-0,266	0,027	0,304
BLZF1	-0,266	0,334	0,731
TPMT	-0,266	0,148	0,558
C16orf87	-0,266	0,162	0,577
ZDHHC16	-0,266	0,092	0,481
CDK2AP1	-0,266	0,061	0,416
INTS7	-0,266	0,103	0,495
ATP6V1F	-0,267	0,080	0,463
KTN1	-0,267	0,185	0,609
CERS6	-0,267	0,262	0,676
TUBE1	-0,267	0,262	0,676
BRIX1	-0,267	0,131	0,536
PIN4	-0,268	0,148	0,557
APTX	-0,268	0,060	0,416
RAMAC	-0,268	0,191	0,616
APOL2	-0,268	0,111	0,506
MTFR1	-0,268	0,128	0,531
TMEM14B	-0,268	0,211	0,632
C12orf4	-0,268	0,213	0,634
PTGDS	-0,268	0,480	0,819
CDO1	-0,268	0,505	0,832
MYO19	-0,268	0,143	0,551
DAZAP1	-0,268	0,106	0,501
COPS8	-0,269	0,202	0,624
SMTN	-0,269	0,268	0,681
TAF11	-0,269	0,088	0,477
CYTH4	-0,269	0,313	0,718
LRIF1	-0,269	0,146	0,556
PGM2L1	-0,269	0,337	0,732
TNFRSF1B	-0,270	0,247	0,661
CA8	-0,270	0,367	0,755
DPH6	-0,270	0,247	0,661
ORC4	-0,270	0,130	0,535
TMEM50B	-0,270	0,043	0,366
GPR89A	-0,270	0,051	0,388
MMUT	-0,270	0,145	0,554
BCKDK	-0,270	0,115	0,514
ANKMY2	-0,270	0,292	0,703
IFITM2	-0,271	0,354	0,744
C1QB	-0,271	0,441	0,797
MRPL17	-0,271	0,056	0,403
MICOS13	-0,271	0,252	0,666
RPS6KA5	-0,271	0,200	0,624
USP16	-0,271	0,230	0,650
UBE2D1	-0,272	0,273	0,686
VPS50	-0,272	0,251	0,665
KBTBD8	-0,272	0,506	0,832
TEAD1	-0,272	0,066	0,431

LSG1	-0,273	0,022	0,284
GPD1L	-0,273	0,333	0,730
FEM1C	-0,273	0,181	0,601
TUSC2	-0,273	0,080	0,463
NDUFS8	-0,273	0,253	0,666
NKTR	-0,273	0,100	0,489
LSM8	-0,274	0,077	0,456
PSMD4	-0,274	0,095	0,484
PSMD13	-0,274	0,032	0,325
PGM1	-0,274	0,066	0,431
FADS1	-0,274	0,146	0,556
C19orf12	-0,274	0,048	0,380
A2M	-0,274	0,127	0,530
COA3	-0,275	0,076	0,452
FOLR2	-0,275	0,234	0,654
MRPL40	-0,275	0,195	0,619
XYLT1	-0,275	0,247	0,661
SNF8	-0,275	0,096	0,484
SEPTIN9	-0,275	0,222	0,644
ARRB2	-0,275	0,305	0,713
LPAR6	-0,276	0,330	0,729
CCT7	-0,276	0,050	0,383
ATIC	-0,276	0,188	0,613
ZNF589	-0,276	0,134	0,540
TLE5	-0,276	0,144	0,554
CSRNP3	-0,276	0,289	0,701
BCO2	-0,277	0,235	0,655
SLC30A9	-0,277	0,273	0,686
ZBED5	-0,277	0,044	0,369
ESD	-0,277	0,119	0,518
SLC8B1	-0,277	0,123	0,525
ATF4	-0,277	0,076	0,453
AK6	-0,277	0,301	0,711
MTSS1	-0,277	0,137	0,545
AGFG1	-0,277	0,094	0,484
DEK	-0,277	0,085	0,472
GPR89B	-0,278	0,034	0,332
MLLT11	-0,278	0,390	0,768
GAPDH	-0,278	0,229	0,649
KANSL1	-0,278	0,175	0,594
FBXW7	-0,278	0,216	0,637
DSP	-0,278	0,339	0,735
ATPSCKMT	-0,279	0,120	0,519
COA4	-0,279	0,073	0,448
PDGFRA	-0,279	0,259	0,674
GRPEL1	-0,279	0,029	0,316
CD53	-0,279	0,312	0,717
HSD17B12	-0,279	0,156	0,569
SPAG7	-0,280	0,124	0,526
GOT2	-0,280	0,243	0,660
GAN	-0,280	0,144	0,554
ADAM19	-0,280	0,371	0,756
NEDD8	-0,280	0,081	0,464

THOC5	-0,280	0,044	0,370
ST3GAL2	-0,280	0,154	0,566
STK40	-0,280	0,111	0,506
RAB4A	-0,281	0,054	0,395
OCRL	-0,281	0,034	0,334
IDE	-0,281	0,139	0,547
DYSF	-0,281	0,325	0,724
THAP4	-0,281	0,198	0,622
RAB12	-0,282	0,106	0,500
CYBC1	-0,282	0,192	0,616
ZCRB1	-0,282	0,156	0,568
PSMA1	-0,282	0,218	0,638
TAF13	-0,282	0,158	0,571
TXNDC9	-0,283	0,269	0,682
ZDHHC11B	-0,283	0,292	0,703
NKAPD1	-0,283	0,074	0,450
TCP1	-0,283	0,028	0,309
C14orf119	-0,283	0,048	0,380
PRDX5	-0,283	0,174	0,593
MANF	-0,283	0,279	0,691
FLOT1	-0,283	0,071	0,446
GALNT2	-0,283	0,131	0,536
SHLD2	-0,283	0,171	0,588
LDB3	-0,283	0,406	0,777
CNKSR3	-0,284	0,271	0,684
MTREX	-0,284	0,187	0,611
RRP36	-0,284	0,067	0,434
NADK2	-0,284	0,044	0,370
ANKRD20A4	-0,284	0,344	0,739
C1D	-0,284	0,107	0,501
CEP63	-0,285	0,019	0,271
TYRP1	-0,285	0,400	0,774
DDX1	-0,285	0,151	0,563
OSBPL11	-0,285	0,078	0,459
MTUS2	-0,286	0,294	0,705
HNRNPAB	-0,286	0,085	0,472
NNMT	-0,287	0,402	0,774
MAPRE3	-0,287	0,137	0,546
TMLHE	-0,287	0,074	0,450
GTF3C5	-0,287	0,130	0,535
FARSB	-0,287	0,018	0,257
MCTS1	-0,287	0,037	0,344
GULP1	-0,288	0,283	0,695
MCM4	-0,288	0,102	0,492
GYG1	-0,288	0,090	0,481
GPR155	-0,288	0,068	0,437
PSMA4	-0,288	0,191	0,616
ABCA8	-0,288	0,132	0,537
WDR75	-0,289	0,138	0,546
ARMC8	-0,289	0,039	0,351
RBM28	-0,290	0,078	0,458
RBM17	-0,290	0,017	0,253
XK	-0,290	0,251	0,665

ST7	-0,290	0,014	0,235
NAA40	-0,290	0,189	0,614
EIF2S1	-0,290	0,082	0,467
MOCS2	-0,290	0,059	0,414
AK2	-0,290	0,004	0,143
TUSC3	-0,291	0,100	0,489
FNBP4	-0,291	0,220	0,642
MRPL11	-0,291	0,099	0,489
MAD2L1BP	-0,291	0,088	0,476
NUP93	-0,291	0,025	0,295
SF3B5	-0,291	0,165	0,580
LSM12	-0,291	0,026	0,297
UBAC1	-0,292	0,050	0,383
ST3GAL4	-0,292	0,045	0,373
PRKG1	-0,292	0,076	0,453
METTL22	-0,292	0,118	0,518
PDE3A	-0,292	0,336	0,732
MAT2B	-0,292	0,063	0,424
HPF1	-0,292	0,091	0,481
COQ9	-0,292	0,105	0,497
PLCB1	-0,292	0,150	0,560
SSU72	-0,293	0,090	0,480
MYCT1	-0,293	0,086	0,473
LMBRD2	-0,293	0,252	0,666
SYNE2	-0,293	0,069	0,441
LYRM7	-0,293	0,099	0,489
MCUR1	-0,294	0,039	0,352
NEK7	-0,294	0,407	0,779
MRPS16	-0,294	0,022	0,285
ADCY4	-0,294	0,129	0,533
ATP8B4	-0,294	0,316	0,719
ZNF35	-0,295	0,121	0,522
TCEAL8	-0,295	0,282	0,693
CLDND1	-0,295	0,079	0,461
LIMK2	-0,295	0,176	0,596
RAD51C	-0,295	0,108	0,502
STAT3	-0,295	0,170	0,588
CIAO2B	-0,295	0,146	0,556
MRTO4	-0,295	0,089	0,478
SNRPE	-0,296	0,259	0,674
FRG1	-0,296	0,114	0,512
PPP1R11	-0,297	0,044	0,371
CALM2	-0,297	0,228	0,648
SERPINF1	-0,297	0,163	0,578
NRDC	-0,297	0,013	0,228
NFATC4	-0,297	0,190	0,615
HEATR3	-0,298	0,057	0,408
CMIP	-0,298	0,305	0,713
TPM1	-0,298	0,295	0,706
TAF9B	-0,298	0,269	0,682
CSE1L	-0,298	0,153	0,565
SAMM50	-0,299	0,017	0,253
MFHAS1	-0,299	0,060	0,415

TMEM62	-0,299	0,108	0,502
ACTR10	-0,299	0,082	0,468
COQ6	-0,299	0,114	0,512
AGL	-0,299	0,216	0,637
LGALS3BP	-0,299	0,322	0,723
VLDLR	-0,300	0,110	0,505
PIK3R3	-0,300	0,144	0,554
ARHGAP5	-0,300	0,304	0,712
LGMN	-0,300	0,074	0,450
H1F0	-0,300	0,097	0,485
TXNDC17	-0,300	0,098	0,489
ANAPC16	-0,300	0,061	0,416
COA1	-0,301	0,004	0,143
DMAC2	-0,301	0,083	0,468
MB	-0,301	0,227	0,647
DENND3	-0,301	0,206	0,626
SLC5A3	-0,301	0,440	0,797
TTYH2	-0,302	0,221	0,643
EIF2S2	-0,302	0,072	0,446
TSEN2	-0,302	0,079	0,460
TMEM106B	-0,302	0,266	0,679
NUDC	-0,302	0,124	0,527
ARMT1	-0,302	0,186	0,610
NECAB1	-0,302	0,272	0,685
L2HGDH	-0,302	0,104	0,496
LSM1	-0,302	0,145	0,555
ETV6	-0,303	0,103	0,495
ANAPC7	-0,303	0,017	0,253
CCDC47	-0,303	0,018	0,256
FLAD1	-0,303	0,100	0,489
NABP1	-0,303	0,599	0,882
RPF1	-0,304	0,129	0,534
PSMD6	-0,304	0,059	0,414
TSEN15	-0,304	0,069	0,441
PAK1IP1	-0,304	0,101	0,491
SLCO2B1	-0,304	0,234	0,655
ANKRD46	-0,304	0,222	0,645
NOL11	-0,305	0,119	0,518
CAPS2	-0,305	0,110	0,504
GEN1	-0,305	0,060	0,415
NIPSNAP2	-0,305	0,180	0,600
ZNF195	-0,305	0,015	0,243
CD34	-0,305	0,214	0,634
TMEM63B	-0,305	0,072	0,447
PRUNE2	-0,305	0,372	0,756
ZNF480	-0,306	0,103	0,494
CDC123	-0,306	0,099	0,489
HSPA5	-0,306	0,276	0,688
MYL2	-0,306	0,480	0,819
LTN1	-0,306	0,217	0,638
C1QBP	-0,306	0,020	0,278
COPS2	-0,307	0,284	0,696
NGLY1	-0,307	0,030	0,319

DRG1	-0,307	0,078	0,459
NKIRAS1	-0,307	0,063	0,424
FABP3	-0,307	0,312	0,717
MPHOSPH9	-0,307	0,089	0,478
INTS12	-0,307	0,102	0,493
SERPINB1	-0,308	0,179	0,600
LTV1	-0,308	0,114	0,512
LARP7	-0,308	0,135	0,541
MAPKAPK3	-0,308	0,164	0,579
PSMB5	-0,308	0,028	0,309
CCDC92	-0,308	0,091	0,481
MOCS1	-0,308	0,199	0,623
QPRT	-0,309	0,122	0,525
MICAL1	-0,309	0,170	0,588
BCAS2	-0,309	0,172	0,589
ENY2	-0,309	0,075	0,452
CARS	-0,309	0,079	0,460
CLCN4	-0,309	0,143	0,551
WASHC2C	-0,309	0,170	0,588
INAFM2	-0,309	0,083	0,468
C1orf122	-0,309	0,104	0,496
PARK7	-0,309	0,068	0,436
OLA1	-0,310	0,075	0,452
ZDHHC21	-0,310	0,180	0,600
CYP2R1	-0,310	0,082	0,467
VPS29	-0,311	0,039	0,351
MRPL16	-0,311	0,051	0,387
NFE2L3	-0,311	0,126	0,527
DNAJC11	-0,311	0,132	0,536
NIT2	-0,311	0,010	0,207
ZNF143	-0,312	0,087	0,475
CNN1	-0,313	0,362	0,751
S100A13	-0,313	0,139	0,547
NDUFAF7	-0,313	0,060	0,415
COQ5	-0,313	0,035	0,336
HCLS1	-0,313	0,266	0,679
EIF1AY	-0,314	0,656	0,900
COMMD9	-0,315	0,034	0,334
PGD	-0,315	0,285	0,699
B4GALT5	-0,315	0,075	0,452
LYRM1	-0,315	0,128	0,532
S100A4	-0,315	0,288	0,700
IFNGR1	-0,315	0,050	0,387
NAA38	-0,316	0,168	0,587
MAST2	-0,316	0,211	0,632
ATP5F1B	-0,316	0,125	0,527
GNPAT	-0,316	0,050	0,385
GET1	-0,316	0,135	0,542
RPGR	-0,316	0,094	0,484
PDZRN3	-0,316	0,120	0,521
PSMA7	-0,316	0,051	0,388
NALCN	-0,316	0,196	0,620
PNPLA8	-0,316	0,228	0,648

PPP1R13B	-0,316	0,139	0,547
ZNF32	-0,316	0,032	0,327
SLC11A2	-0,316	0,015	0,239
SLC4A7	-0,317	0,177	0,598
SLC25A25	-0,317	0,224	0,646
GIN1	-0,317	0,148	0,557
AUTS2	-0,317	0,061	0,416
PDCD10	-0,317	0,142	0,551
ADAL	-0,318	0,154	0,566
SYNPO2	-0,318	0,047	0,379
PPM1B	-0,318	0,048	0,380
COL4A6	-0,319	0,185	0,609
SYPL1	-0,319	0,062	0,422
DCAF13	-0,319	0,156	0,569
TCEAL1	-0,319	0,104	0,496
MPC1	-0,319	0,238	0,656
BUD23	-0,319	0,043	0,365
ZNF658	-0,319	0,175	0,594
TTPAL	-0,320	0,180	0,600
GRSF1	-0,320	0,016	0,249
ATP1A1	-0,320	0,199	0,623
AK9	-0,320	0,056	0,403
EIF3K	-0,321	0,070	0,442
LACTB	-0,321	0,047	0,379
ABL1	-0,321	0,196	0,621
RPRD1A	-0,321	0,094	0,484
PON2	-0,321	0,028	0,309
FAM78A	-0,321	0,194	0,618
CBWD2	-0,321	0,046	0,378
UBE2N	-0,322	0,095	0,484
TMA7	-0,322	0,259	0,674
PCSK5	-0,322	0,167	0,585
PNPT1	-0,322	0,022	0,284
SNAPC5	-0,323	0,084	0,470
RYR2	-0,323	0,553	0,857
SCAPER	-0,323	0,051	0,388
UBE2M	-0,323	0,071	0,445
ESRRG	-0,323	0,313	0,718
MPI	-0,323	0,068	0,436
ZSCAN9	-0,324	0,086	0,474
CHCHD7	-0,324	0,173	0,591
TSPAN12	-0,324	0,258	0,672
IL4R	-0,324	0,313	0,718
DRAM2	-0,325	0,108	0,503
GSTM3	-0,325	0,107	0,502
NEBL	-0,325	0,449	0,802
LIMS2	-0,325	0,208	0,629
COL5A3	-0,325	0,229	0,649
STAU2	-0,325	0,111	0,506
ADRB1	-0,326	0,311	0,716
OSTM1	-0,326	0,121	0,521
DNMT1	-0,326	0,151	0,562
TMEM59	-0,326	0,067	0,434

B3GALNT2	-0,326	0,014	0,235
GNAO1	-0,326	0,370	0,756
STOML2	-0,326	0,037	0,344
TRIT1	-0,326	0,028	0,309
SEM1	-0,326	0,018	0,264
CCNB1IP1	-0,327	0,037	0,346
WASHC3	-0,327	0,037	0,345
PIP4P2	-0,327	0,066	0,431
MRPS11	-0,327	0,061	0,416
CACNA2D3	-0,327	0,113	0,511
PODXL	-0,328	0,135	0,541
MAN2A2	-0,328	0,240	0,659
KARS	-0,328	0,039	0,352
EMC7	-0,328	0,005	0,152
HHATL	-0,328	0,113	0,510
ESAM	-0,328	0,072	0,446
COBL	-0,329	0,095	0,484
CPAMD8	-0,329	0,438	0,797
TMEM178B	-0,329	0,384	0,764
RARB	-0,329	0,075	0,452
SMIM10L1	-0,329	0,107	0,502
FUNDC1	-0,329	0,119	0,518
CEMIP2	-0,329	0,366	0,755
PID1	-0,330	0,237	0,656
TMEM242	-0,330	0,018	0,264
ABCD3	-0,330	0,171	0,588
CFL2	-0,330	0,360	0,750
RRAGD	-0,330	0,166	0,584
UQCC2	-0,331	0,119	0,518
LRP2BP	-0,331	0,224	0,646
ZNF436	-0,331	0,140	0,547
VRK2	-0,331	0,031	0,320
NCF2	-0,331	0,303	0,712
EMC2	-0,332	0,153	0,565
FAM229B	-0,332	0,087	0,475
COQ2	-0,332	0,082	0,466
DDX21	-0,332	0,130	0,534
ZDHHC13	-0,332	0,043	0,368
VTA1	-0,332	0,145	0,554
TADA1	-0,333	0,056	0,402
CCT2	-0,333	0,097	0,485
MRPL3	-0,333	0,125	0,527
NUDT15	-0,333	0,098	0,487
KCNA5	-0,333	0,204	0,625
ZBTB8OS	-0,333	0,069	0,441
MED9	-0,333	0,041	0,361
SLC41A1	-0,334	0,237	0,656
SMIM4	-0,334	0,060	0,416
PROM1	-0,334	0,273	0,687
MRPL18	-0,334	0,021	0,283
S100A1	-0,334	0,155	0,567
MRPS18B	-0,334	0,016	0,244
ACACB	-0,335	0,410	0,782

C3AR1	-0,335	0,208	0,629
PLIN2	-0,335	0,370	0,756
MAP2K3	-0,335	0,204	0,625
TIMM44	-0,336	0,044	0,370
HSP90AA1	-0,336	0,202	0,624
PLD1	-0,336	0,132	0,536
ACADM	-0,337	0,226	0,646
DRAM1	-0,337	0,139	0,547
TAX1BP1	-0,337	0,121	0,522
CHRM2	-0,337	0,281	0,692
CRYZ	-0,337	0,105	0,497
RUVBL1	-0,338	0,022	0,285
PSMC1	-0,338	0,144	0,553
SNRPD1	-0,339	0,028	0,308
STARD7	-0,339	0,022	0,285
TPST2	-0,339	0,035	0,336
RAF1	-0,339	0,035	0,336
PPARGC1A	-0,339	0,342	0,737
HBS1L	-0,339	0,110	0,505
ITPKC	-0,339	0,209	0,630
PLAT	-0,339	0,203	0,625
AHSA1	-0,340	0,063	0,425
ENOSF1	-0,341	0,166	0,583
ZMPSTE24	-0,341	0,033	0,330
PTPRB	-0,341	0,094	0,484
MED20	-0,341	0,063	0,424
TAGLN	-0,341	0,288	0,700
SFXN4	-0,341	0,024	0,290
SNN	-0,342	0,067	0,434
SLC19A2	-0,342	0,227	0,647
RPE	-0,342	0,034	0,332
RTN4	-0,342	0,019	0,271
TC2N	-0,342	0,288	0,700
CLCN3	-0,343	0,023	0,287
SEPHS2	-0,343	0,022	0,285
FASTKD2	-0,343	0,104	0,495
KCND3	-0,343	0,104	0,495
P4HA2	-0,344	0,075	0,452
PLAG1	-0,344	0,280	0,692
PGS1	-0,344	0,100	0,489
PDIA4	-0,344	0,250	0,663
UQCRQ	-0,344	0,170	0,588
ZNF330	-0,344	0,089	0,478
RIDA	-0,345	0,204	0,625
STOM	-0,345	0,010	0,207
FAM234A	-0,345	0,147	0,556
C1GALT1C1	-0,345	0,109	0,503
OSBPL1A	-0,346	0,011	0,210
MRPL35	-0,346	0,174	0,594
NDUFB5	-0,346	0,161	0,577
CYBB	-0,346	0,287	0,699
TGFBR2	-0,346	0,036	0,339
OPHN1	-0,346	0,039	0,352

ETS2	-0,346	0,088	0,477
RBM4	-0,347	0,065	0,429
SPRTN	-0,347	0,060	0,416
MTMR9	-0,347	0,023	0,285
LRRC37A2	-0,348	0,115	0,512
UTP15	-0,348	0,034	0,333
PHC2	-0,348	0,066	0,431
CBWD1	-0,348	0,045	0,372
ECT2	-0,348	0,109	0,503
DCBLD2	-0,348	0,084	0,472
FAM210A	-0,348	0,082	0,467
RAB6B	-0,348	0,120	0,521
TLNRD1	-0,349	0,070	0,441
NANS	-0,349	0,044	0,369
COMMD10	-0,349	0,125	0,527
CBWD6	-0,349	0,010	0,207
BEX3	-0,350	0,054	0,396
FOXN2	-0,350	0,217	0,638
PLCXD3	-0,350	0,149	0,559
ASCC1	-0,350	0,018	0,256
SLCO3A1	-0,350	0,040	0,358
GLRX3	-0,350	0,017	0,254
NDUFAF2	-0,351	0,079	0,460
LYPLA1	-0,351	0,149	0,558
PIK3AP1	-0,351	0,136	0,543
UCHL5	-0,352	0,048	0,380
POLR2K	-0,352	0,145	0,555
RAB11A	-0,352	0,015	0,237
SMIM26	-0,352	0,099	0,489
BAG2	-0,353	0,078	0,459
METAP2	-0,353	0,098	0,488
CLIC2	-0,353	0,151	0,562
VDAC1	-0,353	0,104	0,495
AURKAIP1	-0,353	0,154	0,566
SMARCA1	-0,353	0,023	0,285
CD36	-0,353	0,253	0,666
MMP2	-0,353	0,116	0,514
PPP1R1C	-0,354	0,379	0,761
ZNF25	-0,354	0,082	0,467
TXNL1	-0,355	0,017	0,251
PRXL2C	-0,355	0,034	0,332
ECI2	-0,355	0,083	0,468
BCAP29	-0,356	0,131	0,535
GFM2	-0,356	0,183	0,605
NEK10	-0,356	0,291	0,702
SCN7A	-0,356	0,164	0,579
SNRPA1	-0,356	0,030	0,319
ACSS2	-0,356	0,068	0,436
GLRX	-0,356	0,052	0,389
PPIC	-0,357	0,008	0,182
STARD13	-0,357	0,005	0,152
ZGRF1	-0,357	0,075	0,452
ATP5MC2	-0,357	0,048	0,380

CCDC71L	-0,358	0,292	0,703
EI24	-0,358	0,007	0,171
C1orf43	-0,358	0,007	0,177
NCEH1	-0,358	0,085	0,472
STAMBPL1	-0,359	0,120	0,519
PSMB6	-0,359	0,031	0,320
MRPS23	-0,359	0,042	0,364
PHB	-0,359	0,043	0,365
PRDX3	-0,359	0,111	0,506
MPST	-0,359	0,088	0,476
PPRC1	-0,360	0,172	0,589
SLC35B1	-0,360	0,013	0,228
ECSIT	-0,361	0,134	0,540
MFSD6	-0,361	0,042	0,364
DLC1	-0,361	0,030	0,319
CBWD3	-0,361	0,009	0,193
LRRTM3	-0,361	0,252	0,666
SESN1	-0,362	0,036	0,340
THBD	-0,362	0,134	0,540
CREM	-0,362	0,164	0,580
CALU	-0,362	0,138	0,546
DHX32	-0,362	0,163	0,578
FDX1	-0,363	0,016	0,244
NDUFB8	-0,363	0,054	0,394
SNRPG	-0,364	0,062	0,420
UBE2L3	-0,364	0,007	0,171
TAF9	-0,364	0,143	0,551
MLX	-0,364	0,033	0,330
ADAM15	-0,364	0,141	0,550
COG5	-0,365	0,033	0,330
SMIM20	-0,365	0,071	0,443
HAX1	-0,365	0,029	0,311
B3GALNT1	-0,365	0,197	0,621
LRRC37A	-0,365	0,139	0,546
SRP9	-0,366	0,151	0,563
ANGPTL1	-0,367	0,147	0,556
EFCAB2	-0,367	0,147	0,556
SRGN	-0,367	0,309	0,715
CCDC43	-0,367	0,106	0,499
RPUSD3	-0,368	0,032	0,328
MKKS	-0,368	0,005	0,150
ERC2	-0,368	0,164	0,579
ARV1	-0,370	0,075	0,451
EIF4EBP2	-0,370	0,021	0,282
GPATCH4	-0,371	0,039	0,352
DIRAS1	-0,372	0,130	0,534
ASF1A	-0,373	0,089	0,478
ASB8	-0,373	0,008	0,190
COL12A1	-0,374	0,150	0,561
METTL5	-0,374	0,083	0,468
ITGA6	-0,374	0,058	0,412
CCT5	-0,375	0,026	0,302
MRPS10	-0,375	0,087	0,475

PATJ	-0,375	0,070	0,442
C9orf72	-0,375	0,151	0,562
TMX4	-0,375	0,019	0,269
TPM2	-0,375	0,147	0,556
CCT8	-0,375	0,090	0,480
COMMD7	-0,376	0,029	0,315
ZNF438	-0,376	0,008	0,180
RNF149	-0,376	0,122	0,523
COMMD3	-0,376	0,075	0,452
ETFDH	-0,377	0,088	0,477
HEBP2	-0,377	0,057	0,406
NQO2	-0,377	0,037	0,346
LSM5	-0,378	0,099	0,489
ATP6V1D	-0,378	0,120	0,519
PRKG2	-0,378	0,391	0,769
CMSS1	-0,378	0,014	0,233
AMD1	-0,379	0,028	0,308
CHCHD10	-0,379	0,118	0,517
HIKESHI	-0,379	0,046	0,379
PMVK	-0,379	0,097	0,485
MRPL20	-0,379	0,032	0,328
DHRS7C	-0,379	0,244	0,660
DLD	-0,380	0,108	0,502
PSD3	-0,381	0,224	0,646
SURF1	-0,382	0,038	0,347
PGK1	-0,382	0,035	0,336
SMAD3	-0,382	0,110	0,505
FPGS	-0,382	0,096	0,484
MARC2	-0,383	0,004	0,133
DHRS3	-0,383	0,131	0,535
HMCES	-0,383	0,027	0,305
DDO	-0,384	0,112	0,508
CCT3	-0,384	0,017	0,253
MRPL22	-0,384	0,017	0,253
CMC1	-0,385	0,016	0,244
ARHGAP26	-0,385	0,061	0,417
LSM3	-0,385	0,140	0,549
GJA3	-0,385	0,205	0,625
DPH3	-0,385	0,049	0,383
SCOC	-0,387	0,208	0,629
PPP2R3A	-0,387	0,090	0,480
WBP4	-0,387	0,015	0,241
MTRES1	-0,387	0,023	0,290
EMCN	-0,388	0,125	0,527
SMIM30	-0,388	0,227	0,647
EIF2B3	-0,388	0,049	0,383
MPDU1	-0,389	0,028	0,309
TSPAN14	-0,389	0,080	0,463
CLDN12	-0,390	0,040	0,356
MTCH2	-0,390	0,006	0,167
VASP	-0,390	0,055	0,399
HTATIP2	-0,390	0,039	0,352
MRPS21	-0,391	0,032	0,329

LITAF	-0,391	0,064	0,427
ACP6	-0,391	0,059	0,414
ATP1A2	-0,391	0,151	0,562
TTN	-0,391	0,432	0,794
AP5M1	-0,391	0,101	0,492
SCML1	-0,391	0,011	0,210
THOC7	-0,392	0,072	0,446
AIFM1	-0,392	0,033	0,330
MAP1B	-0,392	0,079	0,460
CASQ2	-0,393	0,306	0,713
PFDN4	-0,393	0,127	0,529
TACO1	-0,393	0,030	0,318
CTSA	-0,393	0,010	0,206
ZDHHC9	-0,393	0,037	0,344
STIP1	-0,393	0,031	0,320
TRPT1	-0,393	0,047	0,379
ERGIC2	-0,393	0,155	0,568
GHITM	-0,394	0,114	0,512
GLO1	-0,395	0,080	0,461
PPP1R14B	-0,395	0,128	0,531
MRPL32	-0,395	0,034	0,333
MS4A6A	-0,395	0,153	0,565
SLC38A1	-0,396	0,224	0,646
HSBP1	-0,397	0,037	0,345
FLT1	-0,397	0,110	0,504
MRPS31	-0,398	0,033	0,331
GREB1L	-0,398	0,138	0,546
PLK2	-0,399	0,152	0,564
KIF21A	-0,399	0,098	0,488
RTRAF	-0,399	0,038	0,346
ICMT	-0,399	0,012	0,218
PCSK6	-0,399	0,100	0,489
ABCA1	-0,399	0,147	0,556
AAMDC	-0,400	0,023	0,289
HSPB1	-0,400	0,462	0,811
DHRS7B	-0,400	0,013	0,228
SCN5A	-0,400	0,211	0,632
TMEM176A	-0,400	0,408	0,780
CLIC4	-0,400	0,095	0,484
SNTA1	-0,400	0,122	0,523
EVA1C	-0,401	0,061	0,417
PIGA	-0,401	0,048	0,380
PITPNC1	-0,401	0,085	0,472
FILIP1L	-0,401	0,045	0,372
SNU13	-0,402	0,036	0,338
TMEM147	-0,403	0,034	0,333
ABCC5	-0,403	0,126	0,527
CSDC2	-0,404	0,134	0,540
MTRNR2L8	-0,404	0,534	0,849
TES	-0,404	0,028	0,309
VDAC2	-0,405	0,015	0,240
DNAJC27	-0,405	0,006	0,170
C1QTNF1	-0,405	0,260	0,674

TSPAN13	-0,405	0,033	0,330
ITGA11	-0,406	0,091	0,481
COX4I1	-0,406	0,046	0,378
ADGRF5	-0,406	0,007	0,176
MTX2	-0,406	0,008	0,186
MMADHC	-0,407	0,123	0,525
CUTC	-0,407	0,016	0,244
GTPBP8	-0,408	0,022	0,284
TMX2	-0,408	0,001	0,078
ELN	-0,408	0,292	0,703
SFRP1	-0,408	0,158	0,571
GNG11	-0,409	0,054	0,394
LAMTOR5	-0,409	0,012	0,221
ZNF22	-0,409	0,090	0,481
RNF5	-0,409	0,007	0,171
NDUFA9	-0,409	0,062	0,418
MALL	-0,409	0,069	0,441
HAT1	-0,409	0,035	0,336
EMP3	-0,409	0,096	0,485
NDUFV3	-0,409	0,010	0,207
TRIM63	-0,409	0,101	0,491
ZNF189	-0,410	0,047	0,379
IDH3B	-0,410	0,022	0,284
ADGRL4	-0,410	0,066	0,432
CALCRL	-0,410	0,269	0,682
PTER	-0,410	0,045	0,375
TRAPPC13	-0,410	0,100	0,489
CAV1	-0,410	0,153	0,565
PPP1R14C	-0,410	0,070	0,442
UGP2	-0,411	0,095	0,484
CFD	-0,411	0,286	0,699
FXN	-0,412	0,036	0,340
TIMM8B	-0,412	0,060	0,415
RPAP3	-0,412	0,023	0,287
SEMA6A	-0,413	0,048	0,380
PSMB7	-0,413	0,005	0,150
ATP5F1A	-0,414	0,091	0,481
ALAS1	-0,414	0,039	0,352
RBIS	-0,414	0,043	0,368
ZFHX3	-0,414	0,144	0,554
DYNC1I1	-0,415	0,073	0,448
MT-ND3	-0,415	0,181	0,602
ADAMTS1	-0,415	0,161	0,577
RGS5	-0,415	0,104	0,495
QSOX1	-0,416	0,110	0,505
GTF2F2	-0,416	0,039	0,352
SIAE	-0,416	0,017	0,253
OXSM	-0,416	0,033	0,330
PLCB4	-0,416	0,161	0,577
JMJD6	-0,417	0,060	0,416
PSMC6	-0,417	0,121	0,521
PDE10A	-0,417	0,222	0,644
IFI16	-0,418	0,072	0,446

DUSP22	-0,418	0,002	0,107
FAHD1	-0,418	0,015	0,240
POLR2J3	-0,418	0,217	0,638
DCTN6	-0,418	0,141	0,551
KITLG	-0,419	0,212	0,634
TEAD2	-0,419	0,034	0,332
RPF2	-0,419	0,031	0,322
PPA2	-0,420	0,073	0,450
LRRC37A3	-0,420	0,096	0,484
MERTK	-0,422	0,152	0,564
PHACTR1	-0,422	0,208	0,629
SDHD	-0,423	0,133	0,539
ASNSD1	-0,423	0,052	0,389
LRRC49	-0,423	0,199	0,623
ADSSL1	-0,423	0,043	0,369
ACOX3	-0,423	0,093	0,483
HACD1	-0,424	0,085	0,472
TTC1	-0,424	0,026	0,297
RWDD1	-0,424	0,025	0,295
DNAJC21	-0,424	0,003	0,113
HSPE1	-0,424	0,047	0,380
CCDC69	-0,424	0,017	0,251
COPS3	-0,424	0,012	0,219
SELENOK	-0,425	0,102	0,492
SLC25A12	-0,426	0,065	0,431
NDUFS3	-0,426	0,017	0,251
AVEN	-0,426	0,016	0,250
SNRPC	-0,427	0,012	0,222
LAP3	-0,427	0,008	0,180
MALSU1	-0,427	0,005	0,154
FH	-0,428	0,054	0,395
ATP6V1E1	-0,428	0,031	0,322
LDLR	-0,429	0,247	0,661
GLRX5	-0,429	0,025	0,295
UQCRC2	-0,429	0,087	0,475
NDUFC2	-0,429	0,010	0,207
HK2	-0,431	0,207	0,629
IQGAP2	-0,431	0,143	0,551
CASTOR2	-0,431	0,240	0,658
LMOD3	-0,432	0,238	0,656
MRPL34	-0,432	0,033	0,330
SRPX	-0,432	0,025	0,295
TMEM176B	-0,433	0,301	0,711
MRPL37	-0,434	0,007	0,174
CLTCL1	-0,434	0,051	0,388
PTGES2	-0,434	0,073	0,448
CYC1	-0,434	0,114	0,512
NDUFS2	-0,435	0,020	0,274
MICU2	-0,435	0,012	0,223
TINAGL1	-0,435	0,044	0,370
FAR2	-0,435	0,007	0,172
ADCY1	-0,435	0,126	0,527
COX7A2	-0,435	0,065	0,430

MFN1	-0,436	0,056	0,403
SLC2A1	-0,437	0,064	0,427
PAG1	-0,437	0,022	0,285
GLA	-0,437	0,027	0,303
GIMAP7	-0,437	0,030	0,319
AOX1	-0,438	0,441	0,797
CACYBP	-0,438	0,009	0,191
CIAO2A	-0,439	0,028	0,309
MMP15	-0,439	0,186	0,609
PPIF	-0,439	0,046	0,377
TPM3	-0,440	0,146	0,556
HINT1	-0,440	0,007	0,176
MRPS5	-0,440	0,003	0,115
MDH2	-0,441	0,037	0,346
TMEM182	-0,441	0,366	0,755
PALMD	-0,441	0,011	0,217
PERM1	-0,442	0,146	0,556
COA8	-0,443	0,026	0,300
LRRC40	-0,443	0,100	0,489
SRL	-0,443	0,163	0,578
PDHX	-0,444	0,050	0,386
TIMM23	-0,444	0,003	0,114
FCGR2A	-0,444	0,090	0,481
PLPP3	-0,444	0,001	0,090
PEBP4	-0,445	0,156	0,568
NDUFAF5	-0,445	0,016	0,244
PSMC2	-0,445	0,052	0,389
CKB	-0,445	0,210	0,632
SPTSSA	-0,446	0,008	0,180
KLHL31	-0,446	0,197	0,621
PLCL2	-0,446	0,068	0,437
ZNF449	-0,446	0,048	0,380
GPX3	-0,447	0,062	0,422
CEP83	-0,447	0,059	0,415
MRPL50	-0,447	0,091	0,481
FAM160A1	-0,447	0,044	0,371
HMGN3	-0,448	0,039	0,350
PRADC1	-0,448	0,032	0,326
GFRA1	-0,449	0,158	0,571
ASB15	-0,449	0,307	0,714
MRPL14	-0,449	0,031	0,320
GSTM4	-0,450	0,013	0,232
MRPL48	-0,450	0,016	0,249
PTBP2	-0,450	0,013	0,232
APCDD1	-0,450	0,150	0,560
ILRUN	-0,451	0,016	0,251
MLIP	-0,452	0,143	0,551
PUDP	-0,453	0,011	0,216
RBPMS2	-0,455	0,109	0,503
MRPL58	-0,455	0,008	0,187
MRPS18C	-0,455	0,020	0,274
NFU1	-0,457	0,022	0,284
CWC15	-0,457	0,033	0,330

COPS4	-0,458	0,096	0,484
UBA3	-0,458	0,072	0,446
ZADH2	-0,458	0,017	0,253
APIP	-0,459	0,010	0,206
MSRA	-0,460	0,007	0,171
RWDD2B	-0,460	0,016	0,250
TRMT10C	-0,460	0,063	0,422
MAP3K6	-0,461	0,090	0,480
KCNK1	-0,461	0,041	0,359
GJA5	-0,461	0,222	0,644
WARS	-0,461	0,017	0,251
CHCHD1	-0,462	0,006	0,167
FARS2	-0,462	0,009	0,197
TUBB2A	-0,462	0,102	0,493
RASGRF2	-0,462	0,020	0,274
KCTD9	-0,463	0,011	0,215
MTHFR	-0,463	0,076	0,453
ME2	-0,464	0,009	0,193
FASTKD5	-0,465	0,003	0,114
ELOC	-0,465	0,012	0,221
IL17RA	-0,465	0,079	0,460
MRPS15	-0,465	0,002	0,105
MRPS9	-0,466	0,010	0,201
STOX2	-0,466	0,001	0,073
IP6K3	-0,467	0,216	0,637
MRPS7	-0,467	0,018	0,256
CTTNBP2	-0,467	0,049	0,382
LDHA	-0,468	0,017	0,254
HJV	-0,468	0,107	0,501
SLC36A4	-0,468	0,005	0,152
LARP6	-0,468	0,014	0,235
PRXL2A	-0,469	0,025	0,295
ATP5MG	-0,469	0,091	0,481
GMPR	-0,469	0,048	0,380
WDYHV1	-0,470	0,048	0,380
EGFR	-0,470	0,131	0,535
SEC61G	-0,470	0,096	0,484
HCCS	-0,471	0,009	0,197
FAM20A	-0,472	0,041	0,361
SLC25A3	-0,472	0,012	0,218
ATP1B1	-0,473	0,014	0,234
BPGM	-0,474	0,068	0,436
UQCR11	-0,474	0,063	0,425
C1orf115	-0,474	0,039	0,351
RRP15	-0,476	0,005	0,144
ACACA	-0,476	0,068	0,437
NUDT19	-0,478	0,013	0,232
NUDT12	-0,478	0,075	0,452
MTLN	-0,478	0,058	0,409
CACNB2	-0,478	0,042	0,364
SUCLG1	-0,479	0,010	0,202
ANK3	-0,479	0,024	0,290
BZW2	-0,481	0,076	0,454

NOP10	-0,481	0,025	0,295
SPOCK1	-0,481	0,053	0,391
COX7A1	-0,482	0,088	0,477
RDH14	-0,483	0,011	0,216
PDE7A	-0,483	0,118	0,517
UQCRC1	-0,483	0,041	0,361
FAM162A	-0,483	0,020	0,273
CADPS	-0,483	0,238	0,656
SLC43A3	-0,484	0,033	0,330
PRELID2	-0,485	0,031	0,322
CALCOCO2	-0,486	0,000	0,040
NAMPT	-0,486	0,212	0,633
IGFBP4	-0,486	0,188	0,613
C3orf18	-0,486	0,015	0,242
PSMD14	-0,487	0,037	0,345
TPI1	-0,488	0,021	0,283
KLHL7	-0,488	0,061	0,418
CAST	-0,488	0,033	0,330
PTRH2	-0,489	0,007	0,180
MRPL21	-0,489	0,013	0,228
CKMT2	-0,490	0,076	0,452
GPC6	-0,490	0,134	0,540
TTC39A	-0,490	0,048	0,380
RAMP1	-0,490	0,108	0,502
PDHB	-0,491	0,030	0,318
CYYR1	-0,491	0,011	0,210
PSMB3	-0,491	0,009	0,192
MPP1	-0,491	0,002	0,104
KIAA1217	-0,495	0,049	0,383
NCOA4	-0,495	0,052	0,388
ASB11	-0,496	0,245	0,661
TMEM126B	-0,496	0,044	0,370
SOD1	-0,497	0,027	0,303
PCDH1	-0,499	0,097	0,485
COX5B	-0,499	0,048	0,380
LPAR3	-0,499	0,043	0,368
WDR12	-0,500	0,001	0,073
IDH3A	-0,500	0,106	0,500
SREBF1	-0,501	0,131	0,536
MTRF1L	-0,502	0,001	0,056
RCAN2	-0,504	0,010	0,207
TIMMDC1	-0,505	0,016	0,244
PTPRG	-0,505	0,010	0,205
ARL17A	-0,505	0,093	0,483
GDF11	-0,505	0,027	0,305
AASS	-0,506	0,009	0,195
ENPP4	-0,507	0,123	0,525
TUBB6	-0,507	0,005	0,144
ATP5MF	-0,507	0,011	0,213
SIRT2	-0,507	0,022	0,284
ACOT13	-0,508	0,001	0,084
DTNA	-0,508	0,025	0,295
SDCBP	-0,508	0,020	0,275

NDUFS6	-0,508	0,029	0,313
CAPZA2	-0,509	0,141	0,550
LRRC20	-0,509	0,070	0,441
DNAJC19	-0,510	0,023	0,285
ANXA3	-0,511	0,038	0,348
HADHB	-0,512	0,038	0,350
ARL17B	-0,513	0,075	0,452
TRIM69	-0,513	0,002	0,111
DLAT	-0,513	0,039	0,351
SSBP1	-0,513	0,008	0,189
AP1S2	-0,514	0,051	0,388
TEK	-0,514	0,001	0,090
NDUFA8	-0,514	0,019	0,269
RIMKLA	-0,514	0,092	0,482
SHISA4	-0,514	0,035	0,336
MRPL15	-0,515	0,001	0,085
PLSCR1	-0,516	0,024	0,291
SUCLA2	-0,519	0,057	0,405
RAB27A	-0,519	0,047	0,379
SH3KBP1	-0,520	0,003	0,114
NDUFAF4	-0,520	0,077	0,456
NPAS2	-0,521	0,067	0,434
FUNDC2	-0,521	0,004	0,143
MRPL13	-0,523	0,025	0,295
C2orf88	-0,524	0,004	0,136
PSMA3	-0,524	0,001	0,087
ATG4A	-0,524	0,030	0,318
PSMD7	-0,524	0,004	0,143
CDC42EP4	-0,525	0,049	0,383
NDUFS4	-0,527	0,049	0,382
MPC2	-0,527	0,013	0,232
MCUB	-0,528	0,029	0,313
DECR1	-0,530	0,010	0,207
HDDC2	-0,530	0,003	0,123
NDUFB11	-0,531	0,015	0,235
COPS5	-0,531	0,006	0,164
ATAD1	-0,531	0,081	0,465
TM4SF18	-0,531	0,086	0,474
ITGAM	-0,532	0,041	0,361
FASTKD1	-0,532	0,081	0,464
TMEM70	-0,532	0,014	0,233
VSTM2L	-0,532	0,105	0,497
UBE2F	-0,534	0,003	0,114
PRRX1	-0,534	0,012	0,223
COQ8A	-0,534	0,029	0,311
UGCG	-0,535	0,099	0,489
YARS	-0,536	0,008	0,187
CTSB	-0,536	0,007	0,180
CHCHD4	-0,536	0,002	0,094
FITM1	-0,536	0,085	0,472
PCMT1	-0,536	0,020	0,274
UQCRHL	-0,536	0,039	0,350
MRPL51	-0,537	0,006	0,161

RBFOX1	-0,538	0,076	0,454
PIGK	-0,538	0,078	0,458
MYL7	-0,540	0,170	0,588
PARVB	-0,540	0,031	0,322
B4GALT6	-0,540	0,007	0,174
PDCD5	-0,541	0,009	0,192
RNASE1	-0,541	0,016	0,244
IER3IP1	-0,541	0,055	0,399
VBP1	-0,541	0,057	0,406
COX8A	-0,541	0,021	0,281
PKDCC	-0,541	0,028	0,309
VDAC3	-0,542	0,043	0,365
H2AFZ	-0,543	0,010	0,203
GNB5	-0,543	0,002	0,113
SLCO2A1	-0,543	0,111	0,506
GFPT2	-0,544	0,306	0,713
SNRNP25	-0,544	0,009	0,195
CEP85L	-0,544	0,011	0,209
USP2	-0,544	0,093	0,483
PGAP1	-0,545	0,082	0,468
PLEK	-0,545	0,074	0,450
TMEM273	-0,545	0,001	0,073
TMEM116	-0,545	0,022	0,285
EMC3	-0,546	0,006	0,171
HRC	-0,547	0,060	0,416
COX14	-0,548	0,003	0,113
TUBB4B	-0,548	0,066	0,432
MDH1	-0,548	0,070	0,441
GOT1	-0,549	0,057	0,407
KPNA2	-0,549	0,004	0,143
CBLB	-0,549	0,008	0,185
CD14	-0,550	0,170	0,588
NIPSNAP3B	-0,550	0,022	0,285
CLMN	-0,551	0,120	0,521
FBLIM1	-0,552	0,016	0,244
AIF1L	-0,553	0,002	0,098
NDUFAF1	-0,554	0,014	0,234
FCGR2B	-0,555	0,023	0,289
PDHA1	-0,555	0,007	0,176
MRVI1	-0,556	0,017	0,253
NDUFB4	-0,556	0,005	0,151
PPA1	-0,558	0,020	0,273
VWC2	-0,558	0,075	0,452
FGF12	-0,558	0,254	0,666
ETFRF1	-0,559	0,067	0,434
PRDX6	-0,559	0,003	0,128
GTF3C6	-0,560	0,003	0,113
DBN1	-0,561	0,048	0,380
TRDN	-0,561	0,084	0,472
RANBP6	-0,561	0,067	0,434
MRPL47	-0,562	0,030	0,318
PLEKHG1	-0,562	0,026	0,298
MTIF2	-0,563	0,022	0,285

SERPINB6	-0,563	0,001	0,073
PPL	-0,563	0,138	0,546
MRPS22	-0,563	0,022	0,284
MEOX1	-0,564	0,040	0,352
QRSL1	-0,564	0,005	0,148
MRPL27	-0,564	0,001	0,061
SYNM	-0,565	0,029	0,315
GRK3	-0,566	0,053	0,391
MYZAP	-0,566	0,045	0,372
BHMT2	-0,566	0,012	0,218
SLCO5A1	-0,567	0,093	0,484
ATP5ME	-0,568	0,021	0,282
RALGPS2	-0,568	0,001	0,090
PLXDC1	-0,569	0,050	0,383
SOCS3	-0,570	0,157	0,569
TBC1D1	-0,572	0,001	0,061
NDUFC1	-0,572	0,006	0,167
ATP5PD	-0,574	0,048	0,380
ABRA	-0,575	0,125	0,527
ACLY	-0,576	0,023	0,285
MOB4	-0,576	0,055	0,401
ADK	-0,577	0,001	0,084
SLC2A4	-0,578	0,063	0,425
PMP22	-0,578	0,001	0,073
SMIM12	-0,579	0,000	0,020
C8orf88	-0,579	0,052	0,389
MICOS10	-0,579	0,000	0,038
CSRP2	-0,579	0,031	0,322
NFIL3	-0,580	0,042	0,364
KLHL23	-0,581	0,010	0,205
MRPL39	-0,581	0,018	0,256
AGTPBP1	-0,582	0,044	0,371
IMPA1	-0,583	0,089	0,479
TMEM67	-0,583	0,028	0,309
NDUFB2	-0,584	0,005	0,156
NDUFB10	-0,584	0,005	0,147
MTHFD2	-0,585	0,003	0,123
TPRKB	-0,585	0,011	0,216
TRNP1	-0,585	0,009	0,200
TGFBI	-0,587	0,009	0,192
SELENOW	-0,587	0,007	0,177
CKM	-0,588	0,049	0,380
HSDL1	-0,588	0,001	0,064
CPM	-0,589	0,280	0,692
AKTIP	-0,590	0,000	0,039
PPTC7	-0,592	0,023	0,290
PLN	-0,592	0,303	0,712
COX5A	-0,592	0,011	0,216
ATP5F1E	-0,592	0,024	0,292
TBC1D7	-0,593	0,002	0,094
ATP5MC3	-0,594	0,002	0,097
TMTC1	-0,594	0,003	0,123
ACSM3	-0,594	0,108	0,502

NCALD	-0,594	0,008	0,180
MRPL1	-0,595	0,044	0,370
WASHC2A	-0,596	0,030	0,316
APOBEC2	-0,596	0,031	0,321
GALNT16	-0,598	0,053	0,391
UQCRFS1	-0,600	0,004	0,138
IGFBP6	-0,600	0,200	0,624
LMOD1	-0,600	0,080	0,463
ABCG1	-0,601	0,048	0,380
TCN2	-0,603	0,045	0,372
GTF3A	-0,605	0,000	0,013
FKBP3	-0,607	0,001	0,080
UQCR10	-0,608	0,027	0,303
COX7C	-0,611	0,012	0,221
PTP4A3	-0,611	0,081	0,464
ABCA9	-0,611	0,015	0,242
HMOX2	-0,612	0,001	0,064
SLC25A4	-0,613	0,019	0,271
TOMM40L	-0,613	0,004	0,138
FADS2	-0,617	0,061	0,418
KIAA0040	-0,618	0,016	0,244
ASB2	-0,620	0,041	0,361
SMYD1	-0,622	0,032	0,327
ADAMTS15	-0,624	0,091	0,481
ATP5PB	-0,625	0,017	0,253
VWDE	-0,625	0,079	0,460
TIMM21	-0,626	0,005	0,158
MAMDC2	-0,627	0,021	0,281
COX6B1	-0,627	0,007	0,174
SLC39A14	-0,629	0,008	0,180
POMP	-0,629	0,011	0,215
KCNK3	-0,629	0,011	0,217
FPGT-TNNI3K	-0,631	0,235	0,655
UTP11	-0,631	0,003	0,116
C11orf74	-0,633	0,029	0,311
HYAL1	-0,634	0,010	0,207
CD59	-0,634	0,000	0,039
RPL3L	-0,635	0,046	0,377
ART3	-0,637	0,051	0,388
RAB15	-0,642	0,038	0,350
ARL3	-0,642	0,001	0,073
MRPS33	-0,643	0,004	0,143
LRRC39	-0,643	0,109	0,503
HSPB3	-0,645	0,036	0,340
SDHB	-0,646	0,004	0,143
POPDC3	-0,646	0,013	0,228
CHCHD3	-0,646	0,001	0,056
ALPK2	-0,647	0,075	0,452
PPP1R3C	-0,647	0,030	0,318
MT2A	-0,647	0,223	0,646
EDA	-0,648	0,006	0,171
DNAJC15	-0,648	0,016	0,244
NDUFB9	-0,649	0,001	0,076

SYT7	-0,649	0,027	0,305
RBM38	-0,649	0,057	0,407
MCTP1	-0,650	0,032	0,329
RAD1	-0,652	0,000	0,015
NDUFA6	-0,653	0,007	0,176
ATP1B3	-0,655	0,015	0,235
CISD1	-0,655	0,025	0,295
DIPK2B	-0,655	0,003	0,115
CHPT1	-0,657	0,002	0,108
ZNF385D	-0,658	0,027	0,305
STXBP6	-0,658	0,005	0,153
NRXN1	-0,659	0,034	0,333
SLIRP	-0,659	0,011	0,215
RTN4IP1	-0,661	0,004	0,133
TCAP	-0,663	0,090	0,481
RNF144B	-0,664	0,002	0,107
SLC25A26	-0,664	0,000	0,029
APOO	-0,665	0,001	0,090
ATP5PF	-0,667	0,003	0,128
ALDOC	-0,668	0,007	0,171
PDGFRL	-0,668	0,005	0,144
CTSL	-0,670	0,000	0,034
FAM155B	-0,670	0,025	0,295
AMPH	-0,672	0,014	0,233
OSMR	-0,673	0,022	0,285
ACKR3	-0,674	0,012	0,223
KCNJ5	-0,675	0,079	0,460
MRPS6	-0,676	0,027	0,305
GFOD1	-0,677	0,007	0,171
TPD52L1	-0,680	0,002	0,107
TUBA4A	-0,684	0,066	0,432
PIP5K1B	-0,685	0,011	0,215
MTERF3	-0,688	0,000	0,029
ATP5MD	-0,691	0,032	0,327
BDH1	-0,692	0,035	0,336
EMILIN2	-0,692	0,019	0,266
UQCRH	-0,692	0,012	0,218
RAB3IP	-0,693	0,017	0,251
NDUFB1	-0,696	0,005	0,156
COQ3	-0,696	0,004	0,136
PLGRKT	-0,696	0,006	0,165
EPB41L3	-0,696	0,008	0,180
MRPL33	-0,697	0,003	0,113
PVR	-0,699	0,001	0,080
TECRL	-0,700	0,129	0,534
ADCY3	-0,702	0,026	0,297
MEIS1	-0,703	0,001	0,080
DAPK2	-0,703	0,010	0,205
TMEM126A	-0,703	0,010	0,208
SAMHD1	-0,703	0,024	0,291
ULK4	-0,705	0,000	0,042
RASL12	-0,710	0,005	0,145
CMC2	-0,710	0,002	0,105

FNDC5	-0,712	0,029	0,315
NDUFS5	-0,712	0,009	0,193
EDNRB	-0,712	0,036	0,341
RSRC1	-0,714	0,000	0,041
COQ10A	-0,715	0,007	0,178
F5	-0,716	0,136	0,544
ABCA10	-0,717	0,018	0,264
PDE4D	-0,719	0,009	0,199
MRPS36	-0,719	0,022	0,285
LDHB	-0,723	0,078	0,459
GATB	-0,724	0,004	0,136
CADM2	-0,725	0,043	0,366
PCNT	-0,726	0,019	0,266
ATP5MPL	-0,727	0,004	0,141
FAM216B	-0,731	0,056	0,402
EGFLAM	-0,731	0,004	0,141
ATP5PO	-0,735	0,004	0,133
SPARCL1	-0,737	0,002	0,111
S100A8	-0,738	0,224	0,646
ACTC1	-0,743	0,022	0,285
CYCS	-0,745	0,047	0,379
DESI1	-0,748	0,001	0,090
MYOZ2	-0,748	0,063	0,425
F8	-0,756	0,014	0,233
KCNJ3	-0,759	0,015	0,240
MYO5C	-0,766	0,003	0,123
CLGN	-0,767	0,085	0,473
ATP5MC1	-0,770	0,002	0,100
ATP5F1C	-0,770	0,005	0,153
EBF2	-0,772	0,002	0,091
MTIF3	-0,774	0,000	0,020
PPM1L	-0,781	0,000	0,034
CAMK2A	-0,783	0,007	0,171
PRDX1	-0,788	0,002	0,094
COX7B	-0,788	0,003	0,113
C11orf21	-0,792	0,006	0,171
GPSM2	-0,792	0,002	0,095
SLA	-0,792	0,036	0,337
NDUFA4	-0,793	0,007	0,173
SLC5A1	-0,798	0,055	0,397
SIGLEC1	-0,804	0,006	0,171
SKAP2	-0,806	0,000	0,017
HSF2	-0,807	0,001	0,076
HRH2	-0,811	0,013	0,232
NDUFA1	-0,812	0,004	0,133
MRPS25	-0,813	0,000	0,029
SPSB1	-0,813	0,002	0,107
ENPEP	-0,815	0,001	0,090
HIGD1A	-0,815	0,043	0,368
ITGB1BP2	-0,816	0,008	0,189
SRPX2	-0,819	0,044	0,370
PPP1R1A	-0,820	0,020	0,277
NDUFB6	-0,821	0,003	0,123

SH3PXD2B	-0,825	0,020	0,278
CSRP3	-0,830	0,024	0,292
FGF7	-0,830	0,022	0,285
TMEM38B	-0,834	0,012	0,225
PIM1	-0,834	0,026	0,300
BCL6	-0,834	0,005	0,153
GPT2	-0,835	0,048	0,380
MLF1	-0,839	0,015	0,241
ME1	-0,840	0,000	0,013
PPP1R3A	-0,842	0,105	0,497
S100A9	-0,845	0,139	0,546
UAP1	-0,846	0,009	0,196
MRC1	-0,847	0,007	0,173
AK1	-0,849	0,011	0,215
STEAP4	-0,850	0,002	0,107
FCER1G	-0,854	0,005	0,157
MYH6	-0,857	0,096	0,484
RGS6	-0,860	0,001	0,078
TIMM17A	-0,860	0,000	0,049
ATP2A2	-0,868	0,008	0,189
IDH2	-0,869	0,002	0,110
RD3L	-0,869	0,047	0,380
SCARA5	-0,870	0,017	0,251
COX17	-0,871	0,000	0,047
TRMT9B	-0,872	0,008	0,184
PPP1CC	-0,873	0,000	0,002
AACS	-0,873	0,005	0,144
TOGARAM2	-0,874	0,004	0,138
MPP3	-0,881	0,014	0,233
VTN	-0,886	0,041	0,361
TBX3	-0,887	0,000	0,047
NT5DC2	-0,889	0,009	0,196
CASQ1	-0,892	0,089	0,479
ISOC1	-0,893	0,000	0,034
NDUFB3	-0,898	0,006	0,167
UQCRB	-0,905	0,007	0,177
COX6C	-0,906	0,005	0,152
SMPX	-0,908	0,013	0,232
B3GALT2	-0,910	0,040	0,357
FASN	-0,910	0,184	0,606
SCN9A	-0,913	0,004	0,140
GLUL	-0,916	0,006	0,167
PPIL1	-0,922	0,000	0,023
SMCO1	-0,933	0,014	0,234
C6	-0,938	0,002	0,109
PYGM	-0,939	0,001	0,076
FKBP5	-0,942	0,060	0,416
LRRC10	-0,943	0,005	0,153
MS4A4A	-0,949	0,001	0,090
INSYN1	-0,950	0,006	0,166
NDUFAB1	-0,952	0,000	0,047
CNTNAP3	-0,954	0,034	0,332
NDUFA12	-0,960	0,001	0,083

NID1	-0,961	0,003	0,113
PITX2	-0,965	0,001	0,064
EGF	-0,968	0,009	0,197
DEPP1	-0,968	0,011	0,213
UGT2B4	-0,981	0,032	0,328
ADAMTSL4	-0,982	0,011	0,210
RAP1GAP2	-0,983	0,004	0,141
PLP1	-0,983	0,001	0,074
NDUFA5	-0,995	0,021	0,281
ACKR1	-0,995	0,017	0,254
CADPS2	-1,007	0,000	0,032
LILRB5	-1,008	0,001	0,068
FAM107A	-1,010	0,004	0,136
MYBPHL	-1,022	0,000	0,047
ENO3	-1,030	0,006	0,171
CNTNAP3B	-1,036	0,026	0,298
HAS2	-1,042	0,021	0,281
GCOM1	-1,049	0,006	0,171
PLTP	-1,050	0,001	0,078
DCLK1	-1,057	0,004	0,137
ARNTL	-1,058	0,000	0,009
HTR4	-1,058	0,000	0,009
KCNIP2	-1,059	0,002	0,092
LRMDA	-1,071	0,000	0,009
CMTM5	-1,082	0,003	0,128
FABP5	-1,086	0,000	0,028
TNFRSF19	-1,094	0,000	0,022
OSR1	-1,099	0,000	0,029
GABRB3	-1,099	0,002	0,092
PTGFR	-1,104	0,003	0,113
CILP	-1,111	0,005	0,144
HMOX1	-1,116	0,028	0,308
ACTA2	-1,120	0,002	0,111
TGFBR3	-1,128	0,000	0,035
STC2	-1,131	0,000	0,038
MYOC	-1,145	0,006	0,159
PTDSS1	-1,150	0,000	0,008
PLAU	-1,174	0,004	0,134
LRRC7	-1,181	0,004	0,141
REPS2	-1,186	0,001	0,060
SLC1A3	-1,187	0,001	0,073
MYL4	-1,188	0,004	0,141
CHL1	-1,189	0,000	0,047
GALNT15	-1,192	0,015	0,239
VIT	-1,213	0,003	0,123
RETREG1	-1,226	0,006	0,161
KLHL41	-1,238	0,015	0,243
THBS1	-1,264	0,012	0,217
CD209	-1,266	0,000	0,025
KCNN2	-1,273	0,000	0,013
S1PR3	-1,282	0,000	0,009
LRP1B	-1,288	0,005	0,150
ADAMTS9	-1,291	0,016	0,247

LYVE1	-1,356	0,000	0,025
SLC27A6	-1,424	0,000	0,009
AKR1B1	-1,434	0,000	0,039
VSIG4	-1,445	0,000	0,029
FBN2	-1,474	0,001	0,090
SELE	-1,526	0,031	0,320
MFAP5	-1,544	0,001	0,090
ADAMTS4	-1,564	0,000	0,039
CR1	-1,587	0,001	0,065
DHRS9	-1,617	0,001	0,076
APOD	-1,629	0,000	0,013
METTL7B	-1,657	0,001	0,073
F13A1	-1,673	0,000	0,002
SNX22	-1,691	0,000	0,041
LRRN3	-1,831	0,000	0,039
CRISPLD2	-2,019	0,000	0,003
NFXL1	-2,033	0,000	0,039
CD163	-2,147	0,000	0,039
**Up-regulated**			
TMSB4XP8	0,642	0,047	0,379
TMSB4XP4	0,608	0,131	0,536
TMSB4XP1	0,567	0,074	0,450
AL162151.2	0,528	0,112	0,507
EEF1A1P19	0,501	0,051	0,388
RPL3P4	0,496	0,156	0,568
EEF1A1P5	0,446	0,108	0,502
EEF1A1P6	0,442	0,102	0,492
EEF1A1P4	0,436	0,106	0,500
EEF1A1P11	0,436	0,137	0,545
EEF1A1P13	0,435	0,123	0,525
RPL13P12	0,432	0,063	0,425
EEF1A1P14	0,417	0,202	0,624
RPL15P2	0,410	0,065	0,429
RPS28P7	0,406	0,099	0,489
RPL15P3	0,391	0,065	0,430
HNRNPA1P10	0,388	0,032	0,329
RPL10P16	0,386	0,086	0,474
EEF1A1P12	0,383	0,211	0,632
RPS3AP47	0,375	0,134	0,540
EEF1A1P8	0,369	0,216	0,637
HNRNPA1P7	0,367	0,060	0,415
RPL7AP66	0,362	0,040	0,358
AC097523.1	0,362	0,104	0,495
RPL7AP6	0,355	0,078	0,459
AC005912.1	0,351	0,122	0,525
EEF1A1P25	0,339	0,210	0,631
EEF1A1P9	0,339	0,295	0,706
AC099560.2	0,334	0,163	0,578
EIF4BP3	0,329	0,083	0,468
AP000936.3	0,328	0,213	0,634
EIF4BP6	0,318	0,100	0,489
RPS3AP5	0,310	0,244	0,660
TTC3P1	0,305	0,041	0,361

AC008026.1	0,304	0,114	0,511
EIF3FP3	0,303	0,094	0,484
AC010468.1	0,300	0,235	0,655
EIF4BP7	0,295	0,101	0,490
RPS3AP25	0,292	0,354	0,745
RPL18AP3	0,290	0,201	0,624
AC099336.2	0,290	0,223	0,646
RPL10P6	0,288	0,263	0,676
HNRNPA1P35	0,285	0,116	0,514
RPL10P9	0,282	0,202	0,624
EEF1A1P29	0,280	0,272	0,685
RPS15AP17	0,279	0,399	0,774
RPS3AP6	0,274	0,300	0,710
RPS10P3	0,272	0,154	0,566
AC010343.1	0,271	0,235	0,655
AL033519.3	0,262	0,434	0,795
RPS3AP26	0,262	0,326	0,725
AC008038.1	0,258	0,188	0,613
AL591135.1	0,257	0,123	0,525
RPL13AP25	0,255	0,235	0,655
RPL13AP5	0,250	0,271	0,684
ANXA2P2	0,246	0,398	0,774
RPL9P7	0,242	0,329	0,728
AC024293.1	0,240	0,276	0,688
AC020898.1	0,226	0,401	0,774
RPL12P4	0,224	0,349	0,743
AC133134.1	0,221	0,416	0,784
AL589880.1	0,206	0,308	0,715
RPL29P11	0,201	0,309	0,715
RPS23P8	0,200	0,443	0,798
AC079922.1	0,195	0,484	0,821
HSP90AB3P	0,189	0,198	0,622
BZW1P2	0,184	0,393	0,769
RPL39P3	0,179	0,441	0,797
RPL13AP20	0,178	0,475	0,818
AP001324.1	0,172	0,415	0,784
RPL7P19	0,164	0,567	0,863
AC110749.1	0,160	0,593	0,879
AL590867.2	0,149	0,365	0,754
RPSAP19	0,149	0,561	0,860
AC016596.3	0,147	0,494	0,826
RPL41P5	0,146	0,475	0,818
AC078817.1	0,135	0,481	0,820
RPL22P1	0,132	0,507	0,832
RPL4P5	0,132	0,550	0,856
RPSAP12	0,131	0,577	0,870
AC144530.1	0,128	0,653	0,899
NPM1P27	0,126	0,588	0,875
RPL41P1	0,122	0,569	0,865
RPS2P5	0,117	0,582	0,872
RPSAP58	0,116	0,654	0,899
RPL41P2	0,113	0,618	0,887
AC090498.1	0,111	0,600	0,882

**Processed pseudogene**	RPL23AP42	0,107	0,717	0,925
	RPL14P1	0,107	0,629	0,891
	RPS2P46	0,106	0,565	0,862
	AC073861.1	0,105	0,755	0,937
	RALGAPA1P1	0,096	0,477	0,819
	AC012085.1	0,093	0,585	0,872
	BX679664.3	0,092	0,721	0,927
	MTND4P12	0,090	0,729	0,930
	RPL4P4	0,088	0,676	0,909
	AC087343.1	0,087	0,734	0,931
	RPS26P47	0,079	0,812	0,954
	AC009487.2	0,075	0,548	0,855
	RPL26P19	0,074	0,790	0,948
	AC078819.1	0,069	0,680	0,911
	AC115223.1	0,062	0,816	0,954
	AC138392.1	0,052	0,767	0,941
	AC113935.1	0,050	0,851	0,969
	RPLP0P6	0,050	0,850	0,968
	AC009245.1	0,043	0,865	0,973
	AL450405.1	0,039	0,848	0,968
	RPS13P2	0,039	0,858	0,971
	RPS7P1	0,031	0,900	0,980
	RPL21P134	0,030	0,917	0,985
	BCLAF1P2	0,030	0,850	0,968
	AC064799.1	0,028	0,916	0,984
	AC116533.1	0,027	0,911	0,983
	RPL24P4	0,026	0,885	0,977
	AC104563.1	0,025	0,904	0,981
	FTH1P2	0,016	0,951	0,993
	AC107032.1	0,015	0,954	0,994
	ZNF322P1	0,013	0,942	0,992
	RPL7P32	0,008	0,980	0,998
	AC034236.1	0,007	0,977	0,998
	RPL21P93	0,007	0,978	0,998
	RPS2P55	0,005	0,981	0,998
	FTH1P7	0,001	0,996	1,000
	**Down-regulated**			
	RPSAP54	-0,004	0,984	0,998
	AC005000.1	-0,005	0,994	1,000
	AC092155.2	-0,005	0,986	0,999
	H3F3BP1	-0,006	1,000	1,000
	RPL7P1	-0,007	0,983	0,998
	HNRNPCP2	-0,008	0,958	0,995
	RPL7P9	-0,009	0,977	0,998
	AC106872.2	-0,010	0,969	0,997
	RPSAP15	-0,011	0,959	0,995
	HMGB1P10	-0,014	0,953	0,994
	RPS7P11	-0,016	0,943	0,993
	RPL21P28	-0,016	0,948	0,993
	AC114728.1	-0,018	0,939	0,992
	AC022149.1	-0,019	0,925	0,988
	HNRNPA3P6	-0,022	0,893	0,979
	AC093591.1	-0,023	0,920	0,986

TCEA1P2	-0,024	0,931	0,990
RPS26P15	-0,025	0,944	0,993
FTH1P5	-0,025	0,913	0,984
RPL7P23	-0,027	0,930	0,990
AC026403.1	-0,027	0,923	0,987
RPS24P8	-0,029	0,912	0,984
RPL21P75	-0,034	0,883	0,977
RPL21P16	-0,035	0,887	0,978
RPL21P39	-0,035	0,891	0,978
RPL21P119	-0,038	0,874	0,976
AC011979.1	-0,039	0,856	0,970
RPL21P11	-0,040	0,881	0,977
AL009174.1	-0,041	0,878	0,977
H3F3AP6	-0,047	0,778	0,945
HMGB1P6	-0,050	0,801	0,950
AL592114.1	-0,053	0,805	0,952
H3F3AP4	-0,061	0,624	0,889
FTH1P8	-0,077	0,749	0,936
AC004552.1	-0,077	0,831	0,962
NUDT4P2	-0,078	0,680	0,911
FTH1P20	-0,081	0,748	0,936
YBX1P1	-0,090	0,537	0,850
PPIAP29	-0,092	0,607	0,885
RPS26P8	-0,092	0,778	0,945
HSP90AA2P	-0,100	0,671	0,908
RPS2P7	-0,102	0,734	0,931
DYNC1I2P1	-0,103	0,606	0,885
YBX1P10	-0,107	0,504	0,832
SPCS2P4	-0,121	0,476	0,818
PPIAP22	-0,125	0,496	0,827
AC026271.1	-0,141	0,513	0,837
RPSAP9	-0,147	0,568	0,864
MORF4L1P1	-0,151	0,318	0,721
HSPD1P1	-0,161	0,372	0,756
RPS26P6	-0,166	0,614	0,887
TUBAP2	-0,186	0,272	0,685
EIF4EP2	-0,204	0,450	0,803
EIF2S2P4	-0,224	0,238	0,657
ZNF204P	-0,295	0,226	0,646
SRP9P1	-0,315	0,155	0,568
RHEBP2	-0,316	0,111	0,506
MTCO2P2	-0,332	0,316	0,720
RPSAP47	-0,336	0,217	0,638
MTCO1P40	-0,375	0,213	0,634
PSMC1P1	-0,390	0,103	0,495
AC007318.1	-0,402	0,014	0,234
VDAC1P1	-0,413	0,046	0,377
VDAC1P6	-0,441	0,036	0,343
TPI1P1	-0,444	0,028	0,309
NAMPTP1	-0,458	0,216	0,637
UQCRFS1P1	-0,527	0,006	0,166
AC112191.2	-0,778	0,019	0,266
CYCSP55	-0,854	0,042	0,362

	UQCRBP1	-0,907	0,013	0,232
	FABP5P7	-1,029	0,000	0,039
**Polymorphic pseudogene**	**Up-regulated**			
	PKD1L2	0,268	0,439	0,797
	**Down-regulated**			
	FCGR2C	-0,385	0,094	0,484
**Mt_tRNA**	**Up-regulated**			
	MT-RNR1	1,036	0,271	0,684
	MT-RNR2	0,481	0,252	0,666
	MT-TA	0,134	0,766	0,941
	MT-TN	0,127	0,744	0,934
	**Down-regulated**			
	MT-TC	-0,008	0,983	0,998
	MT-TY	-0,287	0,444	0,798
	MT-TP	-0,509	0,244	0,661
**miscRNA**	**Up-regulated**			
	RNY4	1,639	0,044	0,370
	RN7SKP255	0,969	0,165	0,582
	RNY1	0,911	0,148	0,557
	RNY3P1	0,879	0,114	0,512
	Y_RNA	0,873	0,119	0,518
	Y_RNA	0,869	0,122	0,522
	RNY3	0,867	0,124	0,527
	RN7SKP203	0,812	0,182	0,604
	Y_RNA	0,765	0,137	0,545
	Y_RNA	0,754	0,193	0,617
	Y_RNA	0,623	0,127	0,530
	RN7SL1	0,517	0,342	0,737
	RN7SL3	0,517	0,262	0,676
	RN7SL5P	0,500	0,291	0,702
	RN7SL4P	0,485	0,314	0,718
	RN7SKP71	0,478	0,361	0,750
	RN7SL674P	0,463	0,318	0,721
	RN7SL396P	0,441	0,245	0,661
	RN7SL128P	0,364	0,434	0,795
	7SK	0,310	0,350	0,743
	RN7SKP230	0,283	0,439	0,797
	RN7SKP80	0,269	0,491	0,824
**miRNA**	**Up-regulated**			
	MIR1244-2	0,342	0,317	0,720
	MIR3609	0,003	0,993	1,000
	**Up-regulated**			
	FP236383.4	2,659	0,051	0,387
	FP671120.6	2,558	0,080	0,463
	FP671120.7	2,290	0,134	0,540
	FP236383.5	2,146	0,213	0,634
	AC159540.2	0,792	0,004	0,133
	LINC02208	0,778	0,087	0,475
	AL078590.2	0,775	0,032	0,328
	AC020916.1	0,771	0,070	0,441
	AL138828.1	0,687	0,025	0,295
	AC092683.1	0,659	0,020	0,274

HELLPAR	0,593	0,309	0,715
SNHG29	0,558	0,009	0,197
GAS5	0,542	0,014	0,234
LINC00702	0,532	0,082	0,467
AC239809.3	0,480	0,035	0,336
NR2F1-AS1	0,441	0,059	0,415
CYTOR	0,433	0,179	0,599
AC104083.1	0,416	0,093	0,483
NORAD	0,412	0,004	0,136
OVCH1-AS1	0,405	0,001	0,074
MIR4435-2HG	0,405	0,027	0,303
BX890604.2	0,402	0,224	0,646
LINC02211	0,362	0,022	0,285
HCP5	0,347	0,080	0,463
CASC15	0,345	0,261	0,675
HCG11	0,335	0,014	0,233
STARD4-AS1	0,327	0,242	0,659
LINC00342	0,325	0,179	0,600
LINC00894	0,323	0,100	0,489
ADAMTS9-AS2	0,307	0,092	0,481
FGD5-AS1	0,293	0,022	0,285
LINC00174	0,280	0,174	0,593
FLG-AS1	0,264	0,400	0,774
LINC01278	0,255	0,175	0,594
LINC00963	0,253	0,252	0,666
TTTY14	0,252	0,593	0,879
AC097448.1	0,246	0,141	0,550
SNHG8	0,239	0,292	0,703
GABPB1-AS1	0,231	0,426	0,791
LINC00641	0,229	0,251	0,665
SLC16A1-AS1	0,228	0,380	0,761
LINC00632	0,227	0,710	0,923
MIR100HG	0,216	0,218	0,640
PAXIP1-AS2	0,206	0,299	0,710
AC068587.4	0,204	0,414	0,783
AC015813.1	0,200	0,506	0,832
AL356124.1	0,199	0,342	0,737
AL356599.1	0,175	0,350	0,743
LINC02035	0,173	0,400	0,774
SNHG14	0,173	0,583	0,872
TPT1-AS1	0,167	0,360	0,750
EBLN3P	0,164	0,202	0,624
AC098617.1	0,159	0,511	0,836
AC005261.1	0,153	0,403	0,776
PAX8-AS1	0,137	0,700	0,918
AC005670.3	0,131	0,507	0,832
AL137782.1	0,125	0,729	0,930
AL670729.3	0,113	0,778	0,945
EIF1B-AS1	0,109	0,604	0,883
AL021368.2	0,107	0,556	0,858
COX10-AS1	0,106	0,610	0,885
LINC00630	0,102	0,584	0,872
AF117829.1	0,102	0,600	0,882

	SNHG5	0,095	0,593	0,879
	AC006460.2	0,095	0,689	0,914
	HIF1A-AS3	0,079	0,773	0,944
	CCDC18-AS1	0,058	0,758	0,938
	THAP9-AS1	0,051	0,729	0,930
	WAC-AS1	0,050	0,667	0,907
	AC016831.1	0,049	0,883	0,977
	OIP5-AS1	0,049	0,740	0,933
	RASSF8-AS1	0,048	0,843	0,967
	MAGI2-AS3	0,048	0,755	0,937
	TTTY10	0,027	0,955	0,994
	AC021078.1	0,025	0,941	0,992
	DANT2	0,014	0,956	0,994
	AC060780.1	0,009	0,964	0,995
	HAND2-AS1	0,007	0,972	0,998
	SLC8A1-AS1	0,006	0,985	0,999
	C5orf56	0,002	0,995	1,000
	**Down-regulated**			
	AC010186.3	-0,012	0,954	0,994
	ITGA9-AS1	-0,015	0,951	0,993
	RBMS3-AS2	-0,016	0,939	0,992
	PCBP1-AS1	-0,020	0,933	0,991
	AC007406.5	-0,020	0,904	0,981
	AC012459.1	-0,024	0,895	0,979
	LINC02693	-0,025	0,890	0,978
	CHROMR	-0,025	0,889	0,978
	Z83843.1	-0,026	0,910	0,983
	AC093297.2	-0,027	0,906	0,982
	MBNL1-AS1	-0,029	0,897	0,979
	HCG18	-0,035	0,855	0,970
	AL157392.3	-0,037	0,858	0,970
	FTX	-0,040	0,866	0,973
	SH3BP5-AS1	-0,041	0,831	0,962
	SDCBP2-AS1	-0,043	0,794	0,948
	DNM3OS	-0,044	0,844	0,967
	ERVK13-1	-0,052	0,816	0,954
	TRDN-AS1	-0,058	0,915	0,984
	WARS2-AS1	-0,062	0,737	0,932
	AC010680.1	-0,065	0,899	0,980
	LINC01954	-0,068	0,835	0,963
	ZBTB20-AS1	-0,071	0,745	0,935
	LINC00863	-0,071	0,675	0,908
	FAM13A-AS1	-0,073	0,752	0,936
	AC046134.2	-0,075	0,693	0,917
	GMDS-DT	-0,077	0,675	0,908
	TTC28-AS1	-0,077	0,626	0,889
	PLAC4	-0,081	0,769	0,942
	LINC01359	-0,082	0,792	0,948
	FAM106A	-0,085	0,707	0,922
	VLDLR-AS1	-0,085	0,714	0,924
	AC008124.1	-0,090	0,573	0,867
**lncRNA**	NUTM2B-AS1	-0,093	0,668	0,907
	GABPB1-IT1	-0,093	0,610	0,885

GARS-DT	-0,100	0,608	0,885
LINC01719	-0,103	0,668	0,907
LINC01355	-0,112	0,633	0,892
AC092828.1	-0,114	0,745	0,934
SNHG16	-0,116	0,351	0,744
AC245297.3	-0,119	0,401	0,774
THUMPD3-AS1	-0,119	0,406	0,777
AL158152.1	-0,120	0,486	0,822
AC092353.2	-0,127	0,741	0,933
AC004889.1	-0,133	0,595	0,879
AC006059.1	-0,134	0,577	0,870
PSMA3-AS1	-0,136	0,418	0,784
LINC01266	-0,136	0,588	0,875
LINC00662	-0,141	0,327	0,726
DLEU2	-0,143	0,518	0,839
OSER1-DT	-0,147	0,397	0,773
AL080250.1	-0,148	0,732	0,931
ZNF667-AS1	-0,151	0,353	0,744
AC007878.1	-0,161	0,404	0,776
AC003681.1	-0,162	0,498	0,828
AC016831.5	-0,163	0,575	0,868
AC116351.1	-0,167	0,596	0,880
NIPBL-DT	-0,171	0,342	0,738
ZSCAN16-AS1	-0,176	0,315	0,718
LINC00667	-0,179	0,162	0,577
CELF2-AS2	-0,184	0,488	0,823
SNHG1	-0,186	0,201	0,624
AC087854.1	-0,187	0,388	0,767
LINC00710	-0,188	0,560	0,860
DANCR	-0,191	0,266	0,679
PLBD1-AS1	-0,196	0,308	0,715
ENTPD1-AS1	-0,197	0,323	0,724
LINC01578	-0,198	0,292	0,703
NR2F2-AS1	-0,202	0,346	0,740
NPTN-IT1	-0,208	0,456	0,807
AC104825.1	-0,212	0,431	0,794
LINC01184	-0,214	0,170	0,588
LINC00472	-0,216	0,366	0,755
LINC-PINT	-0,216	0,314	0,718
DLEU1	-0,220	0,264	0,677
NUTM2A-AS1	-0,221	0,268	0,682
AC058791.1	-0,223	0,456	0,807
SUCLG2-AS1	-0,225	0,198	0,622
MIR29B2CHG	-0,227	0,475	0,818
AC106791.1	-0,228	0,345	0,740
LINC01128	-0,231	0,332	0,730
MALAT1	-0,231	0,416	0,784
JPX	-0,234	0,442	0,798
AC009318.1	-0,235	0,273	0,685
AF111167.2	-0,238	0,387	0,766
N4BP2L2-IT2	-0,242	0,320	0,721
AC083843.3	-0,244	0,315	0,718
Z95331.1	-0,252	0,414	0,784

MIR22HG	-0,256	0,188	0,613
DUBR	-0,258	0,084	0,470
LINC00882	-0,262	0,204	0,625
MIR99AHG	-0,265	0,091	0,481
FAM157C	-0,271	0,417	0,784
NNT-AS1	-0,287	0,266	0,679
CKMT2-AS1	-0,293	0,219	0,640
KCNQ1OT1	-0,296	0,395	0,771
LINC02248	-0,305	0,430	0,793
LINC00909	-0,305	0,082	0,466
AC100800.1	-0,308	0,426	0,791
MUC20-OT1	-0,319	0,244	0,660
LINC01409	-0,325	0,100	0,489
STARD13-AS	-0,327	0,138	0,546
AC010680.5	-0,344	0,388	0,767
AL627309.5	-0,354	0,310	0,715
AC005332.6	-0,365	0,034	0,332
CACNA1C-AS4	-0,375	0,368	0,756
RAB30-DT	-0,379	0,022	0,284
LINC00513	-0,381	0,246	0,661
TTN-AS1	-0,382	0,434	0,795
AC092279.1	-0,393	0,168	0,585
MIR4458HG	-0,396	0,037	0,345
AL161457.2	-0,397	0,228	0,648
OTUD6B-AS1	-0,398	0,148	0,557
AC009878.2	-0,399	0,267	0,679
TMEM161B-AS1	-0,408	0,132	0,536
AC093843.1	-0,433	0,313	0,717
AC048341.1	-0,436	0,070	0,443
SNHG6	-0,447	0,041	0,359
AC093879.1	-0,450	0,226	0,646
AC009264.1	-0,453	0,357	0,747
CFLAR-AS1	-0,460	0,175	0,594
CU633967.1	-0,472	0,160	0,574
MEG3	-0,477	0,066	0,432
CU634019.1	-0,489	0,162	0,577
TBX5-AS1	-0,494	0,125	0,527
MLIP-AS1	-0,502	0,228	0,648
CU633904.1	-0,521	0,142	0,551
H19	-0,536	0,037	0,344
AL159166.1	-0,538	0,378	0,761
FP236241.1	-0,577	0,088	0,476
AC007126.1	-0,602	0,061	0,416
AL136171.2	-0,607	0,134	0,540
ADAMTS9-AS1	-0,634	0,160	0,574
LUCAT1	-0,660	0,110	0,504
AC106845.1	-0,666	0,014	0,235
AL591543.1	-0,681	0,052	0,388
NEAT1	-0,700	0,179	0,600
LINC02762	-0,770	0,004	0,136
AC117453.1	-0,804	0,004	0,141
AC018464.1	-0,848	0,028	0,310
CARMN	-0,877	0,014	0,233

	MIR133A1HG	-0,909	0,064	0,427
	LINC00881	-1,145	0,002	0,100
	AC087457.1	-1,300	0,002	0,105
	Z85996.3	-1,383	0,007	0,171
	AC119673.2	-1,397	0,006	0,166
	MEG8	-1,399	0,009	0,195
	LINC02541	-1,630	0,000	0,013
	AC107068.1	-1,768	0,003	0,122
	AC107068.2	-2,596	0,000	0,028

Table S2

Gene biotype	Gene name	logFC	P Value	FDR
	**Up-regulated**			
	SULF1	3,147	0,009	0,192
	HBB	2,946	0,005	0,150
	ID4	2,946	0,000	0,009
	KCNT2	2,563	0,007	0,171
	NPR3	2,476	0,000	0,009
	CP	2,358	0,000	0,003
	CDH11	2,340	0,003	0,128
	FRAS1	2,220	0,003	0,115
	HBA1	2,104	0,001	0,073
	HBA2	2,088	0,001	0,064
	PTGS1	1,983	0,001	0,085
	INMT	1,926	0,004	0,138
	FAM107B	1,818	0,008	0,180
	POSTN	1,800	0,006	0,167
	TBX18	1,794	0,002	0,100
	OMD	1,744	0,002	0,091
	EFEMP1	1,680	0,003	0,113
	ALDH1A2	1,664	0,000	0,029
	SMYD2	1,635	0,000	0,009
	CFH	1,634	0,001	0,061
	NR4A2	1,612	0,004	0,143
	SLC39A8	1,576	0,008	0,190
	ASPN	1,564	0,001	0,080
	FMOD	1,490	0,000	0,020
	HLA-DQA1	1,474	0,000	0,039
	DGKI	1,464	0,000	0,049
	TNC	1,445	0,001	0,064
	SERPINE1	1,354	0,002	0,091
	UCHL1	1,345	0,000	0,049
	MYH10	1,341	0,000	0,047
	SIK1	1,340	0,002	0,094
	ECM2	1,318	0,002	0,111
	MXRA5	1,308	0,010	0,203
	SIK1B	1,297	0,002	0,111
	ZNF385B	1,297	0,002	0,094
	LEPR	1,275	0,007	0,177
	IGFBP3	1,243	0,005	0,147
	TEF	1,234	0,000	0,039
	DUSP1	1,233	0,007	0,174
	GRIN2A	1,220	0,009	0,196
	HLA-DPB1	1,202	0,000	0,032
	EPHA3	1,173	0,001	0,087
	F2R	1,170	0,000	0,020
	HSPA2	1,165	0,003	0,123
	HLA-DPA1	1,143	0,001	0,078
	PLEKHA6	1,133	0,001	0,073
	COL8A1	1,126	0,000	0,029
	NOX4	1,113	0,002	0,094
	CSRNP1	1,111	0,006	0,162

HES1	1,097	0,001	0,090
PDGFD	1,077	0,003	0,124
LBH	1,066	0,000	0,029
PLXDC2	1,063	0,004	0,135
SMAD6	1,049	0,003	0,113
ID2	1,049	0,007	0,176
FN1	1,039	0,002	0,112
PER2	1,025	0,000	0,047
BGN	1,023	0,007	0,177
PROS1	1,009	0,000	0,002
LRP5	0,986	0,002	0,110
MTURN	0,973	0,000	0,028
LUM	0,972	0,004	0,133
DUSP6	0,971	0,001	0,076
FZD7	0,964	0,000	0,002
SMAD7	0,960	0,001	0,081
SMAD9	0,949	0,009	0,196
GCNT2	0,949	0,001	0,087
ADM	0,932	0,002	0,112
DPT	0,928	0,002	0,096
ROBO1	0,925	0,002	0,100
ZNF676	0,919	0,003	0,113
LTBP2	0,913	0,003	0,128
PRKCA	0,885	0,008	0,187
SPRY4	0,872	0,009	0,192
MICAL2	0,871	0,000	0,034
MYO1D	0,867	0,002	0,091
PHLDA1	0,867	0,007	0,172
VAT1L	0,865	0,003	0,116
PRSS23	0,864	0,007	0,174
ROR1	0,863	0,000	0,020
ABAT	0,855	0,009	0,193
COL21A1	0,849	0,001	0,087
PER3	0,842	0,002	0,094
HLA-DRB1	0,834	0,004	0,141
TMEM98	0,819	0,003	0,116
MXRA8	0,808	0,004	0,143
IFI6	0,799	0,002	0,106
CYP27A1	0,784	0,001	0,083
UBA7	0,776	0,002	0,107
PNMA8A	0,776	0,003	0,128
CD9	0,769	0,001	0,087
BTN3A1	0,756	0,003	0,123
PIK3IP1	0,749	0,006	0,166
ANTXR1	0,745	0,006	0,171
GATM	0,730	0,007	0,171
COL16A1	0,725	0,008	0,186
CCND1	0,724	0,001	0,087
BHLHE41	0,722	0,000	0,019
DOK5	0,717	0,007	0,173
FMNL3	0,713	0,005	0,148
SERTAD4	0,711	0,004	0,135
ITGB5	0,708	0,002	0,096

TRANK1	0,698	0,009	0,197
IGFBP7	0,693	0,005	0,147
PRICKLE1	0,685	0,000	0,046
DNAJB1	0,678	0,000	0,055
HTRA1	0,674	0,001	0,061
KDELR3	0,671	0,001	0,073
RAB11FIP1	0,667	0,001	0,081
DDR1	0,634	0,006	0,160
XPC	0,629	0,001	0,061
PLXNB2	0,625	0,005	0,152
ATP7B	0,625	0,002	0,094
GPX8	0,611	0,006	0,161
ETV5	0,607	0,003	0,123
ZNF704	0,599	0,005	0,153
USP11	0,591	0,005	0,157
HMGN2	0,584	0,002	0,106
ODC1	0,579	0,007	0,177
BCL2L11	0,565	0,004	0,138
GAB2	0,549	0,009	0,193
TMCO3	0,545	0,000	0,013
SLC16A12	0,542	0,008	0,190
EXT1	0,534	0,001	0,087
ULK2	0,534	0,000	0,039
COPZ2	0,516	0,001	0,064
CDR2	0,508	0,003	0,123
ID3	0,494	0,008	0,186
HLF	0,484	0,008	0,183
ALDH18A1	0,470	0,008	0,190
MR1	0,467	0,006	0,164
KIF13A	0,467	0,002	0,107
ENAH	0,465	0,002	0,110
DCTD	0,437	0,003	0,116
IGIP	0,416	0,003	0,117
SEC31A	0,416	0,000	0,035
CTTN	0,414	0,009	0,191
LTBP1	0,412	0,004	0,133
ARMCX2	0,404	0,004	0,133
YWHAQ	0,401	0,003	0,114
FAM114A1	0,398	0,008	0,180
ETV3	0,395	0,010	0,203
TERF2IP	0,385	0,001	0,073
DOCK1	0,371	0,006	0,169
DNAL1	0,366	0,008	0,188
PTPRA	0,365	0,006	0,171
CDKN1B	0,326	0,007	0,171
SRSF8	0,299	0,008	0,190
COPA	0,283	0,008	0,180
**Down-regulated**			
AK2	-0,290	0,004	0,143
COA1	-0,301	0,004	0,143
EMC7	-0,328	0,005	0,152
PPIC	-0,357	0,008	0,182
STARD13	-0,357	0,005	0,152

EI24	-0,358	0,007	0,171
C1orf43	-0,358	0,007	0,177
CBWD3	-0,361	0,009	0,193
UBE2L3	-0,364	0,007	0,171
MKKS	-0,368	0,005	0,150
ASB8	-0,373	0,008	0,190
ZNF438	-0,376	0,008	0,180
MARC2	-0,383	0,004	0,133
MTCH2	-0,390	0,006	0,167
DNAJC27	-0,405	0,006	0,170
ADGRF5	-0,406	0,007	0,176
MTX2	-0,406	0,008	0,186
TMX2	-0,408	0,001	0,078
RNF5	-0,409	0,007	0,171
PSMB7	-0,413	0,005	0,150
DUSP22	-0,418	0,002	0,107
DNAJC21	-0,424	0,003	0,113
LAP3	-0,427	0,008	0,180
MALSU1	-0,427	0,005	0,154
MRPL37	-0,434	0,007	0,174
FAR2	-0,435	0,007	0,172
CACYBP	-0,438	0,009	0,191
HINT1	-0,440	0,007	0,176
MRPS5	-0,440	0,003	0,115
TIMM23	-0,444	0,003	0,114
PLPP3	-0,444	0,001	0,090
SPTSSA	-0,446	0,008	0,180
MRPL58	-0,455	0,008	0,187
APIP	-0,459	0,010	0,206
MSRA	-0,460	0,007	0,171
CHCHD1	-0,462	0,006	0,167
FARS2	-0,462	0,009	0,197
ME2	-0,464	0,009	0,193
FASTKD5	-0,465	0,003	0,114
MRPS15	-0,465	0,002	0,105
MRPS9	-0,466	0,010	0,201
STOX2	-0,466	0,001	0,073
SLC36A4	-0,468	0,005	0,152
HCCS	-0,471	0,009	0,197
RRP15	-0,476	0,005	0,144
SUCLG1	-0,479	0,010	0,202
CALCOCO2	-0,486	0,000	0,040
PTRH2	-0,489	0,007	0,180
PSMB3	-0,491	0,009	0,192
MPP1	-0,491	0,002	0,104
WDR12	-0,500	0,001	0,073
MTRF1L	-0,502	0,001	0,056
PTPRG	-0,505	0,010	0,205
AASS	-0,506	0,009	0,195
TUBB6	-0,507	0,005	0,144
ACOT13	-0,508	0,001	0,084
TRIM69	-0,513	0,002	0,111
SSBP1	-0,513	0,008	0,189

**Protein Coding**	TEK	-0,514	0,001	0,090
	MRPL15	-0,515	0,001	0,085
	SH3KBP1	-0,520	0,003	0,114
	FUNDC2	-0,521	0,004	0,143
	C2orf88	-0,524	0,004	0,136
	PSMA3	-0,524	0,001	0,087
	PSMD7	-0,524	0,004	0,143
	HDDC2	-0,530	0,003	0,123
	COPS5	-0,531	0,006	0,164
	UBE2F	-0,534	0,003	0,114
	YARS	-0,536	0,008	0,187
	CTSB	-0,536	0,007	0,180
	CHCHD4	-0,536	0,002	0,094
	MRPL51	-0,537	0,006	0,161
	B4GALT6	-0,540	0,007	0,174
	PDCD5	-0,541	0,009	0,192
	H2AFZ	-0,543	0,010	0,203
	GNB5	-0,543	0,002	0,113
	SNRNP25	-0,544	0,009	0,195
	TMEM273	-0,545	0,001	0,073
	EMC3	-0,546	0,006	0,171
	COX14	-0,548	0,003	0,113
	KPNA2	-0,549	0,004	0,143
	CBLB	-0,549	0,008	0,185
	AIF1L	-0,553	0,002	0,098
	PDHA1	-0,555	0,007	0,176
	NDUFB4	-0,556	0,005	0,151
	PRDX6	-0,559	0,003	0,128
	GTF3C6	-0,560	0,003	0,113
	SERPINB6	-0,563	0,001	0,073
	QRSL1	-0,564	0,005	0,148
	MRPL27	-0,564	0,001	0,061
	RALGPS2	-0,568	0,001	0,090
	TBC1D1	-0,572	0,001	0,061
	NDUFC1	-0,572	0,006	0,167
	ADK	-0,577	0,001	0,084
	PMP22	-0,578	0,001	0,073
	SMIM12	-0,579	0,000	0,020
	MICOS10	-0,579	0,000	0,038
	KLHL23	-0,581	0,010	0,205
	NDUFB2	-0,584	0,005	0,156
	NDUFB10	-0,584	0,005	0,147
	MTHFD2	-0,585	0,003	0,123
	TRNP1	-0,585	0,009	0,200
	TGFBI	-0,587	0,009	0,192
	SELENOW	-0,587	0,007	0,177
	HSDL1	-0,588	0,001	0,064
	AKTIP	-0,590	0,000	0,039
	TBC1D7	-0,593	0,002	0,094
	ATP5MC3	-0,594	0,002	0,097
	TMTC1	-0,594	0,003	0,123
	NCALD	-0,594	0,008	0,180
	UQCRFS1	-0,600	0,004	0,138

GTF3A	-0,605	0,000	0,013
FKBP3	-0,607	0,001	0,080
HMOX2	-0,612	0,001	0,064
TOMM40L	-0,613	0,004	0,138
TIMM21	-0,626	0,005	0,158
COX6B1	-0,627	0,007	0,174
SLC39A14	-0,629	0,008	0,180
UTP11	-0,631	0,003	0,116
CD59	-0,634	0,000	0,039
ARL3	-0,642	0,001	0,073
MRPS33	-0,643	0,004	0,143
SDHB	-0,646	0,004	0,143
CHCHD3	-0,646	0,001	0,056
EDA	-0,648	0,006	0,171
NDUFB9	-0,649	0,001	0,076
RAD1	-0,652	0,000	0,015
NDUFA6	-0,653	0,007	0,176
DIPK2B	-0,655	0,003	0,115
CHPT1	-0,657	0,002	0,108
STXBP6	-0,658	0,005	0,153
RTN4IP1	-0,661	0,004	0,133
RNF144B	-0,664	0,002	0,107
SLC25A26	-0,664	0,000	0,029
APOO	-0,665	0,001	0,090
ATP5PF	-0,667	0,003	0,128
ALDOC	-0,668	0,007	0,171
PDGFRL	-0,668	0,005	0,144
CTSL	-0,670	0,000	0,034
GFOD1	-0,677	0,007	0,171
TPD52L1	-0,680	0,002	0,107
MTERF3	-0,688	0,000	0,029
NDUFB1	-0,696	0,005	0,156
COQ3	-0,696	0,004	0,136
PLGRKT	-0,696	0,006	0,165
EPB41L3	-0,696	0,008	0,180
MRPL33	-0,697	0,003	0,113
PVR	-0,699	0,001	0,080
MEIS1	-0,703	0,001	0,080
DAPK2	-0,703	0,010	0,205
ULK4	-0,705	0,000	0,042
RASL12	-0,710	0,005	0,145
CMC2	-0,710	0,002	0,105
NDUFS5	-0,712	0,009	0,193
RSRC1	-0,714	0,000	0,041
COQ10A	-0,715	0,007	0,178
PDE4D	-0,719	0,009	0,199
GATB	-0,724	0,004	0,136
ATP5MPL	-0,727	0,004	0,141
EGFLAM	-0,731	0,004	0,141
ATP5PO	-0,735	0,004	0,133
SPARCL1	-0,737	0,002	0,111
DESI1	-0,748	0,001	0,090
MYO5C	-0,766	0,003	0,123

ATP5MC1	-0,770	0,002	0,100
ATP5F1C	-0,770	0,005	0,153
EBF2	-0,772	0,002	0,091
MTIF3	-0,774	0,000	0,020
PPM1L	-0,781	0,000	0,034
CAMK2A	-0,783	0,007	0,171
PRDX1	-0,788	0,002	0,094
COX7B	-0,788	0,003	0,113
C11orf21	-0,792	0,006	0,171
GPSM2	-0,792	0,002	0,095
NDUFA4	-0,793	0,007	0,173
SIGLEC1	-0,804	0,006	0,171
SKAP2	-0,806	0,000	0,017
HSF2	-0,807	0,001	0,076
NDUFA1	-0,812	0,004	0,133
MRPS25	-0,813	0,000	0,029
SPSB1	-0,813	0,002	0,107
ENPEP	-0,815	0,001	0,090
ITGB1BP2	-0,816	0,008	0,189
NDUFB6	-0,821	0,003	0,123
BCL6	-0,834	0,005	0,153
ME1	-0,840	0,000	0,013
UAP1	-0,846	0,009	0,196
MRC1	-0,847	0,007	0,173
STEAP4	-0,850	0,002	0,107
FCER1G	-0,854	0,005	0,157
RGS6	-0,860	0,001	0,078
TIMM17A	-0,860	0,000	0,049
ATP2A2	-0,868	0,008	0,189
IDH2	-0,869	0,002	0,110
COX17	-0,871	0,000	0,047
TRMT9B	-0,872	0,008	0,184
PPP1CC	-0,873	0,000	0,002
AACS	-0,873	0,005	0,144
TOGARAM2	-0,874	0,004	0,138
TBX3	-0,887	0,000	0,047
NT5DC2	-0,889	0,009	0,196
ISOC1	-0,893	0,000	0,034
NDUFB3	-0,898	0,006	0,167
UQCRB	-0,905	0,007	0,177
COX6C	-0,906	0,005	0,152
SCN9A	-0,913	0,004	0,140
GLUL	-0,916	0,006	0,167
PPIL1	-0,922	0,000	0,023
C6	-0,938	0,002	0,109
PYGM	-0,939	0,001	0,076
LRRC10	-0,943	0,005	0,153
MS4A4A	-0,949	0,001	0,090
INSYN1	-0,950	0,006	0,166
NDUFAB1	-0,952	0,000	0,047
NDUFA12	-0,960	0,001	0,083
NID1	-0,961	0,003	0,113
PITX2	-0,965	0,001	0,064

EGF	-0,968	0,009	0,197
RAP1GAP2	-0,983	0,004	0,141
PLP1	-0,983	0,001	0,074
CADPS2	-1,007	0,000	0,032
LILRB5	-1,008	0,001	0,068
FAM107A	-1,010	0,004	0,136
MYBPHL	-1,022	0,000	0,047
ENO3	-1,030	0,006	0,171
GCOM1	-1,049	0,006	0,171
PLTP	-1,050	0,001	0,078
DCLK1	-1,057	0,004	0,137
ARNTL	-1,058	0,000	0,009
HTR4	-1,058	0,000	0,009
KCNIP2	-1,059	0,002	0,092
LRMDA	-1,071	0,000	0,009
CMTM5	-1,082	0,003	0,128
FABP5	-1,086	0,000	0,028
TNFRSF19	-1,094	0,000	0,022
OSR1	-1,099	0,000	0,029
GABRB3	-1,099	0,002	0,092
PTGFR	-1,104	0,003	0,113
CILP	-1,111	0,005	0,144
ACTA2	-1,120	0,002	0,111
TGFBR3	-1,128	0,000	0,035
STC2	-1,131	0,000	0,038
MYOC	-1,145	0,006	0,159
PTDSS1	-1,150	0,000	0,008
PLAU	-1,174	0,004	0,134
LRRC7	-1,181	0,004	0,141
REPS2	-1,186	0,001	0,060
SLC1A3	-1,187	0,001	0,073
MYL4	-1,188	0,004	0,141
CHL1	-1,189	0,000	0,047
VIT	-1,213	0,003	0,123
RETREG1	-1,226	0,006	0,161
CD209	-1,266	0,000	0,025
KCNN2	-1,273	0,000	0,013
S1PR3	-1,282	0,000	0,009
LRP1B	-1,288	0,005	0,150
LYVE1	-1,356	0,000	0,025
SLC27A6	-1,424	0,000	0,009
AKR1B1	-1,434	0,000	0,039
VSIG4	-1,445	0,000	0,029
FBN2	-1,474	0,001	0,090
MFAP5	-1,544	0,001	0,090
ADAMTS4	-1,564	0,000	0,039
CR1	-1,587	0,001	0,065
DHRS9	-1,617	0,001	0,076
APOD	-1,629	0,000	0,013
METTL7B	-1,657	0,001	0,073
F13A1	-1,673	0,000	0,002
SNX22	-1,691	0,000	0,041
LRRN3	-1,831	0,000	0,039

	CRISPLD2	-2,019	0,000	0,003
	NFXL1	-2,033	0,000	0,039
	CD163	-2,147	0,000	0,039
**lncRNA**	**Up-regulated**			
	AC159540.2	0,792	0,004	0,133
	SNHG29	0,558	0,009	0,197
	NORAD	0,412	0,004	0,136
	OVCH1-AS1	0,405	0,001	0,074
	**Down-reguated**			
	LINC02762	-0,770	0,004	0,136
	AC117453.1	-0,804	0,004	0,141
	LINC00881	-1,145	0,002	0,100
	AC087457.1	-1,300	0,002	0,105
	Z85996.3	-1,383	0,007	0,171
	AC119673.2	-1,397	0,006	0,166
	MEG8	-1,399	0,009	0,195
	LINC02541	-1,630	0,000	0,013
	AC107068.1	-1,768	0,003	0,122
	AC107068.2	-2,596	0,000	0,028

Table S3. Gene Ontology Biological Process for over-expressed genes

Signalling	DEG number	P DE	Gene name
Biological process	140	0,0216	CFH, COPZ2, TMEM98, CD9, BTN3A1, GAB2, TNC, EPHA3, CP, PER3, LTBP1, ALDH18A1, ITGB5, ULK2, COL16A1, CTTN, NOX4, PTGS1, PIK3IP1, KDELR3, DOK5, SMAD7, MXRA5, USP11, SERPINE1, ASPN, ECM2, HLF, CCND1, CDKN1B, GCNT2, TBX18, NPR3, HES1, EFEMP1, FN1, ID2, ODC1, LEPR, ETV3, ID3, DNAL1, LTBP2, DUSP1, SMAD9, FMOD, COPA, BHLHE41, ATP7B, COL21A1, IFI6, HSPA2, OMD, ALDH1A2, DCTD, DNAJB1, PER2, PTPRA", "MYH10, POSTN, MICAL2, YWHAQ, CYP27A1, KIF13A, SULF1, SMAD6, SEC31A, FRAS1, SLC39A8, PRICKLE1, PHLDA1, DUSP6, LUM, CDH11, SIK1, DPT, SMYD2, ZNF385B, CSRNP1, COL8A1, IGFBP3, ADM, TMCO3, PRSS23, DOCK1, SLC16A12, MR1, BCL2L11, NR4A2, PRKCA, UCHL1, ENAH, XPC, FZD7, RAB11FIP1, DGKI, FMNL3, LRP5, MXRA8, KCNT2, IGFBP7, GPX8, ZNF704, HTRA1, TERF2IP, TEF, ANTXR1, ROBO1, PDGFD, VAT1L, GATM, ID4, SNHG29", "MYO1D, MTURN, F2R, UBA7, EXT1, BGN, ABAT, GRIN2A, PROS1, ROR1, SPRY4, HBA2, ZNF676, HLA-DRB1, PLXNB2, HLA-DQA1, HMGN2, DDR1, HBA1, LBH, HLA-DPB1, HLA-DPA1, INMT, ETV5, HBB, SRSF8, SIK1B, HYDIN2

Cellular process	130	0,0247	TMEM98, CD9, BTN3A1, GAB2, TNC, EPHA3, CP, PER3, LTBP1, ALDH18A1, ITGB5, ULK2, COL16A1, CTTN, NOX4, PTGS1, PIK3IP1, KDELR3, DOK5, SMAD7, USP11, SERPINE1, ASPN, ECM2, HLF, CCND1, CDKN1B, GCNT2, TBX18, NPR3, HES1, EFEMP1, FN1, ID2, ODC1, LEPR, ETV3, ID3, DNAL1, LTBP2, DUSP1, SMAD9, FMOD, COPA, BHLHE41, ATP7B, COL21A1, IFI6, HSPA2, OMD, ALDH1A2, DCTD, DNAJB1, PER2, PTPRA, MYH10, POSTN", "MICAL2, YWHAQ, CYP27A1, KIF13A, SULF1, SMAD6, SEC31A, FRAS1, SLC39A8, PRICKLE1, PHLDA1, DUSP6, LUM, CDH11, SIK1, DPT, SMYD2, ZNF385B, CSRNP1, COL8A1, IGFBP3, ADM, PRSS23, DOCK1, BCL2L11, NR4A2, PRKCA, UCHL1, ENAH, XPC, FZD7, RAB11FIP1, DGKI, FMNL3, LRP5, MXRA8, KCNT2, IGFBP7, GPX8, ZNF704, HTRA1, TERF2IP, TEF, ANTXR1, ROBO1, PDGFD, GATM, ID4, SNHG29, MYO1D, F2R, UBA7, EXT1, BGN, ABAT, GRIN2A", "PROS1, ROR1, SPRY4, HBA2, ZNF676, HLA-DRB1, PLXNB2, HLA-DQA1, DDR1, HBA1, LBH, HLA-DPB1, HLA-DPA1, ETV5, HBB, SRSF8, SIK1B, HYDIN2

Metabolic process	102	0,0188	CFH, TMEM98, CD9, TNC, EPHA3, CP, PER3, LTBP1, ALDH18A1, ULK2, CTTN, NOX4, PTGS1, PIK3IP1, DOK5, SMAD7, USP11, SERPINE1, HLF, CCND1, CDKN1B, GCNT2, TBX18, NPR3, HES1, EFEMP1, FN1, ID2, ODC1, LEPR, ETV3, ID3, DUSP1, SMAD9, FMOD, BHLHE41, ATP7B, IFI6, HSPA2, OMD, ALDH1A2, DCTD, DNAJB1, PER2, PTPRA, MICAL2, YWHAQ, CYP27A1, SULF1, SMAD6, PRICKLE1, PHLDA1, DUSP6, LUM, SIK1, SMYD2, CSRNP1", "IGFBP3, ADM, PRSS23, BCL2L11, NR4A2, PRKCA, UCHL1, XPC, FZD7, DGKI, LRP5, MXRA8, IGFBP7, GPX8, ZNF704, HTRA1, TERF2IP, TEF, ANTXR1, ROBO1, PDGFD, VAT1L, GATM, ID4, SNHG29, MYO1D, F2R, UBA7, EXT1, BGN, ABAT, GRIN2A, PROS1, ROR1, SPRY4, HBA2, ZNF676, PLXNB2, DDR1, HBA1, LBH, INMT, ETV5, HBB, SRSF8, SIK1B, HYDIN2

Multicellular organismal process	97	0,0000	TMEM98, CD9, BTN3A1, GAB2, TNC, EPHA3, PER3, LTBP1, ITGB5, ULK2, COL16A1, CTTN, NOX4, PTGS1, DOK5, SMAD7, SERPINE1, ASPN, HLF, CCND1, CDKN1B, GCNT2, TBX18, NPR3, HES1, EFEMP1, FN1, ID2, ODC1, LEPR, ID3, COPA, BHLHE41, ATP7B, COL21A1, HSPA2, OMD, ALDH1A2, DNAJB1, PER2, PTPRA, MYH10, POSTN, MICAL2, YWHAQ, SULF1, SMAD6, FRAS1, PRICKLE1, DUSP6, LUM, CDH11, SIK1, SMYD2, CSRNP1, COL8A1, IGFBP3", "ADM, DOCK1, MR1, BCL2L11, NR4A2, PRKCA, UCHL1, ENAH, FZD7, RAB11FIP1, DGKI, FMNL3, LRP5, MXRA8, IGFBP7, HTRA1, ANTXR1, ROBO1, PDGFD, GATM, ID4, MYO1D, MTURN, F2R, UBA7, EXT1, BGN, ABAT, GRIN2A, PROS1, ROR1, SPRY4, PLXNB2, DDR1, LBH, HLA-DPB1, HLA-DPA1, ETV5, HBB

Cellular metabolic process	96	0,0058	TMEM98, CD9, TNC, EPHA3, CP, PER3, LTBP1, ALDH18A1, ULK2, CTTN, NOX4, PTGS1, PIK3IP1, DOK5, SMAD7, USP11, SERPINE1, HLF, CCND1, CDKN1B, GCNT2, TBX18, HES1, EFEMP1, FN1, ID2, ODC1, LEPR, ETV3, ID3, DUSP1, SMAD9, FMOD, BHLHE41, IFI6, HSPA2, OMD, ALDH1A2, DCTD, DNAJB1, PER2, PTPRA, MICAL2, YWHAQ, CYP27A1, SULF1, SMAD6, PRICKLE1, PHLDA1, DUSP6, LUM, SIK1, SMYD2, CSRNP1, IGFBP3, ADM, PRSS23", "BCL2L11, NR4A2, PRKCA, UCHL1, XPC, FZD7, DGKI, LRP5, MXRA8, IGFBP7, ZNF704, HTRA1, TERF2IP, TEF, ANTXR1, ROBO1, PDGFD, GATM, ID4, SNHG29, MYO1D, F2R, UBA7, EXT1, BGN, ABAT, GRIN2A, PROS1, ROR1, SPRY4, HBA2, ZNF676, PLXNB2, DDR1, HBA1, LBH, ETV5, HBB, SRSF8, SIK1B, HYDIN2

Developmental process	84	0,0000	TMEM98, CD9, GAB2, TNC, EPHA3, LTBP1, ITGB5, ULK2, CTTN, NOX4, DOK5, SMAD7, SERPINE1, ASPN, HLF, CCND1, CDKN1B, GCNT2, TBX18, NPR3, HES1, EFEMP1, FN1, ID2, ODC1, LEPR, ETV3, ID3, BHLHE41, ATP7B, COL21A1, HSPA2, OMD, ALDH1A2, DNAJB1, PER2, PTPRA, MYH10, POSTN, MICAL2, YWHAQ, SULF1, SMAD6, FRAS1, PRICKLE1, DUSP6, LUM, CDH11, SIK1, SMYD2, CSRNP1, COL8A1, IGFBP3, ADM, DOCK1, BCL2L11, NR4A2", "PRKCA, UCHL1, ENAH, FZD7, FMNL3, LRP5, MXRA8, IGFBP7, HTRA1, ANTXR1, ROBO1, PDGFD, GATM, ID4, MYO1D, MTURN, F2R, EXT1, ABAT, GRIN2A, ROR1, SPRY4, PLXNB2, DDR1, LBH, ETV5
Anatomical structure development	83	0,0000	TMEM98, CD9, GAB2, TNC, EPHA3, LTBP1, ITGB5, ULK2, CTTN, NOX4, DOK5, SMAD7, SERPINE1, ASPN, HLF, CCND1, CDKN1B, GCNT2, TBX18, NPR3, HES1, EFEMP1, FN1, ID2, ODC1, LEPR, ID3, BHLHE41, ATP7B, COL21A1, HSPA2, OMD, ALDH1A2, DNAJB1, PER2, PTPRA, MYH10, POSTN, MICAL2, YWHAQ, SULF1, SMAD6, FRAS1, PRICKLE1, DUSP6, LUM, CDH11, SIK1, SMYD2, CSRNP1, COL8A1, IGFBP3, ADM, DOCK1, BCL2L11, NR4A2, PRKCA", "UCHL1, ENAH, FZD7, FMNL3, LRP5, MXRA8, IGFBP7, HTRA1, ANTXR1, ROBO1, PDGFD, GATM, ID4, MYO1D, MTURN, F2R, EXT1, ABAT, GRIN2A, ROR1, SPRY4, PLXNB2, DDR1, LBH, ETV5

Cell communication	77	0,0000	BTN3A1, GAB2, TNC, EPHA3, LTBP1, ITGB5, ULK2, COL16A1, CTTN, NOX4, PIK3IP1, KDELR3, DOK5, SMAD7, SERPINE1, ASPN, CCND1, CDKN1B, GCNT2, TBX18, NPR3, HES1, EFEMP1, FN1, LEPR, LTBP2, DUSP1, SMAD9, FMOD, COPA, IFI6, ALDH1A2, PER2, PTPRA, POSTN, YWHAQ, SULF1, SMAD6, SEC31A, FRAS1, PRICKLE1, DUSP6, CDH11, SIK1, SMYD2, ZNF385B, CSRNP1, IGFBP3, ADM, DOCK1, BCL2L11, NR4A2, PRKCA, UCHL1, FZD7, RAB11FIP1", "DGKI, LRP5, HTRA1, TERF2IP, ROBO1, PDGFD, F2R, EXT1, ABAT, GRIN2A, ROR1, SPRY4, HLA-DRB1, PLXNB2, HLA-DQA1, DDR1, LBH, HLA-DPB1, HLA-DPA1, ETV5, SIK1B
Signaling	76	0,0000	BTN3A1, GAB2, TNC, EPHA3, LTBP1, ITGB5, ULK2, COL16A1, CTTN, NOX4, PIK3IP1, KDELR3, DOK5, SMAD7, SERPINE1, ASPN, CCND1, CDKN1B, GCNT2, TBX18, NPR3, HES1, EFEMP1, FN1, LEPR, LTBP2, DUSP1, SMAD9, FMOD, COPA, IFI6, ALDH1A2, PER2, PTPRA, POSTN, YWHAQ, SULF1, SMAD6, SEC31A, PRICKLE1, DUSP6, CDH11, SIK1, SMYD2, ZNF385B, CSRNP1, IGFBP3, ADM, DOCK1, BCL2L11, NR4A2, PRKCA, UCHL1, FZD7, RAB11FIP1", "DGKI, LRP5, HTRA1, TERF2IP, ROBO1, PDGFD, F2R, EXT1, ABAT, GRIN2A, ROR1, SPRY4, HLA-DRB1, PLXNB2, HLA-DQA1, DDR1, LBH, HLA-DPB1, HLA-DPA1, ETV5, SIK1B

System development	73	0,0000	TMEM98, CD9, GAB2, TNC, EPHA3, LTBP1, ULK2, CTTN, NOX4, DOK5, SMAD7, SERPINE1, ASPN, HLF, CCND1, CDKN1B, GCNT2, TBX18, NPR3, HES1, EFEMP1, FN1, ID2, ODC1, LEPR, ID3, BHLHE41, ATP7B, COL21A1, OMD, ALDH1A2, DNAJB1, PER2, PTPRA, MYH10, POSTN, MICAL2, YWHAQ, SULF1, SMAD6, FRAS1, PRICKLE1, DUSP6, LUM, CDH11, SIK1, SMYD2, CSRNP1, COL8A1, ADM, BCL2L11, NR4A2, PRKCA, UCHL1, ENAH, FZD7, FMNL3", "LRP5, MXRA8, HTRA1, ANTXR1, ROBO1, PDGFD, ID4, MYO1D, EXT1, ABAT, GRIN2A, ROR1, PLXNB2, DDR1, LBH, ETV5
Cellular component organization	67	0,0002	CD9, GAB2, TNC, EPHA3, ITGB5, ULK2, COL16A1, CTTN, NOX4, DOK5, SMAD7, SERPINE1, ECM2, CDKN1B, HES1, FN1, ID2, DNAL1, LTBP2, DUSP1, FMOD, COL21A1, IFI6, HSPA2, ALDH1A2, DNAJB1, PER2, PTPRA, MYH10, POSTN, MICAL2, YWHAQ, KIF13A, SULF1, SMAD6, SEC31A, LUM, CDH11, DPT, SMYD2, COL8A1, IGFBP3, ADM, DOCK1, BCL2L11, NR4A2, PRKCA, UCHL1, ENAH, XPC, FZD7, FMNL3, LRP5, IGFBP7, HTRA1, TERF2IP, ANTXR1", "ROBO1, F2R, EXT1, BGN, HBA2, PLXNB2, DDR1, HBA1, ETV5, HBB

Cell differentiation	61	0,0000	TMEM98, CD9, GAB2, TNC, EPHA3, ITGB5, ULK2, CTTN, NOX4, DOK5, SMAD7, SERPINE1, HLF, CCND1, CDKN1B, GCNT2, TBX18, HES1, EFEMP1, FN1, ID2, LEPR, ETV3, ID3, BHLHE41, COL21A1, HSPA2, ALDH1A2, PER2, PTPRA, MYH10, POSTN, SULF1, SMAD6, PRICKLE1, DUSP6, CDH11, SIK1, COL8A1, IGFBP3, ADM, DOCK1, NR4A2, PRKCA, UCHL1, ENAH, FZD7, LRP5, MXRA8, HTRA1, ANTXR1, ROBO1, ID4, EXT1, GRIN2A, ROR1, PLXNB2",<br>"DDR1, LBH, ETV5
Signal transduction	61	0,0000	BTN3A1, GAB2, EPHA3, LTBP1, ITGB5, ULK2, COL16A1, CTTN, NOX4, PIK3IP1, KDELR3, DOK5, SMAD7, SERPINE1, ASPN, CCND1, CDKN1B, GCNT2, TBX18, NPR3, HES1, EFEMP1, FN1, LEPR, LTBP2, DUSP1, SMAD9, FMOD, COPA, IFI6, ALDH1A2, PTPRA, POSTN, YWHAQ, SULF1, SMAD6, SEC31A, PRICKLE1, DUSP6, SIK1, SMYD2, ZNF385B, CSRNP1, IGFBP3, ADM, DOCK1, BCL2L11, NR4A2, PRKCA, UCHL1, FZD7, DGKI, LRP5, HTRA1, TERF2IP",<br>"ROBO1, PDGFD, F2R, EXT1, ABAT, GRIN2A, ROR1, SPRY4, HLA-DRB1, PLXNB2, HLA-DQA1, DDR1, LBH, HLA-DPB1, HLA-DPA1, SIK1B

Biosynthetic process	58	0,0147	PER3, ALDH18A1, NOX4, PTGS1, SMAD7, SERPINE1, HLF, CCND1, CDKN1B, GCNT2, TBX18, HES1, EFEMP1, ID2, ODC1, LEPR, ETV3, ID3, DUSP1, SMAD9, FMOD, BHLHE41, OMD, ALDH1A2, DCTD, DNAJB1, PER2, MICAL2, YWHAQ, CYP27A1, SULF1, SMAD6, PRICKLE1, PHLDA1, LUM, SIK1, SMYD2, CSRNP1, ADM, NR4A2, FZD7, LRP5, IGFBP7, ZNF704, HTRA1, TERF2IP, TEF, GATM, ID4, F2R, EXT1, BGN, ABAT, ROR1, ZNF676, LBH, ETV5",<br>"HBB, HYDIN2
Protein modification process	58	0,0017	TNC, EPHA3, CP, LTBP1, ULK2, NOX4, DOK5, SMAD7, USP11, CCND1, CDKN1B, GCNT2, HES1, EFEMP1, FN1, DUSP1, HSPA2, PER2, PTPRA, SMAD6, PRICKLE1, DUSP6, SIK1, SMYD2, IGFBP3, PRSS23, PRKCA, UCHL1, FZD7, LRP5, MXRA8, IGFBP7, TERF2IP, ROBO1, PDGFD, F2R, UBA7, EXT1, BGN, ROR1, SPRY4, PLXNB2, DDR1, LBH, SIK1B
Cellular biosynthetic process	57	0,0129	PER3, ALDH18A1, NOX4, PTGS1, SMAD7, SERPINE1, HLF, CCND1, CDKN1B, GCNT2, TBX18, HES1, EFEMP1, ID2, ODC1, LEPR, ETV3, ID3, DUSP1, SMAD9, FMOD, BHLHE41, OMD, ALDH1A2, DCTD, DNAJB1, PER2, MICAL2, YWHAQ, CYP27A1, SULF1, SMAD6, PRICKLE1, PHLDA1, LUM, SIK1, SMYD2, CSRNP1, ADM, NR4A2, FZD7, LRP5, ZNF704, HTRA1, TERF2IP, TEF, GATM, ID4, F2R, EXT1, BGN, ABAT, ROR1, ZNF676, LBH, ETV5, HBB, HYDIN2

Anatomical structure morphogenesis	53	0,0000	CD9, GAB2, TNC, EPHA3, ITGB5, ULK2, CTTN, NOX4, DOK5, SMAD7, SERPINE1, ASPN, TBX18, HES1, EFEMP1, FN1, ID2, LEPR, ID3, BHLHE41, COL21A1, ALDH1A2, PTPRA, MYH10, POSTN, MICAL2, SULF1, SMAD6, FRAS1, PRICKLE1, CDH11, CSRNP1, COL8A1, ADM, DOCK1, BCL2L11, NR4A2, PRKCA, UCHL1, ENAH, FZD7, FMNL3, LRP5, HTRA1, ANTXR1, ROBO1, ID4, F2R, EXT1, ROR1, PLXNB2, DDR1, ETV5
Response to stress	53	0,0000	CFH, CD9, TNC, ULK2, NOX4, PTGS1, KDELR3, SMAD7, SERPINE1, CCND1, CDKN1B, FN1, ID2, ID3, DUSP1, IFI6, HSPA2, DNAJB1, PER2, MYH10, POSTN, SMAD6, SEC31A, DUSP6, SIK1, SMYD2, ADM, DOCK1, MR1, BCL2L11, NR4A2, PRKCA, UCHL1, XPC, FZD7, DGKI, IGFBP7, GPX8, HTRA1, TERF2IP, PDGFD, F2R, UBA7, ABAT, GRIN2A, PROS1, HBA2, HLA-DRB1, HLA-DQA1, DDR1, HBA1, HLA-DPB1, HLA-DPA1, ETV5, HBB, SIK1B
Transport	49	0,0158	COPZ2, CD9, BTN3A1, GAB2, EPHA3, CP, CTTN, KDELR3, SERPINE1, CCND1, CDKN1B, NPR3, FN1, LEPR, LTBP2, COPA, ATP7B, HSPA2, PER2, MYH10, POSTN, YWHAQ, KIF13A, SEC31A, FRAS1, SLC39A8, PRICKLE1, SIK1, ADM, TMCO3, DOCK1, SLC16A12, BCL2L11, PRKCA, UCHL1, RAB11FIP1, DGKI, LRP5, KCNT2, ANTXR1, MYO1D, F2R, ABAT, GRIN2A, PROS1, HBA2, DDR1, HBA1, HBB

Regulation of gene expression	48	0,0199	CFH, TMEM98, TNC, PER3, SMAD7, SERPINE1, HLF, CCND1, CDKN1B, GCNT2, TBX18, HES1, EFEMP1, FN1, ID2, ETV3, ID3, SMAD9, BHLHE41, ALDH1A2, DNAJB1, PER2, MICAL2, YWHAQ, SULF1, SMAD6, PRICKLE1, LUM, SIK1, SMYD2, CSRNP1, NR4A2, PRKCA, FZD7, LRP5, ZNF704, HTRA1, TERF2IP, TEF, ROBO1, ID4, F2R, PROS1, ROR1, ZNF676, LBH, ETV5, SRSF8
Regulation of cell communication	46	0,0000	LTBP1, CTTN, NOX4, PIK3IP1, DOK5, SMAD7, SERPINE1, ASPN, GCNT2, TBX18, HES1, FN1, DUSP1, IFI6, PER2, PTPRA, POSTN, YWHAQ, SULF1, SMAD6, PRICKLE1, DUSP6, CDH11, SIK1, SMYD2, IGFBP3, ADM, BCL2L11, NR4A2, PRKCA, UCHL1, FZD7, RAB11FIP1, DGKI, LRP5, HTRA1, TERF2IP, ROBO1, PDGFD, F2R, ABAT, GRIN2A, ROR1, SPRY4, LBH, SIK1B
Regulation of signaling	46	0,0000	LTBP1, CTTN, NOX4, PIK3IP1, DOK5, SMAD7, SERPINE1, ASPN, GCNT2, TBX18, HES1, FN1, DUSP1, IFI6, PER2, PTPRA, POSTN, YWHAQ, SULF1, SMAD6, PRICKLE1, DUSP6, CDH11, SIK1, SMYD2, IGFBP3, ADM, BCL2L11, NR4A2, PRKCA, UCHL1, FZD7, RAB11FIP1, DGKI, LRP5, HTRA1, TERF2IP, ROBO1, PDGFD, F2R, ABAT, GRIN2A, ROR1, SPRY4, LBH, SIK1B

Cellular protein modification process	45	0,0017	TNC, EPHA3, CP, LTBP1, ULK2, NOX4, DOK5, SMAD7, USP11, CCND1, CDKN1B, GCNT2, HES1, EFEMP1, FN1, DUSP1, HSPA2, PER2, PTPRA, SMAD6, PRICKLE1, DUSP6, SIK1, SMYD2, IGFBP3, PRSS23, PRKCA, UCHL1, FZD7, LRP5, MXRA8, IGFBP7, TERF2IP, ROBO1, PDGFD, F2R, UBA7, EXT1, BGN, ROR1, SPRY4, PLXNB2, DDR1, LBH, SIK1B
Cell proliferation	44	0,0000	CD9, BTN3A1, GAB2, TNC, NOX4, PTGS1, CCND1, CDKN1B, GCNT2, NPR3, HES1, FN1, ID2, ODC1, LEPR, ETV3, DUSP1, BHLHE41, ALDH1A2, PER2, MYH10, SULF1, SMAD6, DPT, SMYD2, COL8A1, IGFBP3, ADM, PRKCA, UCHL1, FZD7, LRP5, IGFBP7, HTRA1, ROBO1, PDGFD, ID4, F2R, PLXNB2, DDR1, LBH, HLA-DPB1, HLA-DPA1, ETV5
Tissue development	43	0,0000	CD9, TNC, EPHA3, ITGB5, NOX4, SMAD7, SERPINE1, ASPN, HLF, CCND1, CDKN1B, GCNT2, TBX18, HES1, EFEMP1, FN1, ID2, ID3, COL21A1, OMD, ALDH1A2, MYH10, POSTN, MICAL2, SULF1, SMAD6, FRAS1, PRICKLE1, LUM, SIK1, COL8A1, ADM, FZD7, LRP5, ROBO1, PDGFD, ID4, EXT1, ROR1, PLXNB2, DDR1, LBH, ETV5
Regulation of signal transduction	41	0,0001	LTBP1, CTTN, NOX4, PIK3IP1, DOK5, SMAD7, SERPINE1, ASPN, GCNT2, TBX18, HES1, FN1, DUSP1, IFI6, POSTN, YWHAQ, SULF1, SMAD6, PRICKLE1, DUSP6, SIK1, SMYD2, IGFBP3, ADM, BCL2L11, NR4A2, PRKCA, UCHL1, FZD7, DGKI, HTRA1, TERF2IP, ROBO1, PDGFD, F2R, ABAT, GRIN2A, ROR1, SPRY4, LBH, SIK1B

Response to external stimulus	40	0,0000	CFH, CD9, GAB2, TNC, EPHA3, PER3, ULK2, DOK5, SERPINE1, CCND1, CDKN1B, ID2, ODC1, DUSP1, IFI6, ALDH1A2, PER2, PTPRA, MYH10, POSTN, SMAD6, SIK1, ADM, MR1, BCL2L11, NR4A2, PRKCA, ENAH, XPC, HTRA1, ROBO1, PDGFD, F2R, EXT1, ABAT, GRIN2A, PROS1, PLXNB2, HMGN2, SIK1B
Multicellular organism development	39	0,0000	TMEM98, CD9, GAB2, TNC, EPHA3, LTBP1, ITGB5, ULK2, CTTN, NOX4, DOK5, SMAD7, SERPINE1, ASPN, HLF, CCND1, CDKN1B, GCNT2, TBX18, NPR3, HES1, EFEMP1, FN1, ID2, ODC1, LEPR, ID3, BHLHE41, ATP7B, COL21A1, OMD, ALDH1A2, DNAJB1, PER2, PTPRA, MYH10, POSTN, MICAL2, YWHAQ, SULF1, SMAD6, FRAS1, PRICKLE1, DUSP6, LUM, CDH11, SIK1, SMYD2, CSRNP1, COL8A1, ADM, BCL2L11, NR4A2, PRKCA, UCHL1, ENAH, FZD7", "FMNL3, LRP5, MXRA8, IGFBP7, HTRA1, ANTXR1, ROBO1, PDGFD, GATM, ID4, MYO1D, MTURN, EXT1, ABAT, GRIN2A, ROR1, SPRY4, PLXNB2, DDR1, LBH, ETV5
Regulation of RNA metabolic process	39	0,0060	PER3, SMAD7, SERPINE1, HLF, CCND1, CDKN1B, TBX18, HES1, EFEMP1, ID2, ETV3, ID3, SMAD9, BHLHE41, DNAJB1, PER2, MICAL2, YWHAQ, SULF1, SMAD6, PRICKLE1, LUM, SIK1, SMYD2, CSRNP1, NR4A2, PRKCA, FZD7, LRP5, ZNF704, HTRA1, TERF2IP, TEF, ID4, F2R, ROR1, ZNF676, LBH, ETV5, SRSF8

Regulation of RNA biosynthetic process	38	0,0059	PER3, SMAD7, SERPINE1, HLF, CCND1, CDKN1B, TBX18, HES1, EFEMP1, ID2, ETV3, ID3, SMAD9, BHLHE41, DNAJB1, PER2, MICAL2, YWHAQ, SULF1, SMAD6, PRICKLE1, LUM, SIK1, SMYD2, CSRNP1, NR4A2, FZD7, LRP5, ZNF704, HTRA1, TERF2IP, TEF, ID4, F2R, ROR1, ZNF676, LBH, ETV5
Regulation of transcription, DNA-templated	38	0,0042	PER3, SMAD7, SERPINE1, HLF, CCND1, CDKN1B, TBX18, HES1, EFEMP1, ID2, ETV3, ID3, SMAD9, BHLHE41, DNAJB1, PER2, MICAL2, YWHAQ, SULF1, SMAD6, PRICKLE1, LUM, SIK1, SMYD2, CSRNP1, NR4A2, FZD7, LRP5, ZNF704, HTRA1, TERF2IP, TEF, ID4, F2R, ROR1, ZNF676, LBH, ETV5
RNA biosynthetic process	38	0,0119	PER3, SMAD7, SERPINE1, HLF, CCND1, CDKN1B, TBX18, HES1, EFEMP1, ID2, ETV3, ID3, SMAD9, BHLHE41, DNAJB1, PER2, MICAL2, YWHAQ, SULF1, SMAD6, PRICKLE1, LUM, SIK1, SMYD2, CSRNP1, NR4A2, FZD7, LRP5, ZNF704, HTRA1, TERF2IP, TEF, ID4, F2R, ROR1, ZNF676, LBH, ETV5
Transcription, DNA-templated	38	0,0090	PER3, SMAD7, SERPINE1, HLF, CCND1, CDKN1B, TBX18, HES1, EFEMP1, ID2, ETV3, ID3, SMAD9, BHLHE41, DNAJB1, PER2, MICAL2, YWHAQ, SULF1, SMAD6, PRICKLE1, LUM, SIK1, SMYD2, CSRNP1, NR4A2, FZD7, LRP5, ZNF704, HTRA1, TERF2IP, TEF, ID4, F2R, ROR1, ZNF676, LBH, ETV5

Regulation of cell proliferation	37	0,0000	CD9, GAB2, TNC, NOX4, PTGS1, CCND1, CDKN1B, GCNT2, NPR3, HES1, FN1, ID2, ODC1, ETV3, DUSP1, ALDH1A2, PER2, SULF1, SMAD6, DPT, SMYD2, IGFBP3, ADM, PRKCA, FZD7, LRP5, IGFBP7, HTRA1, ROBO1, PDGFD, ID4, F2R, DDR1, LBH, HLA-DPB1, HLA-DPA1, ETV5
Cell adhesion	34	0,0000	CD9, TNC, EPHA3, ITGB5, COL16A1, CTTN, SMAD7, SERPINE1, ECM2, GCNT2, HES1, FN1, DUSP1, OMD, MYH10, POSTN, SMAD6, CDH11, DPT, COL8A1, DOCK1, BCL2L11, PRKCA, FZD7, MXRA8, IGFBP7, ANTXR1, ROBO1, ABAT, PLXNB2, DDR1, HLA-DPB1, HLA-DPA1, HBB
Intracellular signal transduction	34	0,0027	GAB2, NOX4, PIK3IP1, DOK5, CDKN1B, GCNT2, NPR3, HES1, FN1, DUSP1, IFI6, PTPRA, YWHAQ, DUSP6, SIK1, SMYD2, ZNF385B, IGFBP3, ADM, DOCK1, BCL2L11, PRKCA, UCHL1, FZD7, DGKI, TERF2IP, ROBO1, PDGFD, F2R, GRIN2A, ROR1, SPRY4, LBH, SIK1B
Immune system process	31	0,0421	CFH, TMEM98, CD9, BTN3A1, GAB2, ITGB5, SMAD7, SERPINE1, HES1, FN1, ID2, LEPR, DUSP1, IFI6, SMAD6, SEC31A, DOCK1, MR1, BCL2L11, PRKCA, FZD7, LRP5, HTRA1, PDGFD, PROS1, HLA-DRB1, HLA-DQA1, HMGN2, HLA-DPB1, HLA-DPA1, HBB
Response to endogenous stimulus	31	0,0000	CD9, TNC, LTBP1, ITGB5, COL16A1, NOX4, SMAD7, MXRA5, ASPN, CCND1, CDKN1B, GCNT2, HES1, LEPR, LTBP2, DUSP1, SMAD9, FMOD, ALDH1A2, PTPRA, POSTN, SULF1, SMAD6, ADM, BCL2L11, NR4A2, IGFBP7, HTRA1, PDGFD, SPRY4, LBH

Protein localization	30	0,0116	COPZ2, TMEM98, BTN3A1, EPHA3, LTBP1, CTTN, KDELR3, SMAD7, FN1, LTBP2, COPA, PER2, MYH10, POSTN, YWHAQ, KIF13A, SEC31A, FRAS1, PRICKLE1, BCL2L11, PRKCA, RAB11FIP1, LRP5, TERF2IP, ID4, MYO1D, F2R, ABAT, GRIN2A, ETV5
Cell death	28	0,0025	CTTN, NOX4, SERPINE1, CDKN1B, ID3, DUSP1, IFI6, ALDH1A2, YWHAQ, SULF1, SMAD6, PHLDA1, DUSP6, SIK1, ZNF385B, CSRNP1, IGFBP3, ADM, DOCK1, BCL2L11, NR4A2, PRKCA, ROBO1, F2R, GRIN2A, HBA2, HBA1, HBB
Circulatory system development	28	0,0000	LTBP1, NOX4, SMAD7, SERPINE1, CDKN1B, HES1, FN1, ID2, LEPR, ID3, ALDH1A2, MYH10, MICAL2, SULF1, SMAD6, PRICKLE1, DUSP6, SIK1, SMYD2, COL8A1, ADM, PRKCA, FZD7, FMNL3, LRP5, ANTXR1, ROBO1, PDGFD
Regulation of transcription by RNA polymerase II	28	0,0236	PER3, SMAD7, SERPINE1, HLF, CCND1, TBX18, HES1, ID2, ETV3, ID3, SMAD9, BHLHE41, DNAJB1, PER2, MICAL2, SULF1, SMAD6, LUM, SMYD2, CSRNP1, NR4A2, LRP5, ZNF704, HTRA1, TEF, ID4, ZNF676, ETV5
Transcription by RNA polymerase II	28	0,0413	PER3, SMAD7, SERPINE1, HLF, CCND1, TBX18, HES1, ID2, ETV3, ID3, SMAD9, BHLHE41, DNAJB1, PER2, MICAL2, SULF1, SMAD6, LUM, SMYD2, CSRNP1, NR4A2, LRP5, ZNF704, HTRA1, TEF, ID4, ZNF676, ETV5

Cell morphogenesis	26	0,0000	GAB2, EPHA3, ULK2, CTTN, NOX4, DOK5, HES1, FN1, ID2, COL21A1, PTPRA, MYH10, POSTN, CDH11, DOCK1, NR4A2, PRKCA, UCHL1, ENAH, FZD7, FMNL3, ANTXR1, ROBO1, EXT1, PLXNB2, DDR1
Apoptotic process	25	0,0036	CTTN, NOX4, SERPINE1, CDKN1B, ID3, DUSP1, IFI6, ALDH1A2, YWHAQ, SULF1, SMAD6, PHLDA1, DUSP6, SIK1, ZNF385B, CSRNP1, IGFBP3, ADM, DOCK1, BCL2L11, NR4A2, PRKCA, ROBO1, F2R, GRIN2A
Epithelium development	25	0,0000	TNC, SMAD7, SERPINE1, CCND1, CDKN1B, TBX18, HES1, ID2, ID3, ALDH1A2, MICAL2, SULF1, SMAD6, FRAS1, PRICKLE1, ADM, FZD7, LRP5, ROBO1, ID4, ROR1, PLXNB2, DDR1, LBH, ETV5
Programmed cell death	25	0,0086	CTTN, NOX4, SERPINE1, CDKN1B, ID3, DUSP1, IFI6, ALDH1A2, YWHAQ, SULF1, SMAD6, PHLDA1, DUSP6, SIK1, ZNF385B, CSRNP1, IGFBP3, ADM, DOCK1, BCL2L11, NR4A2, PRKCA, ROBO1, F2R, GRIN2A
Regulation of protein phosphorylation	24	0,0001	NOX4, DOK5, SMAD7, CCND1, CDKN1B, GCNT2, HES1, FN1, DUSP1, HSPA2, SMAD6, DUSP6, IGFBP3, PRKCA, UCHL1, FZD7, LRP5, TERF2IP, ROBO1, PDGFD, F2R, SPRY4, PLXNB2, LBH
Vesicle-mediated transport	24	0,0140	COPZ2, CD9, GAB2, EPHA3, CTTN, KDELR3, SERPINE1, FN1, LEPR, COPA, HSPA2, MYH10, KIF13A, SEC31A, ADM, DOCK1, RAB11FIP1, DGKI, LRP5, MYO1D, PROS1, HBA2, HBA1, HBB

Regulation of cell death	23	0,0027	CTTN, NOX4, SERPINE1, CDKN1B, ID3, DUSP1, IFI6, ALDH1A2, YWHAQ, SMAD6, DUSP6, SIK1, IGFBP3, ADM, BCL2L11, NR4A2, PRKCA, ROBO1, F2R, GRIN2A, HBA2, HBA1, HBB
Protein transport	21	0,0454	COPZ2, BTN3A1, CTTN, KDELR3, FN1, LTBP2, COPA, PER2, MYH10, POSTN, YWHAQ, KIF13A, SEC31A, FRAS1, PRICKLE1, PRKCA, RAB11FIP1, LRP5, MYO1D, F2R, ABAT
Regulation of apoptotic process	20	0,0090	CTTN, NOX4, SERPINE1, CDKN1B, ID3, DUSP1, IFI6, ALDH1A2, YWHAQ, SMAD6, DUSP6, SIK1, IGFBP3, ADM, BCL2L11, NR4A2, PRKCA, ROBO1, F2R, GRIN2A
Regulation of programmed cell death	20	0,0106	CTTN, NOX4, SERPINE1, CDKN1B, ID3, DUSP1, IFI6, ALDH1A2, YWHAQ, SMAD6, DUSP6, SIK1, IGFBP3, ADM, BCL2L11, NR4A2, PRKCA, ROBO1, F2R, GRIN2A
Smooth muscle cell regulation	20	0,0003	CD9, ITGB5, SERPINE1, TBX18, HES1, FN1, LEPR, ALDH1A2, MYH10, SULF1, PRICKLE1, COL8A1, ADM, BCL2L11, PRKCA, FZD7, FMNL3, HTRA1, ROBO1, PLXNB2
Blood vessel development	19	0,0000	LTBP1, SMAD7, SERPINE1, HES1, FN1, LEPR, ALDH1A2, MYH10, SULF1, SMAD6, PRICKLE1, COL8A1, ADM, PRKCA, FMNL3, LRP5, ANTXR1, ROBO1, PDGFD
Vasculature development	19	0,0000	LTBP1, SMAD7, SERPINE1, HES1, FN1, LEPR, ALDH1A2, MYH10, SULF1, SMAD6, PRICKLE1, COL8A1, ADM, PRKCA, FMNL3, LRP5, ANTXR1, ROBO1, PDGFD
Heart development	17	0,0000	LTBP1, NOX4, SMAD7, CDKN1B, HES1, ID2, ID3, ALDH1A2, MYH10, MICAL2, SMAD6, PRICKLE1, DUSP6, SIK1, SMYD2, ADM, FZD7, ROBO1

Extracellular matrix organization	16	0,0000	TNC, ITGB5, COL16A1, SERPINE1, ECM2, FN1, FMOD, POSTN, SULF1, LUM, DPT, COL8A1, HTRA1, ANTXR1, BGN, DDR1
MAPK activity	16	0,0013	NOX4, DOK5, GCNT2, FN1, DUSP1, PTPRA, DUSP6, IGFBP3, PRKCA, UCHL1, FZD7, ROBO1, PDGFD, F2R, SPRY4, LBH
Muscle structure development	14	0,0003	CD9, NOX4, SMAD7, HLF, HES1, ID3, BHLHE41, MYH10, PRICKLE1, SIK1, IGFBP3, ADM, UCHL1, FZD7
Regulation of transcription by RNA polymerase II	14	0,0027	PER3, SMAD7, CCND1, TBX18, HES1, ID2, ETV3, ID3, BHLHE41, DNAJB1, PER2, SMYD2, NR4A2, ID4
Cytokine production	13	0,0043	BTN3A1, LTBP1, SMAD7, SERPINE1, FN1, POSTN, SULF1, LUM, MR1, F2R, UBA7, HLA-DPB1, HLA-DPA1
Mitotic cell cycle	13	0,0299	CCND1, CDKN1B, HES1, ID2, DUSP1, HSPA2, MYH10, PHLDA1, SIK1, PRKCA, XPC, LRP5, ID4
Response to hormone	13	0,0242	TNC, CCND1, CDKN1B, LEPR, DUSP1, ALDH1A2, PTPRA, POSTN, ADM, BCL2L11, NR4A2, IGFBP7, LBH
Blood vessel morphogenesis	12	0,003535	SMAD7, SERPINE1, HES1, FN1, LEPR, SULF1, COL8A1, ADM, PRKCA, FMNL3, LRP5, ROBO1
Circulatory system process	12	0,0006	NOX4, PTGS1, SMAD7, NPR3, ID2, PER2, POSTN, ADM, LRP5, F2R, ABAT, HBB
Blood circulation	11	0,0017	PTGS1, SMAD7, NPR3, ID2, PER2, POSTN, ADM, LRP5, F2R, ABAT, HBB
Response to oxidative stress	10	0,0015	NOX4, PTGS1, DUSP1, NR4A2, GPX8, PDGFD, HBA2, HBA1, ETV5, HBB
Angiogenesis	9	0,0249	SERPINE1, FN1, LEPR, SULF1, COL8A1, ADM, PRKCA, FMNL3, ROBO1
Blood coagulation	9	0,0007	CD9, SERPINE1, DOCK1, PRKCA, DGKI, F2R, ABAT, PROS1, HBB

Hemostasis	9	0,0008	CD9, SERPINE1, DOCK1, PRKCA, DGKI, F2R, ABAT, PROS1, HBB
Regulation of blood pressure	9	0,0000	PTGS1, NPR3, ID2, POSTN, ADM, LRP5, F2R, ABAT, HBB
Response to extracellular stimulus	9	0,0140	TNC, ULK2, CCND1, ALDH1A2, POSTN, SIK1, ADM, NR4A2, SIK1B
Cardiac chamber development	8	0,0002	LTBP1, SMAD7, HES1, ID2, MYH10, SMAD6, ROBO1
Muscle system process	8	0,0185	ITGB5, CTTN, SMAD7, SULF1, PRKCA, GATM, F2R, ABAT
Muscle tissue development	8	0,0090	NOX4, SMAD7, HLF, ID2, ALDH1A2, MYH10, SIK1, FZD7
Striated muscle tissue development	8	0,0069	NOX4, SMAD7, HLF, ID2, ALDH1A2, MYH10, SIK1, FZD7
Artery development	7	0,0000	LTBP1, SMAD7, HES1, MYH10, SMAD6, PRICKLE1, ROBO1
Cardiac muscle tissue development	7	0,0014	NOX4, SMAD7, ID2, ALDH1A2, MYH10, SIK1, FZD7
Cardiac septum development	7	0,0000	LTBP1, SMAD7, HES1, ID2, MYH10, SMAD6, ROBO1
Heart morphogenesis	7	0,0026	SMAD7, HES1, ID2, ALDH1A2, MICAL2, SMAD6, ROBO1
Regulation of cell growth	7	0,0297	ULK2, CTTN, CDKN1B, FN1, IGFBP3, IGFBP7, DDR1
Cardiac ventricle development	6	0,0003	LTBP1, SMAD7, HES1, ID2, SMAD6, ROBO1
Muscle cell migration	6	0,0001	NOX4, SERPINE1, POSTN, IGFBP3, PDGFD, DDR1
Muscle contraction	6	0,0444	ITGB5, CTTN, SMAD7, SULF1, F2R, ABAT
Platelet activation	6	0,0008	CD9, PRKCA, DGKI, F2R, ABAT, HBB
Regulation of blood coagulation	6	0,0000	CD9, SERPINE1, PRKCA, F2R, ABAT, PROS1
Response to hypoxia	6	0,0439	NOX4, CDKN1B, POSTN, ADM, NR4A2, ABAT
Ventricular septum development	6	0,0000	LTBP1, SMAD7, HES1, ID2, SMAD6, ROBO1
Response to ROS	5	0,0255	DUSP1, PDGFD, HBA2, HBA1, HBB
Temperature homeostasis	5	0,0080	NPR3, LEPR, PER2, GATM, ABAT

Coronary vasculature development	4	0,0004	LTBP1, MYH10, SMAD6, PRICKLE1
Regulation of muscle contraction	4	0,0342	CTTN, SMAD7, F2R, ABAT
Smooth muscle contraction	4	0,0080	CTTN, SULF1, F2R, ABAT
Vascular process in circulatory system	4	0,0354	PER2, ADM, F2R, HBB
Neuromuscular junction development	3	0,0048	TNC, F2R, ETV5
Regulation of platelet activation	3	0,0014	CD9, PRKCA, ABAT
Regulation of smooth muscle contraction	3	0,0112	CTTN, F2R, ABAT
Regulation of systemic arterial blood pressure	3	0,0289	POSTN, ADM, F2R
Aortic valve development	2	0,0218	SMAD6, ROBO1
Aortic valve morphogenesis	2	0,0169	SMAD6, ROBO1
Heart formation	2	0,0147	HES1, ROBO1
Pulmonary valve development	2	0,0097	SMAD6, ROBO1
Pulmonary valve morphogenesis	2	0,0064	SMAD6, ROBO1
Regulation of membrane depolarization	2	0,0477	SMAD7, IFI6

Table S4. Gene Ontology Molecular Function for over-expressed genes

Molecular Function	DEG number	P DE	Gene name
Molecular Function	138	0,0012	CFH, CD9, BTN3A1, GAB2, TNC, EPHA3, CP, PER3, LTBP1, ALDH18A1, ITGB5, ULK2, COL16A1, CTTN, NOX4, PTGS1, PIK3IP1, KDELR3, SMAD7, MXRA5, USP11, SERPINE1, ASPN, ECM2, HLF, CCND1, CDKN1B, GCNT2, TBX18, NPR3, HES1, EFEMP1, FN1, ID2, ODC1, LEPR, ETV3, ID3, DNAL1, LTBP2, DUSP1, PLXDC2, SMAD9, FMOD, COPA, BHLHE41, ATP7B, COL21A1, IFI6, HSPA2, ALDH1A2, DCTD, DNAJB1, PER2, PTPRA, MYH10, POSTN", "MICAL2, YWHAQ, CYP27A1, KIF13A, SULF1, SMAD6, SEC31A, FRAS1, SLC39A8, PRICKLE1, PHLDA1, DUSP6, LUM, CDR2, CDH11, SIK1, DPT, SMYD2, ZNF385B, CSRNP1, COL8A1, IGFBP3, ADM, TMCO3, PRSS23, DOCK1, SLC16A12, MR1, BCL2L11, NR4A2, PRKCA, UCHL1, ENAH, XPC, FZD7, RAB11FIP1, DGKI, FMNL3, LRP5, MXRA8, KCNT2, IGFBP7, GPX8, ZNF704, HTRA1, TERF2IP, TEF, ANTXR1, ROBO1, PDGFD, VAT1L, GATM, ID4, MYO1D, F2R", "UBA7, EXT1, BGN, ABAT, GRIN2A, PROS1, ROR1, SPRY4, HBA2, ZNF676, HLA-DRB1, PLXNB2, HLA-DQA1, FAM114A1, HMGN2, DDR1, HBA1, LBH, HLA-DPB1, HLA-DPA1, INMT, ETV5, HBB, SRSF8, SIK1B, HYDIN2,

Binding	126	0,0004	CFH, CD9, BTN3A1, GAB2, TNC, EPHA3, CP, PER3, LTBP1, ALDH18A1, ITGB5, ULK2, COL16A1, CTTN, NOX4, PTGS1, PIK3IP1, KDELR3, SMAD7, USP11, SERPINE1, ASPN, ECM2, HLF, CCND1, CDKN1B, TBX18, NPR3, HES1, EFEMP1, FN1, ID2, ODC1, LEPR, ETV3, ID3, DNAL1, LTBP2, DUSP1, PLXDC2, SMAD9, COPA, BHLHE41, ATP7B, IFI6, HSPA2, ALDH1A2, DCTD, DNAJB1, PER2, PTPRA, MYH10, POSTN, MICAL2, YWHAQ, CYP27A1, KIF13A", "SULF1, SMAD6, SEC31A, FRAS1, PRICKLE1, PHLDA1, LUM, CDR2, CDH11, SIK1, DPT, SMYD2, ZNF385B, CSRNP1, COL8A1, IGFBP3, ADM, DOCK1, MR1, BCL2L11, NR4A2, PRKCA, UCHL1, ENAH, XPC, FZD7, RAB11FIP1, DGKI, FMNL3, LRP5, KCNT2, IGFBP7, ZNF704, HTRA1, TERF2IP, TEF, ANTXR1, ROBO1, PDGFD, VAT1L, ID4, MYO1D, F2R, UBA7, EXT1, BGN, ABAT, GRIN2A, PROS1, ROR1, SPRY4, HBA2, ZNF676, HLA-DRB1, PLXNB2, HLA-DQA1", "FAM114A1, HMGN2, DDR1, HBA1, LBH, HLA-DPB1, HLA-DPA1, INMT, ETV5, HBB, SRSF8, SIK1B, HYDIN2,

Protein binding	110	0,0000	CFH, CD9, BTN3A1, GAB2, TNC, EPHA3, CP, PER3, LTBP1, ALDH18A1, ITGB5, ULK2, COL16A1, CTTN, NOX4, PIK3IP1, SMAD7, USP11, SERPINE1, ECM2, CCND1, CDKN1B, TBX18, NPR3, HES1, EFEMP1, FN1, ID2, ODC1, LEPR, ETV3, ID3, DNAL1, LTBP2, DUSP1, PLXDC2, SMAD9, COPA, BHLHE41, ATP7B, IFI6, HSPA2, DCTD, DNAJB1, PER2, PTPRA, MYH10, POSTN, MICAL2, YWHAQ, KIF13A, SMAD6, SEC31A, PRICKLE1, PHLDA1, LUM, CDR2", "CDH11, SIK1, DPT, SMYD2, ZNF385B, CSRNP1, COL8A1, IGFBP3, ADM, DOCK1, MR1, BCL2L11, NR4A2, PRKCA, UCHL1, ENAH, XPC, FZD7, RAB11FIP1, DGKI, FMNL3, LRP5, IGFBP7, HTRA1, TERF2IP, TEF, ANTXR1, ROBO1, PDGFD, VAT1L, ID4, MYO1D, F2R, UBA7, EXT1, BGN, ABAT, GRIN2A, ROR1, SPRY4, HBA2, PLXNB2, HLA-DQA1, FAM114A1, HMGN2, DDR1, HBA1, LBH, INMT, ETV5, HBB, SRSF8,
Ion binding	56	0,0232	CFH, GAB2, EPHA3, CP, LTBP1, ALDH18A1, ULK2, NOX4, PTGS1, SMAD7, ASPN, ECM2, NPR3, EFEMP1, FN1, ID3, LTBP2, SMAD9, ATP7B, HSPA2, DCTD, MYH10, POSTN, MICAL2, CYP27A1, KIF13A, SULF1, SMAD6, FRAS1, PRICKLE1, CDH11, SIK1, SMYD2, ZNF385B, IGFBP3, NR4A2, PRKCA, FZD7, DGKI, KCNT2, ZNF704, ANTXR1, VAT1L, MYO1D, UBA7, EXT1, ABAT, GRIN2A, PROS1, ROR1, HBA2, ZNF676, DDR1, HBA1, HBB, SIK1B,

Enzyme binding	28	0,0019	GAB2, PER3, NOX4, PIK3IP1, SMAD7, SERPINE1, CCND1, CDKN1B, HES1, FN1, ETV3, DUSP1, BHLHE41, HSPA2, DNAJB1, SMAD6, SIK1, SMYD2, DOCK1, BCL2L11, PRKCA, UCHL1, RAB11FIP1, DGKI, FMNL3, TERF2IP, ABAT,
Anion binding	28	0,0345	CFH, GAB2, EPHA3, ALDH18A1, ULK2, NOX4, ECM2, NPR3, FN1, ID3, LTBP2, ATP7B, HSPA2, MYH10, POSTN, MICAL2, KIF13A, SIK1, PRKCA, FZD7, DGKI, KCNT2, MYO1D, UBA7, ABAT, ROR1, DDR1, SIK1B,
Molecular transducer activity	24	0,0002	EPHA3, LTBP1, ITGB5, SMAD7, CDKN1B, NPR3, EFEMP1, LEPR, SMAD9, PTPRA, SMAD6, MR1, NR4A2, FZD7, LRP5, ANTXR1, ROBO1, F2R, GRIN2A, ROR1, PLXNB2, HLA-DQA1, DDR1, HLA-DPA1,
Carbohydrate derivative binding	24	0,0247	CFH, TNC, EPHA3, ALDH18A1, ULK2, ECM2, FN1, LTBP2, ATP7B, HSPA2, MYH10, POSTN, KIF13A, SULF1, SIK1, PRKCA, DGKI, KCNT2, MYO1D, UBA7, BGN, ROR1, DDR1, SIK1B,
Signaling receptor activity	20	0,0044	EPHA3, LTBP1, ITGB5, NPR3, EFEMP1, LEPR, PTPRA, MR1, NR4A2, FZD7, LRP5, ANTXR1, ROBO1, F2R, GRIN2A, ROR1, PLXNB2, HLA-DQA1, DDR1, HLA-DPA1,
Signaling receptor binding	19	0,0221	CD9, BTN3A1, GAB2, ITGB5, COL16A1, SMAD7, SERPINE1, ECM2, EFEMP1, FN1, COPA, SMAD6, ADM, NR4A2, PRKCA, UCHL1, FZD7, PDGFD, F2R,
Structural molecule activity	19	0,0000	TNC, LTBP1, COL16A1, MXRA5, ASPN, EFEMP1, FN1, LTBP2, FMOD, COPA, COL21A1, POSTN, SEC31A, FRAS1, LUM, DPT, COL8A1, IGFBP7, BGN,

Protein-containing complex binding	19	0,0005	CD9, ITGB5, COL16A1, CTTN, SMAD7, ASPN, ECM2, CCND1, CDKN1B, FN1, MYH10, LUM, PRKCA, XPC, ANTXR1, MYO1D, HLA-DRB1, HMGN2, DDR1,
Transmembrane signaling receptor activity	18	0,0043	EPHA3, LTBP1, NPR3, EFEMP1, LEPR, PTPRA, MR1, FZD7, LRP5, ANTXR1, ROBO1, F2R, GRIN2A, ROR1, PLXNB2, HLA-DQA1, DDR1, HLA-DPA1,
Extracellular matrix structural constituent	17	0,0000	TNC, LTBP1, COL16A1, MXRA5, ASPN, EFEMP1, FN1, LTBP2, FMOD, COL21A1, POSTN, FRAS1, LUM, DPT, COL8A1, IGFBP7, BGN,
Sequence-specific DNA binding	14	0,0329	PER3, HLF, TBX18, HES1, ETV3, BHLHE41, PER2, SMAD6, CSRNP1, NR4A2, ZNF704, TERF2IP, TEF, ETV5,
Transcription regulatory region sequence-specific DNA binding	13	0,0170	PER3, HLF, TBX18, HES1, ETV3, BHLHE41, PER2, SMAD6, SMAD7, NR4A2, ZNF704, TEF, ETV5,
Transcription regulatory region DNA binding	13	0,0189	PER3, SMAD7, HLF, TBX18, HES1, ETV3, BHLHE41, PER2, SMAD6, NR4A2, ZNF704, TEF, ETV5,
Cytoskeletal protein binding	12	0,0467	CTTN, DNAL1, HSPA2, MYH10, MICAL2, KIF13A, CDH11, BCL2L11, ENAH, FMNL3, ANTXR1, MYO1D,
Oxidoreductase activity	12	0,0073	CP, ALDH18A1, NOX4, PTGS1, ALDH1A2, MICAL2, CYP27A1, GPX8, VAT1L, HBA2, HBA1, HBB,
Sequence-specific double-stranded DNA binding	12	0,0253	PER3, HLF, TBX18, HES1, ETV3, BHLHE41, PER2, SMAD6, NR4A2, ZNF704, TEF, ETV5,
Transcription factor binding	11	0,0078	PER3, CCND1, TBX18, HES1, ID2, ID3, BHLHE41, PER2, SIK1, NR4A2, ID4,
Kinase activity	11	0,0206	EPHA3, LTBP1, ALDH18A1, ULK2, CCND1, EFEMP1, SIK1, PRKCA, DGKI, DDR1, SIK1B,
Phosphotransferase activity, alcohol group as acceptor	10	0,0273	EPHA3, LTBP1, ULK2, CCND1, EFEMP1, SIK1, PRKCA, DGKI, DDR1, SIK1B,

Kinase binding	10	0,0363	GAB2, PER3, NOX4, PIK3IP1, CCND1, CDKN1B, DUSP1, SIK1, BCL2L11,
Cell adhesion molecule binding	10	0,0031	CD9, ITGB5, COL16A1, CTTN, ECM2, FN1, DNAJB1, POSTN, CDH11, PRKCA,
Protein kinase activity	9	0,0261	EPHA3, LTBP1, ULK2, CCND1, EFEMP1, SIK1, PRKCA, DDR1, SIK1B,
Amide binding	8	0,0041	KDELR3, NPR3, LEPR, GRIN2A, HLA-DRB1, HLA-DQA1, HLA-DPB1, HLA-DPA1,
Peptide binding	8	0,0013	KDELR3, NPR3, LEPR, GRIN2A, HLA-DRB1, HLA-DQA1, HLA-DPB1, HLA-DPA1,
Cofactor binding	8	0,0287	NOX4, PTGS1, MICAL2, CYP27A1, ABAT, HBA2, HBA1, HBB,
Actin binding	7	0,0350	CTTN, MYH10, MICAL2, ENAH, FMNL3, ANTXR1, MYO1D,
Collagen binding	7	0,0000	SMAD7, ASPN, ECM2, FN1, LUM, ANTXR1, DDR1,
Integrin binding	6	0,0004	CD9, ITGB5, COL16A1, ECM2, FN1, PRKCA,
Growth factor binding	6	0,0005	LTBP1, LTBP2, DUSP1, IGFBP3, IGFBP7, HTRA1,
Heme binding	6	0,0004	NOX4, PTGS1, CYP27A1, HBA2, HBA1, HBB,
Chaperone binding	6	0,0001	CP, CDKN1B, HES1, FN1, HSPA2, DNAJB1,
RNA polymerase II transcription factor binding	5	0,0051	TBX18, ID2, ID3, BHLHE41, ID4,
Heparin binding	5	0,0073	CFH, ECM2, FN1, LTBP2, POSTN,
Antioxidant activity	5	0,0004	PTGS1, GPX8, HBA2, HBA1, HBB,
Sulfur compound binding	5	0,0336	CFH, ECM2, FN1, LTBP2, POSTN,
Motor activity	4	0,0164	DNAL1, MYH10, KIF13A, MYO1D,
Antigen binding	4	0,0277	HLA-DRB1, HLA-DQA1, HLA-DPB1, HLA-DPA1,
Transforming growth factor beta receptor, cytoplasmic mediator activity	4	0,0000	SMAD7, CDKN1B, SMAD9, SMAD6,

Extracellular matrix structural constituent conferring compression resistance	4	0,0000	ASPN, FMOD, LUM, BGN,
Peptide antigen binding	4	0,0001	HLA-DRB1, HLA-DQA1, HLA-DPB1, HLA-DPA1,
Transmembrane receptor protein tyrosine kinase activity	3	0,0099	EPHA3, EFEMP1, DDR1,
Extracellular matrix structural constituent conferring tensile strength	3	0,0031	COL16A1, COL21A1, COL8A1,
Haptoglobin binding	3	0,0000	HBA2, HBA1, HBB,
Wnt-activated receptor activity	3	0,0002	FZD7, LRP5, ROR1,
Histone deacetylase binding	3	0,0451	CCND1, HES1, BHLHE41,
Microfilament motor activity	2	0,0106	MYH10, MYO1D,
Actin-dependent ATPase activity	2	0,0032	MYH10, MYO1D,
MHC class II receptor activity	2	0,0022	HLA-DQA1, HLA-DPA1,
Type I transforming growth factor beta receptor binding	2	0,0027	SMAD7, SMAD6,
Microfibril binding	2	0,0002	LTBP1, LTBP2,

Table S5. Gene Ontology Biological Process for under-expressed genes

Signalling	DEG number	P DE	Gene name
Biological process	262	0,0015	LAP3, AK2, NDUFAB1, HCCS, SKAP2, AASS, HSF2, LRRC7, MYOC, DAPK2, C6, GATB, FAR2, NDUFB4, ME1, TBC1D1, MTHFD2, ISOC1, RRP15, PYGM, ADGRF5, TGFBR3, GNB5, CAMK2A, PVR, DHRS9, TIMM21, SLC1A3, KCNN2, AACS, CADPS2, ME2, EPB41L3, CD59, AKR1B1, SIGLEC1, MRPS33, NDUFB2, CD209, DESI1, FKBP3, PSMA3, PLTP, PSMD7, HSDL1, CRISPLD2, HMOX2, RASL12, PDGFRL, NCALD, SLC39A14, PDCD5, LILRB5, SSBP1, CHCHD3", "COA1, PLGRKT, ACTA2, ENO3, MRPL27, PMP22, ALDOC, NDUFC1, MTCH2, MRPL51, CHPT1, HDDC2, TPD52L1, MTRF1L, ACOT13, DUSP22, SLC27A6, PDE4D, RAD1, STC2, BCL6, CBLB, DNAJC27, CACYBP, RALGPS2, MRPL37, MRPS15, NID1, SDHB, UAP1, PRDX1, PRDX6, MARC2, B4GALT6, NDUFB3, KCNIP2, TEK, TGFBI, COPS5, GPSM2, MTIF3, GTF3A, PTGFR, PLAU, PLP1, F13A1, SERPINB6, EMC3, NDUFA1, MKKS, COX6B1, AIF1L, TNFRSF19, STEAP4", "MTX2, RTN4IP1, QRSL1, MPP1, COX7B, MRPS25, PDHA1, RAP1GAP2, COQ3, DCLK1, STARD13, TMTC1, ARNTL, LYVE1, CHL1, EMC7, TIMM17A, CTSL, TBX3, COQ10A, GLUL, MRPS9, CALCOCO2, PSMB7, PPIL1, RNF144B, MRPL15, ARL3, WDR12, COX17, CILP, ENPEP, EGF, FBN2, NDUFB10, PTRH2, C1ORF43, OSR1, MEIS1, MRPS5, PTPRG, SLC25A26, TBC1D7, FARS2, GFOD1, SH3KBP1, ITGB1BP2, NDUFB9, LRMDA, APIP, EI24, SPARCL1, RETREG1, ATP5MC3", "ATP5PF, GTF3C6, VSIG4, ADK, ATP5MPL, UQCRB, MTERF3, PTDSS1, MALSU1, SNX22, EDA, ADAMTS4, FCER1G, TOMM40L, ATP5MC1, SNRNP25, PLPP3, CHCHD4, SUCLG1, PPM1L, PITX2, HTR4, EGFLAM, FABP5, CTSB, COX6C, NDUFB6, SPTSSA, ATP5F1C, FUNDC2, CMTM5, GABRB3, AKTIP, MRPL58, ULK4, NT5DC2, FAM107A, NDUFS5, LRP1B, DNAJC21, PPIC, STXBP6, UQCRFS1, SCN9A, HINT1, REPS2, METTL7B, NFXL1, SPSB1, CHCHD1, LRRN3, STOX2, MICOS10", "ATP2A2, RSRC1, MSRA, TUBB6, CD163, ASB8, COX14, SELENOW, SLC36A4, IDH2, KPNA2, RGS6, UTP11, ZNF438, NDUFB1, UBE2F, NDUFA12, APOO, NDUFA6, UBE2L3, TRIM69, PPP1CC, NDUFA4, APOD, MFAP5, MYL4, LRRC10, CR1, RNF5, VIT, INSYN1, TMX2, S1PR3, FASTKD5, EBF2, MYBPHL, UQCRFS1P1, ATP5PO, MRPL33, TRMT9B, TRNP1, MRC1, TIMM23, TRAC, PSMB3

Cellular process	244	0,0016	AK2, NDUFAB1, HCCS, SKAP2, AASS, HSF2, LRRC7, MYOC, DAPK2, C6, GATB, FAR2, NDUFB4, ME1, TBC1D1, MTHFD2, RRP15, PYGM, ADGRF5, TGFBR3, GNB5, CAMK2A, PVR, DHRS9, TIMM21, SLC1A3, KCNN2, AACS, CADPS2, ME2, EPB41L3, CD59, AKR1B1, MRPS33, NDUFB2, CD209, FKBP3, PSMA3, PLTP, PSMD7, CRISPLD2, HMOX2, RASL12, PDGFRL, NCALD, SLC39A14, PDCD5, LILRB5, SSBP1, CHCHD3, COA1, PLGRKT, ACTA2, ENO3, MRPL27", "PMP22, ALDOC, NDUFC1, MTCH2, MRPL51, CHPT1, HDDC2, TPD52L1, MTRF1L, ACOT13, DUSP22, SLC27A6, PDE4D, RAD1, STC2, BCL6, CBLB, DNAJC27, CACYBP, RALGPS2, MRPL37, MRPS15, NID1, SDHB, UAP1, PRDX1, PRDX6, MARC2, B4GALT6, NDUFB3, KCNIP2, TEK, TGFBI, COPS5, GPSM2, MTIF3, GTF3A, PTGFR, PLAU, PLP1, F13A1, SERPINB6, EMC3, NDUFA1, MKKS, COX6B1, AIF1L, TNFRSF19, STEAP4, RTN4IP1, QRSL1, MPP1, COX7B, MRPS25", "PDHA1, RAP1GAP2, COQ3, DCLK1, STARD13, TMTC1, ARNTL, LYVE1, CHL1, TIMM17A, CTSL, TBX3, COQ10A, GLUL, MRPS9, CALCOCO2, PSMB7, PPIL1, RNF144B, MRPL15, ARL3, WDR12, COX17, CILP, ENPEP, EGF, FBN2, NDUFB10, PTRH2, OSR1, MEIS1, MRPS5, PTPRG, TBC1D7, FARS2, SH3KBP1, ITGB1BP2, NDUFB9, LRMDA, APIP, EI24, SPARCL1, RETREG1, ATP5MC3, ATP5PF, GTF3C6, VSIG4, ADK, UQCRB, MTERF3, PTDSS1, MALSU1, EDA, ADAMTS4", "FCER1G, TOMM40L, ATP5MC1, SNRNP25, PLPP3, CHCHD4, SUCLG1, PPM1L, PITX2, HTR4, EGFLAM, FABP5, CTSB, COX6C, NDUFB6, SPTSSA, ATP5F1C, FUNDC2, CMTM5, GABRB3, AKTIP, MRPL58, ULK4, NT5DC2, FAM107A, NDUFS5, DNAJC21, PPIC, STXBP6, UQCRFS1, SCN9A, HINT1, REPS2, NFXL1, SPSB1, CHCHD1, LRRN3, STOX2, ATP2A2, RSRC1, MSRA, TUBB6, ASB8, COX14, SELENOW, IDH2, KPNA2, RGS6, UTP11, ZNF438, NDUFB1, UBE2F, NDUFA12", "APOO, NDUFA6, UBE2L3, TRIM69, PPP1CC, NDUFA4, APOD, MFAP5, MYL4, LRRC10, CR1, RNF5, VIT, INSYN1, TMX2, S1PR3, FASTKD5, EBF2, UQCRFS1P1, ATP5PO, MRPL33, TRMT9B, TRNP1, MRC1, TIMM23, TRAC, PSMB3

Metabolic process	192	0,0010	LAP3, AK2, NDUFAB1, HCCS, AASS, HSF2, LRRC7, MYOC, DAPK2, C6, GATB, FAR2, NDUFB4, ME1, MTHFD2, RRP15, PYGM, ADGRF5, TGFBR3, CAMK2A, DHRS9, SLC1A3, AACS, ME2, CD59, AKR1B1, MRPS33, NDUFB2, DESI1, FKBP3, PSMA3, PLTP, PSMD7, HSDL1, HMOX2, PDCD5, SSBP1, CHCHD3, PLGRKT, ACTA2, ENO3, MRPL27, ALDOC, NDUFC1, MRPL51, CHPT1, HDDC2, TPD52L1, MTRF1L, ACOT13, DUSP22, SLC27A6, PDE4D, RAD1, STC2, BCL6", "CBLB, DNAJC27, CACYBP, MRPL37, MRPS15, SDHB, UAP1, PRDX1, PRDX6, MARC2, B4GALT6, NDUFB3, TEK, TGFBI, COPS5, MTIF3, GTF3A, PTGFR, PLAU, PLP1, F13A1, SERPINB6, NDUFA1, MKKS, COX6B1, TNFRSF19, STEAP4, RTN4IP1, QRSL1, MPP1, COX7B, MRPS25, PDHA1, COQ3, DCLK1, TMTC1, ARNTL, LYVE1, TIMM17A, CTSL, TBX3, COQ10A, GLUL, MRPS9, CALCOCO2, PSMB7, PPIL1, RNF144B, MRPL15, WDR12, COX17, CILP, ENPEP, EGF, NDUFB10", "PTRH2, OSR1, MEIS1, MRPS5, PTPRG, TBC1D7, FARS2, GFOD1, NDUFB9, APIP, EI24, SPARCL1, RETREG1, ATP5MC3, ATP5PF, GTF3C6, VSIG4, ADK, UQCRB, MTERF3, PTDSS1, MALSU1, EDA, ADAMTS4, FCER1G, ATP5MC1, SNRNP25, PLPP3, CHCHD4, SUCLG1, PPM1L, PITX2, EGFLAM, FABP5, CTSB, COX6C, NDUFB6, SPTSSA, ATP5F1C, FUNDC2, AKTIP, MRPL58, ULK4, NT5DC2, FAM107A, NDUFS5, PPIC, UQCRFS1, HINT1, METTL7B, NFXL1, SPSB1, CHCHD1", "STOX2, RSRC1, MSRA, ASB8, IDH2, KPNA2, UTP11, ZNF438, NDUFB1, UBE2F, NDUFA12, NDUFA6, UBE2L3, TRIM69, PPP1CC, NDUFA4, APOD, CR1, RNF5, FASTKD5, EBF2, UQCRFS1P1, ATP5PO, MRPL33, TRMT9B, PSMB3

Cellular metabolic process	181	0,0001	AK2, NDUFAB1, HCCS, AASS, HSF2, LRRC7, MYOC, DAPK2, C6, GATB, FAR2, NDUFB4, ME1, MTHFD2, RRP15, PYGM, ADGRF5, TGFBR3, CAMK2A, DHRS9, SLC1A3, AACS, ME2, CD59, AKR1B1, MRPS33, NDUFB2, FKBP3, PSMA3, PLTP, PSMD7, HMOX2, PDCD5, SSBP1, CHCHD3, PLGRKT, ACTA2, ENO3, MRPL27, ALDOC, NDUFC1, MRPL51, CHPT1, HDDC2, TPD52L1, MTRF1L, ACOT13, DUSP22, SLC27A6, PDE4D, RAD1, STC2, BCL6, CBLB, DNAJC27, CACYBP", "MRPL37, MRPS15, SDHB, UAP1, PRDX1, PRDX6, MARC2, B4GALT6, NDUFB3, TEK, TGFBI, COPS5, MTIF3, GTF3A, PLP1, F13A1, SERPINB6, NDUFA1, COX6B1, TNFRSF19, STEAP4, QRSL1, MPP1, COX7B, MRPS25, PDHA1, COQ3, DCLK1, TMTC1, ARNTL, LYVE1, TIMM17A, CTSL, TBX3, COQ10A, GLUL, MRPS9, CALCOCO2, PSMB7, PPIL1, RNF144B, MRPL15, WDR12, COX17, CILP, ENPEP, EGF, NDUFB10, OSR1, MEIS1, MRPS5, PTPRG, TBC1D7, FARS2, NDUFB9", "APIP, EI24, SPARCL1, RETREG1, ATP5MC3, ATP5PF, GTF3C6, VSIG4, ADK, UQCRB, MTERF3, PTDSS1, MALSU1, EDA, FCER1G, ATP5MC1, SNRNP25, PLPP3, CHCHD4, SUCLG1, PPM1L, PITX2, EGFLAM, FABP5, CTSB, COX6C, NDUFB6, SPTSSA, ATP5F1C, FUNDC2, AKTIP, MRPL58, ULK4, NT5DC2, FAM107A, NDUFS5, PPIC, UQCRFS1, HINT1, NFXL1, SPSB1, CHCHD1, STOX2, RSRC1, MSRA, ASB8, IDH2, KPNA2, UTP11, ZNF438, NDUFB1, UBE2F, NDUFA12", "NDUFA6, UBE2L3, TRIM69, PPP1CC, NDUFA4, APOD, CR1, RNF5, FASTKD5, EBF2, UQCRFS1P1, ATP5PO, MRPL33, TRMT9B, PSMB3

Organic substance metabolic process	180	0,0109	LAP3, AK2, NDUFAB1, HCCS, AASS, HSF2, LRRC7, MYOC, DAPK2, C6, GATB, FAR2, NDUFB4, ME1, MTHFD2, RRP15, PYGM, ADGRF5, TGFBR3, CAMK2A, DHRS9, SLC1A3, AACS, ME2, CD59, AKR1B1, MRPS33, NDUFB2, DESI1, FKBP3, PSMA3, PLTP, PSMD7, HMOX2, PDCD5, SSBP1, CHCHD3, PLGRKT, ACTA2, ENO3, MRPL27, ALDOC, NDUFC1, MRPL51, CHPT1, TPD52L1, MTRF1L, ACOT13, DUSP22, SLC27A6, PDE4D, RAD1, STC2, BCL6, CBLB, DNAJC27", "CACYBP, MRPL37, MRPS15, SDHB, UAP1, PRDX1, PRDX6, MARC2, B4GALT6, NDUFB3, TEK, TGFBI, COPS5, MTIF3, GTF3A, PTGFR, PLAU, PLP1, F13A1, SERPINB6, NDUFA1, MKKS, COX6B1, TNFRSF19, QRSL1, MPP1, COX7B, MRPS25, PDHA1, COQ3, DCLK1, TMTC1, ARNTL, LYVE1, TIMM17A, CTSL, TBX3, COQ10A, GLUL, MRPS9, PSMB7, PPIL1, RNF144B, MRPL15, WDR12, CILP, ENPEP, EGF, NDUFB10, PTRH2, OSR1, MEIS1, MRPS5, PTPRG, TBC1D7", "FARS2, NDUFB9, APIP, SPARCL1, ATP5MC3, ATP5PF, GTF3C6, VSIG4, ADK, UQCRB, MTERF3, PTDSS1, MALSU1, EDA, ADAMTS4, FCER1G, ATP5MC1, SNRNP25, PLPP3, CHCHD4, SUCLG1, PPM1L, PITX2, EGFLAM, FABP5, CTSB, COX6C, NDUFB6, SPTSSA, ATP5F1C, AKTIP, MRPL58, ULK4, FAM107A, NDUFS5, PPIC, UQCRFS1, HINT1, NFXL1, SPSB1, CHCHD1, STOX2, RSRC1, MSRA, ASB8, IDH2, KPNA2, UTP11, ZNF438, NDUFB1, UBE2F, NDUFA12, NDUFA6", "UBE2L3, TRIM69, PPP1CC, NDUFA4, APOD, CR1, RNF5, FASTKD5, EBF2, UQCRFS1P1, ATP5PO, MRPL33, TRMT9B, PSMB3

Primary metabolic process	170	0,0093	LAP3, AK2, NDUFAB1, HCCS, AASS, HSF2, LRRC7, MYOC, DAPK2, C6, GATB, FAR2, NDUFB4, ME1, RRP15, PYGM, ADGRF5, TGFBR3, CAMK2A, DHRS9, SLC1A3, AACS, ME2, CD59, AKR1B1, MRPS33, NDUFB2, DESI1, FKBP3, PSMA3, PLTP, PSMD7, PDCD5, SSBP1, CHCHD3, PLGRKT, ACTA2, ENO3, MRPL27, ALDOC, NDUFC1, MRPL51, CHPT1, TPD52L1, MTRF1L, ACOT13, DUSP22, SLC27A6, PDE4D, RAD1, STC2, BCL6, CBLB, DNAJC27, CACYBP, MRPL37", "MRPS15, SDHB, UAP1, PRDX1, PRDX6, B4GALT6, NDUFB3, TEK, TGFBI, COPS5, MTIF3, GTF3A, PLAU, PLP1, F13A1, SERPINB6, NDUFA1, COX6B1, TNFRSF19, QRSL1, MPP1, COX7B, MRPS25, PDHA1, COQ3, DCLK1, TMTC1, ARNTL, TIMM17A, CTSL, TBX3, GLUL, MRPS9, PSMB7, PPIL1, RNF144B, MRPL15, WDR12, ENPEP, EGF, NDUFB10, OSR1, MEIS1, MRPS5, PTPRG, TBC1D7, FARS2, NDUFB9, APIP, SPARCL1, ATP5MC3, ATP5PF, GTF3C6, VSIG4, ADK", "UQCRB, MTERF3, PTDSS1, MALSU1, EDA, ADAMTS4, ATP5MC1, SNRNP25, PLPP3, CHCHD4, SUCLG1, PPM1L, PITX2, EGFLAM, FABP5, CTSB, COX6C, NDUFB6, SPTSSA, ATP5F1C, AKTIP, MRPL58, ULK4, FAM107A, NDUFS5, PPIC, UQCRFS1, HINT1, NFXL1, SPSB1, CHCHD1, STOX2, RSRC1, MSRA, ASB8, IDH2, KPNA2, UTP11, ZNF438, NDUFB1, UBE2F, NDUFA12, NDUFA6, UBE2L3, TRIM69, PPP1CC, NDUFA4, APOD, CR1, RNF5, FASTKD5, EBF2, UQCRFS1P1", "ATP5PO, MRPL33, TRMT9B, PSMB3
Nitrogen compound metabolic process	165	0,0062	LAP3, AK2, NDUFAB1, HCCS, AASS, HSF2, LRRC7, MYOC, DAPK2, C6, GATB, FAR2, NDUFB4, ME1, MTHFD2, RRP15, TGFBR3, CAMK2A, SLC1A3, ME2, CD59, AKR1B1, MRPS33, NDUFB2, DESI1, FKBP3, PSMA3, PSMD7, HMOX2, PDCD5, SSBP1, CHCHD3, PLGRKT, ACTA2, ENO3, MRPL27, ALDOC, NDUFC1, MRPL51, CHPT1, TPD52L1, MTRF1L, ACOT13, DUSP22, PDE4D, RAD1, STC2, BCL6, CBLB, DNAJC27, CACYBP, MRPL37, MRPS15, UAP1, PRDX1, PRDX6", "MARC2, B4GALT6, NDUFB3, TEK, TGFBI, COPS5, MTIF3, GTF3A, PLAU, F13A1, SERPINB6, NDUFA1, COX6B1, TNFRSF19, QRSL1, MPP1, COX7B, MRPS25, PDHA1, DCLK1, TMTC1, ARNTL, LYVE1, TIMM17A, CTSL, TBX3, GLUL, MRPS9, PSMB7, PPIL1, RNF144B, MRPL15, WDR12, ENPEP, EGF, NDUFB10, OSR1, MEIS1, MRPS5, PTPRG, TBC1D7, FARS2, NDUFB9, APIP, SPARCL1, ATP5MC3, ATP5PF, GTF3C6, VSIG4, ADK, UQCRB, MTERF3, PTDSS1, MALSU1", "EDA, ADAMTS4, ATP5MC1, SNRNP25, PLPP3, CHCHD4, SUCLG1, PPM1L, PITX2, EGFLAM, FABP5, CTSB, COX6C, NDUFB6, SPTSSA, ATP5F1C, AKTIP, MRPL58, ULK4, FAM107A, NDUFS5, PPIC, UQCRFS1, HINT1, NFXL1, SPSB1, CHCHD1, STOX2, RSRC1, MSRA, ASB8, IDH2, KPNA2, UTP11, ZNF438, NDUFB1, UBE2F, NDUFA12, NDUFA6, UBE2L3, TRIM69, PPP1CC, NDUFA4, APOD, CR1, RNF5, FASTKD5, EBF2, UQCRFS1P1, ATP5PO, MRPL33, TRMT9B, PSMB3

Cellular component organization or biogenesis	121	0,0000	NDUFAB1, HCCS, SKAP2, AASS, LRRC7, MYOC, NDUFB4, ME1, TBC1D1, RRP15, TGFBR3, CAMK2A, PVR, TIMM21, SLC1A3, CADPS2, EPB41L3, CD59, MRPS33, NDUFB2, PLTP, CRISPLD2, PDCD5, SSBP1, CHCHD3, COA1, MRPL27, PMP22, NDUFC1, MRPL51, CHPT1, MTRF1L, ACOT13, DUSP22, RAD1, BCL6, MRPL37, MRPS15, NID1, B4GALT6, NDUFB3, KCNIP2, TEK, TGFBI, COPS5, GPSM2, MTIF3, GTF3A, PLP1, NDUFA1, MKKS, AIF1L, STEAP4, RTN4IP1", "MRPS25, RAP1GAP2, DCLK1, CHL1, TIMM17A, CTSL, GLUL, MRPS9, CALCOCO2, MRPL15, ARL3, WDR12, COX17, EGF, FBN2, NDUFB10, MRPS5, PTPRG, TBC1D7, SH3KBP1, NDUFB9, APIP, EI24, SPARCL1, RETREG1, ATP5MC3, ATP5PF, UQCRB, MTERF3, MALSU1, ADAMTS4, FCER1G, TOMM40L, ATP5MC1, CHCHD4, EGFLAM, NDUFB6, ATP5F1C, FUNDC2, GABRB3, AKTIP, MRPL58, ULK4, FAM107A, NDUFS5, STXBP6, REPS2, CHCHD1, LRRN3, ATP2A2, TUBB6, COX14", "UTP11, NDUFB1, NDUFA12, APOO, NDUFA6, UBE2L3, APOD, MFAP5, RNF5, VIT, ATP5PO, MRPL33, TIMM23
Cellular component organization	116	0,0001	NDUFAB1, HCCS, SKAP2, AASS, LRRC7, MYOC, NDUFB4, ME1, TBC1D1, TGFBR3, CAMK2A, PVR, TIMM21, SLC1A3, CADPS2, EPB41L3, CD59, MRPS33, NDUFB2, PLTP, CRISPLD2, PDCD5, SSBP1, CHCHD3, COA1, MRPL27, PMP22, NDUFC1, MRPL51, CHPT1, MTRF1L, ACOT13, DUSP22, RAD1, BCL6, MRPL37, MRPS15, NID1, B4GALT6, NDUFB3, KCNIP2, TEK, TGFBI, COPS5, GPSM2, MTIF3, PLP1, NDUFA1, MKKS, AIF1L, STEAP4, RTN4IP1, MRPS25, RAP1GAP2", "DCLK1, CHL1, TIMM17A, CTSL, GLUL, MRPS9, CALCOCO2, MRPL15, ARL3, COX17, EGF, FBN2, NDUFB10, MRPS5, PTPRG, TBC1D7, SH3KBP1, NDUFB9, APIP, EI24, SPARCL1, RETREG1, ATP5MC3, ATP5PF, UQCRB, MTERF3, ADAMTS4, FCER1G, TOMM40L, ATP5MC1, CHCHD4, EGFLAM, NDUFB6, ATP5F1C, FUNDC2, GABRB3, AKTIP, MRPL58, ULK4, FAM107A, NDUFS5, STXBP6, REPS2, CHCHD1, LRRN3, ATP2A2, TUBB6, COX14, NDUFB1, NDUFA12, APOO, NDUFA6", "UBE2L3, APOD, MFAP5, RNF5, VIT, ATP5PO, MRPL33, TIMM23

Cellular protein metabolic process	86	0,0186	NDUFAB1, HCCS, LRRC7, MYOC, DAPK2, C6, GATB, TGFBR3, CAMK2A, CD59, MRPS33, FKBP3, PSMA3, PSMD7, PDCD5, PLGRKT, MRPL27, MRPL51, TPD52L1, MTRF1L, DUSP22, PDE4D, STC2, BCL6, CBLB, DNAJC27, MRPL37, MRPS15, PRDX1, B4GALT6, TEK, TGFBI, COPS5, MTIF3, F13A1, SERPINB6, TNFRSF19, QRSL1, MRPS25, DCLK1, TMTC1, ARNTL, TIMM17A, CTSL, GLUL, MRPS9, PSMB7, PPIL1, RNF144B, MRPL15, EGF, MRPS5, PTPRG, TBC1D7", "FARS2, APIP, SPARCL1, VSIG4, MTERF3, MALSU1, PLPP3, CHCHD4, PPM1L, EGFLAM, CTSB, AKTIP, MRPL58, ULK4, FAM107A, PPIC, SPSB1, CHCHD1, RSRC1, MSRA, ASB8, UBE2F, UBE2L3, TRIM69, PPP1CC, CR1, RNF5, FASTKD5, MRPL33, PSMB3
Transport	82	0,0437	LRRC7, TBC1D1, GNB5, CAMK2A, TIMM21, SLC1A3, KCNN2, AACS, CADPS2, CD59, SIGLEC1, CD209, PSMA3, PLTP, PSMD7, CRISPLD2, HMOX2, NCALD, SLC39A14, PDCD5, ALDOC, SLC27A6, PDE4D, STC2, CBLB, DNAJC27, PRDX6, KCNIP2, COPS5, PLAU, F13A1, SERPINB6, MKKS, COX6B1, STEAP4, MTX2, COX7B, DCLK1, ARNTL, TIMM17A, TBX3, GLUL, PSMB7, ARL3, COX17, EGF, OSR1, SLC25A26, SH3KBP1, ATP5MC3, ATP5PF, SNX22, FCER1G, TOMM40L", "ATP5MC1, CHCHD4, FABP5, CTSB, COX6C, ATP5F1C, GABRB3, AKTIP, LRP1B, STXBP6, SCN9A, ATP2A2, RSRC1, CD163, SLC36A4, IDH2, KPNA2, APOO, UBE2L3, PPP1CC, NDUFA4, APOD, CR1, RNF5, ATP5PO, MRC1, TIMM23, PSMB3
Phosphorus metabolic process	76	0,0000	AK2, NDUFAB1, AASS, LRRC7, MYOC, DAPK2, FAR2, NDUFB4, ME1, ADGRF5, TGFBR3, CAMK2A, ME2, NDUFB2, PSMA3, PSMD7, ENO3, ALDOC, NDUFC1, CHPT1, HDDC2, TPD52L1, ACOT13, DUSP22, PDE4D, CBLB, DNAJC27, UAP1, PRDX1, PRDX6, NDUFB3, TEK, COPS5, NDUFA1, COX6B1, TNFRSF19, MPP1, COX7B, PDHA1, DCLK1, PSMB7, CILP, EGF, NDUFB10, PTPRG, NDUFB9, APIP, ATP5MC3, ATP5PF, ADK, UQCRB, PTDSS1, ATP5MC1, PLPP3, SUCLG1", "PPM1L, FABP5, COX6C, NDUFB6, ATP5F1C, AKTIP, ULK4, NT5DC2, NDUFS5, UQCRFS1, HINT1, RSRC1, IDH2, NDUFB1, NDUFA12, NDUFA6, PPP1CC, NDUFA4, FASTKD5, UQCRFS1P1, ATP5PO, PSMB3

Small molecule metabolic process	69	0,0000	AK2, NDUFAB1, AASS, GATB, FAR2, NDUFB4, ME1, MTHFD2, DHRS9, SLC1A3, AACS, ME2, AKR1B1, NDUFB2, PSMA3, PLTP, PSMD7, ENO3, ALDOC, NDUFC1, ACOT13, SLC27A6, PDE4D, SDHB, UAP1, MARC2, B4GALT6, NDUFB3, PLP1, NDUFA1, COX6B1, QRSL1, MPP1, COX7B, PDHA1, COQ3, LYVE1, COQ10A, GLUL, PSMB7, EGF, NDUFB10, FARS2, NDUFB9, APIP, ATP5MC3, ATP5PF, ADK, UQCRB, ATP5MC1, SUCLG1, EGFLAM, FABP5, COX6C, NDUFB6", "ATP5F1C, NDUFS5, UQCRFS1, HINT1, MSRA, IDH2, NDUFB1, NDUFA12, NDUFA6, NDUFA4, APOD, UQCRFS1P1, ATP5PO, PSMB3
Cellular component biogenesis	57	0,0288	NDUFAB1, HCCS, SKAP2, AASS, MYOC, NDUFB4, ME1, RRP15, TGFBR3, TIMM21, CADPS2, EPB41L3, CD59, NDUFB2, COA1, PMP22, NDUFC1, ACOT13, DUSP22, NDUFB3, TEK, COPS5, GPSM2, GTF3A, NDUFA1, MKKS, AIF1L, STEAP4, GLUL, MRPS9, ARL3, WDR12, COX17, EGF, NDUFB10, TBC1D7, NDUFB9, APIP, UQCRB, MTERF3, MALSU1, FCER1G, NDUFB6, GABRB3, FAM107A, NDUFS5, STXBP6, REPS2, LRRN3, COX14, UTP11, NDUFB1, NDUFA12, NDUFA6", "APOD, RNF5
Phosphorylation	56	0,0000	AK2, NDUFAB1, LRRC7, MYOC, DAPK2, NDUFB4, TGFBR3, CAMK2A, NDUFB2, PSMA3, PSMD7, ENO3, ALDOC, NDUFC1, TPD52L1, DUSP22, PDE4D, CBLB, DNAJC27, PRDX1, NDUFB3, TEK, COPS5, NDUFA1, COX6B1, TNFRSF19, COX7B, DCLK1, PSMB7, EGF, NDUFB10, NDUFB9, APIP, ATP5MC3, ATP5PF, ADK, UQCRB, ATP5MC1, PLPP3, SUCLG1, PPM1L, COX6C, NDUFB6, ATP5F1C, AKTIP, ULK4, NDUFS5, UQCRFS1, RSRC1, NDUFB1, NDUFA12, NDUFA6, NDUFA4", "FASTKD5, UQCRFS1P1, ATP5PO, PSMB3

Protein-containing complex subunit organization	53	0,0000	NDUFAB1, HCCS, SKAP2, AASS, NDUFB4, ME1, TGFBR3, TIMM21, CADPS2, CD59, MRPS33, NDUFB2, PLTP, COA1, MRPL27, NDUFC1, MRPL51, MTRF1L, ACOT13, MRPL37, MRPS15, NDUFB3, TEK, MTIF3, NDUFA1, MKKS, STEAP4, MRPS25, GLUL, MRPS9, CALCOCO2, MRPL15, COX17, EGF, NDUFB10, MRPS5, NDUFB9, APIP, UQCRB, MTERF3, FCER1G, NDUFB6, MRPL58, FAM107A, NDUFS5, STXBP6, REPS2, CHCHD1, COX14, NDUFB1, NDUFA12, NDUFA6, MRPL33")
Oxidation-reduction process	50	0,0000	NDUFAB1, HCCS, AASS, FAR2, NDUFB4, ME1, MTHFD2, PYGM, ADGRF5, DHRS9, ME2, AKR1B1, NDUFB2, HSDL1, HMOX2, ENO3, ALDOC, NDUFC1, SDHB, PRDX1, PRDX6, MARC2, NDUFB3, NDUFA1, COX6B1, STEAP4, RTN4IP1, COX7B, PDHA1, COQ10A, NDUFB10, GFOD1, NDUFB9, UQCRB, CHCHD4, SUCLG1, COX6C, NDUFB6, NDUFS5, UQCRFS1, MSRA, IDH2, NDUFB1, NDUFA12, NDUFA6, PPP1CC, NDUFA4, APOD, FASTKD5, UQCRFS1P1, TRMT9B
Generation of precursor metabolites and energy	41	0,0000	NDUFAB1, NDUFB4, ME1, PYGM, ADGRF5, AACS, ME2, AKR1B1, NDUFB2, ENO3, ALDOC, NDUFC1, SDHB, NDUFB3, NDUFA1, COX6B1, STEAP4, COX7B, PDHA1, COQ10A, COX17, NDUFB10, NDUFB9, ATP5MC3, ATP5PF, UQCRB, ATP5MC1, SUCLG1, COX6C, NDUFB6, ATP5F1C, NDUFS5, UQCRFS1, IDH2, NDUFB1, NDUFA12, NDUFA6, PPP1CC, NDUFA4, FASTKD5, UQCRFS1P1, ATP5PO
Nucleoside phosphate metabolic process	39	0,0000	AK2, NDUFAB1, AASS, FAR2, NDUFB4, ME1, ME2, NDUFB2, ENO3, ALDOC, NDUFC1, ACOT13, PDE4D, NDUFB3, NDUFA1, COX6B1, MPP1, COX7B, PDHA1, NDUFB10, NDUFB9, ATP5MC3, ATP5PF, ADK, UQCRB, ATP5MC1, SUCLG1, COX6C, NDUFB6, ATP5F1C, NDUFS5, UQCRFS1, HINT1, IDH2, NDUFB1, NDUFA12, NDUFA6, NDUFA4, UQCRFS1P1, ATP5PO
Nucleotide metabolic process	39	0,0000	AK2, NDUFAB1, AASS, FAR2, NDUFB4, ME1, ME2, NDUFB2, ENO3, ALDOC, NDUFC1, ACOT13, PDE4D, NDUFB3, NDUFA1, COX6B1, MPP1, COX7B, PDHA1, NDUFB10, NDUFB9, ATP5MC3, ATP5PF, ADK, UQCRB, ATP5MC1, SUCLG1, COX6C, NDUFB6, ATP5F1C, NDUFS5, UQCRFS1, HINT1, IDH2, NDUFB1, NDUFA12, NDUFA6, NDUFA4, UQCRFS1P1, ATP5PO
Purine nucleotide metabolic process	36	0,0000	AK2, NDUFAB1, AASS, FAR2, NDUFB4, NDUFB2, ENO3, ALDOC, NDUFC1, ACOT13, PDE4D, NDUFB3, NDUFA1, COX6B1, MPP1, COX7B, PDHA1, NDUFB10, NDUFB9, ATP5MC3, ATP5PF, ADK, UQCRB, ATP5MC1, SUCLG1, COX6C, NDUFB6, ATP5F1C, NDUFS5, UQCRFS1, HINT1, NDUFB1, NDUFA12, NDUFA6, NDUFA4, UQCRFS1P1, ATP5PO
Transmembrane transport	35	0,0025	LRRC7, GNB5, TIMM21, SLC1A3, KCNN2, PSMA3, PSMD7, SLC39A14, PDE4D, KCNIP2, COX6B1, STEAP4, COX7B, TIMM17A, PSMB7, COX17, OSR1, SLC25A26, ATP5MC3, ATP5PF, TOMM40L, ATP5MC1, CHCHD4, FABP5, COX6C, ATP5F1C, GABRB3, SCN9A, ATP2A2, SLC36A4, NDUFA4, RNF5, ATP5PO, TIMM23, PSMB3

Mitochondrion organization	34	0,0000	NDUFAB1, NDUFB4, CAMK2A, TIMM21, NDUFB2, PDCD5, SSBP1, CHCHD3, COA1, NDUFC1, NDUFB3, NDUFA1, TIMM17A, COX17, NDUFB10, NDUFB9, ATP5MC3, ATP5PF, UQCRB, TOMM40L, ATP5MC1, CHCHD4, NDUFB6, ATP5F1C, FUNDC2, NDUFS5, COX14, NDUFB1, NDUFA12, APOO, NDUFA6, UBE2L3, ATP5PO, TIMM23
Organic acid metabolic process	33	0,0001	NDUFAB1, AASS, GATB, ME1, MTHFD2, DHRS9, SLC1A3, AACS, ME2, PSMA3, PSMD7, ENO3, ALDOC, SLC27A6, SDHB, MARC2, B4GALT6, PLP1, QRSL1, PDHA1, LYVE1, GLUL, PSMB7, EGF, FARS2, APIP, SUCLG1, EGFLAM, FABP5, MSRA, IDH2, PSMB3
Ion transport	31	0,0450	LRRC7, GNB5, CAMK2A, SLC1A3, KCNN2, PLTP, SLC39A14, SLC27A6, PDE4D, STC2, KCNIP2, COX6B1, STEAP4, COX7B, COX17, EGF, OSR1, SLC25A26, ATP5MC3, ATP5PF, FCER1G, TOMM40L, ATP5MC1, COX6C, ATP5F1C, GABRB3, SCN9A, ATP2A2, SLC36A4, NDUFA4, ATP5PO
Energy derivation by oxidation of organic compounds	29	0,0000	NDUFAB1, NDUFB4, PYGM, ADGRF5, ME2, NDUFB2, NDUFC1, SDHB, NDUFB3, NDUFA1, COX6B1, COX7B, PDHA1, COQ10A, NDUFB10, NDUFB9, UQCRB, SUCLG1, COX6C, NDUFB6, NDUFS5, UQCRFS1, IDH2, NDUFB1, NDUFA12, NDUFA6, PPP1CC, NDUFA4, FASTKD5, UQCRFS1P1
ATP metabolic process	27	0,0000	AK2, NDUFAB1, NDUFB4, NDUFB2, ENO3, ALDOC, NDUFC1, NDUFB3, NDUFA1, COX6B1, COX7B, NDUFB10, NDUFB9, ATP5MC3, ATP5PF, UQCRB, ATP5MC1, COX6C, NDUFB6, ATP5F1C, NDUFS5, UQCRFS1, NDUFB1, NDUFA12, NDUFA6, NDUFA4, UQCRFS1P1, ATP5PO
Cation transport	26	0,0067	LRRC7, GNB5, CAMK2A, SLC1A3, KCNN2, SLC39A14, PDE4D, STC2, KCNIP2, COX6B1, STEAP4, COX7B, COX17, EGF, OSR1, ATP5MC3, ATP5PF, FCER1G, ATP5MC1, COX6C, ATP5F1C, SCN9A, ATP2A2, SLC36A4, NDUFA4, ATP5PO
Cellular protein-containing complex assembly	26	0,0079	NDUFAB1, HCCS, NDUFB4, TGFBR3, TIMM21, CADPS2, NDUFB2, COA1, NDUFC1, NDUFB3, NDUFA1, MKKS, COX17, EGF, NDUFB10, NDUFB9, UQCRB, MTERF3, NDUFB6, FAM107A, NDUFS5, STXBP6, COX14, NDUFB1, NDUFA12, NDUFA6
Cellular respiration	26	0,0000	NDUFAB1, NDUFB4, ME2, NDUFB2, NDUFC1, SDHB, NDUFB3, NDUFA1, COX6B1, COX7B, PDHA1, COQ10A, NDUFB10, NDUFB9, UQCRB, SUCLG1, COX6C, NDUFB6, NDUFS5, UQCRFS1, IDH2, NDUFB1, NDUFA12, NDUFA6, NDUFA4, FASTKD5, UQCRFS1P1
Oxidative phosphorylation	24	0,0000	NDUFAB1, NDUFB4, NDUFB2, NDUFC1, NDUFB3, NDUFA1, COX6B1, COX7B, NDUFB10, NDUFB9, ATP5MC3, ATP5PF, UQCRB, ATP5MC1, COX6C, NDUFB6, ATP5F1C, NDUFS5, UQCRFS1, NDUFB1, NDUFA12, NDUFA6, NDUFA4, UQCRFS1P1, ATP5PO

Electron transport chain	24	0,0000	NDUFAB1, NDUFB4, ME1, ME2, AKR1B1, NDUFB2, NDUFC1, SDHB, NDUFB3, NDUFA1, COX6B1, STEAP4, COX7B, NDUFB10, NDUFB9, UQCRB, COX6C, NDUFB6, NDUFS5, UQCRFS1, NDUFB1, NDUFA12, NDUFA6, NDUFA4, UQCRFS1P1
Ion transmembrane transport	23	0,0446	LRRC7, GNB5, SLC1A3, KCNN2, SLC39A14, PDE4D, KCNIP2, COX6B1, STEAP4, COX7B, COX17, OSR1, ATP5MC3, ATP5PF, ATP5MC1, COX6C, ATP5F1C, GABRB3, SCN9A, ATP2A2, SLC36A4, NDUFA4, ATP5PO
Peptide metabolic process	22	0,0043	GATB, MRPS33, MRPL27, MRPL51, MTRF1L, MRPL37, MRPS15, COPS5, MTIF3, QRSL1, MRPS25, MRPS9, MRPL15, ENPEP, MRPS5, FARS2, MTERF3, MALSU1, MRPL58, CHCHD1, MRPL33
Translation	21	0,0006	GATB, MRPS33, MRPL27, MRPL51, MTRF1L, MRPL37, MRPS15, COPS5, MTIF3, QRSL1, MRPS25, MRPS9, MRPL15, MRPS5, FARS2, MTERF3, MALSU1, MRPL58, CHCHD1, MRPL33
Exocytosis	21	0,0097	LRRC7, CAMK2A, CADPS2, CD59, PSMD7, CRISPLD2, HMOX2, ALDOC, PRDX6, COPS5, PLAU, F13A1, SERPINB6, PSMB7, EGF, FCER1G, FABP5, CTSB, STXBP6, ATP2A2, CR1
Peptide biosynthetic process	21	0,0010	GATB, MRPS33, MRPL27, MRPL51, MTRF1L, MRPL37, MRPS15, COPS5, MTIF3, QRSL1, MRPS25, MRPS9, MRPL15, MRPS5, FARS2, MTERF3, MALSU1, MRPL58, CHCHD1, MRPL33
Membrane organization	21	0,0086	LRRC7, MYOC, TBC1D1, CAMK2A, EPB41L3, CD59, PDCD5, CHCHD3, KCNIP2, EGF, SH3KBP1, ATP5MC3, ATP5PF, FCER1G, ATP5MC1, ATP5F1C, STXBP6, REPS2, ATP2A2, APOO, ATP5PO
Leukocyte mediated immunity	19	0,0254	LRRC7, C6, PVR, CD59, PSMD7, CRISPLD2, HMOX2, ALDOC, DUSP22, BCL6, PRDX1, PRDX6, PLAU, SERPINB6, PSMB7, FCER1G, FABP5, CTSB, CR1
ATP synthesis coupled electron transport	19	0,0000	NDUFAB1, NDUFB4, NDUFB2, NDUFC1, NDUFB3, NDUFA1, COX6B1, COX7B, NDUFB10, NDUFB9, UQCRB, COX6C, NDUFB6, NDUFS5, UQCRFS1, NDUFB1, NDUFA12, NDUFA6, NDUFA4, UQCRFS1P1
Mitochondrial translation	18	0,0000	GATB, MRPS33, MRPL27, MRPL51, MTRF1L, MRPL37, MRPS15, MTIF3, QRSL1, MRPS25, MRPS9, MRPL15, MRPS5, MTERF3, MALSU1, MRPL58, CHCHD1, MRPL33
Mitochondrial respiratory chain complex assembly	18	0,0000	NDUFAB1, NDUFB4, TIMM21, NDUFB2, COA1, NDUFC1, NDUFB3, NDUFA1, COX17, NDUFB10, NDUFB9, UQCRB, NDUFB6, NDUFS5, COX14, NDUFB1, NDUFA12, NDUFA6

NADH dehydrogenase complex assembly	15	0,0000	NDUFAB1, NDUFB4, TIMM21, NDUFB2, COA1, NDUFC1, NDUFB3, NDUFA1, NDUFB10, NDUFB9, NDUFB6, NDUFS5, NDUFB1, NDUFA12, NDUFA6
Neutrophil activation involved in immune response	14	0,0061	LRRC7, CD59, PSMD7, CRISPLD2, HMOX2, ALDOC, PRDX6, PLAU, SERPINB6, PSMB7, FCER1G, FABP5, CTSB, CR1
Myeloid leukocyte mediated immunity	14	0,0163	LRRC7, CD59, PSMD7, CRISPLD2, HMOX2, ALDOC, PRDX6, PLAU, SERPINB6, PSMB7, FCER1G, FABP5, CTSB, CR1
Neutrophil mediated immunity	14	0,0073	LRRC7, CD59, PSMD7, CRISPLD2, HMOX2, ALDOC, PRDX6, PLAU, SERPINB6, PSMB7, FCER1G, FABP5, CTSB, CR1
Coenzyme metabolic process	14	0,0011	AASS, FAR2, ME1, MTHFD2, ME2, ENO3, ALDOC, ACOT13, PDHA1, COQ3, COQ10A, APIP, SUCLG1, IDH2
Mitochondrial transport	14	0,0000	CAMK2A, TIMM21, PDCD5, MTX2, TIMM17A, ATP5MC3, ATP5PF, TOMM40L, ATP5MC1, CHCHD4, ATP5F1C, UBE2L3, ATP5PO, TIMM23
Granulocyte activation	14	0,0080	LRRC7, CD59, PSMD7, CRISPLD2, HMOX2, ALDOC, PRDX6, PLAU, SERPINB6, PSMB7, FCER1G, FABP5, CTSB, CR1
Neutrophil activation	14	0,0072	LRRC7, CD59, PSMD7, CRISPLD2, HMOX2, ALDOC, PRDX6, PLAU, SERPINB6, PSMB7, FCER1G, FABP5, CTSB, CR1
Cell-substrate adhesion	13	0,0010	MYOC, SIGLEC1, DUSP22, BCL6, NID1, TEK, PLAU, LYVE1, EDA, EGFLAM, FAM107A, APOD, VIT
Skeletal system development	12	0,0457	MYOC, PRDX1, TEK, TGFBI, MKKS, TBX3, FBN2, OSR1, MEIS1, ADAMTS4, PITX2, VIT
Response to oxidative stress	11	0,0401	NDUFB4, HMOX2, STC2, PRDX1, PRDX6, ARNTL, ATP2A2, MSRA, NDUFA12, NDUFA6, APOD
Cell-matrix adhesion	11	0,0002	MYOC, SIGLEC1, DUSP22, BCL6, NID1, TEK, PLAU, LYVE1, EDA, FAM107A, APOD
Protein import	10	0,0004	TIMM21, PDCD5, CBLB, ARNTL, TIMM17A, TOMM40L, CHCHD4, KPNA2, APOD, TIMM23

Mitochondrial transmembrane transport	10	0,0000	TIMM21, TIMM17A, ATP5MC3, ATP5PF, TOMM40L, ATP5MC1, CHCHD4, ATP5F1C, ATP5PO, TIMM23
Protein polyubiquitination	9	0,0237	PSMA3, PSMD7, PSMB7, RNF144B, SPSB1, UBE2L3, TRIM69, RNF5, PSMB3
Innate immune response-activating signal transduction	8	0,0470	CD209, PSMA3, PSMD7, CTSL, PSMB7, FCER1G, CTSB, PSMB3
Protein folding	8	0,0114	GNB5, PDCD5, EMC3, MKKS, PPIL1, CHCHD4, DNAJC21, PPIC
Oxidoreduction coenzyme metabolic process	8	0,0067	ME1, ME2, ENO3, ALDOC, PDHA1, COQ3, COQ10A, IDH2
Regulation of cell-matrix adhesion	7	0,0011	MYOC, DUSP22, BCL6, TEK, PLAU, FAM107A, APOD
Protein targeting to mitochondrion	7	0,0003	TIMM21, PDCD5, TIMM17A, TOMM40L, CHCHD4, UBE2L3, TIMM23
Innate immune response activating cell surface receptor signaling pathway	6	0,0046	CD209, PSMA3, PSMD7, PSMB7, FCER1G, PSMB3
Stimulatory C-type lectin receptor signaling pathway	6	0,0041	CD209, PSMA3, PSMD7, PSMB7, FCER1G, PSMB3

Antigen processing and presentation of exogenous peptide antigen	6	0,0301	PSMA3, PSMD7, CTSL, PSMB7, FCER1G, PSMB3
tRNA metabolic process	6	0,0450	GATB, QRSL1, FARS2, GTF3C6, TRMT9B
Regulation of blood pressure	6	0,0354	NCALD, ACTA2, PDE4D, MKKS, ENPEP, ATP5PF
Regulation of protein maturation	6	0,0354	C6, CD59, PLGRKT, TIMM17A, VSIG4, CR1
Antigen processing and presentation of peptide antigen via MHC class I	5	0,0093	PSMA3, PSMD7, PSMB7, FCER1G, PSMB3
Regulation of lymphocyte mediated immunity	5	0,0495	PVR, DUSP22, BCL6, FCER1G, CR1
Protein transmembrane transport	5	0,0017	TIMM21, TIMM17A, TOMM40L, CHCHD4, TIMM23
Antigen processing and presentation of exogenous peptide antigen via MHC class I, TAP-dependent	4	0,0179	PSMA3, PSMD7, PSMB7, PSMB3

Positive regulation of heart rate	3	0,0048	PDE4D, ATP5PF, ATP2A2
Regulation of the force of heart contraction	2	0,0499	ATP2A2, MYL4
Vascular smooth muscle contraction	2	0,0434	ACTA2, MKKS
Relaxation of cardiac muscle	2	0,0188	PDE4D, ATP2A2
Sinoatrial node development	1	0,0394	TBX3
Atrioventricular bundle cell differentiation	1	0,0133	TBX3

Table S6. Gene Ontology Molecular Function for under-exp

Molecular Function	DEG number	P DE
Molecular function	256	0,0001

Binding	216	0,0436
Catalytic activity	119	0,0000
Oxidoreductase activity	39	0,0000
Transporter activity	24	0,0183
Transmembrane transporter activity	21	0,0404

Structural molecule activity	18	0,0157
NADH dehydrogenase (quinone) activity	14	0,0000
Cofactor binding	13	0,0187
Electron transfer activity	12	0,0000
Structural constituent of ribosome	10	0,0004
Proton transmembrane transporter activity	10	0,0000

ɔressed genes

Gene name
LAP3,AK2,NDUFAB1,HCCS,SKAP2,AASS,HSF2,LRRC7,MYOC,DAPK2,C6,GATB,FAR2,NDUFB4,ME1,TBC1D1,MTHFD2,ISOC1,PYGM,ADGRF5,TGFBR3,GNB5,CAMK2A,PVR,DHRS9,TIMM21,SLC1A3,KCNN2,AACS,CADPS2,ME2,EPB41L3,CD59,AKR1B1,SIGLEC1,MRPS33,NDUFB2,CD209,DESI1,FKBP3,PSMA3,PLTP,PSMD7,HSDL1,CRISPLD2,HMOX2,RASL12,PDGFRL,NCALD,SLC39A14,PDCD5,LILRB5,SSBP1,CHCHD3,PLGRKT","ACTA2,ENO3,MRPL27,PMP22,ALDOC,NDUFC1,MS4A4A,MRPL51,CHPT1,HDDC2,TPD52L1,MTRF1L,ACOT13,DUSP22,SLC27A6,PDE4D,RAD1,STC2,BCL6,CBLB,DNAJC27,CACYBP,RALGPS2,MRPL37,MRPS15,NID1,SDHB,UAP1,PRDX1,PRDX6,MARC2,B4GALT6,NDUFB3,KCNIP2,TEK,TGFBI,COPS5,GPSM2,MTIF3,GTF3A,PTGFR,PLAU,PLP1,F13A1,SERPINB6,EMC3,NDUFA1,MKKS,COX6B1,AIF1L,TNFRSF19,STEAP4,MTX2,MYO5C","RTN4IP1,QRSL1,MPP1,COX7B,MRPS25,PDHA1,RAP1GAP2,COQ3,DCLK1,STARD13,TMTC1,ARNTL,LYVE1,CHL1,EMC7,TIMM17A,CTSL,TBX3,COQ10A,GLUL,MRPS9,CALCOCO2,PSMB7,PPIL1,RNF144B,MRPL15,ARL3,WDR12,COX17,CILP,ENPEP,EGF,FBN2,NDUFB10,PTRH2,OSR1,MEIS1,MRPS5,PTPRG,SLC25A26,TBC1D7,FARS2,GFOD1,SH3KBP1,ITGB1BP2,NDUFB9,APIP,SPARCL1,RETREG1,ATP5MC3,ATP5PF,GTF3C6,VSIG4,ADK","ATP5MPL,UQCRB,MTERF3,PTDSS1,MALSU1,SNX22,EDA,ADAMTS4,FCER1G,TOMM40L,ATP5MC1,SNRNP25,PLPP3,CHCHD4,SUCLG1,PPM1L,PITX2,HTR4,EGFLAM,FABP5,CTSB,COX6C,NDUFB6,SPTSSA,ATP5F1C,CMTM5,GABRB3,AKTIP,MRPL58,ULK4,NT5DC2,FAM107A,NDUFS5,LRP1B,DNAJC21,PPIC,STXBP6,UQCRFS1,SCN9A,HINT1,REPS2,METTL7B,NFXL1,SPSB1,CHCHD1,STOX2,MICOS10,ATP2A2,RSRC1,MSRA,TUBB6,CD163,SELENOW","SLC36A4,IDH2,KPNA2,RGS6,UTP11,ZNF438,NDUFB1,UBE2F,NDUFA12,APOO,NDUFA6,UBE2L3,TRIM69,PPP1CC,C2ORF88,NDUFA4,APOD,CBWD3,MFAP5,MYL4,LRRC10,CR1,RNF5,VIT,KLHL23,TMX2,S1PR3,FASTKD5,EBF2,MYBPHL,UQCRFS1P1,ATP5PO,MRPL33,TRMT9B,TRNP1,MRC1,TIMM23,PSMB3

LAP3,AK2,NDUFAB1,HCCS,SKAP2,HSF2,LRRC7,MYOC,DAPK2,C6,GATB,ME1,TBC1D1,MTHFD2,ISOC1,PYGM,TGFBR3,GNB5,CAMK2A,PVR,TIMM21,SLC1A3,KCNN2,AACS,CADPS2,ME2,EPB41L3,CD59,SIGLEC1,CD209,DESI1,FKBP3,PSMA3,PLTP,PSMD7,HSDL1,CRISPLD2,HMOX2,RASL12,NCALD,PDCD5,SSBP1,CHCHD3,PLGRKT,ACTA2,ENO3,MRPL27,PMP22,ALDOC,MS4A4A,MRPL51,CHPT1,HDDC2,TPD52L1,MTRF1L,DUSP22","SLC27A6,PDE4D,RAD1,STC2,BCL6,CBLB,DNAJC27,CACYBP,RALGPS2,MRPL37,MRPS15,NID1,SDHB,UAP1,PRDX1,PRDX6,MARC2,B4GALT6,KCNIP2,TEK,TGFBI,COPS5,GPSM2,MTIF3,GTF3A,PLAU,PLP1,F13A1,SERPINB6,MKKS,AIF1L,TNFRSF19,STEAP4,MTX2,MYO5C,RTN4IP1,QRSL1,MPP1,PDHA1,RAP1GAP2,COQ3,DCLK1,STARD13,ARNTL,LYVE1,CHL1,EMC7,CTSL,TBX3,COQ10A,GLUL,MRPS9,CALCOCO2,PSMB7,PPIL1","RNF144B,MRPL15,ARL3,WDR12,COX17,ENPEP,EGF,FBN2,NDUFB10,PTRH2,OSR1,MEIS1,MRPS5,PTPRG,TBC1D7,FARS2,GFOD1,SH3KBP1,ITGB1BP2,NDUFB9,APIP,SPARCL1,RETREG1,ATP5MC3,ATP5PF,GTF3C6,VSIG4,ADK,UQCRB,MTERF3,MALSU1,SNX22,EDA,ADAMTS4,FCER1G,TOMM40L,ATP5MC1,SNRNP25,PLPP3,CHCHD4,SUCLG1,PPM1L,PITX2,HTR4,EGFLAM,FABP5,CTSB,SPTSSA,ATP5F1C,CMTM5,GABRB3,AKTIP,MRPL58","ULK4,NT5DC2,FAM107A,LRP1B,DNAJC21,PPIC,STXBP6,UQCRFS1,SCN9A,HINT1,REPS2,NFXL1,SPSB1,CHCHD1,MICOS10,ATP2A2,RSRC1,TUBB6,CD163,IDH2,KPNA2,RGS6,UTP11,ZNF438,UBE2F,APOO,UBE2L3,TRIM69,PPP1CC,C2ORF88,NDUFA4,APOD,CBWD3,MYL4,LRRC10,CR1,RNF5,VIT,KLHL23,S1PR3,FASTKD5,EBF2,MYBPHL,UQCRFS1P1,ATP5PO,TRMT9B,TRNP1,MRC1,TIMM23,PSMB3
LAP3,AK2,NDUFAB1,HCCS,AASS,DAPK2,GATB,FAR2,NDUFB4,ME1,MTHFD2,ISOC1,PYGM,TGFBR3,GNB5,CAMK2A,DHRS9,AACS,ME2,AKR1B1,NDUFB2,DESI1,FKBP3,PSMA3,HMOX2,RASL12,PDGFRL,ENO3,ALDOC,NDUFC1,CHPT1,HDDC2,ACOT13,DUSP22,SLC27A6,PDE4D,RAD1,CBLB,DNAJC27,SDHB,UAP1,PRDX1,PRDX6,MARC2,B4GALT6,NDUFB3,TEK,COPS5,PLAU,F13A1,NDUFA1,COX6B1,STEAP4,MYO5C,RTN4IP1,QRSL1","MPP1,COX7B,PDHA1,COQ3,DCLK1,TMTC1,CTSL,GLUL,PSMB7,PPIL1,RNF144B,ARL3,ENPEP,NDUFB10,PTRH2,PTPRG,FARS2,GFOD1,NDUFB9,APIP,ATP5MC3,ATP5PF,ADK,UQCRB,PTDSS1,ADAMTS4,ATP5MC1,PLPP3,CHCHD4,SUCLG1,PPM1L,CTSB,COX6C,NDUFB6,SPTSSA,ATP5F1C,MRPL58,ULK4,NT5DC2,NDUFS5,PPIC,UQCRFS1,HINT1,METTL7B,ATP2A2,MSRA,TUBB6,IDH2,RGS6,NDUFB1,UBE2F,NDUFA12,NDUFA6,UBE2L3","TRIM69,PPP1CC,NDUFA4,RNF5,FASTKD5,UQCRFS1P1,ATP5PO,TRMT9B,PSMB3
NDUFAB1,AASS,FAR2,NDUFB4,ME1,MTHFD2,DHRS9,ME2,AKR1B1,NDUFB2,HMOX2,NDUFC1,SDHB,PRDX1,PRDX6,MARC2,NDUFB3,NDUFA1,COX6B1,STEAP4,RTN4IP1,COX7B,PDHA1,NDUFB10,GFOD1,NDUFB9,UQCRB,CHCHD4,COX6C,NDUFB6,NDUFS5,UQCRFS1,MSRA,IDH2,NDUFB1,NDUFA12,NDUFA6,NDUFA4,UQCRFS1P1,TRMT9B
SLC1A3,KCNN2,PLTP,SLC39A14,SLC27A6,KCNIP2,COX6B1,COX7B,TIMM17A,SLC25A26,ATP5MC3,ATP5PF,TOMM40L,ATP5MC1,COX6C,ATP5F1C,GABRB3,SCN9A,ATP2A2,SLC36A4,NDUFA4,APOD,ATP5PO,TIMM23
SLC1A3,KCNN2,SLC39A14,KCNIP2,COX6B1,COX7B,TIMM17A,SLC25A26,ATP5MC3,ATP5PF,TOMM40L,ATP5MC1,COX6C,ATP5F1C,GABRB3,SCN9A,ATP2A2,SLC36A4,NDUFA4,ATP5PO,TIMM23

EPB41L3,MRPS33,MRPL27,MRPL51,MRPL37,MRPS15,NID1,TGFBI,PLP1,MRPS25,MRPS9,MRPL15,CILP,FBN2,MRPS5,TUBB6,MFAP5,MRPL33
NDUFAB1,NDUFB4,NDUFB2,NDUFC1,NDUFB3,NDUFA1,NDUFB10,NDUFB9,NDUFB6,NDUFS5,NDUFB1,NDUFA12,NDUFA6,NDUFA4
NDUFAB1,ME1,PYGM,ME2,HMOX2,STC2,SDHB,MARC2,STEAP4,COQ10A,SUCLG1,UQCRFS1,IDH2,UQCRFS1P1
ME1,ME2,AKR1B1,SDHB,COX6B1,STEAP4,COX7B,UQCRB,COX6C,UQCRFS1,NDUFA12,NDUFA4,UQCRFS1P1
MRPS33,MRPL27,MRPL51,MRPL37,MRPS15,MRPS25,MRPS9,MRPL15,MRPS5,MRPL33
COX6B1,COX7B,ATP5MC3,ATP5PF,ATP5MC1,COX6C,ATP5F1C,ATP2A2,NDUFA4,ATP5PO

Table S7. Correlation analysis between DCM-related genes with clinical and echocardiographic parameters in function of severe functional status (NYHA-class III)

PARAMETER	MYH6	NPPA	MT-RNR1	NEAT1
		CORRELATION		
**BMI**	-0,21	0,13	-0,43	-0,51
**Hemoglobin (mg/ml)**	-0,35	0,05	0,25	-0,4
**Hematocrit (%)**	-0,35	-0,02	0,25	-0,47
**Total cholesterol (mg/dl)**	0,19	-0,02	0,66	-0,31
**EF (%)**	-0,11	-0,08	-0,1	-0,39
**LVEDD(mm)**	0,12	0,51	0,25	0,73
**LVESD (mm)**	-0,34	0,96	-0,34	0,59
		P-VALUE		
**BMI**	0,589	0,865	0,371	0,183
**Hemoglobin (mg/ml)**	0,492	0,781	0,677	0,464
**Hematocrit (%)**	0,437	0,973	0,590	0,286
**Total cholesterol (mg/dl)**	0,616	0,916	0,137	0,556
**EF (%)**	0,775	0,795	0,878	0,323
**LVEDD(mm)**	0,726	0,175	0,700	0,049
**LVESD (mm)**	0,461	0,000	0,460	0,160

Abbreviations: *EF=Ejection Fraction; LVEDD=Left Ventricular End Diastolic Diameter; LVESD=Left Ventricular End Systolic Diameter*
